# Supplementary material for: Cost-effectiveness of psychological treatments for post-traumatic stress disorder in adults
Source: PLoS One. 2020 Apr 30;15(4):e0232245. doi: 10.1371/journal.pone.0232245 (PMC7192458; doi:10.1371/journal.pone.0232245)
Supplement: S1 File — (DOCX) [file pone.0232245.s001.docx]

# **Selection of effectiveness data and transformation for use in the economic analysis**

Effectiveness data were obtained from a systematic literature review and network meta-analyses (NMAs) of randomised clinical trials (RCTs) of psychological treatments for adults with PTSD [1]. The NMAs were conducted within a Bayesian framework using Markov Chain Monte Carlo simulation techniques implemented in WinBUGS 1.4.3 [2, 3].

The NMAs included 2 analyses of changes in PTSD symptom scores (between baseline and treatment endpoint; and between baseline and 1-4 month follow-up) and one analysis of dichotomous remission data at treatment endpoint. Although dichotomous data are more suitable for use in economic modelling as they can be easily translated into probabilities of events that correspond directly to the model health states, available dichotomous remission data were more limited and covered a more limited range of interventions of interest in the economic analysis (34 RCTs assessing 16 treatment options reported dichotomous remission at treatment endpoint; in contrast, continuous PTSD symptom change score data between baseline and treatment endpoint were available for 19 treatment options in 71 RCTs). Therefore, the economic analysis utilised the results of the NMAs of changes in PTSD symptom scores; these NMAs were conducted using a generalised linear model (GLM) with a normal likelihood and identity link [4,5]. Results were estimated in the form of standardised mean differences (SMDs), and were subsequently transformed into log-odds ratios of effect [6], so that they could be utilised in the economic analysis, as described in a related publication [1] (see Appendix S3 of the related publication).

The log-odds ratios of remission of each intervention versus no treatment (which served as the baseline treatment) were exponentiated into odds ratios. Subsequently, the probability of remission for each intervention, which was utilised in the economic model, was estimated using the following formulae:

$intervention prob= \frac{odds}{\left( 1+odds \right)}$ (1)

and

$odds= \frac{baseline prob}{\left( 1-baseline prob \right)} OR$(2)

where baseline prob is the probability of remission for the baseline treatment (no treatment), OR is the odds ratio of remission for each intervention versus no treatment as estimated following exponentiation of the log-odds ratios obtained from the NMA, and odds is the odds of each intervention to achieve remission.

The NMA models were run in WinBUGS with an initial burn-in period of 100,000 iterations, followed by 300,000 further iterations, thinned by 30 so as to obtain 10,000 iterations for use in the economic model. These 10,000 samples are representative of the full posterior distributions, and thus the uncertainty in the input estimates is incorporated in the economic model.

The raw data utilised in the NMAs that informed the economic analysis are provided in Tables 1 and 2. Table 3 provides full references to the studies included in the NMAs.

Table 1. Data included in the network meta-analysis of changes in PTSD symptom scores between baseline and treatment endpoint

| t[,1] | y[,1] | sd[,1] | n[,1] | t[,2] | y[,2] | sd[,2] | n[,2] | t[,3] | y[,3] | sd[,3] | n[,3] | t[,4] | y[,4] | sd[,4] | n[,4] | na[] | #Study |
| --- | --- | --- | --- | --- | --- | --- | --- | --- | --- | --- | --- | --- | --- | --- | --- | --- | --- |
| 1 | -2.00 | 9.72 | 24 | 5 | -11.20 | 10.36 | 27 | 6 | -23.10 | 9.47 | 27 | NA | NA | NA | NA | 3 | #Blanchard 2002/2003/2004 |
| 1 | -5.00 | 8.96 | 16 | 6 | -7.69 | 10.53 | 15 | NA | NA | NA | NA | NA | NA | NA | NA | 2 | #Difede 2007b |
| 1 | 0.00 | 5.60 | 11 | 6 | -5.77 | 6.10 | 12 | NA | NA | NA | NA | NA | NA | NA | NA | 2 | #Dunne 2012 |
| 1 | -1.40 | 5.56 | 14 | 6 | -22.10 | 5.89 | 14 | NA | NA | NA | NA | NA | NA | NA | NA | 2 | #Ehlers 2005 |
| 1 | -2.10 | 7.68 | 10 | 6 | -34.55 | 6.55 | 20 | NA | NA | NA | NA | NA | NA | NA | NA | 2 | #Zang 2014 |
| 1 | -0.58 | 2.98 | 17 | 6 | -6.65 | 2.74 | 17 | NA | NA | NA | NA | NA | NA | NA | NA | 2 | #Alghamdi 2015 |
| 1 | -0.10 | 0.35 | 48 | 6 | 0.00 | 0.46 | 52 | 18 | -0.10 | 0.40 | 62 | 19 | 0.00 | 0.47 | 55 | 4 | #Buhmann 2016 |
| 1 | 0.18 | 18.63 | 27 | 6 | -50.03 | 16.93 | 28 | NA | NA | NA | NA | NA | NA | NA | NA | 2 | #Chard 2005 |
| 1 | -15.00 | 19.13 | 24 | 6 | -40.00 | 18.71 | 22 | NA | NA | NA | NA | NA | NA | NA | NA | 2 | #Cloitre 2002 |
| 1 | -6.47 | 17.48 | 31 | 6 | -18.37 | 19.42 | 22 | NA | NA | NA | NA | NA | NA | NA | NA | 2 | #Falsetti 2008 |
| 1 | -1.10 | 6.50 | 14 | 6 | -5.80 | 7.48 | 14 | NA | NA | NA | NA | NA | NA | NA | NA | 2 | #Jung 2013 |
| 1 | -3.22 | 6.22 | 30 | 5 | -14.28 | 9.48 | 30 | 6 | -23.05 | 7.30 | 31 | NA | NA | NA | NA | 3 | #Ehlers 2014 |
| 1 | -2.87 | 8.16 | 24 | 6 | -12.50 | 7.10 | 25 | NA | NA | NA | NA | NA | NA | NA | NA | 2 | #Hollifield 2007 |
| 1 | -2.70 | 16.86 | 10 | 6 | -33.40 | 21.16 | 10 | NA | NA | NA | NA | NA | NA | NA | NA | 2 | #Fecteau 1999 |
| 1 | -0.29 | 0.65 | 66 | 6 | -0.60 | 1.11 | 101 | NA | NA | NA | NA | NA | NA | NA | NA | 2 | #Bolton 2014a |
| 1 | -1.00 | 2.95 | 10 | 6 | -8.00 | 3.85 | 10 | NA | NA | NA | NA | NA | NA | NA | NA | 2 | #Lindauer 2008 |
| 1 | -6.50 | 12.83 | 23 | 6 | -16.80 | 19.64 | 29 | 9 | -20.50 | 14.98 | 22 | NA | NA | NA | NA | 3 | #McDonagh 2005 |
| 1 | -3.46 | 8.16 | 23 | 6 | -12.85 | 7.54 | 41 | NA | NA | NA | NA | NA | NA | NA | NA | 2 | #Pacella 2012 |
| 6 | -19.03 | 7.96 | 89 | 18 | -23.12 | 6.81 | 23 | 19 | -20.94 | 7.16 | 26 | NA | NA | NA | NA | 3 | #Popiel 2015 |
| 18 | 0.40 | 10.10 | 31 | 19 | -5.90 | 7.09 | 34 | NA | NA | NA | NA | NA | NA | NA | NA | 2 | #Rothbaum 2006 |
| 6 | -8.1 | 9.346 | 10 | 8 | -30.36 | 12.45 | 11 | NA | NA | NA | NA | NA | NA | NA | NA | 2 | #Capezzani 2013 |
| 5 | -6.3 | 4.885 | 11 | 6 | -10.38 | 8.05 | 10 | 7 | -13.41 | 4.49 | 14 | NA | NA | NA | NA | 3 | #Foa 1991 |
| 5 | -12.06 | 13.86 | 15 | 6 | -15.18 | 12.90 | 27 | NA | NA | NA | NA | NA | NA | NA | NA | 2 | #Cottraux 2008 |
| 5 | -25.4 | 8.995 | 38 | 6 | -22.70 | 8.70 | 33 | NA | NA | NA | NA | NA | NA | NA | NA | 2 | #Cloitre 2010 |
| 5 | -2.5 | 15.48 | 11 | 6 | -18.30 | 15.57 | 10 | NA | NA | NA | NA | NA | NA | NA | NA | 2 | #Katz 2014 |
| 5 | -3.38 | 13.78 | 42 | 6 | -24.37 | 11.04 | 42 | NA | NA | NA | NA | NA | NA | NA | NA | 2 | #Castillo 2016 |
| 6 | -29.30 | 10.50 | 47 | 9 | -36.30 | 10.88 | 24 | NA | NA | NA | NA | NA | NA | NA | NA | 2 | #Ghafoori 2017 |
| 4 | -18.50 | 18.87 | 13 | 6 | -43.60 | 17.64 | 17 | 10 | -32.60 | 17.27 | 23 | NA | NA | NA | NA | 3 | #Markowitz 2015a |
| 3 | -7.15 | 11.42 | 131 | 6 | -10.01 | 11.38 | 99 | NA | NA | NA | NA | NA | NA | NA | NA | 2 | #Chambers 2014 |
| 6 | -19.56 | 7.37 | 29 | 18 | -13.43 | 6.90 | 20 | NA | NA | NA | NA | NA | NA | NA | NA | 2 | #Echiverri-Cohen 2016 |
| 1 | 2.19 | 23.02 | 22 | 7 | -14.26 | 26.80 | 21 | NA | NA | NA | NA | NA | NA | NA | NA | 2 | #Davis 2007 |
| 1 | -3.48 | 8.76 | 41 | 7 | -12.60 | 7.41 | 39 | NA | NA | NA | NA | NA | NA | NA | NA | 2 | #Krakow 2000 |
| 1 | -3.47 | 20.70 | 23 | 7 | -15.54 | 20.70 | 24 | NA | NA | NA | NA | NA | NA | NA | NA | 2 | #Davis 2011 |
| 1 | -6.20 | 15.42 | 45 | 7 | -23.60 | 16.97 | 48 | 9 | -22.20 | 15.10 | 53 | NA | NA | NA | NA | 3 | #Ford 2011 |
| 2 | -0.20 | 11.16 | 27 | 7 | -5.90 | 11.32 | 33 | NA | NA | NA | NA | NA | NA | NA | NA | 2 | #Nakamura 2017 |
| 1 | -1.40 | 8.18 | 10 | 11 | -32.70 | 12.06 | 10 | NA | NA | NA | NA | NA | NA | NA | NA | 2 | #Wells 2012 |
| 1 | -1.23 | 4.79 | 26 | 8 | -14.72 | 4.41 | 25 | NA | NA | NA | NA | NA | NA | NA | NA | 2 | #Aldahadha 2012 |
| 1 | -2.72 | 11.88 | 14 | 8 | -41.93 | 13.77 | 15 | NA | NA | NA | NA | NA | NA | NA | NA | 2 | #Acarturk 2015 |
| 1 | -3.54 | 13.82 | 49 | 8 | -38.33 | 12.81 | 49 | NA | NA | NA | NA | NA | NA | NA | NA | 2 | #Acarturk 2016 |
| 4 | -8.40 | 12.10 | 12 | 8 | -17.30 | 16.37 | 10 | NA | NA | NA | NA | NA | NA | NA | NA | 2 | #Carlson 1998 |
| 1 | -7.50 | 11.25 | 19 | 8 | -24.60 | 11.43 | 20 | NA | NA | NA | NA | NA | NA | NA | NA | 2 | #Edmond 1999/2004 |
| 1 | -3.35 | 11.51 | 29 | 8 | -14.22 | 12.13 | 18 | NA | NA | NA | NA | NA | NA | NA | NA | 2 | #Yurtsever 2018 |
| 5 | -8.45 | 11.26 | 29 | 8 | -24.64 | 12.30 | 28 | NA | NA | NA | NA | NA | NA | NA | NA | 2 | #Scheck 1998 |
| 7 | -0.11 | 0.41 | 30 | 8 | -0.23 | 0.38 | 32 | NA | NA | NA | NA | NA | NA | NA | NA | 2 | #Ter Heide 2016 |
| 8 | -17.70 | 15.35 | 23 | 12 | -15.80 | 11.20 | 23 | NA | NA | NA | NA | NA | NA | NA | NA | 2 | #Karatzias 2011 |
| 8 | -39.15 | 15.69 | 29 | 18 | -33.23 | 14.66 | 30 | NA | NA | NA | NA | NA | NA | NA | NA | 2 | #van der Kolk 2007 |
| 1 | -5.78 | 12.23 | 16 | 10 | -24.54 | 16.92 | 32 | NA | NA | NA | NA | NA | NA | NA | NA | 2 | #Krupnick 2008 |
| 1 | 0.07 | 0.37 | 38 | 5 | -0.26 | 0.37 | 75 | NA | NA | NA | NA | NA | NA | NA | NA | 2 | #Yeomans 2010 |
| 1 | 0.52 | 7.73 | 25 | 12 | -22.60 | 9.63 | 29 | NA | NA | NA | NA | NA | NA | NA | NA | 2 | #Church 2013/2014 |
| 1 | -13.39 | 30.20 | 74 | 12 | -21.09 | 29.70 | 71 | NA | NA | NA | NA | NA | NA | NA | NA | 2 | #Connolly 2011 |
| 1 | -14.20 | 9.13 | 122 | 12 | -31.90 | 8.43 | 114 | NA | NA | NA | NA | NA | NA | NA | NA | 2 | #Robson 2016 |
| 1 | -0.63 | 6.87 | 19 | 13 | -12.90 | 8.10 | 20 | NA | NA | NA | NA | NA | NA | NA | NA | 2 | #Kent 2011 |
| 2 | -12.95 | 2.51 | 25 | 14 | -3.82 | 1.83 | 27 | NA | NA | NA | NA | NA | NA | NA | NA | 2 | #Bar-Haim 2011/Badura-Brack 2015 study 1 |
| 2 | -8.76 | 2.21 | 24 | 14 | -1.51 | 2.01 | 22 | NA | NA | NA | NA | NA | NA | NA | NA | 2 | #Bar-Haim 2011/Badura-Brack 2015 study 2 |
| 2 | -5.30 | 7.61 | 38 | 14 | -4.90 | 9.09 | 34 | NA | NA | NA | NA | NA | NA | NA | NA | 2 | #Schoorl 2013 |
| 3 | -6.90 | 8.08 | 21 | 15 | -18.68 | 7.99 | 22 | NA | NA | NA | NA | NA | NA | NA | NA | 2 | #Sautter 2015 |
| 1 | -5.68 | 12.12 | 26 | 16 | -23.69 | 10.68 | 28 | NA | NA | NA | NA | NA | NA | NA | NA | 2 | #Ivarsson 2014 |
| 1 | 1.36 | 8.34 | 21 | 16 | -25.34 | 10.50 | 21 | NA | NA | NA | NA | NA | NA | NA | NA | 2 | #Lewis 2017 |
| 1 | -0.48 | 5.97 | 80 | 16 | -10.06 | 8.32 | 79 | NA | NA | NA | NA | NA | NA | NA | NA | 2 | #Knaevelsrud 2015 |
| 1 | -2.85 | 6.38 | 47 | 16 | -7.56 | 6.41 | 47 | NA | NA | NA | NA | NA | NA | NA | NA | 2 | #Knaevelsrud 2017 |
| 16 | -12.50 | 4.40 | 23 | 17 | -12.60 | 5.70 | 28 | NA | NA | NA | NA | NA | NA | NA | NA | 2 | #Littleton 2016 |
| 1 | -15.79 | 14.61 | 14 | 17 | -25.15 | 9.85 | 13 | NA | NA | NA | NA | NA | NA | NA | NA | 2 | #Hirai 2005 |
| 1 | -6.69 | 9.12 | 58 | 17 | -11.26 | 9.37 | 62 | NA | NA | NA | NA | NA | NA | NA | NA | 2 | #Kuhn 2017 |
| 1 | -5.21 | 8.28 | 19 | 17 | -16.00 | 11.81 | 23 | NA | NA | NA | NA | NA | NA | NA | NA | 2 | #Spence 2011 |
| 1 | -2.57 | 6.90 | 29 | 17 | -10.48 | 8.99 | 21 | NA | NA | NA | NA | NA | NA | NA | NA | 2 | #Xu 2016 |
| 1 | -3.56 | 8.74 | 24 | 17 | -6.69 | 7.74 | 25 | NA | NA | NA | NA | NA | NA | NA | NA | 2 | #Miner 2016 |
| 2 | -0.30 | 5.64 | 17 | 17 | -1.32 | 6.43 | 19 | NA | NA | NA | NA | NA | NA | NA | NA | 2 | #Henderson 2007 |
| 2 | -12.30 | 9.10 | 19 | 17 | -17.02 | 10.03 | 42 | NA | NA | NA | NA | NA | NA | NA | NA | 2 | #Truijens 2014 |
| 2 | 1.80 | 4.70 | 23 | 17 | -6.10 | 6.58 | 26 | NA | NA | NA | NA | NA | NA | NA | NA | 2 | #Sloan 2004 |
| 2 | -0.90 | 4.11 | 27 | 17 | -7.54 | 6.72 | 55 | NA | NA | NA | NA | NA | NA | NA | NA | 2 | #Sloan 2007 |
| 2 | -10.20 | 4.77 | 21 | 17 | -8.80 | 5.46 | 21 | NA | NA | NA | NA | NA | NA | NA | NA | 2 | #Sloan 2011 |
| t1, t2, t3, t4 indicate the coded treatment in each trial arm  y1, y2, y3, y4 indicate the mean change in effect in each trial arm  sd1, sd2, sd3, sd4 indicate the standard deviation of the mean change in effect in each trial arm  n1, n2, n3, n4 indicate the number of participants in each trial arm  na indicates number of arms in each trial  NA: non-applicable | | | | | | | | | | | | | | | | | |

Treatment codes: 1. Waitlist; 2. Attention placebo; 3. Psychoeducation; 4. Relaxation; 5. Counselling; 6. TF-CBT; 7. non-TF-CBT; 8. EMDR; 9. Present-centered therapy; 10. IPT; 11. Metacognitive therapy; 12. Combined somatic/cognitive therapies; 13. Resilience-oriented treatment; 14. Attention bias modification; 15. Couple intervention; 16. Self-help with support; 17. Self-help without support; 18. SSRI; 19. TF-CBT + SSRI

*CBT: cognitive behavioural therapy; EMDR: eye movement desensitisation and reprocessing; IPT: interpersonal psychotherapy; SSRI: selective serotonin reuptake inhibitor; TF: trauma-focused*

Table 2. Data included in the network meta-analysis of changes in PTSD symptom scores between baseline and 1-4 month follow-up

| t[,1] | y[,1] | sd[,1] | n[,1] | t[,2] | y[,2] | sd[,2] | n[,2] | t[,3] | y[,3] | sd[,3] | n[,3] | na[] | #Study |
| --- | --- | --- | --- | --- | --- | --- | --- | --- | --- | --- | --- | --- | --- |
| 1 | -3.48 | 10.05 | 41 | 5 | -14.4 | 13.75 | 41 | 12 | -13.55 | 15.26 | 44 | 3 | #van Emmerik 2008 |
| 1 | -0.11 | 0.35 | 22 | 5 | -0.24 | 0.44 | 41 | NA | NA | NA | NA | 2 | #Hijazi 2014 |
| 1 | -5.64 | 11.23 | 38 | 5 | -13.69 | 15.69 | 38 | NA | NA | NA | NA | 2 | #Jacob 2014 |
| 1 | -0.32 | 0.90 | 50 | 5 | -0.91 | 0.38 | 99 | NA | NA | NA | NA | 2 | #Weiss 2015 (study 1) |
| 1 | -0.92 | 0.36 | 64 | 5 | -1.08 | 0.57 | 154 | NA | NA | NA | NA | 2 | #Weiss 2015 (study 2) |
| 1 | -10 | 6.90 | 23 | 5 | -13.47 | 7.93 | 41 | NA | NA | NA | NA | 2 | #Pacella 2012 |
| 5 | -19.74 | 17.72 | 11 | 6 | -2.55 | 12.49 | 10 | NA | NA | NA | NA | 2 | #Hensel-Dittmann 2011 |
| 4 | -14.2 | 10.29 | 26 | 5 | -23.3 | 9.52 | 26 | NA | NA | NA | NA | 2 | #Blanchard 2002/2003/2004 |
| 4 | -22.6 | 8.39 | 38 | 5 | -24.2 | 8.69 | 33 | NA | NA | NA | NA | 2 | #Cloitre 2010 |
| 4 | -21.4 | 9.05 | 111 | 5 | -20.5 | 9.33 | 111 | NA | NA | NA | NA | 2 | #Neuner 2008 |
| 4 | -15.33 | 8.90 | 30 | 5 | -22.29 | 8.09 | 31 | NA | NA | NA | NA | 2 | #Ehlers 2014 |
| 5 | -34.3 | 13.84 | 17 | 8 | -23.1 | 11.85 | 17 | NA | NA | NA | NA | 2 | #McDonagh 2005 |
| 3 | -9.21 | 11.77 | 134 | 5 | -8.95 | 11.17 | 110 | NA | NA | NA | NA | 2 | #Chambers 2014 |
| 2 | -2.6 | 12.22 | 27 | 6 | -9.3 | 11.54 | 33 | NA | NA | NA | NA | 2 | #Nakamura 2017 |
| 6 | -25 | 17.11 | 48 | 8 | -24.4 | 15.53 | 53 | NA | NA | NA | NA | 2 | #Ford 2011 |
| 1 | -2.18 | 14.33 | 49 | 7 | -33.82 | 14.10 | 49 | NA | NA | NA | NA | 2 | #Acarturk 2016 |
| 1 | -3.62 | 10.22 | 29 | 7 | -10.50 | 11.65 | 18 | NA | NA | NA | NA | 2 | #Yurtsever 2018 |
| 6 | -0.14 | 0.41 | 32 | 7 | -0.13 | 0.42 | 31 | NA | NA | NA | NA | 2 | #Ter Heide 2016 |
| 7 | -16.2 | 15.17 | 23 | 9 | -16.8 | 12.08 | 23 | NA | NA | NA | NA | 2 | #Karatzias 2011 |
| 1 | -18.89 | 18.17 | 16 | 10 | -26.63 | 20.54 | 32 | NA | NA | NA | NA | 2 | #Krupnick 2008 |
| 3 | -9.04 | 8.06 | 20 | 11 | -21.3 | 8.05 | 21 | NA | NA | NA | NA | 2 | #Sautter 2015 |
| 1 | -4.7 | 10.37 | 30 | 3 | -7.22 | 11.09 | 29 | NA | NA | NA | NA | 2 | #Ghafoori 2016 |
| 1 | -5.13 | 9.63 | 21 | 12 | -28.52 | 11.18 | 21 | NA | NA | NA | NA | 2 | #Lewis 2017 |
| 12 | -15.8 | 4.53 | 20 | 13 | -16.2 | 4.83 | 21 | NA | NA | NA | NA | 2 | #Littleton 2016 |
| 2 | -0.24 | 5.72 | 17 | 13 | -5.95 | 5.64 | 19 | NA | NA | NA | NA | 2 | #Henderson 2007 |
| 1 | -4.47 | 4.04 | 70 | 14 | -3.66 | 6.56 | 72 | NA | NA | NA | NA | 2 | #Kazak 2004 |
| 1 | -7.3 | 8.97 | 28 | 15 | -16.7 | 9.95 | 31 | NA | NA | NA | NA | 2 | #Basoglu 2005 |
| 1 | -13.2 | 13.45 | 15 | 15 | -32.9 | 14.37 | 16 | NA | NA | NA | NA | 2 | #Basoglu 2007 |
| t1, t2, t3 indicate the coded treatment in each trial arm; y1, y2, y3 indicate the mean change in effect in each trial arm  sd1, sd2, sd3 indicate the standard deviation of the mean change in effect in each trial arm; n1, n2, n3 indicate the number of participants in each trial arm  na indicates number of arms in each trial; NA: non-applicable | | | | | | | | | | | | | |

Treatment codes: 1. Waitlist; 2. Attention placebo; 3. Psychoeducation; 4. Counselling; 5. TF-CBT; 6. non-TF-CBT; 7. EMDR; 8. Present-centered therapy; 9. Combined somatic/cognitive therapies; 10. IPT; 11. Couple intervention; 12. Self-help with support; 13. Self-help without support; 14. Family therapy; 15. Behavioural therapy

*CBT: cognitive behavioural therapy; EMDR: eye movement desensitisation and reprocessing; IPT: interpersonal psychotherapy; TF: trauma-focused*

Table 3. References of studies included in the network meta-analyses that informed the economic analysis

| 1 | Acarturk 2015 | Acarturk C, Konuk E, Cetinkaya M et al. (2015) EMDR for Syrian refugees with posttraumatic stress disorder symptoms: Results of a pilot randomized controlled trial. European Journal of Psychotraumatology 6(1), 27414 |
| --- | --- | --- |
| 2 | Acarturk 2016 | Acarturk C, Konuk E, Cetinkaya M, et al. (2016) The efficacy of eye movement desensitization and reprocessing for post-traumatic stress disorder and depression among Syrian refugees: Results of a randomized controlled trial. Psychological medicine 46(12), 2583-93 |
| 3 | Aldahadha 2012 | Aldahadha B, Al-Harthy H and Sulaiman S (2012) The efficacy of eye movement desensitization reprocessing in resolving the trauma caused by the road accidents in the Sultanate of Oman. Journal of Instructional Psychology 39(3/4), 146 |
| 4 | Alghamdi 2015 | Alghamdi M, Hunt N and Thomas S (2015) The effectiveness of Narrative Exposure Therapy with traumatised firefighters in Saudi Arabia: A randomized controlled study. Behaviour Research and Therapy 66, 64-71 |
| 5 | Bar-Haim 2011/Badura-Brack 2015 study 1 | Bar-Haim Y and Fruchter E (2011) Attention Bias Modification Treatment for Patients With Post Traumatic Stress Disorder (PTSD) [NCT01368302]. Available from: https://clinicaltrials.gov/ct2/show/NCT01368302 [accessed 26.07.2017]  Badura-Brack AS, Naim R, Ryan TJ, et al. (2015) Effect of attention training on attention bias variability and PTSD symptoms: randomized controlled trials in Israeli and US combat veterans. American journal of psychiatry 172(12), 1233-41 |
| 6 | Bar-Haim 2011/Badura-Brack 2015 study 2 | Bar-Haim Y and Fruchter E (2011) Attention Bias Modification Treatment for Patients With Post Traumatic Stress Disorder (PTSD) [NCT01368302]. Available from: https://clinicaltrials.gov/ct2/show/NCT01368302 [accessed 26.07.2017]  Badura-Brack AS, Naim R, Ryan TJ, et al. (2015) Effect of attention training on attention bias variability and PTSD symptoms: randomized controlled trials in Israeli and US combat veterans. American journal of psychiatry 172(12), 1233-41 |
| 7 | Basoglu 2005 | Basoglu M, Salcioglu E and Livanou M (2005) Single-session behavioural treatment of earthquake-related posttraumatic stress disorder: a randomised waiting list controlled trial, Journal of Traumatic Stress 18, 1-11 |
| 8 | Basoglu 2007 | Başoğlu M, Şalcioğlu E and Livanou M (2007) A randomized controlled study of single-session behavioural treatment of earthquake-related post-traumatic stress disorder using an earthquake simulator. Psychological medicine 37(2), 203-13 |
| 9 | Blanchard 2002/2003/2004 | Blanchard EB (2002) Treatment-related changes in cardiovascular reactivity to trauma cues in motor vehicle accident-related PTSD. Behaviour Therapy 33, 417-426  Blanchard EB, Hickling EJ, Devineni T, et al. (2003) A controlled evaluation of cognitive behaviorial therapy for posttraumatic stress in motor vehicle accident survivors. Behaviour Research & Therapy 41, 79-96  Blanchard EB, Hickling EJ, Malta LS, et al. (2004) One-and two-year prospective follow-up of cognitive behavior therapy or supportive psychotherapy. Behaviour research and therapy 42(7), 745-59 |
| 10 | Bolton 2014a | Bolton P, Bass JK, Zangana GA, et al. (2014) A randomized controlled trial of mental health interventions for survivors of systematic violence in Kurdistan, Northern Iraq. BMC psychiatry 14(1), 360 |
| 11 | Buhmann 2016 | Buhmann CB, Nordentoft M, Ekstroem M, et al. (2016) The effect of flexible cognitive–behavioural therapy and medical treatment, including antidepressants on post-traumatic stress disorder and depression in traumatised refugees: pragmatic randomised controlled clinical trial. The British Journal of Psychiatry 208(3), 252-9 |
| 12 | Capezzani 2013 | Capezzani L, Ostacoli L, Cavallo M, et al. (2013) EMDR and CBT for cancer patients: Comparative study of effects on PTSD, anxiety, and depression. Journal of EMDR Practice and Research 7(3), 134-43 |
| 13 | Carlson 1998 | Carlson JG, Chemtob CM, Rusnak K, et al. (1998) Eye movement desensitization and reprocessing (EDMR) treatment for combat‐related posttraumatic stress disorder. Journal of Traumatic Stress 11(1), 3-24 |
| 14 | Castillo 2016 | Castillo DT, Chee CL, Nason E, et al. (2016) Group-delivered cognitive/exposure therapy for PTSD in women veterans: A randomized controlled trial. Psychological trauma: theory, research, practice, and policy 8(3), 404 |
| 15 | Chambers 2014 | Chambers SK, Girgis A, Occhipinti S, et al. (2014) A randomized trial comparing two low-intensity psychological interventions for distressed patients with cancer and their caregivers. InOncology nursing forum 41(4), p.E257 |
| 16 | Chard 2005 | Chard KM (2005) An evaluation of cognitive processing therapy for the treatment of posttraumatic stress disorder related to childhood sexual abuse. Journal of consulting and clinical psychology 73(5), 965 |
| 17 | Church 2013/2014 | Church D, Hawk C, Brooks AJ, et al. (2013) Psychological trauma symptom improvement in veterans using emotional freedom techniques: a randomized controlled trial. The Journal of nervous and mental disease 201(2), 153-60  Church D (2014) Reductions in pain, depression, and anxiety symptoms after PTSD remediation in veterans. Explore: The Journal of Science and Healing 10(3), 162-9 |
| 18 | Cloitre 2002 | Cloitre M, Koenen KC, Cohen LR and Han H (2002) Skills training in affective and interpersonal regulation followed by exposure: a phase-based treatment for PTSD related to childhood abuse. Journal of consulting and clinical psychology 70(5), 1067 |
| 19 | Cloitre 2010 | Cloitre M, Stovall-McClough KC, Nooner K, et al. (2010) Treatment for PTSD related to childhood abuse: A randomized controlled trial. American journal of psychiatry 167(8), 915-24 |
| 20 | Connolly 2011 | Connolly S and Sakai C (2011) Brief trauma intervention with Rwandan genocide-survivors using Thought Field Therapy. International Journal of Emergency Mental Health 13(3), 161 |
| 21 | Cottraux 2008 | Cottraux J, Note I, Yao SN, et al. (2008) Randomized controlled comparison of cognitive behavior therapy with Rogerian supportive therapy in chronic post-traumatic stress disorder: A 2-year follow-up. Psychotherapy and psychosomatics 77(2), 101-10 |
| 22 | Davis 2007 | Davis JL and Wright DC (2007) Randomized clinical trial for treatment of chronic nightmares in trauma‐exposed adults. Journal of Traumatic Stress 20(2), 123-33 |
| 23 | Davis 2011 | Davis JL, Rhudy JL, Pruiksma KE, et al. (2011) Physiological predictors of response to exposure, relaxation, and rescripting therapy for chronic nightmares in a randomized clinical trial. Journal of clinical sleep medicine: JCSM: official publication of the American Academy of Sleep Medicine 7(6), 622 |
| 24 | Difede 2007b | Difede J, Malta LS, Best S, et al. (2007) A randomized controlled clinical treatment trial for World Trade Center attack-related PTSD in disaster workers. The Journal of nervous and mental disease 195(10), 861-5 |
| 25 | Dunne 2012 | Dunne RL, Kenardy J and Sterling M (2012) A randomized controlled trial of cognitive-behavioral therapy for the treatment of PTSD in the context of chronic whiplash. The Clinical journal of pain 28(9), 755-65 |
| 26 | Echiverri-Cohen 2016 | Echiverri-Cohen A, Zoellner LA, Gallop R, et al. (2016) Changes in temporal attention inhibition following prolonged exposure and sertraline in the treatment of PTSD. Journal of consulting and clinical psychology 84(5), 415 |
| 27 | Edmond 1999/2004 | Edmond T, Rubin A and Wambach K (1999) The effectiveness of EMDR with adult female survivors of childhood sexual abuse. Social Work Research 23, 103-116  Edmond T and Rubin A (2004) Assessing the long-term effects of EMDR: Results from an 18-month follow-up study with adult female survivors of CSA. Journal of child sexual abuse 13(1), 69-86 |
| 28 | Ehlers 2005 | Ehlers A, Clark DM, Hackmann A, et al. (2005) Cognitive therapy for post-traumatic stress disorder: development and evaluation. Behaviour research and therapy 43(4), 413-31 |
| 29 | Ehlers 2014 | Ehlers A, Hackmann A, Grey N, et al. (2014) A randomized controlled trial of 7-day intensive and standard weekly cognitive therapy for PTSD and emotion-focused supportive therapy. American Journal of Psychiatry 171(3), 294-304 |
| 30 | Falsetti 2008 | Falsetti SA, Resnick HS and Davis JL (2008) Multiple channel exposure therapy for women with PTSD and comorbid panic attacks. Cognitive Behaviour Therapy 37(2), 117-30 |
| 31 | Fecteau 1999 | Fecteau G and Nicki R (1999) Cognitive behavioural treatment of post traumatic stress disorder after motor vehicle accident. Behavioural & Cognitive Psychotherapy 27, 201-214 |
| 32 | Foa 1991 | Foa EB, Rothbaum BO, Riggs DS and Murdock TB (1991) Treatment of posttraumatic stress disorder in rape victims: a comparison between cognitive-behavioral procedures and counseling. Journal of Consulting & Clinical Psychology 59, 715-723 |
| 33 | Ford 2011 | Ford JD, Steinberg KL and Zhang W (2011) A randomized clinical trial comparing affect regulation and social problem-solving psychotherapies for mothers with victimization-related PTSD. Behavior Therapy 42(4), 560-78 |
| 34 | Ghafoori 2016 | Ghafoori B, Fisher D, Korosteleva O and Hong M (2016) A Randomized, Controlled Pilot Study of a Single-Session Psychoeducation Treatment for Urban, Culturally Diverse, Trauma-Exposed Adults. The Journal of nervous and mental disease 204(6), 421-30 |
| 35 | Ghafoori 2017 | Ghafoori B, Hansen MC, Garibay E and Korosteleva O (2017) Feasibility of training frontline therapists in prolonged exposure: a randomized controlled pilot study of treatment of complex trauma in diverse victims of crime and violence. The Journal of nervous and mental disease 205(4), 283-93 |
| 36 | Henderson 2007 | Henderson P, Rosen D and Mascaro N (2007) Empirical study on the healing nature of mandalas. Psychology of Aesthetics, Creativity, and the Arts 1(3), 148 |
| 37 | Hensel-Dittmann 2011 | Hensel-Dittmann D, Schauer M, Ruf M, et al. (2011) The treatment of traumatized victims of war and torture: a randomized controlled comparison of Narrative Exposure Therapy and Stress Inoculation Training. Psychotherapy and Psychosomatics 80, 345-352 [DOI: 10.1159/000327253] |
| 38 | Hijazi 2014 | Hijazi AM, Lumley MA, Ziadni MS, et al. (2014) Brief Narrative Exposure Therapy for Posttraumatic Stress in Iraqi Refugees: A Preliminary Randomized Clinical Trial. J. Traum. Stress 27, 314–322 [doi: 10.1002/jts.21922] |
| 39 | Hirai 2005 | Hirai M and Clum GA (2005) An Internet‐based self‐change program for traumatic event related fear, distress, and maladaptive coping. Journal of traumatic stress 2005 18(6), 631-6 |
| 40 | Hollifield 2007 | Hollifield M, Sinclair-Lian N, Warner TD and Hammerschlag R (2007) Acupuncture for posttraumatic stress disorder: a randomized controlled pilot trial. The Journal of nervous and mental disease 195(6), 504-13 |
| 41 | Ivarsson 2014 | Ivarsson D, Blom M, Hesser H, et al. (2014) Guided internet-delivered cognitive behavior therapy for post-traumatic stress disorder: a randomized controlled trial. Internet interventions 1(1), 33-40 |
| 42 | Jacob 2014 | Jacob N, Neuner F, Mädl A, et al. (2014) Dissemination of psychotherapy for trauma-spectrum disorders in resource-poor countries: a randomized controlled trial in Rwanda. Psychotherapy & Psychosomatics 83, 354–363 [DOI:10.1159/000365114] |
| 43 | Jung 2013 | Jung K and Steil R (2013) A randomized controlled trial on cognitive restructuring and imagery modification to reduce the feeling of being contaminated in adult survivors of childhood sexual abuse suffering from posttraumatic stress disorder. Psychotherapy and psychosomatics 82(4), 213-20 |
| 44 | Karatzias 2011 | Karatzias T, Power K, Brown K, et al. (2011) A controlled comparison of the effectiveness and efficiency of two psychological therapies for posttraumatic stress disorder: eye movement desensitization and reprocessing vs. emotional freedom techniques. The Journal of nervous and mental disease 199(6), 372-8 |
| 45 | Katz 2014 | Katz LS, Douglas S, Zaleski K, et al. (2014) Comparing holographic reprocessing and prolonged exposure for women veterans with sexual trauma: A pilot randomized trial. Journal of Contemporary Psychotherapy 44(1), 9-19 |
| 46 | Kazak 2004 | Kazak AE, Alderfer MA, Streisand R, et al (2004) Treatment of posttraumatic stress symptoms in adolescent survivors of childhood cancer and their families: A randomized clinical trial. Journal of Family Psychology 18(3), 493-504 |
| 47 | Kent 2011 | Kent M, Davis MC, Stark SL and Stewart LA (2011) A resilience‐oriented treatment for posttraumatic stress disorder: Results of a preliminary randomized clinical trial. Journal of traumatic stress 24(5), 591-5 |
| 48 | Knaevelsrud 2015 | Knaevelsrud C, Brand J, Lange A, et al. (2015) Web-based psychotherapy for posttraumatic stress disorder in war-traumatized Arab patients: randomized controlled trial. Journal of medical Internet research17(3) |
| 49 | Knaevelsrud 2017 | Knaevelsrud C, Böttche M, Pietrzak RH, et al. (2017) Efficacy and Feasibility of a Therapist-Guided Internet-Based Intervention for Older Persons with Childhood Traumatization: A Randomized Controlled Trial. The American Journal of Geriatric Psychiatry |
| 50 | Krakow 2000 | Krakow B, Hollifield M, Schrader R, et al.(2000) A controlled study of imagery rehearsal for chronic nightmares in sexual assault survivors with PTSD: a preliminary report. J Trauma Stress 13(4), 589-609 |
| 51 | Krupnick 2008 | Krupnick JL, Green BL, Stockton P, et al. (2008) Group interpersonal psychotherapy for low-income women with posttraumatic stress disorder. Psychotherapy Research 18(5), 497-507 |
| 52 | Kuhn 2017 | Kuhn E, Kanuri N, Hoffman JE, et al. (2017) A randomized controlled trial of a smartphone app for posttraumatic stress disorder symptoms. Journal of consulting and clinical psychology 85(3), 267 |
| 53 | Lewis 2017 | Lewis CE, Farewell D, Groves V, et al. (2017) Internet‐based guided self‐help for posttraumatic stress disorder (ptsd): Randomized controlled trial. Depression and anxiety 34(6), 555-65 |
| 54 | Lindauer 2008 | Lindauer RJ, Booij J, Habraken JB, et al. (2008) Effects of psychotherapy on regional cerebral blood flow during trauma imagery in patients with post-traumatic stress disorder: a randomized clinical trial. Psychological medicine 38(4), 543-54 |
| 55 | Littleton 2016 | Littleton H, Grills AE, Kline KD, et al. (2016) The From Survivor to Thriver program: RCT of an online therapist-facilitated program for rape-related PTSD. Journal of anxiety disorders 43, 41-51 |
| 56 | Markowitz 2015a | Markowitz JC, Petkova E, Neria Y, et al. (2015) Is exposure necessary? A randomized clinical trial of interpersonal psychotherapy for PTSD. American Journal of Psychiatry 172(5), 430-40 |
| 57 | McDonagh 2005 | McDonagh A, Friedman M, McHugo G, et al. (2005) Randomized trial of cognitive-behavioral therapy for chronic posttraumatic stress disorder in adult female survivors of childhood sexual abuse. Journal of consulting and clinical psychology 73(3), 515 |
| 58 | Miner 2016 | Miner A, Kuhn E, Hoffman JE, et al. (2016) Feasibility, acceptability, and potential efficacy of the PTSD Coach app: A pilot randomized controlled trial with community trauma survivors. Psychological Trauma: Theory, Research, Practice, and Policy 8(3), 384 |
| 59 | Nakamura 2017 | Nakamura Y, Lipschitz DL, Donaldson GW, et al. (2017) Investigating Clinical Benefits of a Novel Sleep-Focused Mind-Body Program on Gulf War Illness Symptoms: A Randomized Controlled Trial. Psychosomatic medicine 79(6), 706-18 |
| 60 | Neuner 2008 | Neuner F, Onyut PL, Ertl V, et al. (2008) Treatment of posttraumatic stress disorder by trained lay counselors in an African refugee settlement. A randomized controlled trial. J Consult Clin Psychol 76, 686-694 |
| 61 | Pacella 2012 | Pacella ML, Armelie A, Boarts J, et al. (2012) The impact of prolonged exposure on PTSD symptoms and associated psychopathology in people living with HIV: A randomized test of concept. AIDS and Behavior 16(5), 1327-40 |
| 62 | Popiel 2015 | Popiel A, Zawadzki B, Pragłowska E and Teichman Y (2015) Prolonged exposure, paroxetine and the combination in the treatment of PTSD following a motor vehicle accident. A randomized clinical trial–The “TRAKT” study. Journal of behavior therapy and experimental psychiatry 48, 17-26 |
| 63 | Robson 2016 | Robson R, Robson P, Ludwig R, et al. (2016) Effectiveness of Thought Field Therapy Provided by Newly Instructed Community Workers to a Traumatized Population in Uganda: A Randomized Trial. Current Research in Psychology 1, 1-11 |
| 64 | Rothbaum 2006 | Rothbaum BO, Cahill SP, Foa EB, et al. (2006) Augmentation of sertraline with prolonged exposure in the treatment of posttraumatic stress disorder. Journal of traumatic stress 19(5), 625-38 |
| 65 | Sautter 2015 | Sautter FJ, Glynn SM, Cretu JB, et al. (2015) Efficacy of structured approach therapy in reducing PTSD in returning veterans: A randomized clinical trial. Psychological services12(3), 199 |
| 66 | Scheck 1998 | Scheck MM, Schaeffer JA and Gillette C (1998) Brief psychological intervention with traumatized young women: The efficacy of eye movement desensitization and reprocessing. Journal of traumatic stress 11(1), 25-44 |
| 67 | Schoorl 2013 | Schoorl M, Putman P and van Der Does W (2013) Attentional bias modification in posttraumatic stress disorder: a randomized controlled trial. Psychotherapy and psychosomatics 82(2), 99-105 |
| 68 | Sloan 2004 | Sloan DM and Marx BP (2004) A closer examination of the structured written disclosure procedure. Journal of consulting and clinical psychology 72(2), 165 |
| 69 | Sloan 2007 | Sloan DM, Marx BP and Epstein EM. (2007) Does altering the writing instructions influence outcome associated with written disclosure? Behavior therapy 38(2), 155-68 |
| 70 | Sloan 2011 | Sloan DM, Marx BP and Greenberg EM (2011) A test of written emotional disclosure as an intervention for posttraumatic stress disorder. Behaviour Research and Therapy 49(4), 299-304 |
| 71 | Spence 2011 | Spence J, Titov N, Dear BF, et al. (2011) Randomized controlled trial of Internet‐delivered cognitive behavioral therapy for posttraumatic stress disorder. Depression and anxiety 28(7), 541-50 |
| 72 | Ter Heide 2016 | Ter Heide FJ, Mooren TM, van de Schoot R, et al. (2016) Eye movement desensitisation and reprocessing therapy v. stabilisation as usual for refugees: Randomised controlled trial. The British Journal of Psychiatry 209(4), 311-318 |
| 73 | Truijens 2014 | Truijens FL and van Emmerik AA (2014) Visual feedback in written imaginal exposure for posttraumatic stress: a preliminary study. Journal of Loss and Trauma 19(5), 403-15 |
| 74 | van der Kolk 2007 | Van der Kolk B, Spinazzola J, Blaustein M, et al. (2007) A randomized clinical trial of EMDR, fluoxetine and pill placebo in the treatment of PTSD: Treatment effects and long-term maintenance. Journal of Clinical Psychiatry 68(1), 37-46 |
| 75 | van Emmerik 2008 | Van Emmerik AA, Kamphuis JH and Emmelkamp PM (2008) Treating acute stress disorder and posttraumatic stress disorder with cognitive behavioral therapy or structured writing therapy: a randomized controlled trial. Psychotherapy and psychosomatics 77(2), 93-100 |
| 76 | Weiss 2015 (study 1) | Weiss WM, Murray LK, Zangana GA, et al. (2015) Community-based mental health treatments for survivors of torture and militant attacks in Southern Iraq: a randomized control trial. BMC psychiatry 15(1), 249 |
| 77 | Weiss 2015 (study 2) | Weiss WM, Murray LK, Zangana GA, et al. (2015) Community-based mental health treatments for survivors of torture and militant attacks in Southern Iraq: a randomized control trial. BMC psychiatry 15(1), 249 |
| 78 | Wells 2012 | Wells A and Colbear JS (2012) Treating posttraumatic stress disorder with metacognitive therapy: A preliminary controlled trial. Journal of Clinical Psychology 68(4), 373-81 |
| 79 | Xu 2016 | Xu W, Wang J, Wang Z, et al. (2016) Web-based intervention improves social acknowledgement and disclosure of trauma, leading to a reduction in posttraumatic stress disorder symptoms. Journal of health psychology 21(11), 2695-708 |
| 80 | Yeomans 2010 | Yeomans PD, Forman EM, Herbert JD and Yuen E (2010) A randomized trial of a reconciliation workshop with and without PTSD psychoeducation in Burundian sample. Journal of traumatic stress 23(3), 305-12 |
| 81 | Yurtsever 2018 | Yurtsever, A., Konuk, E., Akyüz, T., Zat, Z., Tükel, F., Çetinkaya, M et al. (2018). An Eye Movement Desensitization and Reprocessing (EMDR) Group Intervention for Syrian Refugees With Post-traumatic Stress Symptoms: Results of a Randomized Controlled Trial. Frontiers in psychology, 9. |
| 82 | Zang 2014 | Zang Y, Hunt N and Cox T (2014) Adapting narrative exposure therapy for Chinese earthquake survivors: A pilot randomised controlled feasibility study. BMC psychiatry 14(1), 1.v |

**References**

1. Mavranezouli I, Megnin-Viggars O, Daly C, Dias S, Welton N, Stockton S, et al. Psychological treatments for post-traumatic stress disorder in adults: a network meta-analysis. Psychol Med. 2020; 50(4):542-55..
2. Lunn DJ, Thomas A, Best N, Spiegelhalter D. WinBUGS-A Bayesian modelling framework: Concepts, structure, and extensibility. Stat Comput. 2000;10(4):325-37.
3. Spiegelhalter D, Thomas A, Best N, Lunn DJ. WinBUGS user manual: version 1.4. Cambridge: MRC Biostatistics Unit; 2003.
4. Dias S, Sutton AJ, Ades AE, Welton NJ. Evidence synthesis for decision making 2: a generalized linear modeling framework for pairwise and network meta-analysis of randomized controlled trials. Med Decis Making. 2013; 33(5):607-17.
5. Dias S, Ades AE, Welton NJ, Jansen JP, Sutton AJ. Network Meta-analysis for Decision-Making. Wiley: Hoboken NJ; 2018.
6. Chinn S. A simple method for converting an odds ratio to effect size for use in meta-analysis. Stat Med. 2000;19(22):3127-31.
