# Supplementary material for: Cost-effectiveness of psychological treatments for post-traumatic stress disorder in adults
Source: PLoS One. 2020 Apr 30;15(4):e0232245. doi: 10.1371/journal.pone.0232245 (PMC7192458; doi:10.1371/journal.pone.0232245)
Supplement: S2 File — (DOCX) [file pone.0232245.s002.docx]

# **Estimation of the baseline probability of remission**

A number of studies that reported the probability of remission over time in adults with PTSD were identified [1-8].

Three of the studies were survey-based studies of the long-term course of PTSD in the community: Breslau *et al.* [1] estimated the impact of specific type of trauma experienced in the community, by interviewing a representative sample of 2,181 people aged 18-45 years living in the Detroit area, US. The study provided survival curves showing the rates of remission over time (up to 10 years) for 180 people diagnosed with PTSD by gender and trauma type (event to self or event to others). Chapman *et al.* [2] reported remission rates from post-traumatic stress disorder in the general population, using data obtained from 8,841 respondents of the 2007 Australian National Survey of Mental Health and Wellbeing, aged 16-85 years, 664 of whom had experienced PTSD at some point in their life. The study reported remission rates over time and also provided a survival curve of remission up to 60 years from onset of PTSD in the surveyed population. Rosellini *et al.* [6] reported remission data from 1575 respondents with PTSD who participated in 22 World Mental Health surveys. Rates of remission were reported for a period of 120 months (10 years) following PTSD onset, which was the longest follow-up period for which a sufficient number of cases were observed for stable estimation of conditional probability of remission. The probability of PTSD remission over time was graphically shown for different age groups, starting from children aged 0-12 years and up to adults aged 60 years and above.

Two studies [3, 8] were systematic reviews of naturalistic, long term outcome studies on PTSD in adults. Both reviews reported a wide range of remission rates across primary studies, between 6% and 92%. One study was a prospective cohort study of PTSD risk and resilience in 10,835 World Trade Centre responders [4]. Another study assessed the trajectories of PTSD in 214 veterans from the 1982 Lebanon War over 20 years [7]. Finally, one study was a long-term follow-up (8 years) study of female rape survivors with PTSD that had participated in a RCT that compared cognitive processing therapy with prolonged exposure [5].

After reviewing the available studies, we decided to inform the economic model with data reported by Chapman *et al.* [2], as the study sample was more likely to be similar to the UK population presenting to the National Health Service (NHS) for PTSD symptoms. Moreover, the study reported detailed remission data, supplemented with survival curves that were possible to extract and use in the economic model over the time horizon of the analysis. Digital software (<http://www.digitizeit.de>) was used to read and extract the cumulative proportions of adults that remitted from PTSD at 3 months, 12 months, 24 months, and 36 months from PTSD onset and supplement values already reported in the study. The extracted values were used to estimate the probability of remission between 0-3 months, 3-12 months, 12-24 months and 24-36 months, conditional on not having achieved remission prior to the beginning of each interval. The estimated probabilities of remission during these time periods were subsequently transformed into 3-month probabilities that were used to inform the economic model.

The table below shows the estimated cumulative probability of remission for adults at 3, 12, 24 and 36 months from PTSD onset; the probability of remission between 0-3, 3-12, 12-24 and 24-36 months (conditional on not having achieved remission prior to the beginning of the interval); and the 3-monthly probability of remission during these time periods.

**Probability of remission over time in children and young people with PTSD, as estimated based on data extracted from Chapman *et al.* [2]**

| **Time from PTSD onset** | **Cumulative probability of remission** | **Time interval** | **Probability of remission over the time interval*** | **3-month probability during the time interval*** |
| --- | --- | --- | --- | --- |
| 3 months | 0.026 | 0-3 months | 0.026 | 0.026 |
| 12 months | 0.149 | 3-12 months | 0.126 | 0.044 |
| 24 months | 0.266 | 12-24 months | 0.137 | 0.036 |
| 36 months | 0.320 | 24-36 months | 0.074 | 0.019 |
| * conditional on not having achieved remission prior to the beginning of the interval | | | | |

The economic analysis evaluated interventions for the treatment of adults with PTSD initiated more than three months after a traumatic event. The economic model was thus assumed to start at month 3 from PTSD onset. Therefore, remission data corresponding to 0-3 months after PTSD onset were not used in the economic analysis.

The estimated 3-month probability of remission over 3-12 months from PTSD onset informed months 0-9 of the economic model: these data were applied onto the no treatment arm. They also informed all model arms in months 3-6 of the economic model in the base-case analysis, and all model arms in months 6-9 in all analyses of the economic model, as the course of PTSD after 6 months of treatment was assumed to be independent of the treatment received.

The 3-month probability of remission over 12-24 months from PTSD onset informed all model arms in months 9-21 of the economic model. The 3-month probability of remission over 24-36 months from PTSD onset informed all model arms in months 21-36 of the economic model; this 3-month probability was also utilised over the period of 36-39 months from PTSD onset (i.e. months 33-36 of the economic model) for simplicity.

**References**

1. Breslau N, Kessler RC, Chilcoat HD, Schultz LR, Davis GC, Andreski P. Trauma and posttraumatic stress disorder in the community: the 1996 Detroit Area Survey of Trauma. Arch Gen Psychiatry 1998;55(7): 626-32.
2. Chapman C, Mills K, Slade T, McFarlane AC, Bryant RA, Creamer M, et al. Remission from post-traumatic stress disorder in the general population. Psychol Med 2012;42(8): 1695-703.
3. Morina N, Wicherts JM, Lobbrecht J, Priebe S. Remission from post-traumatic stress disorder in adults: a systematic review and meta-analysis of long term outcome studies. Clin Psychol Rev 2014;34(3): 249-55.
4. Pietrzak RH, Feder A, Singh R, Schechter CB, Bromet EJ, Katz CL, et al. Trajectories of PTSD risk and resilience in World Trade Center responders: an 8-year prospective cohort study. Psychol Med 2014;44(1): 205-19.
5. Resick PA, Williams LF, Suvak MK, Monson CM, Gradus JL. Long-term outcomes of cognitive-behavioral treatments for posttraumatic stress disorder among female rape survivors. Journal of Consulting and Clinical Psychology 2012;80(2) :201-10.
6. Rosellini AJ, Liu H, Petukhova MV, Sampson NA, Aguilar-Gaxiola S, Alonso J, et al. Recovery from DSM-IV post-traumatic stress disorder in the WHO World Mental Health surveys. Psychol Med 2018;48(3): 437-50.
7. Solomon Z, Mikulincer M. Trajectories of PTSD: a 20-year longitudinal study. Am J Psychiatry 2006;163(4): 659-66.
8. Steinert C, Hofmann M, Leichsenring F, Kruse J. The course of PTSD in naturalistic long-term studies: high variability of outcomes. A systematic review. Nord J Psychiatry 2015;69(7): 483-96.
