# Supplementary material for: Cost-effectiveness of psychological treatments for post-traumatic stress disorder in adults
Source: PLoS One. 2020 Apr 30;15(4):e0232245. doi: 10.1371/journal.pone.0232245 (PMC7192458; doi:10.1371/journal.pone.0232245)
Supplement: S3 File — (DOCX) [file pone.0232245.s003.docx]

# **Estimation of the unit costs of therapists delivering psychological interventions for PTSD in the British National Health Service (NHS)**

## Estimation of the unit cost of a psychological therapist [salary Band 7 according to the NHS Agenda for Change for community-based scientific and professional staff]

| **Cost element** | **Unit cost (2017 price)** | **Source** |
| --- | --- | --- |
| **Wages – salary (annual)** | £38,951 | [1]; unit cost of community-based scientific & professional staff (Agenda for Change band 7) |
| **Salary on-costs (annual)** | £9,864 |  |
| **Overheads – staff (annual)** | £11,960 |  |
| **Overheads - non-staff (annual)** | £18,647 |  |
| **Capital overheads (annual)** | £5,125 |  |
| **Qualifications (total)** | £12,386 | Based on a mean clinical psychologist training cost estimate of £159,420 [2], annuitised using a published formula [3], assuming a useful working life of 25 years, a time from obtaining the qualification until retirement of 44 years, and an equal distribution of the useful working life over the period of 44 years due to lack of specific information on this distribution. |
| **Supervision (annual)** | £316 | Based on the unit cost of an Agenda for Change band 8a therapist [1] providing 1.5 hour of supervision per month, delivered in groups of 4 participants ([4] and expert advice); qualification costs included, as described above. |
| **SUM of cost elements (annual)** | **£97,249** |  |
| Working time | 42.6 weeks /year  37.5 hours /week  (1,599 hours) | [1] |
| **Total cost per hour** | **£61** |  |
| **Ratio of direct to indirect time*** | 60:40 | Assumption based on expert opinion and a review of respective ratios reported in the literature for clinical psychologists and other therapists delivering psychological interventions [1] |
| **Estimated cost per hour of direct contact** | **£101** |  |
| * ratio of face-to-face time to time for preparation and other administrative tasks | | |

## Estimation of the unit cost of a psychological well-being practitioner [salary Band 5 according to the NHS Agenda for Change for community-based scientific and professional staff]

| **Cost element** | **Unit cost (2017 price)** | **Source** |
| --- | --- | --- |
| **Wages – salary (annual)** | £23,439 | [1]; unit cost of community-based scientific & professional staff (Agenda for Change band 5) |
| **Salary on-costs (annual)** | £5,493 |  |
| **Overheads – staff (annual)** | £7,088 |  |
| **Overheads - non-staff (annual)** | £11,052 |  |
| **Capital overheads (annual)** | £5,125 |  |
| **Qualifications (total)** | £494 | Based on a training cost estimate of £5,000 (expert advice), annuitised using a published formula [3], assuming a useful working life of 20 years, a time from obtaining the qualification until retirement of 44 years, and an equal distribution of the useful working life over the period of 44 years due to lack of specific information on this distribution. |
| **Supervision (annual)** | £1,460 | Based on the unit cost of an Agenda for Change band 7 psychological therapist as estimated in previous table, providing 2 hours of individual supervision per month. |
| **SUM of cost elements (annual)** | **£54,150** |  |
| Working time | 42.6 weeks /year  37.5 hours /week  (1,599 hours) | [1] |
| **Total cost per hour** | **£34** |  |
| **Ratio of direct to indirect time*** | 4:1 | Assumption based on expert opinion |
| **Estimated cost per hour of direct contact** | **£42** |  |
| * ratio of face-to-face time to time for preparation and other administrative tasks | | |

**References**

1. Curtis L, Burns A. Unit Costs of Health & Social Care 2017. Canterbury: PSSRU, University of Kent; 2017.
2. National College for Teaching and Leadership. Review of clinical and educational psychology training arrangements. NHS Health Education England; 2016.
3. Netten A, Knight J, Dennett J, Cooley R, Slight A. Development of a ready reckoner for staff costs in the NHS, Vols 1 & 2. Canterbury: PSSRU, University of Kent; 1998.
4. British Association for Behavioural & Cognitive Psychotherapies. Criteria and guidelines for re-accreditation as a behavioural and/or cognitive psychotherapist. British Association for Behavioural & Cognitive Psychotherapies; 2016. Available: <http://www.babcp.com/files/Accreditation/CBP/Full/CBP-Full-Guidelines-V5-0614.pdf> .
