# Supplementary material for: Cost-effectiveness of psychological treatments for post-traumatic stress disorder in adults
Source: PLoS One. 2020 Apr 30;15(4):e0232245. doi: 10.1371/journal.pone.0232245 (PMC7192458; doi:10.1371/journal.pone.0232245)
Supplement: S4 File — (DOCX) [file pone.0232245.s004.docx]

# **Estimation of annual health and personal social service costs incurred by adults with PTSD and adults without PTSD**

The costs of the PTSD and PTSD-free states were estimated using health and personal social service usage data from the Adult Psychiatric Morbidity Survey conducted in England in 2014 [1], supplemented with other published resource use data and expert opinion. The survey reported the percentage of adults with PTSD and those without PTSD that were currently receiving treatment and/or had used a range of services over the last quarter or year for a mental or emotional problem. Services included inpatient and outpatient care, and contacts with general practitioners (GPs), psychiatrists, psychologists, community psychiatric nurses, community learning disability nurses, other nursing services, social workers, self-help and support groups, home help or home care, outreach or family support workers and community day-care centres. The reported percentages of survey respondents using each service over a period of time were extrapolated, where needed, in order to estimate the percentage of adults with and without PTSD using each service annually. The exact resource use of each service (e.g. number of psychological treatment sessions, number of outpatient visits) was not available from the survey. The mean number of sessions for adults receiving psychological treatment was taken from an annual report on the use of Improving Access to Psychological Therapies (IAPT) services [2]. The mean length of stay for adults receiving inpatient care was taken from national statistics [3]. We further estimated the number and duration of visits to other services, in order to provide a total resource use estimate. Information on the number of GP visits for adults with mental health problems was sought from published UK evidence [4]. The resource use estimates were combined with national unit costs [5-7] to estimate the annual health and personal social service cost incurred by adults with PTSD and by those without PTSD. Unit costs included wages/salary, salary on costs, capital and other overheads, and qualification costs. Details on the data and assumptions used to estimate the annual costs associated with the PTSD and no PTSD health states are provided in the table below.

| Annual health and personal social service costs incurred by adults with PTSD and adults without PTSD (2017 prices) | | | | | | |
| --- | --- | --- | --- | --- | --- | --- |
| Type of service for a mental or emotional problem | % using the service^1^ | | Estimates on resource use | Unit costs | Weighted costs | |
|  | PTSD+ | PTSD- |  |  | PTSD+ | PTSD- |
| Current type of treatment^1^ | | | | | | |
| No treatment | 52.1 | 89.9 | Assumed that no treatment is received over the whole year | Not relevant | £0.0 | £0.0 |
| Psychotropic medication | 38.9 | 8.8 | Reported reasons for medication: sleep problems, anxiety, depression, ADHD, psychosis, BD [1].  Assumed that medication is received over 12 months. | Drug acquisition cost assumed to be £5/month for each type of medication, to account for some people receiving non-generic drugs or combinations of drugs; moreover, some medication requires monitoring testing (e.g. testing of glucose blood levels), which incurs extra costs. For reference, the monthly cost of citalopram 10, 20 or 40mg/day is approximately £1.5/month [7] | £23.4 | £5.3 |
| Substance use medication | 8.7 | 0.7 | Assumed that medication is received over 12 months. |  | £5.2 | £0.4 |
| Psychological treatment | 24.0 | 1.9 | Reported types of treatment: psychotherapy / psychoanalysis; CBT; art, music or drama therapy; social skills training; couple or family therapy; sex therapy; mindfulness; alcohol or drug counselling; counselling; other therapy [1].  Mean number of sessions for people with PTSD 7, based on the range of number of sessions for high-intensity therapies in IAPT services (2.8 to 8.6, with CBT 7.1 and EMDR 6.5), taking into account that “people with PTSD would be expected to receive high intensity therapies from the start of their treatment” [2]. Same mean number of sessions conservatively assumed for people without PTSD. Duration of each session 1.5 hour (expert advice). Therapy delivered by NHS AfC Band 7 psychological therapists (expert advice). | Unit cost of NHS AfC Band 7 psychological therapist £101 per hour of patient contact, as estimated in Appendix 3. | £255.4 | 20.5 |
| Other healthcare service^1^ | | | | | | |
| Inpatient stay in past quarter | 1.7 | 0.1 | Percentages conservatively multiplied x 2 to reflect more accurately annual resource use (considering that some people may have been hospitalised earlier in the year, and others may have had multiple admissions).  Mean LOS 29 days, based on the weighted mean LOS for F30-F39 (Mood [affective] disorders) and F40-F69 (Neurotic, behavioural & personality disorders); mean LOS for PTSD 31 days [3]. | Cost per bed-day £404, based on the weighted mental health care cluster per bed-day [6]. | £389.5 | £14.9 |
| Outpatient visit in past quarter | 6.2 | 0.4 | Percentages conservatively multiplied x 2 to reflect more accurately annual resource use (considering that some people may have had one or more outpatient visits earlier in the year); estimated number of outpatient visits per year 3 (expert opinion). | Unit cost per outpatient visit £141 ([6]; “Other Mental Health Specialist Teams, Adult and Elderly”) | £52.2 | £3.5 |
| Spoken with GP in past year | 60.2 | 10.3 | 9 visits per year based on expert opinion and supported by evidence that the annual number of GP visits per person are 11 for people with SMI and 5 for people without SMI [4]. According to expert advice, the number of visits for people with PTSD are more likely to approximate those for people with SMI; conservatively, this number was also used for people without PTSD. | Unit cost per GP visit £37, including direct care staff and qualification costs [5] | £200.5 | £34.2 |
| Community care - past year^1^ | | | | | | |
| Psychiatrist | 10.5 | 0.6 | 1 consultant psychiatrist visit per year lasting 1 hour (expert opinion) | Unit cost of consultant psychiatrist £361 per hour of patient contact, using national unit cost data [5] and a ratio of direct: indirect time of 1:1.58. | £38.0 | £2.3 |
| Psychologist | 6.4 | 0.6 | 1 Band 7 psychological therapist visit per year lasting 1 hour (expert opinion) | Unit cost of NHS AfC Band 7 therapist £101 per hour of patient contact, as estimated in Appendix 3. | £6.5 | £0.6 |
| Community Psychiatric Nurse | 7.8 | 0.4 | Estimated to reflect care co-ordination; 12 Band 6 nurse visits per year, lasting 45 min each (expert opinion). | Unit cost of Band 6 nurse £85 per hour of patient contact, using national unit cost data [5] and a ratio of direct: indirect time of 60:40. | £59.7 | £3.0 |
| Community Learning Disability nurse | - | 0.0 | 2 Band 5 nurse visits per year, lasting 30 min each (expert opinion) | Unit cost of Band 5 nurse £71 per hour of patient contact, using national unit cost data [5] and a ratio of direct: indirect time of 60:40. | £0.0 | £0.0 |
| Other nursing services | 2.4 | 2.5 | 2 Band 5 nurse visits per year, lasting 30 min each (expert opinion) | Unit cost of Band 5 nurse £71 per hour of patient contact, using national unit cost data [5] and a ratio of direct: indirect time of 60:40. | £1.7 | £1.7 |
| Social worker | 5.3 | 0.8 | Estimated to reflect care co-ordination; 12 social worker visits per year, lasting 45 min each (expert opinion). | Unit cost of social worker for adult services £82 per hour of client-related work [5] | £38.8 | £6.0 |
| Self-help/support group | 4.5 | 0.8 | 10 sessions of 2 hours each delivery by a Band 5 PWP, 10 participants per group (expert opinion) | Unit cost of Band 5 community-based scientific & professional staff [5], assuming a ratio of direct: indirect time of 1:0.25 and a £5,000 qualification cost (expert advice), annuitised using a published formula [8], assuming a useful working life of 20 years, a period from obtaining the qualification until retirement of 44 years, and even spread of useful working life over the period of 44 years | £3.8 | £0.7 |
| Home help/home care | 1.6 | 0.7 | Estimated to reflect care co-ordination; 12 Band 5 nurse visits per year, lasting 45 min each (expert opinion). | Unit cost of Band 5 nurse £71 per hour of patient contact, using national unit cost data [5] and a ratio of direct: indirect time of 60:40. | £10.2 | £4.5 |
| Outreach worker/family support | 6.6 | 0.7 | Estimated to consist of a few visits occurring before outpatient visits or a few visits for support; 5 family support worker visits per year, lasting 1 hour each (expert opinion). | Unit cost of Band 5 nurse £54 per hour of patient contact, using national unit cost data [5], a ratio of direct: indirect time of 60:40, and a £5,000 qualification cost (assumption), annuitised using a published formula [8], assuming a useful working life of 20 years, a period from obtaining the qualification until retirement of 44 years, and even spread of useful working life over the period of 44 years | £17.7 | £1.8 |
| Community day-care centre^3^ | 9.7 | 1.4 | 8 weeks (2 months) of care per year (expert opinion), 3 sessions per week 5] | Cost per session £30 [5] | £70.1 | £10.1 |
| TOTAL ANNUAL COST | | | | | £1,173 | £110 |
| 1 Data from Adult Psychiatry Morbidity Survey, England 2014 [1]  2 Some people receive more than one types of therapy and/or services, hence sums of percentages of people receiving individual therapies and/or services may exceed 100%  3 Includes community mental health centre, sheltered workshop, day activity centre and other day services.  ADHD: attention-deficit hyperactivity disorder; AfC: agenda for change; BD: bipolar disorder; CBT: cognitive behavioural therapy; EMDR: Eye movement desensitisation and reprocessing; GP: general practitioner; IAPT: improving access to psychological therapies; LOS: length of stay; NHS: national health service; PWP: psychological wellbeing practitioner; SMI: severe mental illness | | | | | | |

**References**

1. McManus S, Bebbington P, Jenkins R, Brugha T (eds). Mental health and wellbeing in England: Adult Psychiatric Morbidity Survey 2014. Leeds: NHS Digital; 2016.
2. NHS Digital, Community and Mental Health Team. Psychological Therapies: Annual report on the use of IAPT services. England, 2015-16. NHS Digital; 2016.
3. NHS Digital. Hospital Episode Statistics for England. Admitted Patient Care statistics, 2016-17. NHS Digital; 2017.
4. Kontopantelis E, Olier I, Planner C, Reeves D, Ashcroft DM, Gask L, et al. Primary care consultation rates among people with and without severe mental illness: a UK cohort study using the Clinical Practice Research Datalink. BMJ Open 2015;5(12): e008650.
5. Curtis L, Burns A. Unit Costs of Health & Social Care 2017. Canterbury: PSSRU, University of Kent; 2017.
6. NHS Improvement. National Schedule of Reference Costs, 2016-17. NHS trusts and NHS foundation trusts. NHS Improvement; 2017. Available: <https://improvement.nhs.uk/resources/reference-costs/>
7. NHS Business Services Authority, NHS Prescription Services. NHS England and Wales. Electronic Drug Tariff. Issue: March 2018. Compiled on the behalf of the Department of Health; 2018. Available: <https://www.nhsbsa.nhs.uk/pharmacies-gp-practices-and-appliance-contractors/drug-tariff>
8. Netten A, Knight J, Dennett J, Cooley R, Slight A. Development of a ready reckoner for staff costs in the NHS, Vols 1 & 2. Canterbury: PSSRU, University of Kent; 1998.
