# Supplementary material for: Cost-effectiveness of psychological treatments for post-traumatic stress disorder in adults
Source: PLoS One. 2020 Apr 30;15(4):e0232245. doi: 10.1371/journal.pone.0232245 (PMC7192458; doi:10.1371/journal.pone.0232245)

# **Results of secondary probabilistic economic analysis [beneficial effect up to 3-months post-treatment]**

| **Intervention** | **Mean per person** | | | **NMB (£/ person)** | **Mean rank** | **Prob*** |
| --- | --- | --- | --- | --- | --- | --- |
|  | **QALY** | **Intervention cost (£)** | **Total cost (£)** |  | **(at a threshold of £20,000/QALY)** | |
| EMDR | 1.80 | 746 | 2,042 | 33,906 | 3.20 | 0.14 |
| Combined somatic/cognitive therapies | 1.78 | 360 | 1,781 | 33,884 | 3.20 | 0.31 |
| SH with support | 1.77 | 265 | 1,799 | 33,614 | 3.51 | 0.27 |
| SH without support | 1.75 | 98 | 1,780 | 33,286 | 4.19 | 0.35 |
| SSRI | 1.75 | 146 | 1,848 | 33,198 | 4.33 | 0.47 |
| Psychoeducation | 1.74 | 108 | 1,904 | 32,979 | 4.91 | 0.60 |
| TF-CBT | 1.74 | 1,061 | 2,890 | 31,932 | 7.39 | 0.32 |
| non-TF-CBT | 1.73 | 709 | 2,673 | 31,863 | 7.47 | 0.51 |
| TF-CBT + SSRI | 1.73 | 1,208 | 3,177 | 31,344 | 8.80 | 0.43 |
| Counselling | 1.70 | 785 | 3,018 | 30,938 | 9.66 | 0.40 |
| No treatment | 1.67 | 0 | 2,494 | 30,917 | 9.35 | 1.00 |
| EMDR: eye movement desensitisation reprocessing; NMB: net monetary benefit; Prob: probability of cost-effectiveness; SSRIs: selective serotonin reuptake inhibitors; TF-CBT: trauma-focused cognitive behavioural therapy  *estimated in a step-wise approach, according to which the most cost-effective intervention is omitted at each step, and the probability of cost-effectiveness of the next most cost-effective intervention amongst the remaining treatment options is re-calculated | | | | | | |

Cost-effectiveness plane


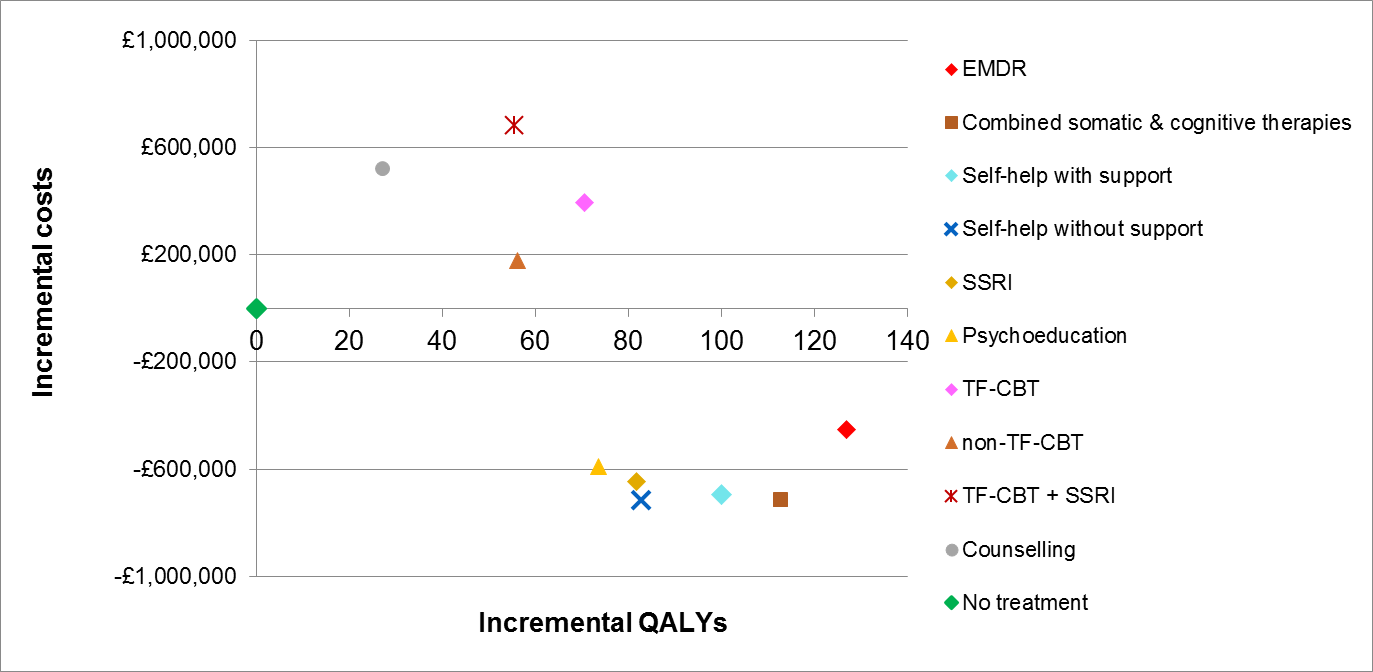


Cost-effectiveness acceptability frontier


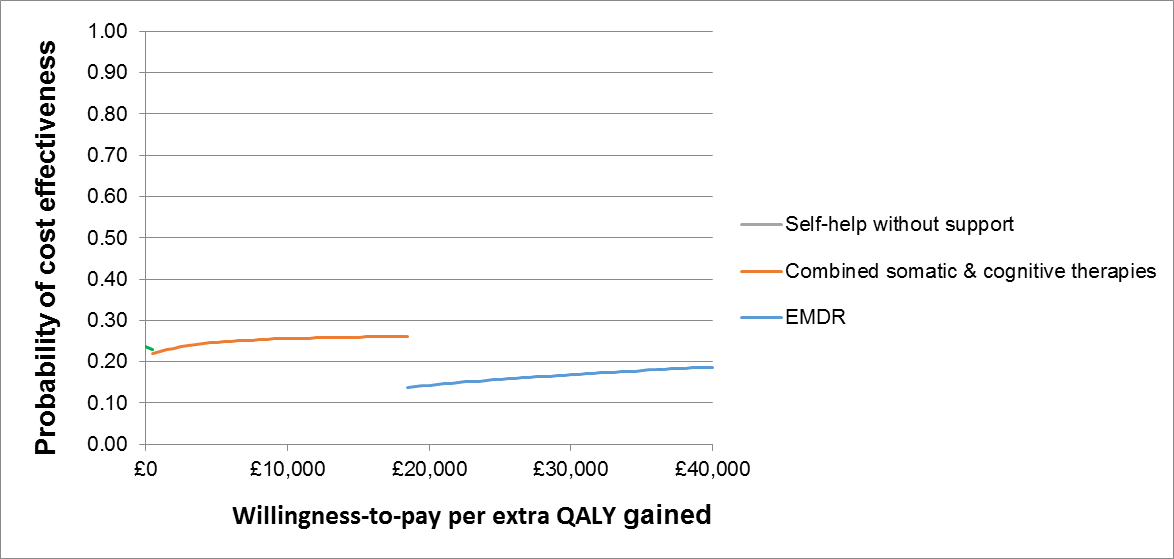

Supplement: S5 File — (DOCX) [file pone.0232245.s005.docx]
