# Supplementary material for: Cost-effectiveness of psychological treatments for post-traumatic stress disorder in adults
Source: PLoS One. 2020 Apr 30;15(4):e0232245. doi: 10.1371/journal.pone.0232245 (PMC7192458; doi:10.1371/journal.pone.0232245)
Supplement: S6 File — (DOCX) [file pone.0232245.s006.docx]

# **Results of deterministic sensitivity analyses**

## Change in the annual risk of relapse

| **Base-case annual risk of relapse (0.10)** | | **Annual risk of relapse 0.05** | | **Annual risk of relapse 0.20** | |
| --- | --- | --- | --- | --- | --- |
| **Intervention** | **NMB (£/person)** | **Intervention** | **NMB (£/person)** | **Intervention** | **NMB (£/person)** |
| EMDR | 32,848 | EMDR | 33,126 | EMDR | 32,322 |
| Combined somatic & cognitive therapies | 32,515 | Combined somatic & cognitive therapies | 32,737 | Combined somatic & cognitive therapies | 32,094 |
| Self-help with support | 32,126 | Self-help with support | 32,313 | Self-help with support | 31,771 |
| Psychoeducation | 31,761 | Psychoeducation | 31,914 | Psychoeducation | 31,469 |
| SSRI | 31,588 | SSRI | 31,733 | SSRI | 31,312 |
| Self-help without support | 31,278 | Self-help without support | 31,397 | Self-help without support | 31,050 |
| TF-CBT | 31,030 | TF-CBT | 31,217 | non-TF-CBT | 30,693 |
| non-TF-CBT | 30,987 | non-TF-CBT | 31,141 | TF-CBT | 30,674 |
| No treatment | 30,620 | No treatment | 30,688 | No treatment | 30,486 |
| TF-CBT + SSRI | 30,348 | TF-CBT + SSRI | 30,502 | TF-CBT + SSRI | 30,055 |
| Counselling | 30,123 | Counselling | 30,226 | Counselling | 29,925 |
| EMDR: eye movement desensitisation reprocessing; NMB: net monetary benefit; SSRIs: selective serotonin reuptake inhibitor; TF-CBT: trauma-focused cognitive behavioural therapy | | | | | |

## Change in the PTSD health state cost

| **Base-case PTSD health state cost** | | **50% increase in PTSD health state cost** | | **50% reduction in PTSD health state cost** | |
| --- | --- | --- | --- | --- | --- |
| **Intervention** | **NMB (£/person)** | **Intervention** | **NMB (£/person)** | **Intervention** | **NMB (£/person)** |
| EMDR | 32,848 | EMDR | 32,306 | EMDR | 33,390 |
| Combined somatic & cognitive therapies | 32,515 | Combined somatic & cognitive therapies | 31,796 | Combined somatic & cognitive therapies | 33,234 |
| Self-help with support | 32,126 | Self-help with support | 31,296 | Self-help with support | 32,956 |
| Psychoeducation | 31,761 | Psychoeducation | 30,824 | Psychoeducation | 32,697 |
| SSRI | 31,588 | SSRI | 30,626 | SSRI | 32,550 |
| Self-help without support | 31,278 | Self-help without support | 30,233 | Self-help without support | 32,322 |
| TF-CBT | 31,030 | TF-CBT | 30,201 | non-TF-CBT | 31,921 |
| non-TF-CBT | 30,987 | non-TF-CBT | 30,053 | TF-CBT | 31,859 |
| No treatment | 30,620 | No treatment | 29,416 | No treatment | 31,823 |
| TF-CBT + SSRI | 30,348 | TF-CBT + SSRI | 29,413 | TF-CBT + SSRI | 31,283 |
| Counselling | 30,123 | Counselling | 29,028 | Counselling | 31,218 |
| EMDR: eye movement desensitisation reprocessing; NMB: net monetary benefit; SSRIs: selective serotonin reuptake inhibitor; TF-CBT: trauma-focused cognitive behavioural therapy | | | | | |

## Use of alternative utility data [1]

| **Base-case utility data** | | **Alternative utility data** | |
| --- | --- | --- | --- |
| **Intervention** | **NMB (£/person)** | **Intervention** | **NMB (£/person)** |
| EMDR | 32,848 | EMDR | 34,212 |
| Combined somatic & cognitive therapies | 32,515 | Combined somatic & cognitive therapies | 34,200 |
| Self-help with support | 32,126 | Self-help with support | 34,013 |
| Psychoeducation | 31,761 | Psychoeducation | 33,840 |
| SSRI | 31,588 | SSRI | 33,713 |
| Self-help without support | 31,278 | Self-help without support | 33,552 |
| TF-CBT | 31,030 | No treatment | 33,182 |
| non-TF-CBT | 30,987 | non-TF-CBT | 33,061 |
| No treatment | 30,620 | TF-CBT | 32,914 |
| TF-CBT + SSRI | 30,348 | Counselling | 32,488 |
| Counselling | 30,123 | TF-CBT + SSRI | 32,423 |
| EMDR: eye movement desensitisation reprocessing; NMB: net monetary benefit; SSRI: selective serotonin reuptake inhibitor; TF-CBT: trauma-focused cognitive behavioural therapy | | | |

[1] Freed MC, Yeager DE, Liu X, Gore KL, Engel CC, Magruder KM. Preference-weighted health status of PTSD among veterans: an outcome for cost-effectiveness analysis using clinical data. Psychiatr Serv. 2009;60(9):1230-8.
