# Supplementary material for: Cost-effectiveness of psychological treatments for post-traumatic stress disorder in adults
Source: PLoS One. 2020 Apr 30;15(4):e0232245. doi: 10.1371/journal.pone.0232245 (PMC7192458; doi:10.1371/journal.pone.0232245)
Supplement: S7 File — (DOCX) [file pone.0232245.s007.docx]

# **Results of the NICE guideline economic analysis**

## Base-case analysis [no beneficial effect beyond treatment endpoint]

| Intervention | Mean per person | | | NMB £/ person | Mean rank |
| --- | --- | --- | --- | --- | --- |
|  | QALY | Inter cost £ | Total cost £ |  |  |
| TF-CBT individual <8 sessions | 1.81 | 541 | 1,722 | 34,467 | 2.49 |
| Psychoeducation | 1.79 | 109 | 1,506 | 34,214 | 3.54 |
| EMDR | 1.79 | 747 | 2,103 | 33,709 | 3.71 |
| Combined somatic & cognitive therapies | 1.76 | 358 | 1,964 | 33,314 | 4.56 |
| SH with support | 1.75 | 266 | 2,036 | 32,876 | 5.44 |
| SSRI | 1.71 | 145 | 2,209 | 32,065 | 7.65 |
| SH without support | 1.71 | 98 | 2,241 | 31,873 | 8.18 |
| TF-CBT individual 8-12 sessions | 1.74 | 1,181 | 2,983 | 31,865 | 9.21 |
| IPT | 1.73 | 810 | 2,747 | 31,805 | 9.66 |
| non-TF-CBT | 1.72 | 706 | 2,676 | 31,800 | 9.13 |
| Present-centred therapy | 1.74 | 1,373 | 3,228 | 31,498 | 10.79 |
| TF-CBT group 8-12 sessions | 1.70 | 362 | 2,592 | 31,334 | 10.91 |
| TF-CBT individual 8-12 sessions + SSRI | 1.72 | 1,326 | 3,348 | 31,022 | 12.49 |
| No treatment | 1.67 | 0 | 2,471 | 30,935 | 12.10 |
| TF-CBT individual >12 sessions | 1.71 | 1,205 | 3,325 | 30,841 | 13.05 |
| Counselling | 1.69 | 785 | 3,038 | 30,838 | 13.09 |
| EMDR: eye movement desensitisation reprocessing; Inter: intervention; NMB: net monetary benefit; Prob: probability; SH: self-help; SSRI: selective serotonin reuptake inhibitor; TF-CBT: trauma-focused cognitive behavioural therapy | | | | | |

## Secondary analysis [beneficial effect up to 3-months post-treatment]

| Intervention | Mean per person | | | NMB £/ person | Mean rank |
| --- | --- | --- | --- | --- | --- |
|  | QALY | Inter cost £ | Total cost £ |  |  |
| TF-CBT individual <8 sessions | 1.808 | 540 | 1,742 | 34,420 | 3.30 |
| Psychoeducation | 1.788 | 108 | 1,500 | 34,250 | 4.01 |
| Combined somatic & cognitive therapies | 1.785 | 360 | 1,763 | 33,939 | 4.23 |
| EMDR | 1.794 | 747 | 2,082 | 33,789 | 4.59 |
| SH with support | 1.769 | 266 | 1,818 | 33,562 | 4.87 |
| SH without support | 1.753 | 98 | 1,783 | 33,270 | 5.69 |
| SSRI | 1.741 | 145 | 1,950 | 32,871 | 6.62 |
| IPT | 1.737 | 811 | 2,670 | 32,078 | 9.67 |
| TF-CBT individual 8-12 sessions | 1.746 | 1,178 | 2,954 | 31,972 | 9.93 |
| non-TF-CBT | 1.727 | 706 | 2,655 | 31,892 | 9.93 |
| TF-CBT individual >12 sessions | 1.732 | 1,204 | 3,097 | 31,549 | 11.39 |
| Present-centred therapy | 1.738 | 1,377 | 3,226 | 31,539 | 11.46 |
| TF-CBT group 8-12 sessions | 1.697 | 362 | 2,599 | 31,338 | 11.65 |
| TF-CBT individual 8-12 sessions + SSRI | 1.719 | 1,323 | 3,347 | 31,042 | 13.01 |
| Counselling | 1.700 | 782 | 2,991 | 31,003 | 13.07 |
| No treatment | 1.671 | 0 | 2,477 | 30,944 | 12.60 |
| EMDR: eye movement desensitisation reprocessing; Inter: intervention; NMB: net monetary benefit; Prob: probability; SH: self-help; SSRI: selective serotonin reuptake inhibitor; TF-CBT: trauma-focused cognitive behavioural therapy | | | | | |
