# Supplementary material for: Cost-effectiveness of psychological treatments for post-traumatic stress disorder in adults
Source: PLoS One. 2020 Apr 30;15(4):e0232245. doi: 10.1371/journal.pone.0232245 (PMC7192458; doi:10.1371/journal.pone.0232245)
Supplement: S1 Appendix — (DOCX) [file pone.0232245.s008.docx]

# **Appendix 1: Search strategy**

**Database:** Epub Ahead of Print, In-Process & Other Non-Indexed Citations, Ovid MEDLINE(R) Daily and Ovid MEDLINE(R), Embase, PsycINFO

**Date of last search:** 29 January 2018

| # | Searches |
| --- | --- |
| 1 | *acute stress/ or *behavioural stress/ or *emotional stress/ or *critical incident stress/ or *mental stress/ or *posttraumatic stress disorder/ or *psychotrauma/ |
| 2 | 1 use emez |
| 3 | stress disorders, traumatic/ or combat disorders/ or psychological trauma/ or stress disorders, post-traumatic/ or stress disorders, traumatic, acute/ or stress, psychological/ |
| 4 | 3 use mesz, prem |
| 5 | exp posttraumatic stress disorder/ or acute stress disorder/ or combat experience/ or emotional trauma/ or post-traumatic stress/ or traumatic neurosis/ or trauma/ or psychological stress/ or chronic stress/ |
| 6 | 5 use psyh |
| 7 | (railway spine or (rape adj2 trauma*) or reexperienc* or re experienc* or torture syndrome or traumatic neuros* or traumatic stress).ti,ab. |
| 8 | (trauma* and (avoidance or grief or horror or death* or nightmare* or night mare* or emotion*)).ti,ab. |
| 9 | (posttraumatic* or post traumatic* or stress disorder* or acute stress or ptsd or asd or desnos or (combat neuros* or combat syndrome or concentration camp syndrome or extreme stress or flashback* or flash back* or hypervigilan* or hypervigilen* or psych* stress or psych* trauma* or psycho?trauma* or psychotrauma*) or (posttrauma* or traumagenic* or traumatic stress*)).ti,ab. |
| 10 | or/2,4,6-9 |
| 11 | *psychotherapy/ use emez or psychotherapy/ use mesz, prem,psyh |
| 12 | (((psycholog* or psycho social* or psychosocial*) adj3 (intervention* or program* or therap* or treat*)) or psychotherap* or psycho therap* or talk* therap* or therapeutic technique* or therapist* or third wave or time limited).ti,ab,sh. |
| 13 | exp *behavior therapy/ or exp *cognitive therapy/ |
| 14 | 13 use emez |
| 15 | exp behavior therapy/ use mesz, prem |
| 16 | exp behavior therapy/ or exp cognitive behavior therapy/ |
| 17 | 16 use psyh |
| 18 | (((behaviour* or behavior*) adj2 cognitiv*) or cbt or ccbt or ((behav* or cognitive*) adj3 (intervention* or manag* or program* or restructure* or therap* or treat*)) or (stress inoculation adj2 (intervention* or program* or therap* or train* or treat*)) or (behav* adj2 activat*) or ((trauma adj (based or focused or led)) or exposure based or prolonged exposure)).ti,ab. |
| 19 | *emotion/ use emez or emotions/ use mesz, prem |
| 20 | emotion focused therapy/ or sympathy/ |
| 21 | 20 use psyh |
| 22 | (((compassion or emotion* or emotive*) adj (based or focused or led)) or emotional processing or ((compassion or emotion* or emotive*) adj3 (coach* or intervention* or program* or therap* or treat*))).ti,ab. |
| 23 | exposure therapy/ or narrative therapy/ or virtual reality exposure therapy/ |
| 24 | 23 use emez |
| 25 | implosive therapy/ or narrative therapy/ or virtual reality exposure therapy/ |
| 26 | 25 use mesz, prem |
| 27 | exposure therapy/ or narrative therapy/ or virtual reality/ |
| 28 | 27 use psyh |
| 29 | (((augmented or virtual) adj2 reality) or (virtual adj (environment or restorative)) or ((exposure or implosive or virtual reality) adj2 (intervention* or program* or therap* or train*))).ti,ab. |
| 30 | ((imagery adj2 (rehears* or re hears*)) or (((lower* or reduc*) adj3 (bad dream* or nightmare*)) and (intervention* or program* or therap* or treat*)) or ((intervention* or program* or therap* or treat*) adj3 nightmare*)).mp. or ((presleep or presleep) adj2 imagery).ti,ab. |
| 31 | (mindfulness or ((exposure or narrative) adj therapy)).sh. |
| 32 | (kidnet or mindful* or narrative therap*).ti,ab. |
| 33 | exp "debriefing (psychological)"/ use psyh |
| 34 | debrief*.ti,ab. |
| 35 | eye movement desensitization reprocessing/ use mesz, prem or eye movement desensitization therapy/ use psyh or (emdr or (eye movement adj2 desensiti*)).ti,ab. |
| 36 | hypnosis/ use emez or exp hypnosis/ use mesz, prem or exp hypnotherapy/ use psyh or (hypnosis or hypnotherap*).ti,ab. |
| 37 | psychodynamic psychotherapy/ use emez or psychotherapy, psychodynamic/ use mesz, prem or psychodynamic psychotherapy/ use psyh or repetitive transcranial magnetic stimulation/ use emez or Transcranial Magnetic Stimulation/ use mesz, prem, psyh |
| 38 | ((psychodynamic or (dynamic adj (psychotherapy* or therap*)) or incident reduction) or ((brain or transcranial) adj2 stimulat*) or rtms).ti,ab. |
| 39 | (psychoanal* or psychosomatic*).ti,ab. |
| 40 | exp counseling/ use emez,mesz,psyh or counsel*.ti,ab. |
| 41 | (hg therap* or human givens).ti,ab. |
| 42 | psychosomatic disorder/th use emez or exp somatoform disorders/th use mesz, prem |
| 43 | (exp somatoform disorders/ or somatization/) and (intervention* or program* or therap* or treat*).ti,ab,hw. use psyh |
| 44 | (psychosomatic* or somatherap* or somatic*).ti,ab. |
| 45 | (emotional freedom or holistic or thought field).ti,ab. |
| 46 | dance therap*.ti,ab,sh. |
| 47 | couple therapy/ or family therapy/ or marital therapy/ or exp parent/ed |
| 48 | 47 use emez |
| 49 | couples therapy/ or family therapy/ or marital therapy/ or exp parents/ed |
| 50 | 49 use mesz, prem |
| 51 | couples therapy/ or family intervention/ or exp family therapy/ or exp marriage counseling/ or parent training/ |
| 52 | 51 use psyh |
| 53 | (((con?joint or couple* or family or families or husband* or marriage* or marital* or partner* or relations* or spous* or wife or wives* or (child* adj5 parent*)) adj6 (counsel* or intervention* or program* or support* or therap* or treat*)) or ((couples* or family* or relations*) adj (based or focused or led)) or ecological therap* or expressed emotion or family dynamics or family relationships).tw. |
| 54 | ((child* adj2 family traumatic stress intervention) or cftsi).ti,ab. |
| 55 | play therapy.sh. |
| 56 | (doll therap* or ((play or playful) adj3 (intervention* or program* or therap* or treat*)) or sandplay or sand play).ti,ab. |
| 57 | meditation.sh. or meditat*.ti,ab. |
| 58 | mindfulness.sh. or (mbsr or mindful*).ti,ab. |
| 59 | exp horticulture/ or occupational therapy/ or recreational therapy/ |
| 60 | 59 use emez |
| 61 | horticultural therapy/ or occupational therapy/ or recreation therapy/ |
| 62 | 61 use mesz, prem |
| 63 | exp "nature (environment)"/ or horticulture therapy/ or recreation therapy/ or occupational therapy/ |
| 64 | 63 use psyh |
| 65 | ((nature adj (assisted or based)) or (nature adj3 (intervention* or program* or therap* or  treat*)) or ecotherap* or e cotherap* or gardening or horticult* or leisure activit* or naturopath* or occupational therap*).ti,ab. or exp animal assisted therapy/ use emez, mesz or animal assisted therapy/ use psyh or (((animal* or dog* or equine* or horse* or pet or pets) adj2(assist* or based or facilitat*)) or ((animal* or dog* or equine* or horse* or pet or pets) adj3(intervention* or therap* or treat* or program*))).ti,ab. |
| 66 | psychoeducation.sh. or (psychoed* or psycho ed*).ti,ab. |
| 67 | exp acupuncture/ use emez or exp alternative medicine/ use emez or biofeedback/ or massage/ use emez or meditation/ use emez or acupressure/ use mesz, prem or massage/ use mesz, prem or acupuncture/ use mesz, prem or exp complementary therapies/ use mesz, prem or exp alternative medicine/ use psyh or biofeedback/ use psyh or massage/ use psyh or mind body therapy/ use psyh |
| 68 | (chinese medicine or medicine, chinese traditional or (moxibustion or electroacupuncture)).sh,id. or ((alternative or complementary) adj2 (medicine* or therap*)).ti,ab,sh. or (acu point* or acupoint* or acupressur* or acupunctur* or (ching adj2 lo) or cizhen or dianzhen or electroacupunctur* or (jing adj2 luo) or jingluo or massag* or needle therap* or tapping or zhenjiu or zhenci).tw. |
| 69 | exp *exercise/ use emez or exp *kinesiotherapy/ use emez or exp exercise/ use mesz, prem or exercise therapy/ use mesz, prem or exp exercise/ use psyh (physiotherap* or physio therap* or rehab*).ti,ab,hw. |
| 70 | (((balance or flexibility or resistance or sitting* or strenth*) adj2 (exercise* or train*)) or aerobic* or anaerobic* or bowls or dancing or dance or cycling or cycle* or elliptical train* or jogging or low impact activit* or running or swimming or sprinting or swim*1 or walking or yoga or tai chi or weight train* or (weight and brain* and (change* or increas* or volum*))).ti,ab. |
| 71 | friendship/ or peer counseling/ or peer group/ or self help/ or self care/ or social network/ or social support/ or support group/ |
| 72 | 71 use emez |
| 73 | community networks/ or friends/ or exp peer group/ or self care/ or self-help groups/ or social networking/ or social support/ |
| 74 | 73 use mesz, prem |
| 75 | friendship/ or network therapy/ or exp social networks/ or peer relations/ or peers/ or peer counseling/ or self care skills/ or exp self help techniques/ or social support/ or exp support groups/ |
| 76 | 75 use psyh |
| 77 | ((self adj (administer* or assess* or attribut* or care or change or directed or efficacy or help* or guide* or instruct* or manag* or medicat* or monitor* or regulat* or reinforc* or re inforc* or support* or technique* or therap* or train* or treat*)) or selfadminister* or selfassess* or selfattribut* or selfcare or selfchange or selfdirected or selfefficacy or selfhelp* or selfguide* or selfinstruct* or selfmanag* or selfmedicat* or selfmonitor* or selfregulat* or selfreinforc* or self re inforc* or selfsupport* or selftechnique* or selftherap* or selftrain* or selftreat* or (wellness adj (therap* or train* or treat*))).ti,ab,sh. |
| 78 | (befriend* or be*1 friend* or buddy or buddies or ((community or lay or paid or support) adj (person or worker*))).ti,ab. |
| 79 | (((consumer* or famil* or friend* or lay or mutual* or peer* or social* or spous* or voluntary or volunteer*) adj3 (assist* or advice* or advis* or counsel* or educat* or forum* or help* or mentor* or network* or support* or visit*)) or ((consumer* or famil* or peer* or self help or social* or support* or voluntary or volunteer*) adj2 group*) or ((consumer* or famil* or friend* or lay or mutual* or peer* or self help or social* or spous* or support* or voluntary or volunteer*) adj3 (intervention* or program* or rehab* or therap* or service* or skill* or treat*)) or (((consumer* or famil* or friend* or lay* or peer* or spous* or user* or support* or voluntary or volunteer*) adj (based or counsel* or deliver* or interact* or led or mediat* or operated or provides or provider* or run*)) or ((consumer* or famil* or friend* or lay* or peer* or relation* or spous* or support*) adj3 trust*) or voluntary work*)).ti,ab. |
| 80 | (((lay or peer*) adj3 (advis* or consultant or educator* or expert* or facilitator* or instructor* or leader* or mentor* or person* or tutor* or worker*)) or expert patient* or mutual aid).ti,ab. |
| 81 | (peer* adj3 (assist* or counsel* or educat* or program* or rehab* or service* or supervis*)).ti,ab. |
| 82 | ((psychoeducat* or psycho educat*) adj3 (group or network* or service*)).ti,ab. |
| 83 | ((psychosocial or social) adj work*).ti,ab. |
| 84 | ((ptsd or posttrauma* or post trauma* or trauma*) adj2 support*).ti,ab. |
| 85 | recovery support.ti,ab. |
| 86 | financial management/ use emez or financial support/ use mesz, prem or finance/ use psyh |
| 87 | ((financ* or money) adj2 (assist* or educat* or guidance or intervention* or program* or support* or train*)).ti,ab. |
| 88 | assisted living facility/ or emergency shelter/ or halfway house/ or housing/ or independent living/ or residential home/ or residential home/ |
| 89 | 88 use emez |
| 90 | assisted living facilities/ or emergency shelter/ or group homes/ or halfway houses/ or housing/ or independent living/ or residential facilities/ |
| 91 | 90 use mesz, prem |
| 92 | assisted living / use psyh or shelters/ use psyh or group homes/ use psyh or halfway houses/ use psyh or housing/ use psyh or residential care institutions/ use psyh or ((resident* or hous* or accommod* or commun* or comu* or home*) adj5 (support* or support* or shelter* or outreach* or visit* or appointment*)).ti,ab. |
| 93 | (residential treatm* or residential facility* or supported hous* or public hous*).ti,ab. |
| 94 | (accomod* or assertive community treatment* or home* or housing* or outreach* or residential*).ti,ab. |
| 95 | absenteeism/ or daily life activity/ or employment/ or medical leave/ or mentoring/ or occupational health/ or occupational therapy/ or return to work/ or supported employment/ or unemployment/ or vocational guidance/ or vocational rehabilitation/ or work capacity/ or work/ |
| 96 | 95 use emez |
| 97 | absenteeism/ or "activities of daily living"/ or employment, supported/ or employment/ or mentoring/ or occupational health/ or occupational therapy/ or rehabilitation, vocational/ or return to work/ or sick leave/ or unemployment/ or vocational guidance/ or work/ |
| 98 | 97 use mesz, prem |
| 99 | "activities of daily living"/ or exp coaching/ or employee absenteeism/ or employment status/ or occupational guidance/ or occupational health/ or occupational therapy/ or reemployment/ or unemployment/ or vocational counselors/ or exp vocational rehabilitation/ |
| 100 | 99 use psyh |
| 101 | (((supp* or transitional*) adj5 (employ* or work*)) or individual placement or (placement* adj3 (employ* or work*))).ti,ab. |
| 102 | ((employ* or placement* or psychosocial* or psycho-social* or occupation* or soc* or vocation* or work* or job* or counsel*) adj5 rehab*).ti,ab. |
| 103 | (sheltered work* or vocatio* or fountain house* or fountainhouse* or clubhouse* or club house* or work therap*).ti,ab. |
| 104 | (transitional employment or rehabilitation counsel* or (occupational adj (health or medicine)) or work* adjustment).ti,ab. |
| 105 | ((performance adj (activit* or coach* or management or occupation*)) or coaching).ti,ab. |
| 106 | (((sheltered or permitted or voluntary or vocational or return* or rehabilitat*) adj3 work*) or work capacity or reemploy* or re employ* or job retention or work capacity).ti,ab. |
| 107 | ((employ* or job or occupation* or vocation* or work*) adj5 (counsel* or educat* or guidance* or intervention* or program* or rehab* or reintegrat* or re integrat* or support* or therap* or train*)).ti,ab. |
| 108 | placement.ti,ab. |
| 109 | or/11-12,14-15,17-19,21-22,24,26,28-46,48,50,52-58,60,62,64-70,72,74,76-87,89,91-94,96,98,100-108 |
| 110 | meta analysis/ or "meta analysis (topic)"/ or systematic review/ |
| 111 | 110 use emez |
| 112 | meta analysis.sh,pt. or "meta-analysis as topic"/ or "review literature as topic"/ |
| 113 | 112 use mesz, prem |
| 114 | (literature review or meta analysis).sh,id,md. or systematic review.id,md. |
| 115 | 114 use psyh |
| 116 | (exp bibliographic database/ or (((electronic or computer* or online) adj database*) or bids or cochrane or embase or index medicus or isi citation or medline or psyclit or psychlit or scisearch or science citation or (web adj2 science)).ti,ab.) and (review*.ti,ab,sh,pt. or systematic*.ti,ab.) |
| 117 | 116 use emez |
| 118 | (exp databases, bibliographic/ or (((electronic or computer* or online) adj database*) or bids or cochrane or embase or index medicus or isi citation or medline or psyclit or psychlit or scisearch or science citation or (web adj2 science)).ti,ab.) and (review*.ti,ab,sh,pt. or systematic*.ti,ab.) |
| 119 | 118 use mesz, prem |
| 120 | (computer searching.sh,id. or (((electronic or computer* or online) adj database*) or bids or cochrane or embase or index medicus or isi citation or medline or psyclit or psychlit or scisearch or science citation or (web adj2 science)).ti,ab.) and (review*.ti,ab,pt. or systematic*.ti,ab.) |
| 121 | 120 use psyh |
| 122 | ((analy* or assessment* or evidence* or methodol* or quantativ* or systematic*) adj2 (overview* or review*)).tw. or ((analy* or assessment* or evidence* or methodol* or quantativ* or systematic*).ti. and review*.ti,pt.) or (systematic* adj2 search*).ti,ab. |
| 123 | (metaanal* or meta anal*).ti,ab. |
| 124 | (research adj (review* or integration)).ti,ab. |
| 125 | reference list*.ab. |
| 126 | bibliograph*.ab. |
| 127 | published studies.ab. |
| 128 | relevant journals.ab. |
| 129 | selection criteria.ab. |
| 130 | (data adj (extraction or synthesis)).ab. |
| 131 | (handsearch* or ((hand or manual) adj search*)).ti,ab. |
| 132 | (mantel haenszel or peto or dersimonian or der simonian).ti,ab. |
| 133 | (fixed effect* or random effect*).ti,ab. |
| 134 | ((pool* or combined or combining) adj2 (data or trials or studies or results)).ti,ab. |
| 135 | or/111,113,115,117,119,121-134 |
| 136 | exp "clinical trial (topic)"/ or exp clinical trial/ or crossover procedure/ or double blind procedure/ or placebo/ or randomization/ or random sample/ or single blind procedure/ |
| 137 | 136 use emez |
| 138 | exp clinical trial/ or exp "clinical trials as topic"/ or cross-over studies/ or double-blind method/ or placebos/ or random allocation/ or single-blind method/ |
| 139 | 138 use mesz, prem |
| 140 | (clinical trials or placebo or random sampling).sh,id. |
| 141 | 140 use psyh |
| 142 | (clinical adj2 trial*).ti,ab. |
| 143 | (crossover or cross over).ti,ab. |
| 144 | (((single* or doubl* or trebl* or tripl*) adj2 blind*) or mask* or dummy or doubleblind* or singleblind* or trebleblind* or tripleblind*).ti,ab. |
| 145 | (placebo* or random*).ti,ab. |
| 146 | treatment outcome*.md. use psyh |
| 147 | animals/ not human*.mp. use emez |
| 148 | animal*/ not human*/ use mesz, prem |
| 149 | (animal not human).po. use psyh |
| 150 | or/137,139,141-146 |
| 151 | 150 not (or/147-149) |
| 152 | or/135,151 |
| 153 | 10 and 109 and 152 |

**Database:** CDSR, DARE, HTA, CENTRAL

**Date of last search:** 29 January 2018

| # | Searches |
| --- | --- |
| #1 | MeSH descriptor: Stress Disorders, Traumatic this term only |
| #2 | MeSH descriptor: Combat Disorders this term only |
| #3 | MeSH descriptor: Psychological Trauma this term only |
| #4 | MeSH descriptor: Stress Disorders, Post-Traumatic this term only |
| #5 | MeSH descriptor: Stress Disorders, Traumatic, Acute this term only |
| #6 | MeSH descriptor: Stress, Psychological this term only |
| #7 | ("railway spine" or (rape near/2 trauma*) or reexperienc* or "re experienc*" or "torture syndrome" or "traumatic neuros*" or "traumatic stress"):ti (Word variations have been searched) |
| #8 | ("railway spine" or (rape near/2 trauma*) or reexperienc* or "re experienc*" or "torture syndrome" or "traumatic neuros*" or "traumatic stress"):ab (Word variations have been searched) |
| #9 | (trauma* and (avoidance or grief or horror or death* or nightmare* or "night mare*" or emotion*)):ti (Word variations have been searched) |
| #10 | (trauma* and (avoidance or grief or horror or death* or nightmare* or "night mare*" or emotion*)):ab (Word variations have been searched) |
| #11 | (posttraumatic* or "post traumatic*" or "stress disorder*" or "acute stress" or ptsd or asd or desnos or ("combat neuros*" or "combat syndrome" or "concentration camp syndrome" or "extreme stress" or flashback* or "flash back*" or hypervigilan* or hypervigilen* or "psych* stress" or "psych* trauma*" or psychotrauma* or psychotrauma*) or (posttrauma* or traumagenic* or "traumatic stress*")):ti (Word variations have been searched) |
| #12 | (posttraumatic* or "post traumatic*" or "stress disorder*" or "acute stress" or ptsd or asd or desnos or ("combat neuros*" or "combat syndrome" or "concentration camp syndrome" or "extreme stress" or flashback* or "flash back*" or hypervigilan* or hypervigilen* or "psych* stress" or "psych* trauma*" or psychotrauma* or psychotrauma*) or (posttrauma* or traumagenic* or "traumatic stress*")):ab (Word variations have been searched) |
| #13 | #1 or #2 or #3 or #4 or #5 or #6 or #7 or #8 or #9 or #10 or #11 or #12 |

**Database:** CINAHL PLUS

**Date of last search:** 29 January 2018

| # | Searches |
| --- | --- |
| s52 | s6 and s51 |
| s51 | s40 or s50 |
| s50 | s48 not s49 |
| s49 | (mh "animals") not (mh "human") |
| s48 | s41 or s42 or s43 or s44 or s45 or s46 or s47 |
| s47 | ti ( placebo* or random* ) or ab ( placebo* or random* ) |
| s46 | ti ( single blind* or double blind* or treble blind* or mask* or dummy* or singleblind* or doubleblind* or trebleblind* or tripleblind* ) or ab ( single blind* or double blind* or treble blind* or mask* or dummy* or singleblind* or doubleblind* or trebleblind* or tripleblind* ) |
| s45 | ti ( crossover or cross over ) or ab ( crossover or cross over ) |
| s44 | ti clinical n2 trial* or ab clinical n2 trial* |
| s43 | (mh "crossover design") or (mh "placebos") or (mh "random assignment") or (mh "random sample") |
| s42 | mw double blind* or single blind* or triple blind* |
| s41 | (mh "clinical trials+") |
| s40 | s7 or s8 or s9 or s10 or s11 or s12 or s13 or s14 or s15 or s16 or s17 or s18 or s19 or s20 or s21 or s22 or s23 or s29 or s30 or s31 or s34 or s35 or s36 or s37 or s38 or s39 |
| s39 | ti ( analy* n5 review* or evidence* n5 review* or methodol* n5 review* or quantativ* n5 review* or systematic* n5 review* ) or ab ( analy* n5 review* or assessment* n5 review* or evidence* n5 review* or methodol* n5 review* or qualitativ* n5 review* or quantativ* n5 review* or systematic* n5 review* ) |
| s38 | ti ( pool* n2 results or combined n2 results or combining n2 results ) or ab ( pool* n2 results or combined n2 results or combining n2 results ) |
| s37 | ti ( pool* n2 studies or combined n2 studies or combining n2 studies ) or ab ( pool* n2 studies or combined n2 studies or combining n2 studies ) |
| s36 | ti ( pool* n2 trials or combined n2 trials or combining n2 trials ) or ab ( pool* n2 trials or combined n2 trials or combining n2 trials ) |
| s35 | ti ( pool* n2 data or combined n2 data or combining n2 data ) or ab ( pool* n2 data or combined n2 data or combining n2 data ) |
| s34 | s32 and s33 |
| s33 | ti review* or pt review* |
| s32 | ti analy* or assessment* or evidence* or methodol* or quantativ* or qualitativ* or systematic* |
| s31 | ti “systematic* n5 search*” or ab “systematic* n5 search*” |
| s30 | ti “systematic* n5 review*” or ab “systematic* n5 review*” |
| s29 | (s24 or s25 or s26) and (s27 or s28) |
| s28 | ti systematic* or ab systematic* |
| s27 | tx review* or mw review* or pt review* |
| s26 | (mh "cochrane library") |
| s25 | ti ( bids or cochrane or embase or “index medicus” or “isi citation” or medline or psyclit or psychlit or scisearch or “science citation” or web n2 science ) or ab ( bids or cochrane or “index medicus” or “isi citation” or psyclit or psychlit or scisearch or “science citation” or web n2 science ) |
| s24 | ti ( “electronic database*” or “bibliographic database*” or “computeri?ed database*” or “online database*” ) or ab ( “electronic database*” or “bibliographic database*” or “computeri?ed database*” or “online database*” ) |
| s23 | (mh "literature review") |
| s22 | pt systematic* or pt meta* |
| s21 | ti ( “fixed effect*” or “random effect*” ) or ab ( “fixed effect*” or “random effect*” ) |
| s20 | ti ( “mantel haenszel” or peto or dersimonian or “der simonian” ) or ab ( “mantel haenszel” or peto or dersimonian or “der simonian” ) |
| s19 | ti ( handsearch* or "hand search*" or "manual search*" ) or ab ( handsearch* or "hand search*" or "manual search*" ) |
| s18 | ab "data extraction" or "data synthesis" |
| s17 | ab "selection criteria" |
| s16 | ab "relevant journals" |
| s15 | ab "published studies" |
| s14 | ab bibliograph* |
| s13 | ti "reference list*" |
| s12 | ab "reference list*" |
| s11 | ti ( “research review*” or “research integration” ) or ab ( “research review*” or “research integration” ) |
| s10 | ti ( metaanal* or “meta anal*” or metasynthes* or “meta synethes*” ) or ab ( metaanal* or “meta anal*” or metasynthes* or “meta synethes*” ) |
| s9 | (mh "meta analysis") |
| s8 | (mh "systematic review") |
| s7 | (mh "literature searching+") |
| s6 | s1 or s2 or s3 or s4 or s5 |
| s5 | ti ( (posttraumatic* or "post traumatic*" or "stress disorder*" or "acute stress" or ptsd or asd or desnos or ("combat neuros*" or "combat syndrome" or "concentration camp syndrome" or "extreme stress" or flashback* or "flash back*" or hypervigilan* or hypervigilen* or "psych* stress" or "psych* trauma*" or psychotrauma* or psychotrauma*) or (posttrauma* or traumagenic* or "traumatic stress*")) ) or ab ( (posttraumatic* or "post traumatic*" or "stress disorder*" or "acute stress" or ptsd or asd or desnos or ("combat neuros*" or "combat syndrome" or "concentration camp syndrome" or "extreme stress" or flashback* or "flash back*" or hypervigilan* or hypervigilen* or "psych* stress" or "psych* trauma*" or psychotrauma* or psychotrauma*) or (posttrauma* or traumagenic* or "traumatic stress*")) ) |
| s4 | ti ( (trauma* and (avoidance or grief or horror or death* or nightmare* or "night mare*" or emotion*)) ) or ab ( (trauma* and (avoidance or grief or horror or death* or nightmare* or "night mare*" or emotion*)) ) |
| s3 | ti ( ("railway spine" or (rape near/2 trauma*) or reexperienc* or "re experienc*" or "torture syndrome" or "traumatic neuros*" or "traumatic stress") ) or ab ( ("railway spine" or (rape near/2 trauma*) or reexperienc* or "re experienc*" or "torture syndrome" or "traumatic neuros*" or "traumatic stress") ) |
| s2 | (mh "stress, psychological") |
| s1 | (mh "stress disorders, post-traumatic") |
