# Supplementary material for: Cost-effectiveness of psychological treatments for post-traumatic stress disorder in adults
Source: PLoS One. 2020 Apr 30;15(4):e0232245. doi: 10.1371/journal.pone.0232245 (PMC7192458; doi:10.1371/journal.pone.0232245)
Supplement: S2 Appendix — (DOCX) [file pone.0232245.s009.docx]

# **Appendix 2: Study protocol**

## Systematic review of psychological, psychosocial and other non-pharmacological interventions for the treatment of PTSD in adults

| Topic | Psychological, psychosocial and other non-pharmacological interventions for the treatment of PTSD in adults |
| --- | --- |
| Review question(s) | For adults with clinically important post-traumatic stress symptoms, what are the relative benefits and harms of psychological, psychosocial or other non-pharmacological interventions targeted at PTSD symptoms? |
| Sub-question(s) | Where evidence exists, consideration will be given to the specific needs of:   - women who have been exposed to sexual abuse or assault, or domestic violence - lesbian, gay, bisexual, transsexual or transgender people - people from black and minority ethnic groups - people who are homeless or in insecure accommodation - asylum seekers or refugees or other immigrants who are entitled to NHS treatment - people who have been trafficked - people who are socially isolated (and who are not captured by any other subgroup listed) - people with complex PTSD - people with neurodevelopmental disorders (including autism) - people with coexisting conditions (drug and alcohol misuse, common mental health disorders, eating disorders, personality disorders, acquired brain injury, physical disabilities and sensory impairments) - people who are critically ill or injured (for instance after a vehicle crash) |
| Objectives | To identify the most effective psychological, psychosocial or other non-pharmacological interventions for the treatment of PTSD in adults |
| Population | Adults with PTSD (as defined by a diagnosis of PTSD according to DSM, ICD or similar criteria, or clinically-significant PTSD symptoms as indicated by baseline scores above threshold on a validated scale more than one month after the traumatic event [see PTSD scales listed under outcomes])  For mixed adult and children populations, where possible disaggregated data will be obtained. If this is not possible then the study will be categorised according to the mean age of the population (<18 years as children and young people and ≥18 years as adult).  If some, but not all, of a study’s participants are eligible for the review, where possible disaggregated data will be obtained. If this is not possible then the study will be included if at least 80% of its participants are eligible for this review. |
| Exclude | Trials of people with adjustment disorders  Trials of people with traumatic grief  Trials of people with psychosis as a coexisting condition  Trials of people with learning disabilities  Trials of women with PTSD during pregnancy or in the first year following childbirth  Trials of adults in contact with the criminal justice system (not solely as a result of being a witness or victim) |
| Intervention | Psychological interventions (psychological interventions listed below are examples of interventions which may be included either alone or in combination in an individual or group format):   - Trauma-focused cognitive behavioural therapies (CBT), including cognitive therapy, cognitive processing therapy, compassion focused therapy, exposure therapy/prolonged exposure (PE), virtual reality exposure therapy (VRET), imagery rehearsal therapy, mindfulness-based cognitive therapy (MBCT) and narrative exposure therapy (NET) - Non-trauma-focused CBT, including stress inoculation training (SIT) - Psychologically-focused debriefing (including single session debriefing) - Eye movement desensitisation and reprocessing (EMDR) - Hypnotherapy - Psychodynamic therapies, including traumatic incident reduction (TIR) - Counselling, including non-directive/supportive/person-centred counselling - Human givens therapy - Combined somatic and cognitive therapies, including thought field therapy (TFT) and emotional freedom technique (EFT) - Couple interventions, including cognitive-behavioural conjoint therapy - Parent training/family interventions, including behavioural family therapy   Psychosocial interventions (psychosocial interventions listed below are examples of interventions which may be included either alone or in combination):   - Meditation - Mindfulness-based stress reduction (MBSR) - Supported employment (including individual placement and support [IPS] supported employment and Veterans Health Administration Vocational Rehabilitation Programme [VRP]) - Practical support (including financial and housing) - Psychoeducational interventions - Peer support (including (including self-help groups and support groups and Trauma Risk Management [TRiM])   Other non-pharmacological interventions (other non-pharmacological interventions listed below are examples of interventions which may be included either alone or in combination):   - Acupuncture (including classical acupuncture, electroacupuncture, auricular acupuncture, laser acupuncture and acupoint stimulation [such as acupressure, moxibustion and tapping]) - Exercise (including anaerobic [such as heavy weight training, sprinting, high-intensity interval training] and aerobic [such as running/jogging, swimming, cycling and walking] exercise, both supervised and unsupervised) - Repetitive transcranial magnetic stimulation (rTMS) - Yoga (including all types of yoga)   Combination interventions, such as combined psychological plus pharmacological versus pharmacological alone, will also be considered here.  A distinction will be made between early interventions (delivered within 3 months of the traumatic event) and delayed interventions (delivered more than 3 months after the traumatic event)  Exclude:  Inoculation interventions for people who may be at risk of experiencing but have not experienced, a traumatic event  Interventions that are not targeted at PTSD symptoms |
| Comparison | Any other intervention  Treatment as usual  Waitlist  Placebo |
| Critical outcomes | Efficacy  PTSD symptomology (mean endpoint score or change in PTSD score from baseline)  Diagnosis of PTSD (number of people meeting diagnostic criteria for PTSD according to DSM, ICD or similar criteria)  Recovery from PTSD/Remission (number of people no longer meeting diagnostic criteria for PTSD according to DSM, ICD or similar criteria at endpoint, or endpoint scores below threshold on a validated scale)  Response (as measured by an agreed percentage improvement in symptoms and/or by a dichotomous rating of much or very much improved on Clinical Global Impressions [CGI] scale)  Relapse (number of people who remitted at endpoint but at follow-up either met diagnostic criteria for PTSD according to DSM, ICD or similar criteria, or whose follow-up scores were above threshold on a validated scale)  The following PTSD scales will be included:  Assessor-rated PTSD symptom scales:   - Clinician-Administered PTSD Scale for DSM–IV (CAPS) or DSM-V (CAPS-5) - Anxiety Disorders Interview Schedule for DSM-IV: Lifetime version (ADIS-IV-L) or DSM-5 (ADIS-5) - Adult and Lifetime Version - PTSD Symptom Scale – Interview Version (PSS-I) - Number of symptoms on the Structured Clinical Interview for DSM-IV (SCID) - Symptoms of Trauma Scale (SOTS)   Self-report instruments of PTSD symptoms:   - PTSD Checklist (PCL), including all versions (PCL-5, PCL-M, PCL-C and PCL-S) - PTSD Symptom Scale – Self Report (PSS-SR) - Life Events Checklist for DSM-5 (LEC-5) - Trauma Screening Questionnaire (TSQ) - Primary Care PTSD Screen (PC-PTSD) - Davidson Trauma Scale (DTS) - Post-Traumatic Diagnostic Scale (PDS) - Impact of Event Scale (IES)/Impact of Event Scale Revised (IES-R)   Acceptability/tolerability  Acceptability of the intervention  Discontinuation due to adverse effects  Discontinuation due to any reason (including adverse effects) |
| Important, but not critical outcomes | Dissociative symptoms as assessed with a validated scale including:  Assessor-rated scales:  Dissociation symptom cluster score on CAPS  Self-report (parent-report) scales:  Dissociative Experiences Scale (DES)  Multiscale Dissociation Inventory (MDI)  Traumatic Dissociation Scale  Personal, social, educational and occupational functioning  Sleeping difficulties (as assessed with a validated scale, including the Pittsburgh Sleep Quality Index Addendum for PTSD [PSQI-A] and Insomnia Severity Index [ISI])  Employment (for instance, number in paid employment)  Housing (for instance, number homeless or in insecure accommodation)  Functional impairment (as assessed with a validated scale including the Work and Social Adjustment Scale [WSAS])  Relationship difficulties (with spouse and/or children)  Quality of life (as assessed with a validated scale including the 36-item Short-Form Survey [SF-36] and Warwick-Edinburgh Mental Well-being Scale [WEMWBS])  Coexisting conditions (note that target of intervention should be PTSD symptoms)  Symptoms of and recovery from a coexisting condition  Self-harm  Suicide |
| Study design | Systematic reviews of RCTs  RCTs |
| Include unpublished data? | Clinical trial registries (ISRCTN and ClinicalTrials.gov) will be searched to identify any relevant unpublished trials and authors will be contacted to request study reports (where these are not available online). Unpublished data will only be included where a full study report is available with sufficient detail to properly assess the risk of bias. Authors of unpublished evidence will be asked for permission to use such data, and will be informed that summary data from the study and the study’s characteristics will be published in the full guideline  Conference abstracts and dissertations will not be included. |
| Restriction by date? | All relevant studies from existing reviews from the 2005 guideline will be carried forward. No restriction on date for the updated search. |
| Minimum sample size | N = 10 in each arm |
| Study setting | Primary, secondary, tertiary, social care and community settings.  Treatment provided to troops on operational deployment or exercise will not be covered. |
| The review strategy | Reviews  If existing systematic reviews are found, the committee will assess their quality, completeness, and applicability to the NHS and to the scope of the guideline. If the committee agrees that a systematic review appropriately addresses a review question, a search for studies published since the review will be conducted.  Data Extraction (selection and coding)  Citations from each search will be downloaded into EndNote and duplicates removed. Titles and abstracts of identified studies will be screened by two reviewers for inclusion against criteria, until a good inter-rater reliability has been observed (percentage agreement =>90% or Kappa statistics, K>0.60). Initially 10% of references will be double-screened. If inter-rater agreement is good then the remaining references will be screened by one reviewer. All primary-level studies included after the first scan of citations will be acquired in full and re-evaluated for eligibility at the time they are being entered into a study database (standardised template created in Microsoft Excel). At least 10% of data extraction will be double-coded. Discrepancies or difficulties with coding will be resolved through discussion between reviewers or the opinion of a third reviewer will be sought.  Non-English-language papers will be excluded (unless data can be obtained from an existing review).  Data Analysis  Where data is available, meta-analysis using a fixed-effects model will be used to combine results from similar studies. Heterogeneity will be considered and if a random-effects model is considered more appropriate it will be conducted.  For risk of bias, outcomes will be downgraded if the randomisation and/or allocation concealment methods are unclear or inadequate. Outcomes will also be downgraded if no attempts are made to blind the assessors or participants in some way, i.e. by either not knowing the aim of the study or the result from other tests. Outcomes will also be downgraded if there is considerable missing data (see below).  Handling missing data:  Where possible an intention to treat approach will be used.  Outcomes will be downgraded if there is a dropout of more than 20%, or if there was a difference of >20% between the groups.  For heterogeneity: outcomes will be downgraded once if I2>50%, twice if I2 >80%  For imprecision: outcomes will be downgraded if:   - Step 1: If the 95% CI is imprecise i.e. crosses 0.8 or 1.25 (dichotomous) or -0.5 or 0.5 (for continuous). Outcomes will be downgraded one or two levels depending on how many lines it crosses. - Step 2: If the clinical decision threshold is not crossed, we will consider whether the criterion for Optimal Information Size is met, if not we will downgrade one level for the following:   - - for dichotomous outcomes: <300 events     - for continuous outcomes: <400 participants   For clinical effectiveness, if studies report outcomes using the same scale mean differences will be considered, if not standardized mean differences (SMDs) will be considered and the following criteria will be used:   - SMD <0.2 too small to likely show an effect - SMD 0.2 small effect - SMD 0.5 moderate effect - SMD 0.8 large effect - RR <0.8 or >1.25 clinical benefit   Anything less (RR >0.8 and <1.25), the absolute numbers will be looked at to make a decision on whether there may be a clinical effect. |
| Heterogeneity  (sensitivity analysis and subgroups) | Where substantial heterogeneity exists, sensitivity analyses will be considered, for instance:   - Studies with <50% completion data (drop out of >50%) will be excluded,   Where possible, the influence of subgroups will be considered, including subgroup analyses giving specific consideration to the groups outlined in the sub-question section and to the following groups:   - Trauma type (including single incident relative to chronic exposure) - Duration of intervention (for instance, short-term [≤12 weeks] relative to long-term [>12 weeks]) - Intensity of intervention (for instance, low intensity [≤15 sessions] relative to high intensity [>15 sessions]) - Format of intervention (individual relative to group) - Mode of intervention delivery (including digital relative to face-to-face) - First-line treatment relative to second-line treatment and treatment-resistant PTSD (≥2 inadequate treatments)   Acute PTSD symptoms (clinically important PTSD symptoms for less than 3 months) relative to chronic PTSD symptoms (clinically important PTSD symptoms for 3 months or more) |
| Notes | Practical and social support (area of scope) is covered quantitatively by interventions listed under psychosocial interventions:   - Supported employment (including individual placement and support [IPS] supported employment and Veterans Health Administration Vocational Rehabilitation Programme [VRP]) - Practical support (including financial and housing) - Peer support (including self-help groups and support groups) |

## Additional criteria applied for the network meta-analysis (population – interventions – outcomes)

| Topic | Psychological interventions for the treatment of PTSD in adults |
| --- | --- |
| Population | Adults with clinically important post-traumatic stress symptoms more than three months after a traumatic event, defined by a diagnosis of PTSD according to DSM, ICD or similar criteria or clinically-significant PTSD symptoms as indicated by baseline scores above threshold on a validated scale |
| Interventions | - Psychological interventions aimed at reducing post-traumatic stress symptoms - Hypnotherapy, meditation, mindfulness-based stress reduction, supported employment, peer support, practical support, relaxation, exercise, yoga, acupuncture, bio-neuro-feedback and repetitive transcranial magnetic stimulation are not part of the decision problem and will be considered only if they serve as comparators to psychological interventions and provide links in the network - Pharmacological and combined psychological and pharmacological interventions that have been compared with psychological interventions and are thus linked in the network will be considered - To be included in the network meta-analysis, interventions need to be linked to the network. - Trauma-focused cognitive behavioural therapy (TF-CBT) interventions will be analysed together, as a class. - Only ‘pure’ interventions or their combinations (i.e. interventions or their combinations delivered as the sole treatment in a trial arm, rather than being added on treatment as usual [TAU]) will be considered. - TAU will not be included even if it can provide links in the network, because it is a heterogeneous comparator and varies widely across trials and settings. |
| Outcomes | PTSD symptomology (change in PTSD score from baseline)  Self-rated scales are prioritised over clinician-rated ones, if both are available in a study.  Recovery from PTSD/Remission (number of people no longer meeting diagnostic criteria for PTSD according to DSM, ICD or similar criteria at endpoint, or endpoint scores below threshold on a validated scale) |
