# Supplementary material for: Cost-effectiveness of psychological treatments for post-traumatic stress disorder in adults
Source: PLoS One. 2020 Apr 30;15(4):e0232245. doi: 10.1371/journal.pone.0232245 (PMC7192458; doi:10.1371/journal.pone.0232245)
Supplement: S3 Appendix — (DOCX) [file pone.0232245.s010.docx]

# **Appendix 3: Details of the statistical analysis and WinBUGS codes for data synthesis**

## Details of the statistical analysis

NMAs were conducted within a Bayesian framework using Markov Chain Monte Carlo simulation techniques implemented in WinBUGS 1.4.3 (Lunn *et al.* 2000; Spiegelhalter *et al.* 2003). Two different sets of initial values were used when running each model; convergence was assessed by visually inspecting the mixing of the two chains in the history plots and the Brooks Gelman-Rubin diagram (Brooks and Gelman, 1998).

For the synthesis of continuous data (changes in PTSD symptom score), a generalised linear model (GLM) with a normal likelihood and identity link was used (Dias *et al.* 2013a; Dias *et al.* 2018). Because the RCTs included in the NMAs used different continuous scales to report change in PTSD symptoms, pooling of the differences in means across different scales was not appropriate. For this reason results were expressed in the form of the Standardised Mean Difference (SMD), where the mean difference is divided by a standardising constant, which can be the population standard deviation for each scale (if known), or its estimate (Cooper *et al.* 2009). In the NMAs of continuous data, this was estimated in each study by pooling the estimated standard deviations across all arms of the study. This SMD is known as Cohen’s d (Cohen, 1969).

The suitability of both fixed and random effect models was assessed and compared. The goodness of fit of each model to the data was assessed by comparing the posterior mean of the residual deviance, which measures the magnitude of the differences between the observed data and the model predictions of the data, with the number of data points in the model (Dempster, 1997). Smaller values of the residual deviance are preferred, and in a well-fitting model the posterior mean residual deviance should be close to the number of data points in the analysis (each study arm contributes one data point) (Spiegelhalter *et al.* 2002). Models were also compared using the deviance information criterion (DIC), a measure of model fit that is penalised by model complexity. It is equal to the sum of the posterior mean deviance and the effective number of parameters; lower values are preferred and typically differences of at least 3 points are considered meaningful (Dias *et al.* 2013a; Spiegelhalter *et al.* 2002). The posterior median between-study standard deviation, which measures the heterogeneity of treatment effects estimated by trials within contrasts, was also used to compare models. When fitting random effects models, it is important to assess whether there is enough evidence informing the between-study standard deviation. This was done by comparing the prior and posterior distributions of the between-study standard deviation. In addition, the magnitude of heterogeneity was considered.

For both NMAs of changes in PTSD symptom scores, a random effects model was fitted with a Uniform(0,5) prior being given to the between-study standard deviation. Non-informative normal prior distributions Normal(0,10000) were assigned to all other parameters, i.e. trial baselines and treatment effects (Dias *et al.* 2013a).

The NMAs that utilised PTSD symptom change scores subsequently informed an economic analysis described in a companion paper (Mavranezouli *et al.* under review). The economic analysis required the outcome to be reported in the form of a probability of effect (remission). SMDs, which were the output of these NMAs, cannot be directly used to estimate these probabilities. However, it was possible to transform the results of the NMAs, expressed on the SMD scale, to a log-odds ratio (LOR) of effect using the following formula (Chinn, 2000):

$$LOR= -\frac{\pi}{\sqrt{3}} SMD$$

This transformation assumes that the remission status is determined based on a scale with an underlying normal distribution that has been dichotomised into a PTSD diagnosis versus no PTSD diagnosis (‘remission’) using a hypothetical cut-off point on the scale.

For the synthesis of dichotomous data (remission), a binomial likelihood and logit link model was used (Dias *et al.* 2013a; Dias *et al.* 2018). The output of this analysis was the LORs between all pairs of interventions assessed. The suitability of both fixed and random effect models was assessed and compared in a similar manner described for the analysis of continuous outcomes above. In the random effects model the prior for the between-study standard deviation was Uniform(0,5) and non-informative normal prior distributions Normal(0,10000) were assigned to all other parameters, i.e. trial baselines and treatment effects (Dias *et al.* 2013a).

We note that our modelling framework permits the inclusion of zero cells, so typically a continuity correction (e.g. adding 0.5 to the number of events and 1 to number of individuals, when the number of events is zero) was not needed. A continuity correction may be helpful when there are many small trials and trials with zero cells, resulting in numerical instability or slow convergence (Dias *et al.*  2013a; Dias *et al.*  2018). For the remission outcome, this was not an issue and models were run using the raw data.

## WinBUGS code for synthesis of changes in PTSD symptom scores (random and fixed effect models) [Dias *et al.* 2013a]

| Normal likelihood and identity link model |
| --- |
| **RANDOM EFFECTS MODEL**  # Normal likelihood, identity link: SMD with arm-based means;  # output as log Odds Ratios  # Random effects model for multi-arm trials  model{ # *** PROGRAM STARTS  for(i in 1:ns){ # LOOP THROUGH STUDIES  w[i,1] <- 0 # adjustment for multi-arm trials is zero for control arm  delta[i,1] <- 0 # treatment effect is zero for control arm  mu[i] ~ dnorm(0,.0001) # vague priors for all trial baselines  }  # CONTINUOUS DATA AS ARM MEANS  for(i in 1:ns){  # calculate pooled.sd and adjustment for SMD  df[i] <- sum(n[i,1:na[i]]) - na[i] # denominator for pooled.var  Pooled.var[i] <- sum(nvar[i,1:na[i]])/df[i]  Pooled.sd[i] <- sqrt(Pooled.var[i]) # pooled sd for study i, for SMD  # H[i] <- 1 - 3/(4*df[i]-1) # use Hedges' g  H[i] <- 1 # use Cohen's d (ie no adjustment)  for (k in 1:na[i]){  se[i,k] <- sd[i,k]/sqrt(n[i,k])  var[i,k] <- pow(se[i,k],2) # calculate variances  prec[i,k] <- 1/var[i,k] # set precisions  y[i,k] ~ dnorm(phi[i,k], prec[i,k]) # normal likelihood  phi[i,k] <- theta[i,k] * (Pooled.sd[i]/H[i]) # theta is standardised mean  theta[i,k] <- mu[i] + delta[i,k] # model for linear predictor, delta is SMD  dev[i,k] <- (y[i,k]-phi[i,k])*(y[i,k]-phi[i,k])*prec[i,k]  nvar[i,k] <- (n[i,k]-1) * pow(sd[i,k],2) # for pooled.sd  }  # summed residual deviance contribution for this trial  resdev[i] <- sum(dev[i,1:na[i]])  }  # RE MODEL  for(i in 1:ns){ # LOOP THROUGH ALL STUDIES  for (k in 2:na[i]){ # LOOP THROUGH ARMS  # trial-specific RE distributions  delta[i,k] ~ dnorm(md[i,k], taud[i,k])  md[i,k] <- d[t[i,k]] - d[t[i,1]] + sw[i,k]  # precision of RE distributions (with multi-arm trial correction)  taud[i,k] <- tau *2*(k-1)/k  # adjustment, multi-arm RCTs  w[i,k] <- delta[i,k] - d[t[i,k]] + d[t[i,1]]  # cumulative adjustment for multi-arm trials  sw[i,k] <-sum(w[i,1:k-1])/(k-1)  }  }  #  totresdev <- sum(resdev[]) # Total Residual Deviance (all data)  # Priors distributions  d[1]<-0 # treatment effect is zero for control arm  # vague prior for treatment effects  for (k in 2:nt){ d[k] ~ dnorm(0, .0001) }  sdev ~ dunif(0,5) # vague prior for between-trial SD  tau <- pow(sdev,-2) # between-trial precision  for (c in 1:(nt-1)){  for (k in (c+1):nt){  diff[c,k] <- d[k] - d[c] # all pairwise differences (SMD)  lor[c,k] <- diff[c,k]*(-3.1416/sqrt(3)) # convert to lor (note sign)  }  }  # rank treatments  for (k in 1:nt) {  rk[k] <- rank(d[],k)  best[k] <- equals(rk[k],1) # Smallest is best (i.e. rank 1)  # prob treat k is h-th best, prob[1,k]=best[k]  for (h in 1:nt) { prob[h,k] <- equals(rk[k],h) }  }  **Only rank treatments with N≥100**  **- changes in PTSD symptom scale scores between baseline and treatment endpoint**  # 1 Waitlist 1312; 2 Attention placebo 221; 3 Psychoeducation 152; 4 Relaxation 25; 5 Counselling 278;  # 6 TF-CBT 903; 7 non-TF-CBT 209; 8 EMDR 260; 9 Present-centered therapy 99; 10 IPT 55; 11 Metacognitive therapy 10;  # 12 Combined somatic/cognitive therapies 237; 13 Resilience-oriented treatment 20; 14 Attention bias modification 83;  # 15 Couple intervention 22; 16 Self-help with support 198; 17 Self-help without support 335; 18 SSRI 166; 19 TF-CBT + SSRI 115  for(k in 1:3){ dR[k]<-d[k] }  for(k in 4:7){ dR[k]<-d[k+1] }  for(k in 8:8){ dR[k]<-d[k+4] }  for(k in 9:12){ dR[k]<-d[k+7] }  for (k in 1:12) {  rk2[k] <- rank(dR[],k)  best2[k] <- equals(rk2[k],1) # Smallest is best (i.e. rank 1)  # prob treat k is h-th best, prob[1,k]=best[k]  for (h in 1:12) { prob2[h,k] <- equals(rk2[k],h) }  }  **- changes in PTSD symptom scale scores between baseline and 1-4-month follow-up**  # 1 Waitlist 496; 2 Attention placebo 44; 3 Psychoeducation 183; 4 Counselling 205; 5 TF-CBT 753; 6 non-TF-CBT 123; 7 EMDR 121;  # 8 Present-centered therapy 70; 9 Combined somatic/cognitive therapies 23; 10 IPT 32; 11 Couple intervention 21;  # 12 Self-help with support 85; 13 Self-help without support 40; 14 Family therapy 72; 15 Behavioural therapy 47  for(k in 1:1){ dR[k]<-d[k] }  for(k in 2:6){ dR[k]<-d[k+1] }  for (k in 1:6) {  rk2[k] <- rank(dR[],k)  best2[k] <- equals(rk2[k],1) # Smallest is best (i.e. rank 1)  # prob treat k is h-th best, prob[1,k]=best[k]  for (h in 1:6) { prob2[h,k] <- equals(rk2[k],h) }  }  } # *** PROGRAM ENDS  **Initial values for each chain**  **- changes in PTSD symptom scale scores between baseline and treatment endpoint**  # chain 1  list(d = c(NA,0,0,0,0, 0,0,0,0,0, 0,0,0,0,0, 0,0,0,0),  mu = c(0,0,0,0,0, 0,0,0,0,0, 0,0,0,0,0, 0,0,0,0,0, 0,0,0,0,0, 0,0,0,0,0, 0,0,0,0,0, 0,0,0,0,0, 0,0,0,0,0, 0,0,0,0,0, 0,0,0,0,0, 0,0,0,0,0, 0,0,0,0,0, 0,0,0,0,0, 0), sdev = 1)  # chain 2  list(d = c(NA,-1,1,1,-0.5, 1,1,1,-1,-0.7, 1,-1,0.5,0.7,-1, -1,0.5,-0.5,1),  mu = c(0.5,1,0.7,1,-1, -0.5,0,1,-0.5,-1, 0.7,1,-0.7,0.5,0.6, -0.4,1,-1,0.5,-1, 1,-0.5,-1,-0.7,0.7, 0.6,-0.5,-0.6,1,-0.4, 0.5,1,0.7,1,-1, -0.5,0,1,-0.5,-1, 0.7,1,-0.7,0.5,0.6, -0.4,1,-1,0.5,-1, 1,-0.5,-1,-0.7,0.7, 0.6,-0.5,-0.6,1,-0.4, 0.5,1,0.7,1,-1, -0.5,0,1,-0.5,-1, 0.7), sdev = 0.7)  **- changes in PTSD symptom scale scores between baseline and 1-4-month follow-up**  # chain 1  list(d = c(NA,0,0,0,0, 0,0,0,0,0, 0,0,0,0,0),  mu = c(0,0,0,0,0, 0,0,0,0,0, 0,0,0,0,0, 0,0,0,0,0, 0,0,0,0,0, 0,0,0), sdev = 1)  # chain 2  list(d = c(NA,-1,1,1,-0.5, 1,1,1,-1,-0.7, -1,0.5,1,0.7,-0.3),  mu = c(0.5,1,0.7,1,-1, -0.5,0,1,-0.5,-1, -1,-1,-0.5,0.5,1, 1,1,1,-1,-0.7, -1,0.5,1,0.5,-1, 0.5,0.3,-0.7), sdev = 0.5) |
| **FIXED EFFECT MODEL**  # Normal likelihood, identity link: SMD with arm-based means;  # output as log Odds Ratios  # Fixed effect model  model{ # *** PROGRAM STARTS  for(i in 1:ns){ # LOOP THROUGH STUDIES  mu[i] ~ dnorm(0,.0001) # vague priors for all trial baselines  # CONTINUOUS DATA AS ARM MEANS  # calculate pooled.sd and adjustment for SMD  df[i] <- sum(n[i,1:na[i]]) - na[i] # denominator for pooled.var  Pooled.var[i] <- sum(nvar[i,1:na[i]])/df[i]  Pooled.sd[i] <- sqrt(Pooled.var[i]) # pooled sd for study i, for SMD  # H[i] <- 1 - 3/(4*df[i]-1) # use Hedges' g  H[i] <- 1 # use Cohen's d (ie no adjustment)  for (k in 1:na[i]){  se[i,k] <- sd[i,k]/sqrt(n[i,k])  var[i,k] <- pow(se[i,k],2) # calculate variances  prec[i,k] <- 1/var[i,k] # set precisions  y[i,k] ~ dnorm(phi[i,k], prec[i,k]) # normal likelihood  phi[i,k] <- theta[i,k] * (Pooled.sd[i]/H[i]) # theta is standardised mean  theta[i,k] <- mu[i] + d[t[i,k]] - d[t[i,1]] # model for linear predictor  dev[i,k] <- (y[i,k]-phi[i,k])*(y[i,k]-phi[i,k])*prec[i,k]  nvar[i,k] <- (n[i,k]-1) * pow(sd[i,k],2) # for pooled.sd  }  # summed residual deviance contribution for this trial  resdev[i] <- sum(dev[i,1:na[i]])  }  totresdev <- sum(resdev[]) # Total Residual Deviance (all data)  # Priors distributions  d[1]<-0 # treatment effect is zero for control arm  # vague prior for treatment effects  for (k in 2:nt){ d[k] ~ dnorm(0, .0001) }  for (c in 1:(nt-1)){  for (k in (c+1):nt){  diff[c,k] <- d[k] - d[c] # all pairwise differences (SMD)  lor[c,k] <- diff[c,k]*(-3.1416/sqrt(3)) # convert to lor (note sign)  }  }  # rank treatments  for (k in 1:nt) {  rk[k] <- rank(d[],k)  best[k] <- equals(rk[k],1) # Smallest is best (i.e. rank 1)  # prob treat k is h-th best, prob[1,k]=best[k]  for (h in 1:nt) { prob[h,k] <- equals(rk[k],h) }  }  } # *** PROGRAM ENDS  **Initial values for each chain**  **- changes in PTSD symptom scale scores between baseline and treatment endpoint**  # chain 1  list(d = c(NA,0,0,0,0, 0,0,0,0,0, 0,0,0,0,0, 0,0,0,0),  mu = c(0,0,0,0,0, 0,0,0,0,0, 0,0,0,0,0, 0,0,0,0,0, 0,0,0,0,0, 0,0,0,0,0, 0,0,0,0,0, 0,0,0,0,0, 0,0,0,0,0, 0,0,0,0,0, 0,0,0,0,0, 0,0,0,0,0, 0,0,0,0,0, 0,0,0,0,0, 0))  # chain 2  list(d = c(NA,-1,1,1,-0.5, 1,1,1,-1,-0.7, 1,-1,0.5,0.7,-1, -1,0.5,-0.5,1),  mu = c(0.5,1,0.7,1,-1, -0.5,0,1,-0.5,-1, 0.7,1,-0.7,0.5,0.6, -0.4,1,-1,0.5,-1, 1,-0.5,-1,-0.7,0.7, 0.6,-0.5,-0.6,1,-0.4, 0.5,1,0.7,1,-1, -0.5,0,1,-0.5,-1, 0.7,1,-0.7,0.5,0.6, -0.4,1,-1,0.5,-1, 1,-0.5,-1,-0.7,0.7, 0.6,-0.5,-0.6,1,-0.4, 0.5,1,0.7,1,-1, -0.5,0,1,-0.5,-1, 0.7))  **- changes in PTSD symptom scale scores between baseline and 1-4-month follow-up**  # chain 1  list(d = c(NA,0,0,0,0, 0,0,0,0,0, 0,0,0,0,0),  mu = c(0,0,0,0,0, 0,0,0,0,0, 0,0,0,0,0, 0,0,0,0,0, 0,0,0,0,0, 0,0,0))  # chain 2  list(d = c(NA,-1,1,1,-0.5, 1,1,1,-1,-0.7, -1,0.5,1,0.7,-0.3),  mu = c(0.5,1,0.7,1,-1, -0.5,0,1,-0.5,-1, -1,-1,-0.5,0.5,1, 1,1,1,-1,-0.7, -1,0.5,1,0.5,-1, -0.5,0.3,-0.7)) |

## WinBUGS code for synthesis of dichotomous remission data at treatment endpoint (random and fixed effect models) [Dias *et al.* 2013a]

| Binomial likelihood and logit link model |
| --- |
| **RANDOM EFFECTS MODEL**  # Binomial likelihood, logit link  # Random effect model, multi-arm trials  model{ # *** PROGRAM STARTS  for(i in 1:ns){ # LOOP THROUGH STUDIES  w[i,1] <- 0 # adjustment for multi-arm trials is zero for control arm  delta[i,1] <- 0 # treatment effect is zero for control arm  mu[i] ~ dnorm(0,.0001) # vague priors for all trial baselines  for (k in 1:na[i]) { # LOOP THROUGH ARMS  r[i,k] ~ dbin(p[i,k],n[i,k]) # binomial likelihood  logit(p[i,k]) <- mu[i] + delta[i,k] # model for linear predictor  rhat[i,k] <- p[i,k] * n[i,k] # expected value of the numerators  dev[i,k] <- 2 * (r[i,k] * (log(r[i,k])-log(rhat[i,k])) #Deviance contribution  + (n[i,k]-r[i,k]) * (log(n[i,k]-r[i,k]) - log(n[i,k]-rhat[i,k])))  }  resdev[i] <- sum(dev[i,1:na[i]]) # summed residual deviance contribution for this trial  for (k in 2:na[i]) { # LOOP THROUGH ARMS  delta[i,k] ~ dnorm(md[i,k],taud[i,k]) # trial-specific LOR distributions  md[i,k] <- d[t[i,k]] - d[t[i,1]] + sw[i,k] # mean of LOR distributions (with multi-arm correction)  taud[i,k] <- tau *2*(k-1)/k # precision of LOR distributions (with multi-arm correction)  w[i,k] <- (delta[i,k] - d[t[i,k]] + d[t[i,1]]) # adjustment for multi-arm RCTs  sw[i,k] <- sum(w[i,1:k-1])/(k-1) # cumulative adjustment for multi-arm trials  }  }  totresdev <- sum(resdev[]) #Total Residual Deviance  d[1]<- 0 # treatment effect is zero for reference treatment  for (k in 2:nt) { d[k] ~ dnorm(0,.0001)} # vague priors for treatment effects  sd ~ dunif(0,2)  tau <- pow(sd,-2)  # pairwise ORs and LORs for all possible pair-wise comparisons  for (c in 1:(nt-1)) { for (k in (c+1):nt) {  or[c,k] <- exp(d[k] - d[c])  lor[c,k] <- (d[k]-d[c])  }  }  # ranking  for (k in 1:nt) {  rk[k] <- nt+1-rank(d[],k) # assumes events are “good”  best[k] <- equals(rk[k],1) #calculate probability that treat k is best  }  **Only rank treatments with N≥100**  # 1 WaitlisT 625; 2 Attention placebo 23; 3 Relaxation 57; 4 Psychoeducation 28; 5 Counselling 150; 6 TF-CBT 601;  # 7 non-TF-CBT 65; 8 EMDR 132; 9 IPT 72; 10 Present-centred therapy 75; 11 Psychodynamic therapy 49;  # 12 Couple intervention 49; 13 Self-help with support 105; 14 Self-help without support 74; 15 SSRI 87; 16 TF-CBT + SSRI 57  for(k in 1:1){ dR[k]<-d[k] }  for(k in 2:3){ dR[k]<-d[k+3] }  for(k in 4:4){ dR[k]<-d[k+4] }  for(k in 5:5){ dR[k]<-d[k+8] }  for (k in 1:5) {  rk2[k] <- 5+1-rank(dR[],k) # assumes events are “good”  best2[k] <- equals(rk2[k],1) # Smallest is best (i.e. rank 1)  # prob treat k is h-th best, prob[1,k]=best[k]  for (h in 1:5) { prob2[h,k] <- equals(rk2[k],h) }  } # *** PROGRAM ENDS  **Initial values for each chain**  # chain 1  list(d=c(NA,0,0,0,0, 0,0,0,0,0, 0,0,0,0,0, 0), sd=1,  mu=c(0,0,0,0,0, 0,0,0,0,0, 0,0,0,0,0, 0,0,0,0,0, 0,0,0,0,0, 0,0,0,0,0, 0,0,0,0))  # chain 2  list(d=c(NA,0.1,-1,-0.2,1, 0.1,1,-0.5,-1,0.4, -1,0.5,-0.6,0.7,0.6, -0.3), sd=0.5,  mu=c(1,-1,-2,0,0, -2,1,0,2,1, 0.1,1,-0.5,-1,0.4, -1,0.5,-0.6,0.7,0.6, -0.3,0.5,-0.8,1,-0.3, -1,-1,0.7,-0.3,0.8, 0.7,-0.6,0.9,-0.3)) |
| **FIXED EFFECTS MODEL**  # Binomial likelihood, logit link, MTC  # Fixed effect model  model{ # *** PROGRAM STARTS  for(i in 1:ns){ # LOOP THROUGH STUDIES  mu[i] ~ dnorm(0,.0001) # vague priors for all trial baselines  for (k in 1:na[i]) { # LOOP THROUGH ARMS  r[i,k] ~ dbin(p[i,k],n[i,k]) # binomial likelihood  logit(p[i,k]) <- mu[i] + d[t[i,k]]-d[t[i,1]] # model for linear predictor  rhat[i,k] <- p[i,k] * n[i,k] # expected value of the numerators  dev[i,k] <- 2 * (r[i,k] * (log(r[i,k])-log(rhat[i,k])) #Deviance contribution  + (n[i,k]-r[i,k]) * (log(n[i,k]-r[i,k]) - log(n[i,k]-rhat[i,k])))  }  resdev[i] <- sum(dev[i,1:na[i]]) # summed residual deviance contribution for this trial  }  totresdev <- sum(resdev[]) #Total Residual Deviance  d[1]<- 0 # treatment effect is zero for reference treatment  for (k in 2:nt) { d[k] ~ dnorm(0,.0001) } # vague priors for treatment effects  # pairwise ORs and LORs for all possible pair-wise comparisons  for (c in 1:(nt-1)) { for (k in (c+1):nt) {  or[c,k] <- exp(d[k] - d[c])  lor[c,k] <- (d[k]-d[c])  }  }  # ranking  for (k in 1:nt) {  rk[k] <- nt+1-rank(d[],k) # assumes events are “good”  best[k] <- equals(rk[k],1) #calculate probability that treat k is best  }  } # *** PROGRAM ENDS  **Initial values for each chain**  # chain 1  list(d=c(NA,0,0,0,0, 0,0,0,0,0, 0,0,0,0,0, 0),  mu=c(0,0,0,0,0, 0,0,0,0,0, 0,0,0,0,0, 0,0,0,0,0, 0,0,0,0,0, 0,0,0,0,0, 0,0,0,0))  # chain 2  list(d=c(NA,0.1,-1,-0.2,1, 0.1,1,-0.5,-1,0.4, -1,0.5,-0.6,0.7,0.6, -0.3),  mu=c(1,-1,-2,0,0, -2,1,0,2,1, 0.1,1,-0.5,-1,0.4, -1,0.5,-0.6,0.7,0.6, -0.3,0.5,-0.8,1,-0.3, -1,-1,0.7,-0.3,0.8, 0.7,-0.6,0.9,-0.3)) |
