# Supplementary material for: Cost-effectiveness of psychological treatments for post-traumatic stress disorder in adults
Source: PLoS One. 2020 Apr 30;15(4):e0232245. doi: 10.1371/journal.pone.0232245 (PMC7192458; doi:10.1371/journal.pone.0232245)
Supplement: S4 Appendix — (DOCX) [file pone.0232245.s011.docx]

# **Appendix 4. Details of the inconsistency checks and WinBUGS codes for inconsistency models**

## Details of the inconsistency checks

The assumption of consistency between direct and indirect evidence was explored by comparing the fit of a ‘base-case’ model (fixed or random effects) that assumes consistency with a model which allowed for inconsistency between direct an indirect evidence (also known as an unrelated mean effects model (Dias *et al.* 2010; Dias *et al.* 2013b). The latter is equivalent to having separate, unrelated meta-analyses for every pair-wise contrast while assuming a common between-study variance parameter across all comparisons in the case of random effects models. Improvement in model fit or a substantial reduction in heterogeneity in the inconsistency model compared with the NMA consistency model indicates evidence of inconsistency. Inconsistency can only be assessed when there are closed loops of direct evidence on 3 treatments that are informed by at least 3 distinct trials (van Valkenhoef *et al.* 2016). Deviance plots, in which the posterior mean deviance of the individual data points in the inconsistency model were plotted against their posterior mean deviance in the consistency model, were inspected in order to identify studies which may have contributed to loops of evidence where inconsistency may be present. Further checks were conducted using a node-split approach implemented in R using the *gemtc* package in R. This method permits the direct and indirect evidence contributing to an estimate of a relative effect to be split and compared (Dias *et al.* 2013b; van Valkenhoef and Kuiper, 2016).

To apply the node splitting method to the two continuous outcomes (‘changes in PTSD symptom scores between baseline and treatment endpoint’ and ‘changes in PTSD symptom scores between baseline and 1-4 month follow-up’) using the *gemtc* package, data were inputted at contrast level, where the SMDs of the treatment in arm *k* compared to the treatment in arm 1 for study *i* were calculated as

$SMD_{ik}=\frac{\overline{x}_{ik}-\overline{x}_{i1}}{SD_{pooled_{i}}}$, $SD_{pooled_{i}}=\left\{\begin{matrix} \sqrt{\frac{(n_{i1}-1)sd_{i1}^{2}+(n_{i2}-1)sd_{i2}^{2}}{n_{i1}+n_{i2}-2}} & \text{2-arm trial} \\ \sqrt{\frac{(n_{i1}-1)sd_{i1}^{2}+(n_{i2}-1)sd_{i2}^{2}+(n_{i3}-1)sd_{i3}^{2}}{n_{i1}+n_{i2}+n_{i3}-3}} & \text{3-arm trial} \\ \sqrt{\frac{(n_{i1}-1)sd_{i1}^{2}+(n_{i2}-1)sd_{i2}^{2}+(n_{i3}-1)sd_{i3}^{2}+(n_{i4}-1)sd_{i4}^{2}}{n_{i1}+n_{i2}+n_{i3}+n_{i4}-4}} & \text{4-arm trial} \end{matrix}\right.$

with standard error

$$SE\left(SMD_{ik}\right)=\sqrt{Var\left(SMD_{ik}\right)}\approx\left\{\begin{matrix} \sqrt{\frac{1}{n_{i1}}+\frac{1}{n_{i2}}+\frac{SMD_{ik}^{2}}{2\left(n_{i1}+n_{i2}-2\right)}} & \text{2-arm trial} \\ \sqrt{\frac{1}{n_{i1}}+\frac{1}{n_{ik}}+\frac{SMD_{ik}^{2}}{2\left(n_{i1}+n_{i2}+n_{i3}-3\right)}} & \text{3-arm trial} \\ \sqrt{\frac{1}{n_{i1}}+\frac{1}{n_{ik}}+\frac{SMD_{ik}^{2}}{2\left(n_{i1}+n_{i2}+n_{i3}+n_{i4}-4\right)}} & \text{4-arm trial} \end{matrix}\right.$$

For trials with more than two arms, the *gemtc* package requires specification of the standard error of the mean of the baseline arm, as this determines the covariance of the differences. On a standardized scale, this is calculated as (Dias *et al.* 2018):

$$se_{i1_{\text{standardized}}}=\frac{sd_{i1}}{SD_{pooled_{i}}\sqrt{n_{i1}}}$$

To apply the node splitting method to the binary outcome (‘remission status at treatment endpoint’) using the *gemtc* package, data were inputted at arm-level. However, in the node-split model for the non-TF-CBT versuss waitlist comparison, results were unstable. Consequently, we ran the node-split model for this comparison with data inputted at contrast level so that 0.5 could be added to zero cells to stabilise results. The LORs of the treatment in arm *k* relative to the treatment in arm 1 for study *i* were calculated as

$$\ln\left(OR_{ik}\right)=\left\{\begin{matrix} \ln\left(\frac{a_{ik}d_{i1}}{b_{ik}c_{i1}}\right) & \text{if }a_{ik},b_{ik},c_{i1}\text{, and }d_{i1}\text{ are all non-zero} \\ \ln\left(\frac{\left(a_{ik}+0.5\right)\left(d_{i1}+0.5\right)}{\left(b_{ik}+0.5\right)\left(c_{i1}+0.5\right)}\right) & \text{if }a_{ik},b_{ik},c_{i1}\text{, or }d_{i1}\text{ are zero} \end{matrix}\right.$$

with standard error

$$se\left(\ln\left(OR_{ik}\right)\right)=\left\{\begin{matrix} \sqrt{\frac{1}{a_{ik}}+\frac{1}{b_{ik}}+\frac{1}{c_{i1}}+\frac{1}{d_{i1}}} & \text{if }a_{ik},b_{ik},c_{i1}\text{, and }d_{i1}\text{ are all non-zero } \\ \sqrt{\frac{1}{a_{ik}+0.5}+\frac{1}{b_{ik}+0.5}+\frac{1}{c_{i1}+0.5}+\frac{1}{d_{i1}+0.5}} & \text{if }a_{i1},b_{i1},c_{i1}\text{, or }d_{i1}\text{ are zero} \end{matrix}\right.$$

where *a_ik_* and *b_ik_* are the numbers of patients who received the treatment in arm k and achieved and did not achieve remission at treatment endpoint, respectively, and *c_i1_* and *d_i1_* are the numbers of patients who received the treatment in arm 1 and achieved and did not achieve remission at treatment endpoint, respectively. For trials with more than two arms, the standard error of the log odds of the baseline arm was calculated as


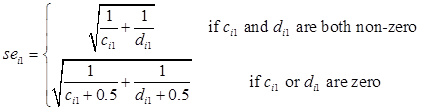


## WinBUGS code for inconsistency random effects models on changes in PTSD symptom scores [Dias *et al.* 2013b]

| **Normal likelihood, identity link: SMD with arm-based means** |
| --- |
| # Normal likelihood, identity link: SMD with arm-based means;  # output as log Odds Ratios  # Random effects model for multi-arm trials  model{ # *** PROGRAM STARTS  for(i in 1:ns){ # LOOP THROUGH STUDIES  delta[i,1] <- 0 # treatment effect is zero for control arm  mu[i] ~ dnorm(0,.0001) # vague priors for all trial baselines  }  # CONTINUOUS DATA AS ARM MEANS  for(i in 1:ns){  # calculate pooled.sd and adjustment for SMD  df[i] <- sum(n[i,1:na[i]]) - na[i] # denominator for pooled.var  Pooled.var[i] <- sum(nvar[i,1:na[i]])/df[i]  Pooled.sd[i] <- sqrt(Pooled.var[i]) # pooled sd for study i, for SMD  # H[i] <- 1 - 3/(4*df[i]-1) # use Hedges' g  H[i] <- 1 # use Cohen's d (ie no adjustment)  for (k in 1:na[i]){  se[i,k] <- sd[i,k]/sqrt(n[i,k])  var[i,k] <- pow(se[i,k],2) # calculate variances  prec[i,k] <- 1/var[i,k] # set precisions  y[i,k] ~ dnorm(phi[i,k], prec[i,k]) # normal likelihood  phi[i,k] <- theta[i,k] * (Pooled.sd[i]/H[i]) # theta is standardised mean  theta[i,k] <- mu[i] + delta[i,k] # model for linear predictor, delta is SMD  dev[i,k] <- (y[i,k]-phi[i,k])*(y[i,k]-phi[i,k])*prec[i,k]  nvar[i,k] <- (n[i,k]-1) * pow(sd[i,k],2) # for pooled.sd  }  # summed residual deviance contribution for this trial  resdev[i] <- sum(dev[i,1:na[i]])  }  # RE MODEL  for(i in 1:ns){ # LOOP THROUGH ALL STUDIES  for (k in 2:na[i]){ # LOOP THROUGH ARMS  # trial-specific RE distributions  delta[i,k] ~ dnorm(d[t[i,1],t[i,k]], tau)  }  }  #  totresdev <- sum(resdev[]) # Total Residual Deviance (all data)  # Priors distributions  sdev ~ dunif(0,5) # vague prior for between-trial SD  tau <- pow(sdev,-2) # between-trial precision  for (c in 1:(nt-1)){  for (k in (c+1):nt){  d[c,k] ~ dnorm(0,.0001) # priors for all mean trt effects  }  }  } # *** PROGRAM ENDS |

## WinBUGS code for inconsistency random effects model on dichotomous remission [Dias *et al.* 2013b]

| **Binomial likelihood, logit link** |
| --- |
| # Binomial likelihood, logit link  # Random effect model, multi-arm trials  model{ # *** PROGRAM STARTS  for(i in 1:ns){ # LOOP THROUGH STUDIES  delta[i,1] <- 0 # treatment effect is zero for control arm  mu[i] ~ dnorm(0,.0001) # vague priors for all trial baselines  for (k in 1:na[i]) { # LOOP THROUGH ARMS  r[i,k] ~ dbin(p[i,k],n[i,k]) # binomial likelihood  logit(p[i,k]) <- mu[i] + delta[i,k] # model for linear predictor  rhat[i,k] <- p[i,k] * n[i,k] # expected value of the numerators  dev[i,k] <- 2 * (r[i,k] * (log(r[i,k])-log(rhat[i,k])) #Deviance contribution  + (n[i,k]-r[i,k]) * (log(n[i,k]-r[i,k]) - log(n[i,k]-rhat[i,k])))  }  resdev[i] <- sum(dev[i,1:na[i]]) # summed residual deviance contribution for this trial  for (k in 2:na[i]) { # LOOP THROUGH ARMS  delta[i,k] ~ dnorm(d[t[i,1],t[i,k]],tau) # trial-specific LOR distributions  }  }  totresdev <- sum(resdev[]) # Total Residual Deviance  sd ~ dunif(0,5)  tau <- pow(sd,-2)  # pairwise LORs for all possible pair-wise comparisons  for (c in 1:(nt-1)){  for (k in (c+1):nt){  d[c,k] ~ dnorm(0,.0001) # priors for all mean trt effects  }  }  } # *** PROGRAM ENDS |
