# Supplementary material for: Cost-effectiveness of psychological treatments for post-traumatic stress disorder in adults
Source: PLoS One. 2020 Apr 30;15(4):e0232245. doi: 10.1371/journal.pone.0232245 (PMC7192458; doi:10.1371/journal.pone.0232245)
Supplement: S5 Appendix — (DOCX) [file pone.0232245.s012.docx]

# **Appendix 5: Characteristics of studies included in the network meta-analysis, and full references**

## Trauma-focused CBT

| Trauma-focused CBT | | | | | | | |
| --- | --- | --- | --- | --- | --- | --- | --- |
|  | Study ID | NMA node: intervention | PTSD details | Trauma type | N | Demographics | Reference |
| 1 | Alghamdi 2015 | Trauma-focused CBT: Narrative exposure therapy (NET)  Waitlist | PTSD diagnosis according to ICD/DSM criteria (including self-report of diagnosis) | Being an emergency responder in a traumatic event (Firefighters exposed to traumatic events: 9% for one time, 18% for 2-3 times and 74%for over 3 times) | 34 | Age range (mean): 22-41 (30.4)  Gender (% female): 0 BME (% non-white): NR Country: Japan Coexisting conditions: NR  Lifetime experience of trauma (mean number of prior traumas/% with previous trauma): NR  Single or multiple incident index trauma: Multiple  ITT or completer continuous data: ITT | Alghamdi M, Hunt N and Thomas S (2015) The effectiveness of Narrative Exposure Therapy with traumatised firefighters in Saudi Arabia: A randomized controlled study. Behaviour Research and Therapy 66, 64-71 |
| 2 | Blanchard 2002/2003/2004 | Trauma-focused CBT: CBT individual  Counselling: Supportive counselling  Waitlist | PTSD diagnosis according to ICD/DSM criteria (including self-report of diagnosis) | Motor Vehicle Collisions (Not reported in details) | 98 | Age range (mean): NR (39.7)  Gender (% female): 73 BME (% non-white): 10 Country: US Coexisting conditions: 49% major depressive disorder (MDD); 35% generalized anxiety disorder (GAD)  Lifetime experience of trauma (mean number of prior traumas/% with previous trauma): NR  Single or multiple incident index trauma: Single  ITT or completer continuous data: completer | Blanchard EB (2002) Treatment-related changes in cardiovascular reactivity to trauma cues in motor vehicle accident-related PTSD. Behaviour Therapy 33, 417-426  Blanchard EB, Hickling EJ, Devineni T, et al. (2003) A controlled evaluation of cognitive behaviorial therapy for posttraumatic stress in motor vehicle accident survivors. Behaviour Research & Therapy 41, 79-96  Blanchard EB, Hickling EJ, Malta LS, et al. (2004) One-and two-year prospective follow-up of cognitive behavior therapy or supportive psychotherapy. Behaviour research and therapy 42(7), 745-59 |
| 3 | Bolton 2014a | Trauma-focused CBT: Cognitive processing therapy  Waitlist | Clinically important PTSD symptoms (scoring above a threshold on validated scale) | Witnessing war as a civilian- ‘Survivor of systematic violence’ (defined as experiencing and/or witnessing physical torture [44% experienced personally; 45% witnessed], imprisonment where torture and other abuse were frequent [58% experienced personally; 52% witnessed], gas attacks [16% experienced personally; 15% witnessed] and/or other military attacks [71% experienced personally; 60% witnessed]) | 167 | Age range (mean): NR (41.8)  Gender (% female): 59 BME (% non-white): NR Country: Iraq Coexisting conditions: Significant depression symptomatology was an inclusion criterion  Lifetime experience of trauma (mean number of prior traumas/% with previous trauma): NR  Single or multiple incident index trauma: Multiple  ITT or completer continuous data: ITT | Bolton P, Bass JK, Zangana GA, et al. (2014) A randomized controlled trial of mental health interventions for survivors of systematic violence in Kurdistan, Northern Iraq. BMC psychiatry 14(1), 360 |
| 4 | Bryant 2003a | Trauma-focused CBT: Exposure therapy/prolonged exposure (PE)  Counselling: Supportive counselling | PTSD diagnosis according to ICD/DSM criteria (including self-report of diagnosis) | Exposure to non-sexual violence - Non-sexual assault (53%); motor vehicle accident (47%) | 58 | Age range (mean): NR (35.2)  Gender (% female): 52 BME (% non-white): NR  Country:  Coexisting conditions: NR  Lifetime experience of trauma (mean number of prior traumas/% with previous trauma): NR  Single or multiple incident index trauma: Single  ITT or completer continuous data: ITT | Bryant RA, Moulds ML, Guthrie RM, et al. (2003) Imaginal exposure alone and imaginal exposure with cognitive restructuring in treatment of posttraumatic stress disorder.Journal of Consulting and Clinical Psychology 71(4), 706-712 |
| 5 | Buhmann 2016 | Trauma-focused CBT: Cognitive therapy  Trauma-focused CBT + SSRI: Cognitive therapy + sertraline  SSRI: sertraline  Waitlist | PTSD diagnosis according to ICD/DSM criteria (including self-report of diagnosis) | Mixed - 43% torture; 28% refugee camp; 63% Danish asylum centre; 24% ex-combatant | 280 | Age range (mean): NR (45)  Gender (% female): 41 BME (% non-white): NR Country: Denmark Coexisting conditions: Patients were not excluded solely based on psychotic symptoms (9% psychotic during treatment). 94% depression according to ICD-10. 27% Personality change after catastrophic events (ICD-10 code F62.0). 25% report traumatic brain injury  Lifetime experience of trauma (mean number of prior traumas/% with previous trauma): NR  Single or multiple incident index trauma: Multiple  ITT or completer continuous data: modified ITT | Buhmann CB, Nordentoft M, Ekstroem M, et al. (2016) The effect of flexible cognitive–behavioural therapy and medical treatment, including antidepressants on post-traumatic stress disorder and depression in traumatised refugees: pragmatic randomised controlled clinical trial. The British Journal of Psychiatry 208(3), 252-9 |
| 6 | Capezzani 2013 | Trauma-focused CBT: CBT individual  EMDR: EMDR | PTSD diagnosis according to ICD/DSM criteria (including self-report of diagnosis) | Diagnosis of life-threatening condition - Participants in follow-up treatment for cancer (breast, colon, uterus, thyroid, melanoma, lung, stomach) | 21 | Age range (mean): NR (51.7)  Gender (% female): 90 BME (% non-white): NR Country: Italy Coexisting conditions: NR  Lifetime experience of trauma (mean number of prior traumas/% with previous trauma): NR  Single or multiple incident index trauma: Single  ITT or completer continuous data: ITT | Capezzani L, Ostacoli L, Cavallo M, et al. (2013) EMDR and CBT for cancer patients: Comparative study of effects on PTSD, anxiety, and depression. Journal of EMDR Practice and Research 7(3), 134-43 |
| 7 | Castillo 2016 | Trauma-focused CBT: Imaginal exposure  Counselling: Supportive counselling | PTSD diagnosis according to ICD/DSM criteria (including self-report of diagnosis) | Military combat - OEF (Afghanistan)/OIF (Iraq) service members (served active duty after September 11th 2001) | 86 | Age range (mean): NR (35.9)  Gender (% female): 100 BME (% non-white): 69 Country: US Coexisting conditions: 62% mood disorder; 60% anxiety disorder; 3% substance use/abuse  Lifetime experience of trauma (mean number of prior traumas/% with previous trauma): 70% 8–17 trauma types; 66% ≥25 trauma incidents  Single or multiple incident index trauma: Multiple  ITT or completer continuous data: modified ITT | Castillo DT, Chee CL, Nason E, et al. (2016) Group-delivered cognitive/exposure therapy for PTSD in women veterans: A randomized controlled trial. Psychological trauma: theory, research, practice, and policy 8(3), 404 |
| 8 | Chambers 2014 | Trauma-focused CBT: CBT individual  Psychoeducation: single psychoeducational phonecall | Clinically important PTSD symptoms (scoring above a threshold on validated scale) | Unintentional injury/illness/medical emergency - Caregivers of patients with cancer (breast (31%), colorectal (9%), prostate (9%), hematologic (8%), lung (8%), and gynaecologic (7%)) | 690 | Age range (mean): NR (52.5)  Gender (% female): 88 BME (% non-white): NR Country: Australia Coexisting conditions: NR  Lifetime experience of trauma (mean number of prior traumas/% with previous trauma): NR  Single or multiple incident index trauma: Single  ITT or completer continuous data: completer | Chambers SK, Girgis A, Occhipinti S, et al. (2014) A randomized trial comparing two low-intensity psychological interventions for distressed patients with cancer and their caregivers. InOncology nursing forum 41(4), p.E257 |
| 9 | Chard 2005 | Trauma-focused CBT: Cognitive processing therapy  Waitlist | PTSD diagnosis according to ICD/DSM criteria (including self-report of diagnosis) | Childhood sexual abuse - Average age at onset of abuse was 6.4 years (SD=2.78); 21% indicated 1-5 incidents of abuse, 12% reported 6-10 incidents, and 10% reported 11-30 incidents; 57% reported >100 abuse incidents | 71 | Age range (mean): 18-56 (32.8)  Gender (% female): 100 BME (% non-white): 19 Country: US Coexisting conditions:  Lifetime experience of trauma (mean number of prior traumas/% with previous trauma): 62% mood disorder; 60% anxiety disorder; 3% substance use/abuse  Single or multiple incident index trauma: Multiple  ITT or completer continuous data: completer | Chard KM (2005) An evaluation of cognitive processing therapy for the treatment of posttraumatic stress disorder related to childhood sexual abuse. Journal of consulting and clinical psychology 73(5), 965 |
| 10 | Cloitre 2002 | Trauma-focused CBT: Exposure therapy/prolonged exposure (PE)  Waitlist | PTSD diagnosis according to ICD/DSM criteria (including self-report of diagnosis) | Childhood sexual abuse - 48% had experienced both sexual and physical abuse, 39% had experienced sexual abuse only, and 13% had experienced physical abuse only | 58 | Age range (mean): NR (34)  Gender (% female): 100 BME (% non-white): 54  Country: US Coexisting conditions: 45% current major depression; 79% anxiety disorder (generalized anxiety disorder [GAD] the most common [48%])  Lifetime experience of trauma (mean number of prior traumas/% with previous trauma): NR  Single or multiple incident index trauma: Multiple  ITT or completer continuous data: completer | Cloitre M, Koenen KC, Cohen LR and Han H (2002) Skills training in affective and interpersonal regulation followed by exposure: a phase-based treatment for PTSD related to childhood abuse. Journal of consulting and clinical psychology 70(5), 1067 |
| 11 | Cloitre 2010 | Trauma-focused CBT: Exposure therapy/prolonged exposure (PE)  Counselling: Supportive counselling | PTSD diagnosis according to ICD/DSM criteria (including self-report of diagnosis) | Childhood sexual abuse - Childhood sexual abuse (90%), childhood physical abuse (79%), emotional abuse or neglect (82%) | 71 | Age range (mean): NR (35.3)  Gender (% female): 100 BME (% non-white): 63 Country: US Coexisting conditions: Current Axis I comorbidity: 89% ≥1; 62% ≥2; 30% ≥3; 20% ≥4  Lifetime experience of trauma (mean number of prior traumas/% with previous trauma): Mean number of lifetime traumas: 6.57 (SD=1.17). Experience of trauma as an adult: Domestic violence (63%); sexual assault (49%); physical assault (24%); other interpersonal victimization (61%)  Single or multiple incident index trauma: Multiple  ITT or completer continuous data: ITT | Cloitre M, Stovall-McClough KC, Nooner K, et al. (2010) Treatment for PTSD related to childhood abuse: A randomized controlled trial. American journal of psychiatry 167(8), 915-24 |
| 12 | Cottraux 2008 | Trauma-focused CBT: Exposure therapy/prolonged exposure (PE)  Counselling: Supportive counselling | PTSD diagnosis according to ICD/DSM criteria (including self-report of diagnosis) | Mixed - Car accidents (33%); physical assault victims (26%); rape (8%); miscellaneous experiences (8%); family violence (7%); witnessed extreme violence (7%); incest (5%); witnessed the death of a close relative (3%); painful and complicated surgery (2%) | 60 | Age range (mean): NR (39)  Gender (% female): 70 BME (% non-white): NR Country: France Coexisting conditions: NR  Lifetime experience of trauma (mean number of prior traumas/% with previous trauma): Mean number of traumatic episodes: 1.78 (0.9)  Single or multiple incident index trauma: Single  ITT or completer continuous data: completer | Cottraux J, Note I, Yao SN, et al. (2008) Randomized controlled comparison of cognitive behavior therapy with Rogerian supportive therapy in chronic post-traumatic stress disorder: A 2-year follow-up. Psychotherapy and psychosomatics 77(2), 101-10 |
| 13 | Difede 2007b | Trauma-focused CBT: Exposure therapy/prolonged exposure (PE)  Waitlist | Clinically important PTSD symptoms (scoring above a threshold on validated scale) | Terrorist attacks - Disaster workers exposed to the World Trade Centre attack and/or its aftermath | 31 | Age range (mean): NR (45.77)  Gender (% female): 3 BME (% non-white): 23 Country: US Coexisting conditions: NR  Lifetime experience of trauma (mean number of prior traumas/% with previous trauma): 67% had trauma history  Single or multiple incident index trauma: Single  ITT or completer continuous data: ITT | Difede J, Malta LS, Best S, et al. (2007) A randomized controlled clinical treatment trial for World Trade Center attack-related PTSD in disaster workers. The Journal of nervous and mental disease 195(10), 861-5 |
| 14 | Dunne 2012 | Trauma-focused CBT: CBT individual  Waitlist | PTSD diagnosis according to ICD/DSM criteria (including self-report of diagnosis) | Motor Vehicle Collisions (Participants were diagnosed with chronic Whiplash-associated disorders, grade II or III) | 26 | Age range (mean): 20-49 (32.5)  Gender (% female): 50 BME (% non-white): 27 Country: Australia Coexisting conditions: 54% met the DSM-IV criteria for comorbid depression and 31% met the criteria for current alcohol use disorder  Lifetime experience of trauma (mean number of prior traumas/% with previous trauma): NR  Single or multiple incident index trauma: Single  ITT or completer continuous data: completer | Dunne RL, Kenardy J and Sterling M (2012) A randomized controlled trial of cognitive-behavioral therapy for the treatment of PTSD in the context of chronic whiplash. The Clinical journal of pain 28(9), 755-65 |
| 15 | Echiverri-Cohen 2016 | Trauma-focused CBT: Exposure therapy/prolonged exposure (PE)  SSRI: sertraline | PTSD diagnosis according to ICD/DSM criteria (including self-report of diagnosis) | Mixed - Sexual assault (31%); physical assault (27%); child sexual assault (22%); child physical assault (8%); motor vehicle accident (6%); natural disaster (4%); death of loved one (2%) | 49 | Age range (mean): NR (37.7)  Gender (% female): 75 BME (% non-white): 33 Country: US Coexisting conditions: NR  Lifetime experience of trauma (mean number of prior traumas/% with previous trauma): NR  Single or multiple incident index trauma: Unclear  ITT or completer continuous data: ITT | Echiverri-Cohen A, Zoellner LA, Gallop R, et al. (2016) Changes in temporal attention inhibition following prolonged exposure and sertraline in the treatment of PTSD. Journal of consulting and clinical psychology 84(5), 415 |
| 16 | Ehlers 2003 | Trauma-focused CBT: Cognitive therapy  Self-help (without support): Cognitive bibliotherapy  Waitlist | PTSD diagnosis according to ICD/DSM criteria (including self-report of diagnosis) | Motor Vehicle Collisions (Involvement in a MVC that required A & E attendance) | 85 | Age range (mean): 18-65 (NR)  Gender (% female): NR BME (% non-white): NR Country: UK Coexisting conditions: NR  Lifetime experience of trauma (mean number of prior traumas/% with previous trauma): NR  Single or multiple incident index trauma: Single  ITT or completer continuous data: NA (only dichotomous data used) | Ehlers A, Clark DM, Hackmann A, et al. (2003) A randomized controlled trial of cognitive therapy, a self-help booklet, and repeated assessments as early interventions for posttraumatic stress disorder. Arch.Gen.Psychiatry 60(10), 1024-1032 |
| 17 | Ehlers 2005 | Trauma-focused CBT: Cognitive therapy  Waitlist | PTSD diagnosis according to ICD/DSM criteria (including self-report of diagnosis) | Mixed - Accident (54%), assault (32%), witnessing death (14%) | 28 | Age range (mean): NR (36.6)  Gender (% female): 54  BME (% non-white): 4 Country: UK Coexisting conditions: 39% current major depression; 21% comorbid anxiety disorders  Lifetime experience of trauma (mean number of prior traumas/% with previous trauma): Half of the participants reported an earlier trauma meeting the A criterion of DSM-IV (but these events were not addressed in treatment)  Single or multiple incident index trauma: Single  ITT or completer continuous data: ITT | Ehlers A, Clark DM, Hackmann A, et al. (2005) Cognitive therapy for post-traumatic stress disorder: development and evaluation. Behaviour research and therapy 43(4), 413-31 |
| 18 | Ehlers 2014 | Trauma-focused CBT: Cognitive therapy  Counselling: supportive counselling  Waitlist | PTSD diagnosis according to ICD/DSM criteria (including self-report of diagnosis) | Mixed - Interpersonal violence (36%); Accidents/disaster (38%); Death/harm to others (8%); Other (18%) | 91 | Age range (mean): NR (38.7)  Gender (% female): 58 BME (% non-white): 31 Country: UK Coexisting conditions: Depressive disorder (35%); anxiety disorder (30%); substance abuse (15%); Axis II disorder (19%)  Lifetime experience of trauma (mean number of prior traumas/% with previous trauma): 70% history of other trauma; 10% reported history of childhood abuse  Single or multiple incident index trauma: Unclear  ITT or completer continuous data: ITT | Ehlers A, Hackmann A, Grey N, et al. (2014) A randomized controlled trial of 7-day intensive and standard weekly cognitive therapy for PTSD and emotion-focused supportive therapy. American Journal of Psychiatry 171(3), 294-304 |
| 19 | Falsetti 2008 | Trauma-focused CBT: Exposure therapy/prolonged exposure (PE)  Waitlist | PTSD diagnosis according to ICD/DSM criteria (including self-report of diagnosis) | Mixed - A mean of 6 traumatic events reported (SD=2.03, range=2–10). The most frequently reported traumatic events included unwanted or forced sexual contact (76%), physical assault without a weapon (71%), unwanted sexual contact before age 18 (69%), natural disaster (65%), and physical assault with a weapon (58%). Physical injury during a traumatic event was reported by 97% of the participants. | 60 | Age range (mean): NR (35)  Gender (% female): 100 BME (% non-white): 31 Country: US Coexisting conditions: 100% panic attacks (inclusion criterion). 89% met DSM-IV criteria for panic disorder (based on ADIS-R)  Lifetime experience of trauma (mean number of prior traumas/% with previous trauma): NR trauma: Multiple  ITT or completer continuous data: modified ITT | Falsetti SA, Resnick HS and Davis JL (2008) Multiple channel exposure therapy for women with PTSD and comorbid panic attacks. Cognitive Behaviour Therapy 37(2), 117-30 |
| 20 | Fecteau 1999 | Trauma-focused CBT: Brief individual CBT  Waitlist | PTSD diagnosis according to ICD/DSM criteria (including self-report of diagnosis) | Motor Vehicle Collisions (Motor vehicle accidents resulting in physical injury) | 24 | Age range (mean): 25-63 (41.3)  Gender (% female): 70 BME (% non-white): NR  Country: Canada Coexisting conditions: 85% had ongoing pain and physical complaints from their MVC  Lifetime experience of trauma (mean number of prior traumas/% with previous trauma): NR  Single or multiple incident index trauma: Single  ITT or completer continuous data: completer | Fecteau G and Nicki R (1999) Cognitive behavioural treatment of post traumatic stress disorder after motor vehicle accident. Behavioural & Cognitive Psychotherapy 27, 201-214 |
| 21 | Foa 1991 | Trauma-focused CBT: Exposure therapy/prolonged exposure (PE)  Non-trauma-focused CBT: Stress inoculation training (SIT)  Counselling: Supportive counselling | PTSD diagnosis according to ICD/DSM criteria (including self-report of diagnosis) | Exposure to sexual abuse or assault (Rape or attempted rape. 54% perpetrator was a stranger; 46% perpetrator was an acquaintance. 60% weapon used) | 55 | Age range (mean): NR (31.8)  Gender (% female): 100 BME (% non-white): 26 Country: US Coexisting conditions: NR  Lifetime experience of trauma (mean number of prior traumas/% with previous trauma): NR  Single or multiple incident index trauma: Single  ITT or completer continuous data: completer | Foa EB, Rothbaum BO, Riggs DS and Murdock TB (1991) Treatment of posttraumatic stress disorder in rape victims: a comparison between cognitive-behavioral procedures and counseling. Journal of Consulting & Clinical Psychology 59, 715-723 |
| 22 | Gersons 2000 | Trauma-focused CBT: Brief eclectic psychotherapy  Waitlist | PTSD diagnosis according to ICD/DSM criteria (including self-report of diagnosis) | Being an emergency responder in a traumatic event - Police officers exposed to trauma in the course of their work. Mean number of traumas in police work 17.1 (SD=8.2) | 42 | Age range (mean): NR (36.4)  Gender (% female): 12 BME (% non-white): 0 Country: Netherlands Coexisting conditions: 86% any other comorbid psychiatric disorder (DSM-III-R): 40% Major Depression; 12% Dysthymia; 26% Alcohol Dependence; 10% Generalized Anxiety; 9% Agoraphobia; 7% Social Phobia; 7% Phobic Disorder; 7% OCD; 5% Panic Disorder  Lifetime experience of trauma (mean number of prior traumas/% with previous trauma): Mean number of traumas outside police work 3.5 (SD=2.5)  Single or multiple incident index trauma: Multiple  ITT or completer continuous data: NA (only dichotomous data used) | Gersons BP, Carlier IV, Lamberts RD and Van der Kolk BA (2000) Randomized clinical trial of brief eclectic psychotherapy for police officers with posttraumatic stress disorder. Journal of Traumatic Stress 13, 333-347 |
| 23 | Ghafoori 2017 | Trauma-focused CBT: Exposure therapy/prolonged exposure (PE)  Present-centered therapy: Present-centered therapy | PTSD diagnosis according to ICD/DSM criteria (including self-report of diagnosis) | Mixed - Experienced or witnessed a lifetime traumatic event that involved actual or threatened death, serious injury or threat to the physical integrity of others | 71 | Age range (mean): 18-71 (35.2)  Gender (% female): 83 BME (% non-white): 72  Country: US Coexisting conditions: NR  Lifetime experience of trauma (mean number of prior traumas/% with previous trauma):  Single or multiple incident index trauma: Mean number of traumas experienced 6.49 (SD=3.45). Traumas reported: Natural disaster (47%); fire or explosion (28%); transportation accident (59%); serious accident at work, home or during a recreational activity (38%); exposure to toxic substance (11%); physical assault (82%); assault with a weapon (52%); sexual assault (49%); other unwanted or uncomfortable sexual experience (61%); combat (9%); captivity (25%); life threatening illness or injury (44%); severe human suffering (28%); sudden violent death (32%); sudden accidental death (18%); serious injury, harm or death you caused to someone else (10%); any other stressful event or experience (56%)  Single or multiple incident index trauma: Single  ITT or completer continuous data: ITT | Ghafoori B, Hansen MC, Garibay E and Korosteleva O (2017) Feasibility of training frontline therapists in prolonged exposure: a randomized controlled pilot study of treatment of complex trauma in diverse victims of crime and violence. The Journal of nervous and mental disease 205(4), 283-93 |
| 24 | Hensel-Dittmann 2011 | Trauma-focused CBT: Narrative exposure therapy (NET)  Non-trauma-focused CBT: Stress inoculation training (SIT) | PTSD diagnosis according to ICD/DSM criteria (including self-report of diagnosis) | Witnessing war as a civilian - 93% asylum seekers who had fled from their countries of origin after experiencing organized violence. 76% reported experiences of torture and >70% had been in detention | 28 | Age range (mean): NR (NR)  Gender (% female): NR BME (% non-white): NR Country: Germany Coexisting conditions: 82% major depression, 18% dysthymia, 54% anxiety disorder/OCD, 11% substance abuse, and 4% psychotic disorder  Lifetime experience of trauma (mean number of prior traumas/% with previous trauma): NR  Single or multiple incident index trauma: Multiple  ITT or completer continuous data: completer | Hensel-Dittmann D, Schauer M, Ruf M, et al. (2011) The treatment of traumatized victims of war and torture: a randomized controlled comparison of Narrative Exposure Therapy and Stress Inoculation Training. Psychotherapy and Psychosomatics 80, 345-352 [DOI: 10.1159/000327253] |
| 25 | Hijazi 2014 | Trauma-focused CBT: Brief narrative exposure therapy (NET)  Waitlist | Clinically important PTSD symptoms (scoring above a threshold on validated scale) | Witnessing war as a civilian - Iraqi and Syrian refugees: Racial/religious oppression (92%); exposure to combat situation (92%); witnessing murder (68%); murder/violent death of family/friends (65%); kidnapping of family/friends (59%); witnessing torture (41%); physically harmed (38%); imprisoned arbitrarily (29%); witnessing mass execution of civilians (27%); kidnapped (27%); tortured (25%); taken hostage (18%); sexually abused/raped (6%). Most participants experienced multiple events (mean 19.8; SD=6.4) | 63 | Age range (mean): NR (48.2)  Gender (% female): 56 BME (% non-white): NR Country: US Coexisting conditions: NR  Lifetime experience of trauma (mean number of prior traumas/% with previous trauma): NR  Single or multiple incident index trauma: Multiple  ITT or completer continuous data: ITT | Hijazi AM, Lumley MA, Ziadni MS, et al. (2014) Brief Narrative Exposure Therapy for Posttraumatic Stress in Iraqi Refugees: A Preliminary Randomized Clinical Trial. J. Traum. Stress 27, 314–322 [doi: 10.1002/jts.21922] |
| 26 | Hollifield 2007 | Trauma-focused CBT: CBT group  Waitlist | PTSD diagnosis according to ICD/DSM criteria (including self-report of diagnosis) | Unclear - 38% reported experiencing ≥3 events; 33% identified ≥5 years of ongoing childhood abuse | 84 | Age range (mean): NR (42.2)  Gender (% female): 66  BME (% non-white): 36  Country: US  Coexisting conditions: NR  Lifetime experience of trauma (mean number of prior traumas/% with previous trauma): NR  Single or multiple incident index trauma: Unclear  ITT or completer continuous data: modified ITT | Hollifield M, Sinclair-Lian N, Warner TD and Hammerschlag R (2007) Acupuncture for posttraumatic stress disorder: a randomized controlled pilot trial. The Journal of nervous and mental disease 195(6), 504-13 |
| 27 | Jacob 2014 | Trauma-focused CBT: Narrative exposure therapy (NET)  Waitlist | PTSD diagnosis according to ICD/DSM criteria (including self-report of diagnosis) | Witnessing war as a civilian - Widowed or orphaned survivors of Rwandan (1994) genocide. Among the 43 widows, the most frequently reported worst life experiences were sexual abuse (21%), the genocide in general (21%), and witnessing a massacre (14%). Among the 33 orphans, the most frequently reported worst life experiences were sexual abuse (21%), witnessing the killing of a parent (15.2%), and the genocide in general (12%) | 76 | Age range (mean): NR (37.6)  Gender (% female): 84 BME (% non-white): 100 Country: Rwanda Coexisting conditions: NR  Lifetime experience of trauma (mean number of prior traumas/% with previous trauma): Mean number of traumatic event types ever experienced: 14.4 (SD=3.8)  Single or multiple incident index trauma: Multiple  ITT or completer continuous data: ITT | Jacob N, Neuner F, Mädl A, et al. (2014) Dissemination of psychotherapy for trauma-spectrum disorders in resource-poor countries: a randomized controlled trial in Rwanda. Psychotherapy & Psychosomatics 83, 354–363 [DOI:10.1159/000365114] |
| 28 | Jung 2013 | Trauma-focused CBT: Brief individual CBT  Waitlist | PTSD diagnosis according to ICD/DSM criteria (including self-report of diagnosis) | Childhood sexual abuse - Participants had experienced childhood sexual abuse (mean reported age at time of first sexual abuse was 7.7 years [SD=4.3]) and also suffered from a feeling of being contaminated (FBC). The duration of abuse lasted 6.8 years (SD=5.2) on average, and the duration of FBC ranged from 2 to 46 years (mean 20 years). 71.4% of abuse was severe, and included penetration, 71.4% of abuse was inflicted by a relative | 34 | Age range (mean): 19-61 (37.2)  Gender (% female): 100  BME (% non-white): 11 Country: Germany Coexisting conditions: Mean 3.4 (SD=1.06) DSM-IV Axis-I or Axis-II diagnoses: 57% major depressive disorder; 32% eating disorders; 32% borderline personality disorder; 25% social anxiety disorder  Lifetime experience of trauma (mean number of prior traumas/% with previous trauma): NR  Single or multiple incident index trauma: Multiple  ITT or completer continuous data: completer | Jung K and Steil R (2013) A randomized controlled trial on cognitive restructuring and imagery modification to reduce the feeling of being contaminated in adult survivors of childhood sexual abuse suffering from posttraumatic stress disorder. Psychotherapy and psychosomatics 82(4), 213-20 |
| 29 | Katz 2014 | Trauma-focused CBT: Exposure therapy/prolonged exposure (PE)  Counselling: Supportive counselling | Clinically important PTSD symptoms (scoring above a threshold on validated scale) | Exposure to sexual abuse or assault - Female veterans who had a history of sexual trauma, including: military sexual trauma (88%); childhood sexual abuse (71%); adult sexual assault (44%); domestic violence (68%) | 34 | Age range (mean): 22-66 (42)  Gender (% female): 100 BME (% non-white): 56 Country: US Coexisting conditions: NR  Lifetime experience of trauma (mean number of prior traumas/% with previous trauma): NR  Single or multiple incident index trauma: Multiple  ITT or completer continuous data: completer | Katz LS, Douglas S, Zaleski K, et al. (2014) Comparing holographic reprocessing and prolonged exposure for women veterans with sexual trauma: A pilot randomized trial. Journal of Contemporary Psychotherapy 44(1), 9-19 |
| 30 | Lindauer 2005 | Trauma-focused CBT: Brief eclectic psychotherapy  Waitlist | PTSD diagnosis according to ICD/DSM criteria (including self-report of diagnosis) | Mixed (25% robbery/weapon used; 13% assaulted by strangers; 13% threatened with death/serious harm; 13% rape; 4% natural disaster; 4% motor vehicle accident; 21% 'other' kind of accident; 4% combat or warfare; 4% life-threatening/disabling event to a loved one) | 24 | Age range (mean): NR (39)  Gender (% female): 54 BME (% non-white): NR Country: Netherlands Coexisting conditions: 13% had mild major depression (those with moderate or severe depression were excluded)  Lifetime experience of trauma (mean number of prior traumas/% with previous trauma): Mean number of prior traumas 3.7 (SD=3.4)  Single or multiple incident index trauma: Single  ITT or completer continuous data: NA (only dichotomous data used) | Lindauer RJ, Gersons BP, van Meijel EP, et al. (2005) Effects of brief eclectic psychotherapy in patients with posttraumatic stress disorder: Randomized clinical trial. Journal of traumatic stress 18(3), 205-12 |
| 31 | Lindauer 2008 | Trauma-focused CBT: Brief eclectic psychotherapy  Waitlist | PTSD diagnosis according to ICD/DSM criteria (including self-report of diagnosis) | Domestic violence (67% interpersonal violence; 33% accidents or disasters) | 24 | Age range (mean): NR (39.7)  Gender (% female): 50 BME (% non-white): NR Country: Netherlands Coexisting conditions: 15% had mild major depression (those with moderate or severe depression were excluded)  Lifetime experience of trauma (mean number of prior traumas/% with previous trauma): NR  Single or multiple incident index trauma: Multiple  ITT or completer continuous data: completer | Lindauer RJ, Booij J, Habraken JB, et al. (2008) Effects of psychotherapy on regional cerebral blood flow during trauma imagery in patients with post-traumatic stress disorder: a randomized clinical trial. Psychological medicine 38(4), 543-54 |
| 32 | Markowitz 2015a | Trauma-focused CBT: Exposure therapy/prolonged exposure (PE)  Interpersonal psychotherapy (IPT): IPT  Relaxation | PTSD diagnosis according to ICD/DSM criteria (including self-report of diagnosis) | Domestic violence - 93% reported interpersonal trauma (42% acute; 58% chronic) | 110 | Age range (mean): NR (40.1)  Gender (% female): 70  BME (% non-white): 35 Country: US Coexisting conditions: Current major depressive disorder (50%); recurrent major depressive disorder (34%); current generalised anxiety disorder (13%). Any axis II diagnosis (49%): 25% paranoid; 14% narcissistic; 5% borderline; 21% avoidant; 3% dependent; 25% obsessive-compulsive; 25% depressive; 15% passive-aggressive.  Lifetime experience of trauma (mean number of prior traumas/% with previous trauma): Mean number of traumas 2.8 (SD=1.8). 36% reported trauma in childhood or adolescence  Single or multiple incident index trauma: Multiple  ITT or completer continuous data: completer | Markowitz JC, Petkova E, Neria Y, et al. (2015) Is exposure necessary? A randomized clinical trial of interpersonal psychotherapy for PTSD. American Journal of Psychiatry 172(5), 430-40 |
| 33 | McDonagh 2005 | Trauma-focused CBT: Exposure therapy/prolonged exposure (PE)  Present-centered therapy: Present-centered therapy  Waitlist | PTSD diagnosis according to ICD/DSM criteria (including self-report of diagnosis) | Childhood sexual abuse (Childhood sexual abuse characteristics: 23% experienced life threat; 34% injured; 64% penetrated. Perpetrator of worst CSA event: 32% father or stepfather; 35% other male relative; 31% known male; 1% male stranger) | 74 | Age range (mean): NR (40.4)  Gender (% female): 100 BME (% non-white): 7 Country: US Coexisting conditions: 11% met criteria for borderline personality disorder  Lifetime experience of trauma (mean number of prior traumas/% with previous trauma): Mean number of trauma types 3.3 (SD=1.1). Trauma history: 80% childhood physical abuse; 62% adult physical abuse; 50% adult sexual trauma  Single or multiple incident index trauma: Multiple  ITT or completer continuous data: ITT post-treatment; completer at follow-up | McDonagh A, Friedman M, McHugo G, et al. (2005) Randomized trial of cognitive-behavioral therapy for chronic posttraumatic stress disorder in adult female survivors of childhood sexual abuse. Journal of consulting and clinical psychology 73(3), 515 |
| 34 | Neuner 2008 | Trauma-focused CBT: Narrative exposure therapy (NET)  Counselling: Supportive counselling | PTSD diagnosis according to ICD/DSM criteria (including self-report of diagnosis) | Witnessing war as a civilian - Rwandan and Somalian refugees settled in a refugee camp in Uganda | 277 | Age range (mean): NR (35)  Gender (% female): 51 BME (% non-white): NR Country: Uganda Coexisting conditions: NR  Lifetime experience of trauma (mean number of prior traumas/% with previous trauma): Mean number of trauma event types 14.1 (SD=5.2)Single or multiple incident index trauma: Multiple  ITT or completer continuous data: ITT | Neuner F, Onyut PL, Ertl V, et al. (2008) Treatment of posttraumatic stress disorder by trained lay counselors in an African refugee settlement. A randomized controlled trial. J Consult Clin Psychol 76, 686-694 |
| 35 | Pacella 2012 | Trauma-focused CBT: Exposure therapy/prolonged exposure (PE)  Waitlist | Clinically important PTSD symptoms (scoring above a threshold on validated scale) | Mixed (100% were living with HIV and 34% reported that their most distressing trauma was related to their HIV diagnosis. 97% reported experiencing both an HIV-and non-HIV-related trauma) | 66 | Age range (mean): 31-61 (46.4)  Gender (% female): 37 BME (% non-white): 61 Country: US Coexisting conditions: NR  Lifetime experience of trauma (mean number of prior traumas/% with previous trauma): Mean 4.91 (SD=1.78) different types of prior trauma  Single or multiple incident index trauma: Unclear  ITT or completer continuous data: modified ITT | Pacella ML, Armelie A, Boarts J, et al. (2012) The impact of prolonged exposure on PTSD symptoms and associated psychopathology in people living with HIV: A randomized test of concept. AIDS and Behavior 16(5), 1327-40 |
| 36 | Popiel 2015 | Trauma-focused CBT: Exposure therapy/prolonged exposure (PE)  Trauma-focused CBT + SSRI: Exposure therapy/prolonged exposure (PE) + paroxetine  SSRI: paroxetine | PTSD diagnosis according to ICD/DSM criteria (including self-report of diagnosis) | Motor Vehicle Collisions - Status during MVC: Driver (38%); Passenger (30%); Cyclist (5%); Pedestrian (14%); Found out about death (7%); Other (5%). Patient considered MVA perpetrator (11%) | 228 | Age range (mean): NR (37.7)  Gender (% female): NR  BME (% non-white): NR Country: Poland Coexisting conditions: 49% Comorbid Axis I disorder; 41% Comorbid personality disorder; 21% traumatic brain injury in MVA; 39% had no comorbid mental disorders; 48% still had ongoing medical sequelae (including chronic pain) related to the accident  Lifetime experience of trauma (mean number of prior traumas/% with previous trauma): Number of previous traumatic events (before current MVA): 2.1 (sd=1.3). 5% childhood trauma  Single or multiple incident index trauma: Single  ITT or completer continuous data: completer | Popiel A, Zawadzki B, Pragłowska E and Teichman Y (2015) Prolonged exposure, paroxetine and the combination in the treatment of PTSD following a motor vehicle accident. A randomized clinical trial–The “TRAKT” study. Journal of behavior therapy and experimental psychiatry 48, 17-26 |
| 37 | van Emmerik 2008 | Trauma-focused CBT: CBT individual  Self help with support: Structured writing therapy  Waitlist | PTSD diagnosis according to ICD/DSM criteria (including self-report of diagnosis) | Exposure to non-sexual violence - Nonsexual violence (50%); Traffic accident (23%); Sexual violence (11%); Other (16%) | 125 | Age range (mean): NR (40.2)  Gender (% female): 67 BME (% non-white): NR Country: Netherlands Coexisting conditions: NR  Lifetime experience of trauma (mean number of prior traumas/% with previous trauma): NR  Single or multiple incident index trauma: Single  ITT or completer continuous data: ITT | Van Emmerik AA, Kamphuis JH and Emmelkamp PM (2008) Treating acute stress disorder and posttraumatic stress disorder with cognitive behavioral therapy or structured writing therapy: a randomized controlled trial. Psychotherapy and psychosomatics 77(2), 93-100 |
| 38 | Weiss 2015 (study 1) | Trauma-focused CBT: CBT individual  Waitlist | Clinically important PTSD symptoms (scoring above a threshold on validated scale) | Witnessing war as a civilian - Survivors of systematic violence (having experienced or witnessed physical torture or militant attacks) in Southern Iraq | 149 | Age range (mean): NR (42.8)  Gender (% female): 31 BME (% non-white): NR Country: Iraq  Coexisting conditions: NR  Lifetime experience of trauma (mean number of prior traumas/% with previous trauma): NR  Single or multiple incident index trauma: Multiple  ITT or completer continuous data: ITT | Weiss WM, Murray LK, Zangana GA, et al. (2015) Community-based mental health treatments for survivors of torture and militant attacks in Southern Iraq: a randomized control trial. BMC psychiatry 15(1), 249 |
| 39 | Weiss 2015 (study 2) | Trauma-focused CBT: Cognitive processing therapy  Waitlist | Clinically important PTSD symptoms (scoring above a threshold on validated scale) | Witnessing war as a civilian - Survivors of systematic violence (having experienced or witnessed physical torture or militant attacks) in Southern Iraq | 193 | Age range (mean): NR (40.3)  Gender (% female): 34 BME (% non-white): NR  Country: Iraq  Coexisting conditions: NR  Lifetime experience of trauma (mean number of prior traumas/% with previous trauma): NR  Single or multiple incident index trauma: Multiple  ITT or completer continuous data: ITT | Weiss WM, Murray LK, Zangana GA, et al. (2015) Community-based mental health treatments for survivors of torture and militant attacks in Southern Iraq: a randomized control trial. BMC psychiatry 15(1), 249 |
| 40 | Zang 2014 | Trauma-focused CBT: Narrative exposure therapy (NET)  Waitlist | PTSD diagnosis according to ICD/DSM criteria (including self-report of diagnosis) | Natural disasters (such as severe floods, earthquakes or tsunamis) - Sichuan earthquake (2008). 27% injured in earthquake; 100% house damage. All participants reported seeing someone seriously injured and death during the earthquake | 30 | Age range (mean): 28-80 (53.6)  Gender (% female): 90 BME (% non-white): NR Country: China Coexisting conditions:  Lifetime experience of trauma (mean number of prior traumas/% with previous trauma): 20% prior trauma (7% 1 prior trauma; 13% 2-3)  Single or multiple incident index trauma: Single  ITT or completer continuous data: ITT | Zang Y, Hunt N and Cox T (2014) Adapting narrative exposure therapy for Chinese earthquake survivors: A pilot randomised controlled feasibility study. BMC psychiatry 14(1), 1.v |

## Trauma-focused CBT + SSRI

| Trauma-focused CBT + SSRI | | | | | | | |
| --- | --- | --- | --- | --- | --- | --- | --- |
|  | Study ID | NMA node: intervention | PTSD details | Trauma type | N | Demographics | Reference |
|  | Buhmann 2016 | Trauma-focused CBT: Cognitive therapy  Trauma-focused CBT + SSRI: Cognitive therapy + sertraline  SSRI: sertraline  Waitlist | **SEE OTHER DETAILS OF THE STUDY UNDER TRAUMA-FOCUSED CBT** | | | | |
|  | Popiel 2015 | Trauma-focused CBT: Exposure therapy/prolonged exposure (PE)  Trauma-focused CBT + SSRI: Exposure therapy/prolonged exposure (PE) + paroxetine  SSRI: paroxetine | **SEE OTHER DETAILS OF THE STUDY UNDER TRAUMA-FOCUSED CBT** | | | | |
| 41 | Rothbaum 2006 | Trauma-focused CBT + SSRI: Exposure therapy/prolonged exposure (PE) + sertraline  SSRI: sertraline | PTSD diagnosis according to ICD/DSM criteria (including self-report of diagnosis) | Mixed - Sexual assault (37%); non-sexual assault (25%); death of another (22%); motor vehicle accident (9%); other (8%) | 65 | Age range (mean): NR (39.3)  Gender (% female): 65 BME (% non-white): 20 Country: US Coexisting conditions: NR  Lifetime experience of trauma (mean number of prior traumas/% with previous trauma): NR  Single or multiple incident index trauma: Single  ITT or completer continuous data: ITT | Rothbaum BO, Cahill SP, Foa EB, et al. (2006) Augmentation of sertraline with prolonged exposure in the treatment of posttraumatic stress disorder. Journal of traumatic stress 19(5), 625-38 |

## EMDR

| EMDR | | | | | | | |
| --- | --- | --- | --- | --- | --- | --- | --- |
|  | Study ID | NMA node: intervention | PTSD details | Trauma type | N | Demographics | Reference |
| 42 | Acarturk 2015 | EMDR: EMDR  Waitlist | Clinically important PTSD symptoms (scoring above a threshold on validated scale) | Witnessing war as a civilian (Syrian refugees) | 29 | Age range (mean): 19-63 (36.6)  Gender (% female): 76 BME (% non-white):  Country: Turkey  Coexisting conditions: NR  Lifetime experience of trauma (mean number of prior traumas/% with previous trauma): NR  Single or multiple incident index trauma: Multiple  ITT or completer continuous data: ITT | Acarturk C, Konuk E, Cetinkaya M et al. (2015) EMDR for Syrian refugees with posttraumatic stress disorder symptoms: Results of a pilot randomized controlled trial. European Journal of Psychotraumatology 6(1), 27414 |
| 43 | Acarturk 2016 | EMDR: EMDR  Waitlist | PTSD diagnosis according to ICD/DSM criteria (including self-report of diagnosis) | Witnessing war as a civilian (Syrian refugees. Traumatic events included: death of family members; threatened death to self or others; serious injury to self or loved ones; husband being at war; arrested family members; not being able to bury significant others who have died in Syria; lack of shelter) | 98 | Age range (mean): 17-64  Gender (% female): 74 BME (% non-white): NR Country:  Coexisting conditions: NR  Lifetime experience of trauma (mean number of prior traumas/% with previous trauma): NR  Single or multiple incident index trauma: Multiple  ITT or completer continuous data: ITT | Acarturk C, Konuk E, Cetinkaya M, et al. (2016) The efficacy of eye movement desensitization and reprocessing for post-traumatic stress disorder and depression among Syrian refugees: Results of a randomized controlled trial. Psychological medicine 46(12), 2583-93 |
| 44 | Aldahadha 2012 | EMDR: EMDR  Waitlist | PTSD diagnosis according to ICD/DSM criteria (including self-report of diagnosis) | Motor Vehicle Collisions (no further details reported) | 51 | Age range (mean): 19-37 (26.4)  Gender (% female): 53 BME (% non-white): NR  Country: Oman Coexisting conditions: NR  Lifetime experience of trauma (mean number of prior traumas/% with previous trauma): NR  Single or multiple incident index trauma: Single  ITT or completer continuous data: ITT | Aldahadha B, Al-Harthy H and Sulaiman S (2012) The efficacy of eye movement desensitization reprocessing in resolving the trauma caused by the road accidents in the Sultanate of Oman. Journal of Instructional Psychology 39(3/4), 146 |
|  | Capezzani 2013 | Trauma-focused CBT: CBT individual  EMDR: EMDR | **SEE OTHER DETAILS OF THE STUDY UNDER TRAUMA-FOCUSED CBT** | | | | |
| 45 | Carletto 2016 | EMDR: EMDR  Relaxation | PTSD diagnosis according to ICD/DSM criteria (including self-report of diagnosis) | Diagnosis of life-threatening condition (multiple sclerosis) | 50 | Age range (mean): NR(40.1)  Gender (% female): 81 BME (% non-white): NR Country: Italy Coexisting conditions: NR  Lifetime experience of trauma (mean number of prior traumas/% with previous trauma): Mean number of previous traumas: 4.3 (6.5)  Single or multiple incident index trauma: Single  ITT or completer continuous data: completer | Carletto S, Borghi M, Bertino G, et al. (2016) Treating post-traumatic stress disorder in patients with multiple sclerosis: a randomized controlled trial comparing the efficacy of eye movement desensitization and reprocessing and relaxation therapy. Frontiers in psychology 7 |
| 46 | Carlson 1998 | EMDR: EMDR  Relaxation | PTSD diagnosis according to ICD/DSM criteria (including self-report of diagnosis) | Military combat (97% Vietnam veterans, 3% other combat theatre) | 35 | Age range (mean): 41-70 (48)  Gender (% female): 0 BME (% non-white): 46 Country: US Coexisting conditions: NR  Lifetime experience of trauma (mean number of prior traumas/% with previous trauma): NR  Single or multiple incident index trauma: Multiple  ITT or completer continuous data: completer | Carlson JG, Chemtob CM, Rusnak K, et al. (1998) Eye movement desensitization and reprocessing (EDMR) treatment for combat‐related posttraumatic stress disorder. Journal of Traumatic Stress 11(1), 3-24 |
| 47 | Edmond 1999/2004 | EMDR: EMDR  Waitlist | Clinically important PTSD symptoms (scoring above a threshold on validated scale) | Childhood sexual abuse - lasted for mean of 6.5 years (the mean age at which abuse began was 6.5 years, and the mean age at which it stopped was 13 years) | 59 | Age range (mean): NR (35)  Gender (% female): 100 BME (% non-white): 15 Country: US Coexisting conditions: NR  Lifetime experience of trauma (mean number of prior traumas/% with previous trauma): 58% of participants also experienced childhood physical abuse and 66% some form of adult revictimization, such as domestic violence and rape  Single or multiple incident index trauma: Multiple  ITT or completer continuous data: ITT post-treatment; completer at follow-up | Edmond T, Rubin A and Wambach K (1999) The effectiveness of EMDR with adult female survivors of childhood sexual abuse. Social Work Research 23, 103-116  Edmond T and Rubin A (2004) Assessing the long-term effects of EMDR: Results from an 18-month follow-up study with adult female survivors of CSA. Journal of child sexual abuse 13(1), 69-86 |
| 48 | Karatzias 2011 | EMDR: EMDR  Combined somatic & cognitive therapies: Emotional freedom technique | PTSD diagnosis according to ICD/DSM criteria (including self-report of diagnosis) | Mixed - Accident (37%), assault/murder (43%), 'other' (20%) | 46 | Age range (mean): 18-65 (40.6)  Gender (% female): 57 BME (% non-white): NR Country: UK Coexisting conditions: NR  Lifetime experience of trauma (mean number of prior traumas/% with previous trauma): NR  Single or multiple incident index trauma: Single  ITT or completer continuous data: ITT | Karatzias T, Power K, Brown K, et al. (2011) A controlled comparison of the effectiveness and efficiency of two psychological therapies for posttraumatic stress disorder: eye movement desensitization and reprocessing vs. emotional freedom techniques. The Journal of nervous and mental disease 199(6), 372-8 |
| 49 | Scheck 1998 | EMDR: EMDR  Counselling: Supportive counselling | Clinically important PTSD symptoms (scoring above a threshold on validated scale) | Mixed - 90% childhood physical/emotional abuse, >50% traumatic sexual experiences, such as rape or child molestation | 67 | Age range (mean):16-25 (20.9)  Gender (% female): 100 BME (% non-white): 38 Country: US Coexisting conditions: NR  Lifetime experience of trauma (mean number of prior traumas/% with previous trauma): NR  Single or multiple incident index trauma: Multiple  ITT or completer continuous data: completer | Scheck MM, Schaeffer JA and Gillette C (1998) Brief psychological intervention with traumatized young women: The efficacy of eye movement desensitization and reprocessing. Journal of traumatic stress 11(1), 25-44 |
| 50 | Ter Heide 2016 | EMDR: EMDR  Non-trauma-focused CBT: Stabilisation as usual | PTSD diagnosis according to ICD/DSM criteria (including self-report of diagnosis) | Witnessing war as a civilian - Refugee sample, with most frequently reported traumatic events being close to death (83%), murder of family or friend (75%) and threatened with torture (72%) | 74 | Age range (mean): NR (41.5)  Gender (% female): 28 BME (% non-white): NR Country: Netherlands Coexisting conditions: 74% comorbid depression  Lifetime experience of trauma (mean number of prior traumas/% with previous trauma): Mean number of types of traumatic events: 13.8 (sd=5.5)  Single or multiple incident index trauma: Multiple  ITT or completer continuous data: completer | Ter Heide FJ, Mooren TM, van de Schoot R, et al. (2016) Eye movement desensitisation and reprocessing therapy v. stabilisation as usual for refugees: Randomised controlled trial. The British Journal of Psychiatry 209(4), 311-318 |
| 51 | van der Kolk 2007 | EMDR: EMDR  SSRI: fluoxetine | PTSD diagnosis according to ICD/DSM criteria (including self-report of diagnosis) | Mixed - 28% child sexual abuse; 5% child physical abuse; 9% child sexual and physical abuse; 9% adult sexual assault; 6% adult physical assault; 8% domestic violence; 7% other adult victimization; 9% traumatic loss; 3% war/terrorism/violence; 16% injury/accident | 88 | Age range (mean): NR (36.1)  Gender (% female): 83 BME (% non-white): 33 Country: US Coexisting conditions: Mean 3.2 comorbid Axis I/II diagnoses  Lifetime experience of trauma (mean number of prior traumas/% with previous trauma): NR  Single or multiple incident index trauma: Multiple  ITT or completer continuous data: ITT | Van der Kolk B, Spinazzola J, Blaustein M, et al. (2007) A randomized clinical trial of EMDR, fluoxetine and pill placebo in the treatment of PTSD: Treatment effects and long-term maintenance. Journal of Clinical Psychiatry 68(1), 37-46 |
| 52 | Yurtsever 2018 | EMDR: EMDR group  Waitlist | PTSD diagnosis according to ICD/DSM criteria (including self-report of diagnosis)  Note: Data is only reported for those who met diagnostic criteria (assessed with MINI) even though this was not an inclusion/exclusion criterion. | Witnessing war as a civilian: Syrian refugees residing in a refugee camp in southeast Turkey on the Syrian border | 67 | Age range (mean): NR (37.5)  Gender (% female): 77 BME (% non-white): NR Country: Turkey Coexisting conditions: NR  Lifetime experience of trauma (mean number of prior traumas/% with previous trauma): NR  Single or multiple incident index trauma: Multiple  ITT or completer continuous data: completer | Yurtsever, A., Konuk, E., Akyüz, T., Zat, Z., Tükel, F., Çetinkaya, M., ... & Shapiro, E. (2018). An Eye Movement Desensitization and Reprocessing (EMDR) Group Intervention for Syrian Refugees With Post-traumatic Stress Symptoms: Results of a Randomized Controlled Trial. Frontiers in psychology, 9. |

## Non-trauma-focused CBT

| Non-trauma-focused CBT | | | | | | | |
| --- | --- | --- | --- | --- | --- | --- | --- |
|  | Study ID | NMA node: intervention | PTSD details | Trauma type | N | Demographics | Reference |
| 53 | Davis 2007 | Non-trauma-focused CBT: CBT for insomnia (CBT-I)  Waitlist | Clinically important PTSD symptoms (scoring above a threshold on validated scale) | Mixed - Most frequently reported types of trauma: car accidents (59%); unwanted sexual contact (59%); physical assault with a weapon (53%) | 43 | Age range (mean): NR (40)  Gender (% female): 82 BME (% non-white): 24 Country: US Coexisting conditions: NR  Lifetime experience of trauma (mean number of prior traumas/% with previous trauma): Mean 4.6 traumatic events (SD=2.0; range 1-9)  Single or multiple incident index trauma: Single  ITT or completer continuous data: ITT | Davis JL and Wright DC (2007) Randomized clinical trial for treatment of chronic nightmares in trauma‐exposed adults. Journal of Traumatic Stress 20(2), 123-33 |
| 54 | Davis 2011 | Non-trauma-focused CBT: CBT for insomnia (CBT-I)  Waitlist | Clinically important PTSD symptoms (scoring above a threshold on validated scale) | Mixed - The most frequent types of trauma reported were unwanted sexual contact (60%), serious accidents (57%), physical assault with a weapon (57%), combat exposure (13%) | 47 | Age range (mean): NR (47)  Gender (% female): 75 BME (% non-white): 19 Country: US Coexisting conditions: NR  Lifetime experience of trauma (mean number of prior traumas/% with previous trauma): Mean 4.6 traumatic events (SD=2.0; range 1-9)  Single or multiple incident index trauma: Single  ITT or completer continuous data: ITT | Davis JL, Rhudy JL, Pruiksma KE, et al. (2011) Physiological predictors of response to exposure, relaxation, and rescripting therapy for chronic nightmares in a randomized clinical trial. Journal of clinical sleep medicine: JCSM: official publication of the American Academy of Sleep Medicine 7(6), 622 |
|  | Foa 1991 | Trauma-focused CBT: Exposure therapy/prolonged exposure (PE)  Non-trauma-focused CBT: Stress inoculation training (SIT)  Counselling: Supportive counselling | **SEE OTHER DETAILS OF THE STUDY UNDER TRAUMA-FOCUSED CBT** | | | | |
| 55 | Ford 2011 | Non-trauma-focused CBT: Affect regulation (individual)  Present-centered therapy: Present-centered therapy  Waitlist | PTSD diagnosis according to ICD/DSM criteria (including self-report of diagnosis) | Mixed (Exposure to victimization or incarceration) | 146 | Age range (mean): 18-45 (30.7)  Gender (% female): 100 BME (% non-white): 59 Country: US Coexisting conditions: Most (72%) participants met Structured Clinical Interview for DSM-IV criteria for a current Axis I disorder other than PTSD. These included anxiety disorders (61%) and depressive (34%), bipolar (8%), or psychotic (9%) disorders  Lifetime experience of trauma (mean number of prior traumas/% with previous trauma):  Single or multiple incident index trauma: Multiple  ITT or completer continuous data: ITT | Ford JD, Steinberg KL and Zhang W (2011) A randomized clinical trial comparing affect regulation and social problem-solving psychotherapies for mothers with victimization-related PTSD. Behavior Therapy 42(4), 560-78 |
|  | Hensel-Dittmann 2011 | Trauma-focused CBT: Narrative exposure therapy (NET)  Non-trauma-focused CBT: Stress inoculation training (SIT) | **SEE OTHER DETAILS OF THE STUDY UNDER TRAUMA-FOCUSED CBT** | | | | |
| 56 | Krakow 2000 | Non-trauma-focused CBT: Imagery rehearsal therapy for nightmares  Waitlist | Clinically important PTSD symptoms (scoring above a threshold on validated scale) | Exposure to sexual abuse or assault - 97% reported history of sexual assault: 50% raped as adults; 54% raped as children; >60% experienced multiple episodes of sexual assault | 169 | Age range (mean): NR (37)  Gender (% female): 100 BME (% non-white): 3 Country: US Coexisting conditions: All participants had regular nightmares (≥1 a week for >6 months) and insomnia  Lifetime experience of trauma (mean number of prior traumas/% with previous trauma): 68% experienced non-sexual violent assaults as adults and 72% as children. 78% reported other traumatic events including unexpected deaths in the family, witnessing violence, motor vehicle accidents, or natural disasters  Single or multiple incident index trauma: Multiple  ITT or completer continuous data: completer | Krakow B, Hollifield M, Schrader R, et al.(2000) A controlled study of imagery rehearsal for chronic nightmares in sexual assault survivors with PTSD: a preliminary report. J Trauma Stress 13(4), 589-609 |
| 57 | Nakamura 2017 | Non-trauma-focused CBT: Mind-Body Bridging (MBB)  Attention placebo | Clinically important PTSD symptoms (scoring above a threshold on validated scale) | Military combat - Gulf War veterans (US military service members with sleep and physical health complaints who were deployed in 1990–1991). Mean months in Persian Gulf War 7.3 (SD=3.8); Mean months of service 7.5 (SD=3.3); Mean years in military 15.1 (SD=8.1) | 60 | Age range (mean): 39-69 (10)  Gender (% female): 10 BME (% non-white): 12 Country: US Coexisting conditions: All participants had self-reported sleep disturbance and Gulf War Illness (inclusion criteria)  Lifetime experience of trauma (mean number of prior traumas/% with previous trauma): NR  Single or multiple incident index trauma: Multiple  ITT or completer continuous data: ITT | Nakamura Y, Lipschitz DL, Donaldson GW, et al. (2017) Investigating Clinical Benefits of a Novel Sleep-Focused Mind-Body Program on Gulf War Illness Symptoms: A Randomized Controlled Trial. Psychosomatic medicine 79(6), 706-18 |
|  | Ter Heide 2016 | EMDR: EMDR  Non-trauma-focused CBT: Stabilisation as usual | **SEE OTHER DETAILS OF THE STUDY UNDER EMDR** | | | | |

## Combined somatic/cognitive therapies

| Combined somatic/cognitive therapies | | | | | | | |
| --- | --- | --- | --- | --- | --- | --- | --- |
|  | Study ID | NMA node: intervention | PTSD details | Trauma type | N | Demographics | Reference |
| 58 | Church 2013/2014 | Combined somatic and cognitive therapies: Emotional freedom technique (EFT)  Waitlist | Clinically important PTSD symptoms (scoring above a threshold on validated scale) | Military combat - 41% Gulf war era deployments; 58% other deployments. Mean number of tours 1.2 (sd=0.4) | 59 | Age range (mean): 24-86 (51.7)  Gender (% female): 10  BME (% non-white): NR Country: US Coexisting conditions: NR  Lifetime experience of trauma (mean number of prior traumas/% with previous trauma): NR  Single or multiple incident index trauma: Multiple  ITT or completer continuous data: completer | Church D, Hawk C, Brooks AJ, et al. (2013) Psychological trauma symptom improvement in veterans using emotional freedom techniques: a randomized controlled trial. The Journal of nervous and mental disease 201(2), 153-60  Church D (2014) Reductions in pain, depression, and anxiety symptoms after PTSD remediation in veterans. Explore: The Journal of Science and Healing 10(3), 162-9 |
| 59 | Connolly 2011 | Combined somatic/ cognitive therapies: Thought field therapy (TFT)  Waitlist | Clinically important PTSD symptoms (scoring above a threshold on validated scale) | Witnessing war as a civilian - Rwandan genocide (1994) survivors. Reported experiences during the 1994 genocide included: being beaten (60%), having been abused (55.2%), witnessing others being beaten (80%), witnessing others being killed (85.5%), hearing others being hit or beaten (81.4%) and being forced to do things they were against (22.1%) | 171 | Age range (mean): 18-73 (38)  Gender (% female): 82 BME (% non-white): NR Country: Rwanda Coexisting conditions: NR  Lifetime experience of trauma (mean number of prior traumas/% with previous trauma): NR  Single or multiple incident index trauma: Multiple  ITT or completer continuous data: completer | Connolly S and Sakai C (2011) Brief trauma intervention with Rwandan genocide-survivors using Thought Field Therapy. International Journal of Emergency Mental Health 13(3), 161 |
|  | Karatzias 2011 | EMDR: EMDR  Combined somatic/ cognitive therapies: Emotional freedom technique | **SEE OTHER DETAILS OF THE STUDY UNDER EMDR** | | | | |
| 60 | Robson 2016 | Combined somatic/ cognitive therapies: Thought field therapy (TFT)  Waitlist | Clinically important PTSD symptoms (scoring above a threshold on validated scale) | Witnessing war as a civilian (Western Uganda, where there had been intermittent conflict since Uganda gained independence in 1963) | 256 | Age range (mean): NR (44.7)  Gender (% female): 85 BME (% non-white): NR Country: Uganda Coexisting conditions: NR  Lifetime experience of trauma (mean number of prior traumas/% with previous trauma): NR  Single or multiple incident index trauma: Multiple  ITT or completer continuous data: completer | Robson R, Robson P, Ludwig R, et al. (2016) Effectiveness of Thought Field Therapy Provided by Newly Instructed Community Workers to a Traumatized Population in Uganda: A Randomized Trial. Current Research in Psychology 1, 1-11 |

## Present-centered therapy

| Present-centered therapy | | | | | | | |
| --- | --- | --- | --- | --- | --- | --- | --- |
|  | Study ID | NMA node: intervention | PTSD details | Trauma type | N | Demographics | Reference |
|  | Ford 2011 | Non-trauma-focused CBT: Affect regulation (individual)  Present-centered therapy: Present-centered therapy  Waitlist | **SEE OTHER DETAILS OF THE STUDY UNDER NON-TRAUMA-FOCUSED CBT** | | | | |
|  | Ghafoori 2017 | Trauma-focused CBT: Exposure therapy/prolonged exposure (PE)  Present-centered therapy: Present-centered therapy | **SEE OTHER DETAILS OF THE STUDY UNDER TRAUMA-FOCUSED CBT** | | | | |
|  | McDonagh 2005 | Trauma-focused CBT: Exposure therapy/prolonged exposure (PE)  Present-centered therapy: Present-centered therapy  Waitlist | **SEE OTHER DETAILS OF THE STUDY UNDER TRAUMA-FOCUSED CBT** | | | | |

## Self-help with support

| Self-help with support | | | | | | | |
| --- | --- | --- | --- | --- | --- | --- | --- |
|  | Study ID | NMA node: intervention | PTSD details | Trauma type | N | Demographics | Reference |
| 61 | Ivarsson 2014 | Self-help with support: Computerised trauma-focused CBT with support  Waitlist | PTSD diagnosis according to ICD/DSM criteria (including self-report of diagnosis) | Mixed - Sexual, physical, and/or psychological abuse by partner (23%); life-threatening disease (13%); severe offense by significant other (perceived as threatening to integrity) (10%); life-threatening accident (8%); non-sexual assault by stranger (8%); murder of close relative (6%); non-sexual assault by family member (5%); death of close relative (5%); severe maltreatment in health care (5%); multiple stressors (5%); life-threatening disease of close relative (3%); military combat (3%); torture (2%); rape by stranger (2%); rape by family member (2%); tsunami disaster (2%) | 62 | Age range (mean): 21-67 (46)  Gender (% female): 82  BME (% non-white): NR  Country: Sweden  Coexisting conditions: NR  Lifetime experience of trauma (mean number of prior traumas/% with previous trauma): 41% had experienced more than one traumatic event  Single or multiple incident index trauma: Single  ITT or completer continuous data: completer | Ivarsson D, Blom M, Hesser H, et al. (2014) Guided internet-delivered cognitive behavior therapy for post-traumatic stress disorder: a randomized controlled trial. Internet interventions 1(1), 33-40 |
| 62 | Knaevelsrud 2015 | Self-help with support: Computerised trauma-focused CBT with support  Waitlist | Clinically important PTSD symptoms (scoring above a threshold on validated scale) | Witnessing war as a civilian - Sexual violence (war-related and sexual abuse; 40%); experienced the killing of a family member or close person (15%); being exposed to violence (e.g., kidnapping, witnessing bomb attacks) and war or torture (19%); Others (e.g., kidnapping, witnessing bomb attacks) (33%) | 159 | Age range (mean): 18-56 (28.1)  Gender (% female): 72 BME (% non-white): NR Country: Iraq Coexisting conditions: NR  Lifetime experience of trauma (mean number of prior traumas/% with previous trauma): Mean 3.4 traumatic events  Single or multiple incident index trauma: Multiple  ITT or completer continuous data: ITT | Knaevelsrud C, Brand J, Lange A, et al. (2015) Web-based psychotherapy for posttraumatic stress disorder in war-traumatized Arab patients: randomized controlled trial. Journal of medical Internet research17(3) |
| 63 | Knaevelsrud 2017 | Self-help with support: Computerised trauma-focused CBT with support  Waitlist | Clinically important PTSD symptoms (scoring above a threshold on validated scale) | Witnessing war as a civilian (World War II) | 94 | Age range (mean): 63-85 (71.4)  Gender (% female): 65 BME (% non-white): NR Country: Germany Coexisting conditions: NR  Lifetime experience of trauma (mean number of prior traumas/% with previous trauma): NR  Single or multiple incident index trauma: Multiple  ITT or completer continuous data: ITT | Knaevelsrud C, Böttche M, Pietrzak RH, et al. (2017) Efficacy and Feasibility of a Therapist-Guided Internet-Based Intervention for Older Persons with Childhood Traumatization: A Randomized Controlled Trial. The American Journal of Geriatric Psychiatry |
| 64 | Lewis 2017 | Self-help with support: Computerised trauma-focused CBT with support  Waitlist | PTSD diagnosis according to ICD/DSM criteria (including self-report of diagnosis) | Mixed - Transportation accidents (21%); witnessing a sudden, violent, or accidental death (21%); traumatic childbirth or stillbirth (19%); sexual assault or rape (12%); physical attack (10%); life threatening illness or injury (7%); serious accident (2%); learning of the violent death of a loved one (2%); seeing a mutilated body (2%); and being held hostage/detained (2%) | 42 | Age range (mean): 20-65 (39.3)  Gender (% female): 60  BME (% non-white): BR Country: US Coexisting conditions: NR  Lifetime experience of trauma (mean number of prior traumas/% with previous trauma): NR  Single or multiple incident index trauma: Single  ITT or completer continuous data: ITT | Lewis CE, Farewell D, Groves V, et al. (2017) Internet‐based guided self‐help for posttraumatic stress disorder (ptsd): Randomized controlled trial. Depression and anxiety 34(6), 555-65 |
| 65 | Littleton 2016 | Self-help with support: Computerised trauma-focused CBT with support  Self-help without support: psychoeducational website | PTSD diagnosis according to ICD/DSM criteria (including self-report of diagnosis) | Exposure to sexual abuse or assault (Women who had experienced a completed rape since the age of 14) | 87 | Age range (mean): 18-42(22)  Gender (% female): 100 BME (% non-white): 54 Country: US Coexisting conditions: NR  Lifetime experience of trauma (mean number of prior traumas/% with previous trauma): >50% had experienced some other form of interpersonal violence, with childhood/adolescent physical and/or sexual abuse being most commonly reported, followed by physical abuse by a romantic partner  Single or multiple incident index trauma: Single  ITT or completer continuous data: completer | Littleton H, Grills AE, Kline KD, et al. (2016) The From Survivor to Thriver program: RCT of an online therapist-facilitated program for rape-related PTSD. Journal of anxiety disorders 43, 41-51 |
|  | van Emmerik 2008 | Trauma-focused CBT: CBT individual  Self help with support: Structured writing therapy  Waitlist | **SEE OTHER DETAILS OF THE STUDY UNDER TRAUMA-FOCUSED CBT** | | | | |

## Self-help without support

| Self-help without support | | | | | | | |
| --- | --- | --- | --- | --- | --- | --- | --- |
|  | Study ID | NMA node: intervention | PTSD details | Trauma type | N | Demographics | Reference |
|  | Ehlers 2003 | Trauma-focused CBT: Cognitive therapy  Self-help (without support): Cognitive bibliotherapy  Waitlist | **SEE OTHER DETAILS OF THE STUDY UNDER TRAUMA-FOCUSED CBT** | | | | |
| 66 | Henderson 2007 | Self-help (without support): Mandalas (expressive drawing)  Attention placebo | Clinically important PTSD symptoms (scoring above a threshold on validated scale) | Mixed - Assault (8%); motor vehicle accident (11%); death or suicide of a family member or close friend (19%), physical abuse (11%); separation of parents or other family stressor (11%); serious health concern of family or self (11%); sexual abuse (11%); verbal abuse (6%); witness to a traumatic event (11%) | 36 | Age range (mean): 18-23 (18.4)  Gender (% female): 78 BME (% non-white): NR Country: US Coexisting conditions: NR  Lifetime experience of trauma (mean number of prior traumas/% with previous trauma): NR  Single or multiple incident index trauma: Single  ITT or completer continuous data: ITT | Henderson P, Rosen D and Mascaro N (2007) Empirical study on the healing nature of mandalas. Psychology of Aesthetics, Creativity, and the Arts 1(3), 148 |
| 67 | Hirai 2005 | Self-help (without support): Computerised trauma-focused CBT  Waitlist | Clinically important PTSD symptoms (scoring above a threshold on validated scale) | Mixed - MVCs (33%), interpersonal violence (22%), eye-witnessed traumatic events (11%), life-threatening disease (11%), illness or traumatic loss (22%) | 36 | Age range (mean): NR (29.4)  Gender (% female): 78  BME (% non-white): 22  Country: US  Coexisting conditions: NR  Lifetime experience of trauma (mean number of prior traumas/% with previous trauma): NR  Single or multiple incident index trauma: Single  ITT or completer continuous data: completer | Hirai M and Clum GA (2005) An Internet‐based self‐change program for traumatic event related fear, distress, and maladaptive coping. Journal of traumatic stress 2005 18(6), 631-6 |
| 68 | Kuhn 2017 | Self-help (without support): Computerised non-trauma-focused CBT  Waitlist | Clinically important PTSD symptoms (scoring above a threshold on validated scale) | Mixed - Physical assault (47%); sexual assault (14%); serious accident (21%); life-threatening illness or injury (6%); disaster exposure (3%); combat exposure (3%); other event (7%) | 120 | Age range (mean): NR (39.3)  Gender (% female): 69 BME (% non-white): 33 Country: US Coexisting conditions: NR  Lifetime experience of trauma (mean number of prior traumas/% with previous trauma): Mean number of traumatic event types 8.5 (SD=3.5). Lifetime trauma exposure: Physical assault (87%); Sexual assault (73%); Serious accident (79%); Life-threatening illness or injury (60%); Disaster exposure (74%); Combat exposure (7%); Other event (93%)  Single or multiple incident index trauma: Single  ITT or completer continuous data: ITT | Kuhn E, Kanuri N, Hoffman JE, et al. (2017) A randomized controlled trial of a smartphone app for posttraumatic stress disorder symptoms. Journal of consulting and clinical psychology 85(3), 267 |
|  | Littleton 2016 | Self-help with support: Computerised trauma-focused CBT with support  Self-help without support: psychoeducational website | **SEE OTHER DETAILS OF THE STUDY UNDER SELF-HELP WITH SUPPORT** | | | | |
| 69 | Miner 2016 | Self-help (without support): Computerised trauma-focused CBT  Waitlist | Clinically important PTSD symptoms (scoring above a threshold on validated scale) | Unclear | 49 | Age range (mean): NR (45.7)  Gender (% female): 82 BME (% non-white): 43 Country: US Coexisting conditions: NR  Lifetime experience of trauma (mean number of prior traumas/% with previous trauma): NR  Single or multiple incident index trauma: Unclear  ITT or completer continuous data: ITT | Miner A, Kuhn E, Hoffman JE, et al. (2016) Feasibility, acceptability, and potential efficacy of the PTSD Coach app: A pilot randomized controlled trial with community trauma survivors. Psychological Trauma: Theory, Research, Practice, and Policy 8(3), 384 |
| 70 | Sloan 2004 | Self-help (without support): Expressive writing  Attention placebo | Clinically important PTSD symptoms (scoring above a threshold on validated scale) | Mixed - The types of traumatic events endorsed by the participants included rape, witness to murder, physical assault by stranger, life-threatening car accident, and childhood sexual assault by family member | 51 | Age range (mean): NR (18.9)  Gender (% female): 100 BME (% non-white): 51 Country: US Coexisting conditions: NR  Lifetime experience of trauma (mean number of prior traumas/% with previous trauma): 63% reported experiencing more than one traumatic event  Single or multiple incident index trauma: Unclear  ITT or completer continuous data: completer | Sloan DM and Marx BP (2004) A closer examination of the structured written disclosure procedure. Journal of consulting and clinical psychology 72(2), 165 |
| 71 | Sloan 2007 | Self-help (without support): Expressive writing  Attention placebo | Clinically important PTSD symptoms (scoring above a threshold on validated scale) | Mixed - The most frequently reported traumatic events were sexual assault (65%), physical assault by stranger (48%), motor vehicle accident (43%), and witness to murder (15%) | 85 | Age range (mean): NR (18.7)  Gender (% female): 80 BME (% non-white): 41 Country: US Coexisting conditions: NR  Lifetime experience of trauma (mean number of prior traumas/% with previous trauma): 68% reported experiencing more than one traumatic event  Single or multiple incident index trauma: Unclear  ITT or completer continuous data: completer | Sloan DM, Marx BP and Epstein EM. (2007) Does altering the writing instructions influence outcome associated with written disclosure? Behavior therapy 38(2), 155-68 |
| 72 | Sloan 2011 | Self-help (without support): Expressive writing  Attention placebo | PTSD diagnosis according to ICD/DSM criteria (including self-report of diagnosis) | Mixed - Index traumatic events included sexual assault (40%), physical assault by stranger (31%), motor vehicle accident (14%), witness to a murder (7%) and warzone experience (7%) | 57 | Age range (mean): NR (18.9)  Gender (% female): NR BME (% non-white): 43 Country: US Coexisting conditions: NR  Lifetime experience of trauma (mean number of prior traumas/% with previous trauma): NR  Single or multiple incident index trauma: Single  ITT or completer continuous data: completer | Sloan DM, Marx BP and Greenberg EM (2011) A test of written emotional disclosure as an intervention for posttraumatic stress disorder. Behaviour Research and Therapy 49(4), 299-304 |
| 73 | Sloan 2012 | Self-help (without support): Expressive writing  Waitlist | PTSD diagnosis according to ICD/DSM criteria (including self-report of diagnosis) | Motor Vehicle Collisions (Not reported in details) | 46 | Age range (mean): NR (40.7)  Gender (% female): 65 BME (% non-white): 63 Country: US Coexisting conditions: 25% major depressive episode, 10% alcohol abuse  Lifetime experience of trauma (mean number of prior traumas/% with previous trauma): Median=10.0 events that met DSM-IV PTSD Criterion A for a traumatic stressor. Approximately 85% of the sample reported a history of physical assault and approximately 60% reported a history of sexual assault  Single or multiple incident index trauma: Single  ITT or completer continuous data: NA (only dichotomous data used) | Sloan DM, Marx BP, Bovin MJ, et al. (2012) Written exposure as an intervention for PTSD: A randomized clinical trial with motor vehicle accident survivors. Behaviour research and therapy 50(10), 627-35 |
| 74 | Spence 2011 | Self-help (without support): Computerised trauma-focused CBT  Waitlist | PTSD diagnosis according to ICD/DSM criteria (including self-report of diagnosis) | Mixed - Trauma types reported to have been experienced personally or witnessed by more than 50% of the treatment group: physical assault (74%), other unwanted sexual experience (70%), sexual assault (57%), transportation accidents (52%), and other stressful experiences (52%) | 44 | Age range (mean): 21-68 (42.6)  Gender (% female): 81 BME (% non-white): NR Country: Australia Coexisting conditions: 57% reported taking medication for anxiety or depression at baseline  Lifetime experience of trauma (mean number of prior traumas/% with previous trauma): Mean number of traumatic events: 6.3. Most participants had experienced multiple types of trauma  Single or multiple incident index trauma: Multiple  ITT or completer continuous data: ITT | Spence J, Titov N, Dear BF, et al. (2011) Randomized controlled trial of Internet‐delivered cognitive behavioral therapy for posttraumatic stress disorder. Depression and anxiety 28(7), 541-50 |
| 75 | Truijens 2014 | Self-help (without support): Expressive writing  Attention placebo | Clinically important PTSD symptoms (scoring above a threshold on validated scale) | Mixed - Traumatic events reported by the participants included having experienced or witnessed an accident (16.4%); physical, mental, or sexual abuse (34.5%); severe illness or death of a loved one (34.5%); and natural disaster or war (14.6%) | 64 | Age range (mean): NR (23.7)  Gender (% female): 82 BME (% non-white): NR Country: Netherlands Coexisting conditions: NR  Lifetime experience of trauma (mean number of prior traumas/% with previous trauma): NR  Single or multiple incident index trauma: Single  ITT or completer continuous data: completer | Truijens FL and van Emmerik AA (2014) Visual feedback in written imaginal exposure for posttraumatic stress: a preliminary study. Journal of Loss and Trauma 19(5), 403-15 |
| 76 | Xu 2016 | Self-help (without support): Computerised trauma-focused CBT  Waitlist | Clinically important PTSD symptoms (scoring above a threshold on validated scale) | Mixed - Witnessing others sudden death (37%); Physical abuse (30%), sexual abuse (17%), serious accident in workplace or at home (17%), fire or natural disasters (8%), traffic accidents (7%), hurting others seriously (4%) | 82 | Age range (mean): NR (NR)  Gender (% female): 75  BME (% non-white): NR  Country: China  Coexisting conditions: NR  Lifetime experience of trauma (mean number of prior traumas/% with previous trauma): NR  Single or multiple incident index trauma: Multiple  ITT or completer continuous data: completer | Xu W, Wang J, Wang Z, et al. (2016) Web-based intervention improves social acknowledgement and disclosure of trauma, leading to a reduction in posttraumatic stress disorder symptoms. Journal of health psychology 21(11), 2695-708 |

## Counselling

| Counselling | | | | | | | |
| --- | --- | --- | --- | --- | --- | --- | --- |
|  | Study ID | NMA node: intervention | PTSD details | Trauma type | N | Demographics | Reference |
|  | Blanchard 2002/2003/2004 | Trauma-focused CBT: CBT individual  Counselling: Supportive counselling  Waitlist | **SEE OTHER DETAILS OF THE STUDY UNDER TRAUMA-FOCUSED CBT** | | | | |
|  | Bryant 2003a | Trauma-focused CBT: Exposure therapy/prolonged exposure (PE)  Counselling: Supportive counselling | **SEE OTHER DETAILS OF THE STUDY UNDER TRAUMA-FOCUSED CBT** | | | | |
|  | Castillo 2016 | Trauma-focused CBT: Imaginal exposure  Counselling: Supportive counselling | **SEE OTHER DETAILS OF THE STUDY UNDER TRAUMA-FOCUSED CBT** | | | | |
|  | Cloitre 2010 | Trauma-focused CBT: Exposure therapy/prolonged exposure (PE)  Counselling: Supportive counselling | **SEE OTHER DETAILS OF THE STUDY UNDER TRAUMA-FOCUSED CBT** | | | | |
|  | Cottraux 2008 | Trauma-focused CBT: Exposure therapy/prolonged exposure (PE)  Counselling: Supportive counselling | **SEE OTHER DETAILS OF THE STUDY UNDER TRAUMA-FOCUSED CBT** | | | | |
|  | Ehlers 2014 | Trauma-focused CBT: Cognitive therapy  Counselling: Supportive counselling  Waitlist | **SEE OTHER DETAILS OF THE STUDY UNDER TRAUMA-FOCUSED CBT** | | | | |
|  | Foa 1991 | Trauma-focused CBT: Exposure therapy/prolonged exposure (PE)  Non-trauma-focused CBT: Stress inoculation training (SIT)  Counselling: Supportive counselling | **SEE OTHER DETAILS OF THE STUDY UNDER TRAUMA-FOCUSED CBT** | | | | |
|  | Katz 2014 | Trauma-focused CBT: Exposure therapy/prolonged exposure (PE)  Counselling: Supportive counselling | **SEE OTHER DETAILS OF THE STUDY UNDER TRAUMA-FOCUSED CBT** | | | | |
|  | Neuner 2008 | Trauma-focused CBT: Narrative exposure therapy (NET)  Counselling: Supportive counselling | **SEE OTHER DETAILS OF THE STUDY UNDER TRAUMA-FOCUSED CBT** | | | | |
|  | Scheck 1998 | EMDR: EMDR  Counselling: Supportive counselling | **SEE OTHER DETAILS OF THE STUDY UNDER EMDR** | | | | |
| 77 | Yeomans 2010 | Counselling: Supportive psychotherapy group  Waitlist | Clinically important PTSD symptoms (scoring above a threshold on validated scale) | Witnessing war as a civilian - Almost all participants had been directly victimized by violence during or since the onset of conflict in Burundi in 1993. Frequency and types of events: Combat situation (99% experienced; 0.4% witnessed); Forced to hide (97% experienced; 0.8% witnessed); Unnatural death of family member (97% experienced; 0.8% witnessed); Lack of food and water (95% experienced; 0.4% witnessed); Narrowly escaping death (92% experienced; 6% witnessed); Lack of shelter (90% experienced); Ill health and no medical care (86% experienced; 8% witnessed); Loss of personal property (82% experienced; 9% witnessed); Confined to indoors because of danger (80% experienced; 6% witnessed); Betrayed and placed at risk of death (42% experienced; 18% witnessed); Serious physical injury from  combat (35% experienced; 45% witnessed); Forced to hide among the dead (28% experienced; 23% witnessed); Imprisonment (24% experienced; 18% witnessed); Sexual abuse/humiliation (10% experienced; 25% witnessed); Forced to harm or kill a stranger (10% experienced; 25% witnessed); Forced to harm or kill a family member or friend (9% experienced; 24% witnessed); Disappearance/kidnapping of spouse (9% experienced; 18% witnessed); Rape (5% experienced; 25% witnessed); Disappearance/kidnapping of son or daughter (4% experienced; 20% witnessed) | 124 | Age range (mean): NR (38.6)  Gender (% female): 44 BME (% non-white): NR Country: Burundi Coexisting conditions:  Lifetime experience of trauma (mean number of prior traumas/% with previous trauma): Mean number of types of events experienced was 9.9 (SD=2.1). The mean number of types of events experienced or witnessed was 12.6 (SD = 3.2)  Single or multiple incident index trauma: Multiple  ITT or completer continuous data: completer | Yeomans PD, Forman EM, Herbert JD and Yuen E (2010) A randomized trial of a reconciliation workshop with and without PTSD psychoeducation in Burundian sample. Journal of traumatic stress 23(3), 305-12 |

## Attention bias modification

| Attention bias modification | | | | | | | |
| --- | --- | --- | --- | --- | --- | --- | --- |
|  | Study ID | NMA node: intervention | PTSD details | Trauma type | N | Demographics | Reference |
| 78 | Bar-Haim 2011/Badura-Brack 2015 study 1 | Attention Bias Modification: Attention Bias Modification  Attention placebo | PTSD diagnosis according to ICD/DSM criteria (including self-report of diagnosis) | Military combat (Israel Defence Forces veterans) | 52 | Age range (mean): 22-65 (36.1)  Gender (% female): 0  BME (% non-white): NR Country: Israel Coexisting conditions: 55% depression; 39% GAD; 15% Personality Disorder- Cluster B  Lifetime experience of trauma (mean number of prior traumas/% with previous trauma): NR  Single or multiple incident index trauma: Multiple  ITT or completer continuous data: ITT | Bar-Haim Y and Fruchter E (2011) Attention Bias Modification Treatment for Patients With Post Traumatic Stress Disorder (PTSD) [NCT01368302]. Available from: https://clinicaltrials.gov/ct2/show/NCT01368302 [accessed 26.07.2017]  Badura-Brack AS, Naim R, Ryan TJ, et al. (2015) Effect of attention training on attention bias variability and PTSD symptoms: randomized controlled trials in Israeli and US combat veterans. American journal of psychiatry 172(12), 1233-41 |
| 79 | Bar-Haim 2011/Badura-Brack 2015 study 2 | Attention Bias Modification: Attention Bias Modification  Attention placebo | PTSD diagnosis according to ICD/DSM criteria (including self-report of diagnosis) | Military combat (US military veterans who served in recent conflicts in Iraq and Afghanistan) | 46 | Age range (mean): NR (36.3)  Gender (% female): 0 BME (% non-white): NR Country: US Coexisting conditions: 59% depression; 8% GAD; 16% panic disorder; 4% social phobia; 4% Personality Disorder- Cluster B  Lifetime experience of trauma (mean number of prior traumas/% with previous trauma): NR  Single or multiple incident index trauma: Multiple  ITT or completer continuous data: ITT | Bar-Haim Y and Fruchter E (2011) Attention Bias Modification Treatment for Patients With Post Traumatic Stress Disorder (PTSD) [NCT01368302]. Available from: https://clinicaltrials.gov/ct2/show/NCT01368302 [accessed 26.07.2017]  Badura-Brack AS, Naim R, Ryan TJ, et al. (2015) Effect of attention training on attention bias variability and PTSD symptoms: randomized controlled trials in Israeli and US combat veterans. American journal of psychiatry 172(12), 1233-41 |
| 80 | Schoorl 2013 | Attention Bias Modification: Attention Bias Modification  Attention placebo | PTSD diagnosis according to ICD/DSM criteria (including self-report of diagnosis) | Unclear | 102 | Age range (mean): NR (37.1)  Gender (% female): 75  BME (% non-white): NR Country: Netherlands Coexisting conditions: 2.7 additional diagnoses per patient. Depression: 70%, Dysthymia: 13%, Panic: 33%, Social anxiety: 36%, GAD: 38%, OCD: 16%, Somatization: 8%  Lifetime experience of trauma (mean number of prior traumas/% with previous trauma): 93% 2+ traumas. Most of the patients had experienced multiple traumas (93.1%). More than half (56.9%) of the patients had been traumatized in childhood and 40.6% had experienced both childhood trauma and more recent trauma  Single or multiple incident index trauma: Multiple  ITT or completer continuous data: completer | Schoorl M, Putman P and van Der Does W (2013) Attentional bias modification in posttraumatic stress disorder: a randomized controlled trial. Psychotherapy and psychosomatics 82(2), 99-105 |

## Interpersonal Psychotherapy (IPT)

| Interpersonal Psychotherapy (IPT) | | | | | | | |
| --- | --- | --- | --- | --- | --- | --- | --- |
|  | Study ID | NMA node: intervention | PTSD details | Trauma type | N | Demographics | Reference |
| 81 | Krupnick 2008 | Interpersonal psychotherapy (IPT): IPT (group)  Waitlist | PTSD diagnosis according to ICD/DSM criteria (including self-report of diagnosis) | Mixed - Study participants had experienced multiple episodes of trauma, usually beginning in childhood. 98% sexual assault (96% first assaulted before age 12); 96% physical assault before age 12 | 48 | Age range (mean): NR (32)  Gender (% female): 100 BME (% non-white): 94  Country: US Coexisting conditions: NR  Lifetime experience of trauma (mean number of prior traumas/% with previous trauma): Mean 6.4 prior traumas  Single or multiple incident index trauma: Multiple  ITT or completer continuous data: ITT | Krupnick JL, Green BL, Stockton P, et al. (2008) Group interpersonal psychotherapy for low-income women with posttraumatic stress disorder. Psychotherapy Research 18(5), 497-507 |
|  | Markowitz 2015a | Trauma-focused CBT: Exposure therapy/prolonged exposure (PE)  Interpersonal psychotherapy (IPT): IPT  Relaxation | **SEE OTHER DETAILS OF THE STUDY UNDER TRAUMA-FOCUSED CBT** | | | | |

## Metacognitive therapy

| Metacognitive therapy | | | | | | | |
| --- | --- | --- | --- | --- | --- | --- | --- |
|  | Study ID | NMA node: intervention | PTSD details | Trauma type | N | Demographics | Reference |
| 82 | Wells 2012 | Metacognitive therapy  Waitlist | PTSD diagnosis according to ICD/DSM criteria (including self-report of diagnosis) | Mixed - Assault (35%), MVC (20%), robbery (10%), sexual assault (15%), witness (10%), work accident (10%) | 20 | Age range (mean): NR (37.4)  Gender (% female): 55 BME (% non-white): NR  Country: UK  Coexisting conditions: 15% minor depressive disorder; 45% major depressive disorder; 15% GAD  Lifetime experience of trauma (mean number of prior traumas/% with previous trauma): Median number of traumas=1/1.5  Single or multiple incident index trauma: Single  ITT or completer continuous data: ITT | Wells A and Colbear JS (2012) Treating posttraumatic stress disorder with metacognitive therapy: A preliminary controlled trial. Journal of Clinical Psychology 68(4), 373-81 |

## Couple intervention

| Couple intervention | | | | | | | |
| --- | --- | --- | --- | --- | --- | --- | --- |
|  | Study ID | NMA node: intervention | PTSD details | Trauma type | N | Demographics | Reference |
| 83 | Monson 2008/2012 | Couple intervention: Cognitive-behavioural conjoint therapy  Waitlist | PTSD diagnosis according to ICD/DSM criteria (including self-report of diagnosis) | Mixed - Adult sexual trauma (20%); child sexual trauma (28%); noncombat physical assault (15%); motor vehicle collision (8%); witnessing/learning about death/illness (13%); combat (5%); other (13%) | 40 | Age range (mean): NR (37.1)  Gender (% female): 75 BME (% non-white): 28 Country: US and Canada Coexisting conditions: 63% any comorbidity, 40% mood disorder, 30% anxiety disorder, 0% substance abuse, 10% 'other'.  Lifetime experience of trauma (mean number of prior traumas/% with previous trauma): NR  Single or multiple incident index trauma: Unclear  ITT or completer continuous data: NA (only dichotomous data used) | Monson CM and Vorstenbosch V (2008) Cognitive-behavioral couples therapy for posttraumatic stress disorder [NCT00669981]. Available from: https://clinicaltrials.gov/ct2/show/NCT00669981 [accessed 08.08.2017]  Monson CM, Fredman SJ, Macdonald A, et al. (2012) Effect of cognitive-behavioral couple therapy for PTSD: A randomized controlled trial. Jama 308(7), 700-9 |
| 84 | Sautter 2015 | Couple intervention: Cognitive-behavioural conjoint therapy  Psychoeducation: PTSD family education | PTSD diagnosis according to ICD/DSM criteria (including self-report of diagnosis) | Military combat - Veterans of Operation Iraqi Freedom (OIF)/Operation Enduring Freedom (OEF) | 57 | Age range (mean): NR (33.1)  Gender (% female): 2 BME (% non-white): 34 Country: US Coexisting conditions: NR  Lifetime experience of trauma (mean number of prior traumas/% with previous trauma): NR  Single or multiple incident index trauma: Multiple  ITT or completer continuous data: completer | Sautter FJ, Glynn SM, Cretu JB, et al. (2015) Efficacy of structured approach therapy in reducing PTSD in returning veterans: A randomized clinical trial. Psychological services12(3), 199 |

## Psychoeducation

| Psychoeducation | | | | | | | |
| --- | --- | --- | --- | --- | --- | --- | --- |
|  | Study ID | NMA node: intervention | PTSD details | Trauma type | N | Demographics | Reference |
|  | Chambers 2014 | Trauma-focused CBT: CBT individual  Psychoeducation: single psychoeducational phonecall | **SEE OTHER DETAILS OF THE STUDY UNDER TRAUMA-FOCUSED CBT** | | | | |
| 85 | Ghafoori 2016 | Psychoeducation: Single psychoeducation session  Waitlist | Clinically important PTSD symptoms (scoring above a threshold on validated scale) | Unclear (not reported in details) | 86 | Age range (mean): NR (NR)  Gender (% female): 45 BME (% non-white): 73 Country: US Coexisting conditions: NR  Lifetime experience of trauma (mean number of prior traumas/% with previous trauma): Mean number of lifetime traumas 8.3 (SD=3.6)  Single or multiple incident index trauma: Unclear  ITT or completer continuous data: completer | Ghafoori B, Fisher D, Korosteleva O and Hong M (2016) A Randomized, Controlled Pilot Study of a Single-Session Psychoeducation Treatment for Urban, Culturally Diverse, Trauma-Exposed Adults. The Journal of nervous and mental disease 204(6), 421-30 |
|  | Sautter 2015 | Couple intervention: Cognitive-behavioural conjoint therapy  Psychoeducation: PTSD family education | **SEE OTHER DETAILS OF THE STUDY UNDER COUPLE INTERVENTION** | | | | |

## Behavioural therapy

| Behavioural therapy | | | | | | | |
| --- | --- | --- | --- | --- | --- | --- | --- |
|  | Study ID | NMA node: intervention | PTSD details | Trauma type | N | Demographics | Reference |
| 86 | Basoglu 2005 | Behavioural therapy: Imaginal exposure  Waitlist | PTSD diagnosis according to ICD/DSM criteria (including self-report of diagnosis) | Natural disasters (such as severe floods, earthquakes or tsunamis) – Survivors of earthquake in Turkey on August 17, 1999: 20% survivors were trapped under rubble; 39% suffered varying degrees of physical injury; 5% lost at least one first-degree relative and 70% lost at least a second-degree relative or a friend; 19% survivors participated in rescue work | 59 | Age range (mean): NR (36.3)  Gender (% female): 85 BME (% non-white): NR Country: Turkey Coexisting conditions: NR  Lifetime experience of trauma (mean number of prior traumas/% with previous trauma): 63% previous trauma (MVCs, fire, floods)  Single or multiple incident index trauma: Single  ITT or completer continuous data: ITT | Basoglu M, Salcioglu E and Livanou M (2005) Single-session behavioural treatment of earthquake-related posttraumatic stress disorder: a randomised waiting list controlled trial, Journal of Traumatic Stress 18, 1-11 |
| 87 | Basoglu 2007 | Behavioural therapy: In vivo exposure  Waitlist | PTSD diagnosis according to ICD/DSM criteria (including self-report of diagnosis) | Natural disasters (such as severe floods, earthquakes or tsunamis) – Survivors of earthquake in Turkey on August 17, 1999: 20% survivors were trapped under rubble; 39% suffered varying degrees of physical injury; 5% lost at least one first-degree relative and 70% lost at least a second-degree relative or a friend; 19% survivors participated in rescue work | 31 | Age range (mean): NR (34)  Gender (% female): 87 BME (% non-white): NR Country:  Coexisting conditions: Major depression: 36%, Panic disorder: 10%, panic disorder with agoraphobia: 19%  Lifetime experience of trauma (mean number of prior traumas/% with previous trauma): NR  Single or multiple incident index trauma: Single  ITT or completer continuous data: ITT | Başoğlu M, Şalcioğlu E and Livanou M (2007) A randomized controlled study of single-session behavioural treatment of earthquake-related post-traumatic stress disorder using an earthquake simulator. Psychological medicine 37(2), 203-13 |

## Resilience-oriented treatment

| Resilience-oriented treatment | | | | | | | |
| --- | --- | --- | --- | --- | --- | --- | --- |
|  | Study ID | NMA node: intervention | PTSD details | Trauma type | N | Demographics | Reference |
| 88 | Kent 2011 | Resilience-oriented treatment: Resilience-oriented treatment  Waitlist | Clinically important PTSD symptoms (scoring above a threshold on validated scale) | Mixed - All participants were veterans from the Vietnam war era up through the Gulf war. The traumas indexed by the CAPS were combat (31%), childhood sexual abuse (21%), childhood physical abuse (18%), violent unexpected death of another (14%), sexual assault (6%), physical assault (5%), and accident (5%) | 39 | Age range (mean): 34-66 (54)  Gender (% female): 33 BME (% non-white): 24  Country: US Coexisting conditions: NR  Lifetime experience of trauma (mean number of prior traumas/% with previous trauma): NR  Single or multiple incident index trauma: Multiple  ITT or completer continuous data: ITT | Kent M, Davis MC, Stark SL and Stewart LA (2011) A resilience‐oriented treatment for posttraumatic stress disorder: Results of a preliminary randomized clinical trial. Journal of traumatic stress 24(5), 591-5 |

## Family therapy

| Family therapy | | | | | | | |
| --- | --- | --- | --- | --- | --- | --- | --- |
|  | Study ID | NMA node: intervention | PTSD details | Trauma type | N | Demographics | Reference |
| 89 | Kazak 2004 | Family therapy: Family therapy group  Waitlist | Clinically important PTSD symptoms (scoring above a threshold on validated scale) | Family member or carer of person with life-threatening illness or injury (Mothers of childhood cancer survivors) | 146 | Age range (median): 26-59 (42.9)  Gender (% female): 100 BME (% non-white): 12 Country: US  Coexisting conditions: NR  Lifetime experience of trauma (mean number of prior traumas/% with previous trauma): NR  Single or multiple incident index trauma: Single  ITT or completer continuous data: modified ITT | Kazak AE, Alderfer MA, Streisand R, et al (2004) Treatment of posttraumatic stress symptoms in adolescent survivors of childhood cancer and their families: A randomized clinical trial. Journal of Family Psychology 18(3), 493-504 |

## Psychodynamic therapy

| Psychodynamic therapy | | | | | | | |
| --- | --- | --- | --- | --- | --- | --- | --- |
|  | Study ID | NMA node: intervention | PTSD details | Trauma type | N | Demographics | Reference |
| 90 | Steinert 2017 | Psychodynamic therapy: Resource activation  Waitlist | PTSD diagnosis according to ICD/DSM criteria (including self-report of diagnosis) | Mixed - Domestic violence (23%), sexual abuse (15%), traffic accident (24%), other serious accident, e.g. stepping on a mine (7%), witnessing death of someone close (12%), assault (10%), 'other' such as combat or trafficking (10%) | 86 | Age range (mean): NR (27.5)  Gender (% female): 61 BME (% non-white): NR  Country: Cambodia Coexisting conditions: NR  Lifetime experience of trauma (mean number of prior traumas/% with previous trauma): NR  Single or multiple incident index trauma: Single  ITT or completer continuous data: NA (only dichotomous data used) | Steinert C, Bumke PJ, Hollekamp RL, et al. (2017) Resource activation for treating post-traumatic stress disorder, co-morbid symptoms and impaired functioning: a randomized controlled trial in Cambodia. Psychological medicine 47(3), 553-64 |

# 
