# Supplementary material for: Cost-effectiveness of psychological treatments for post-traumatic stress disorder in adults
Source: PLoS One. 2020 Apr 30;15(4):e0232245. doi: 10.1371/journal.pone.0232245 (PMC7192458; doi:10.1371/journal.pone.0232245)
Supplement: S6 Appendix — (DOCX) [file pone.0232245.s013.docx]

# **Appendix 6: List of excluded studies with reasons for exclusion**

## Excluded from the systematic review of psychological, psychosocial and other non-pharmacological treatments for PTSD in adults

|  | Study ID | Reference | Reason for exclusion |
| --- | --- | --- | --- |
| 1 | Acosta 2017 | Acosta MC, Possemato K, Maisto SA, Marsch LA, Barrie K, Lantinga L, Fong C, Xie H, Grabinski M, Rosenblum A. Web-delivered CBT reduces heavy drinking in OEF-OIF veterans in primary care with symptomatic substance use and PTSD. Behavior therapy. 2017 Mar 31;48(2):262-76. | Efficacy or safety data cannot be extracted |
| 2 | Adenauer 2011/Catani 2010 | Adenauer H, Catani C, Gola H, Keil J, Ruf M, Schauer M, Neuner F. Narrative exposure therapy for PTSD increases top-down processing of aversive stimuli-evidence from a randomized controlled treatment trial. BMC neuroscience. 2011 Dec 19;12(1):127.  Catani C, Neuner F. Change of Neural Network Indicators Through Narrative Treatment of PTSD in Torture Victims [NCT00563888]. 2010. Available from: https://clinicaltrials.gov/ct2/show/NCT00563888 [accessed 28.07.2017] | Sample size (N<10/arm) |
| 3 | Aderka 2013 | Aderka IM, Gillihan SJ, McLean CP, Foa EB. The relationship between posttraumatic and depressive symptoms during prolonged exposure with and without cognitive restructuring for the treatment of posttraumatic stress disorder. Journal of consulting and clinical psychology. 2013 Jun;81(3):375. | Subgroup/secondary analysis of RCT already included |
| 4 | Adler 2008 | Adler AB, Litz BT, Castro CA, Suvak M, Thomas JL, Burrell L, McGurk D, Wright KM, Bliese PD. A group randomized trial of critical incident stress debriefing provided to US peacekeepers. Journal of traumatic stress. 2008 Jun 1;21(3):253-63. | Population outside scope: Trials of soldiers on active service |
| 5 | Ahmadi 2015 | Ahmadi K, Hazrati M, Ahmadizadeh M, Noohi S. REM desensitization as a new therapeutic method for post-traumatic stress disorder: a randomized controlled trial. Acta Medica Indonesiana. 2015;47(2). | Population outside scope: Trials of soldiers on active service |
| 6 | Albright 2010 | Albright DL, Thyer B. Does EMDR reduce post‐traumatic stress disorder symptomatology in combat veterans?. Behavioral Interventions. 2010 Feb 1;25(1):1-9. | Systematic review with no new useable data and any meta-analysis results not appropriate to extract |
| 7 | Allan 2015 | Allan NP, Short NA, Albanese BJ, Keough ME, Schmidt NB. Direct and mediating effects of an anxiety sensitivity intervention on posttraumatic stress disorder symptoms in trauma-exposed individuals. Cognitive behaviour therapy. 2015 Nov 2;44(6):512-24. | Efficacy or safety data cannot be extracted |
| 8 | Amir 2008 | Amir N. Information Processing Modification in the Treatment of PTSD [NCT00604045]. 2014. Available from: https://clinicaltrials.gov/ct2/show/study/NCT00604045 [accessed 08.08.2017] | Sample size (N<10/arm) |
| 9 | Anderson 2010 | Anderson T, Fende Guajardo J, Luthra R, Edwards KM. Effects of clinician-assisted emotional disclosure for sexual assault survivors: A pilot study. Journal of interpersonal violence. 2010 Jun;25(6):1113-31. | Efficacy or safety data cannot be extracted |
| 10 | Anderson 2014 | Anderson ML, Najavits LM. Does seeking safety reduce PTSD symptoms in women receiving physical disability compensation?. Rehabilitation psychology. 2014 Aug;59(3):349. | Subgroup/secondary analysis that is not relevant |
| 11 | Andersson 2013 | Andersson MA, Conley CS. Optimizing the perceived benefits and health outcomes of writing about traumatic life events. Stress and Health. 2013 Feb 1;29(1):40-9. | Comparison outside protocol |
| 12 | Andre 1997 | Andre, C., Lelord, F., Legeron, P., Reignier, A., & Delattre, A. (1997). Effectiveness of early intervention on 132 bus drivers who have been victims of aggression: A controlled study. Encephale, 23, 65-71. | Non-English language paper |
| 13 | Angelakis 2010 | Angelakis, S. The utility of combining cognitive processing therapy and behavioural activation for individuals with comorbid posttraumatic stress disorder and major depressive disorders: Is there added benefit to combining treatments? 2010. Available from: https://www.anzctr.org.au/Trial/Registration/TrialReview.aspx?ACTRN=12611000541909 [accessed 26.07.2017] | Unpublished (registered on clinical trials registry and author contacted for full trial report but not provided) |
| 14 | Anonymous 2004 | NCT00055354. Acupuncture Diagnosis and Treatment of DSM-IV PTSD. Available from: https://clinicaltrials.gov/ct2/show/NCT00055354 [accessed 26.07.2017] | Paper unavailable |
| 15 | Arabia 2011 | Arabia E, Manca ML, Solomon RM. EMDR for survivors of life-threatening cardiac events: results of a pilot study. Journal of EMDR Practice and Research. 2011 Feb 1;5(1):2-13. | Efficacy or safety data cannot be extracted |
| 16 | Arntz 2007 | Arntz A, Tiesema M, Kindt M. Treatment of PTSD: A comparison of imaginal exposure with and without imagery rescripting. Journal of behavior therapy and experimental psychiatry. 2007 Dec 31;38(4):345-70. | Comparison outside protocol |
| 17 | Arroyo 2017 | Arroyo K, Lundahl B, Butters R, Vanderloo M, Wood DS. Short-term interventions for survivors of intimate partner violence: a systematic review and meta-analysis. Trauma, Violence, & Abuse. 2017 Apr;18(2):155-71. | Systematic review with no new useable data and any meta-analysis results not appropriate to extract |
| 18 | Augedal 2013 | Augedal AW, Hansen KS, Kronhaug CR, Harvey AG, Pallesen S. Randomized controlled trials of psychological and pharmacological treatments for nightmares: A meta-analysis. Sleep Medicine Reviews. 2013 Apr 30;17(2):143-52. | Systematic review with no new useable data and any meta-analysis results not appropriate to extract |
| 19 | Back 2011 | Back, S. Integrated Treatment of OEF/OIF Veterans With PTSD & Substance Use Disorders (COPE). NCT01338506. 2011. Available from: https://clinicaltrials.gov/ct2/show/NCT01338506 [accessed 26.07.2017] | Unpublished (registered on clinical trials.gov and author contacted for full trial report but not provided) |
| 20 | Badour 2017 | Badour CL, Flanagan JC, Gros DF, Killeen T, Pericot-Valverde I, Korte KJ, Allan NP, Back SE. Habituation of distress and craving during treatment as predictors of change in PTSD symptoms and substance use severity. Journal of consulting and clinical psychology. 2017 Mar;85(3):274. | Subgroup/secondary analysis that is not relevant |
| 21 | Badura-Brack 2018 | Badura-Brack A, McDermott TJ, Becker KM, Ryan TJ, Khanna MM, Pine DS, Bar-Haim Y, Heinrichs-Graham E, Wilson TW. Attention training modulates resting-state neurophysiological abnormalities in posttraumatic stress disorder. Psychiatry Research: Neuroimaging. 2018 Jan 30;271:135-41. | Subgroup/secondary analysis of RCT already included |
| 22 | Banerjee 2007 | Banerjee, B., Vadiraj, H. S., Ram, A., Rao, R., Jayapal, M., Gopinath, K. S., Ramesh, B. S., Rao, N., Kumar, A., Raghuram, N., Hegde, S., Nagendra, H. R., Prakash Hande, M. (2007) Effects of an integrated yoga program in modulating psychological stress and radiation-induced genotoxic stress in breast cancer patients undergoing radiotherapy, Integrative Cancer Therapies, 6, 242-250 | Intervention not targeted at PTSD symptoms |
| 23 | Banks 2015 | Banks K, Newman E, Saleem J. An overview of the research on mindfulness‐based interventions for treating symptoms of posttraumatic stress disorder: A systematic review. Journal of clinical psychology. 2015 Oct 1;71(10):935-63. | Systematic review with no new useable data and any meta-analysis results not appropriate to extract |
| 24 | Banos 2011 | Baños RM, Guillen V, Quero S, Garcia-Palacios A, Alcaniz M, Botella C. A virtual reality system for the treatment of stress-related disorders: A preliminary analysis of efficacy compared to a standard cognitive behavioral program. International Journal of Human-Computer Studies. 2011 Aug 31;69(9):602-13. | Intervention not targeted at PTSD symptoms |
| 25 | Barabasz 2013 | Barabasz A, Barabasz M, Christensen C, French B, Watkins JG. Efficacy of single-session abreactive ego state therapy for combat stress injury, PTSD, and ASD. International Journal of Clinical and Experimental Hypnosis. 2013 Jan 1;61(1):1-9. | Non-randomised group assignment |
| 26 | Barrera 2013 | Barrera, TL.; Mott, JM.; Hofstein, RF.; Teng, EJ.; (2013) A meta-analytic review of exposure in group cognitive behavioral therapy for posttraumatic stress disorder. Clin Psych Rev 33 (1): 24-32 | Systematic review with no new useable data and any meta-analysis results not appropriate to extract |
| 27 | Barton 2014 | Barton, S.; Karner, C.; Salih, F.; Baldwin, DS.; Edwards, SJ.; (2014) Clinical effectiveness of interventions for treatment-resisitant anxiety in older people: a systematic review. Health Tech Ass 18 (50): 1366-5278 | Systematic review with no new useable data and any meta-analysis results not appropriate to extract |
| 28 | Basoglu (unpublished) | Basoglu, M., Salcioglu, E., Livanou, M., Kalender, D., Acar, G. Single-session behavioral treatment of earthquake-related posttraumatic stress disorder: A randomized waitlist controlled trial. Journal of Traumatic Stress (in press). | Paper unavailable |
| 29 | Basoglu 2003 | Basoglu, M., Livanou, M., Salcioglu, E., & Kalender, D. (2003). A brief behavioural treatment of chronic post-traumatic stress disorder in earthquake survivors: results from an open clinical trial. Psychol.Med, 33, 647-654. | Non-RCT (no control group) |
| 30 | Battersby 2013 | Battersby MW, Beattie J, Pols RG, Smith DP, Condon J, Blunden S. A randomised controlled trial of the Flinders Program™ of chronic condition management in Vietnam veterans with co-morbid alcohol misuse, and psychiatric and medical conditions. Australian & New Zealand Journal of Psychiatry. 2013 May;47(5):451-62. | Population not relevant for this review (to be considered for other relevant RQ) |
| 31 | Bean 2017 | Bean RC, Ong CW, Lee J, Twohig MP. Acceptance and commitment therapy for PTSD and trauma: An empirical review. The Behavior Therapist. 2017;4,145-150. | Systematic review with no new useable data and any meta-analysis results not appropriate to extract |
| 32 | Beatty 2016 | Beatty L, Koczwara B, Wade T. Evaluating the efficacy of a self-guided Web-based CBT intervention for reducing cancer-distress: a randomised controlled trial. Supportive Care in Cancer. 2016 Mar 1;24(3):1043-51. | Comparison outside protocol |
| 33 | Beidel 2011 | Beidel DC, Frueh BC, Uhde TW, Wong N, Mentrikoski JM. Multicomponent behavioral treatment for chronic combat-related posttraumatic stress disorder: A randomized controlled trial. Journal of anxiety disorders. 2011 Mar 31;25(2):224-31. | Comparison outside protocol |
| 34 | Beidel 2017 | Beidel DC, Frueh BC, Neer SM, Bowers CA, Trachik B, Uhde TW, Grubaugh A. Trauma management therapy with virtual-reality augmented exposure therapy for combat-related PTSD: A randomized controlled trial. Journal of anxiety disorders. 2017 Aug 23. | Comparison outside protocol |
| 35 | Bekker 2007 | Bekker, MHJ.; van Mens-Verhulst J.; (2007) Anxiety Disorders: Sex Differences in Prevalence, Degree and Background, But Gender-Neutral Treatment. Gender Med 4 (S2): S178-S193. | Systematic review with no new useable data and any meta-analysis results not appropriate to extract |
| 36 | Belleau 2017 | Belleau EL, Chin EG, Wanklyn SG, Zambrano-Vazquez L, Schumacher JA, Coffey SF. Pre-treatment predictors of dropout from prolonged exposure therapy in patients with chronic posttraumatic stress disorder and comorbid substance use disorders. Behaviour Research and Therapy. 2017 Apr 30;91:43-50. | Efficacy or safety data cannot be extracted |
| 37 | Benish 2008 | Benish, SG.; Imel, ZE.; Wampold, BE.; (2008) The relative efficacy of bona fide psychotherapies for treating post-traumatic stress disorder: A meta-analysis of direct comparisons. | Systematic review with no new useable data and any meta-analysis results not appropriate to extract |
| 38 | Bergen-Cico 2014 | Bergen-Cico D, Possemato K, Pigeon W. Reductions in cortisol associated with primary care brief mindfulness program for veterans with PTSD. Medical Care. 2014 Dec 1;52:S25-31. | Outcomes are not of interest |
| 39 | Berlim 2014 | Berlim, MT.; Wan den Eynde, F.; (2014) Repetitive Transcranial Magnetic Stimulation over the Dorsolateral Prefrontal Cortex for Treating Posttraumatic Stress Disorder: An Exploratory Meta-Analysis of Randomized Double-Blind and Sham-Controlled Trials. The Canadian J of Psychiartry 59 (9) | Systematic review with no new useable data and any meta-analysis results not appropriate to extract |
| 40 | Bichescu 2007 | Bichescu D, Neuner F, Schauer M, Elbert T. Narrative exposure therapy for political imprisonment-related chronic posttraumatic stress disorder and depression. Behaviour research and therapy. 2007 Sep 30;45(9):2212-20. | Sample size (N<10/arm) |
| 41 | Bisson 2005 | Bisson, J.; Andrew,; Psychological treatment of post-traumatic stress disorder (PTSD) (2007)Cochrane Database of Systematic Reviews | Systematic review with no new useable data and any meta-analysis results not appropriate to extract |
| 42 | Bisson 2007 | Bisson, JI.; Ehlers, A.; Matthews, R.; Pilling, S.; Richards, D.; Turner, S.; (2007) Psychological treatments for chronic post-traumatic stress disorder. Systematic review and meta-analysis. British J Psych 190: 97-104 | Systematic review with no new useable data and any meta-analysis results not appropriate to extract |
| 43 | Bisson 2013 | Bisson, J.; Roberts, NP.; Andre, M.; Cooper, R.; Lewis, C.; (2013). Psychological therapies for chronic post-traumatice stress disorder (PTSD) in adults. Cochrane Database of Systematic Reviews | Systematic review with no new useable data and any meta-analysis results not appropriate to extract |
| 44 | Boals 2016 | Boals A, Murrell AR. I am> trauma: Experimentally reducing event centrality and PTSD symptoms in a clinical trial. Journal of Loss and Trauma. 2016 Nov 1;21(6):471-83. | Non-randomised group assignment |
| 45 | Boccia 2015 | Boccia, M.; Piccardi, L.; Cordellieri, P.; Guariglia, C.; Giannini, AM.; (2015) EMDR therapy for PTSD after motor vehicle accidents: meta-analytic evidence for specific treatment. Front Hum Neurosci 9: 213 | Systematic review with no new useable data and any meta-analysis results not appropriate to extract |
| 46 | Boden 2012/2014 | Boden MT, Kimerling R, Jacobs‐Lentz J, Bowman D, Weaver C, Carney D, Walser R, Trafton JA. Seeking Safety treatment for male veterans with a substance use disorder and post‐traumatic stress disorder symptomatology. Addiction. 2012 Mar 1;107(3):578-86.  Boden MT, Kimerling R, Kulkarni M, Bonn-Miller MO, Weaver C, Trafton J. Coping among military veterans with PTSD in substance use disorder treatment. Journal of substance abuse treatment. 2014 Aug 31;47(2):160-7. | Non-randomised group assignment |
| 47 | Boggio 2010 | Boggio PS, Rocha M, Oliveira MO, Fecteau S, Cohen RB, Campanhã C, Ferreira-Santos E, Meleiro A, Corchs F, Zaghi S, Pascual-Leone A. Noninvasive brain stimulation with high-frequency and low-intensity repetitive transcranial magnetic stimulation treatment for posttraumatic stress disorder. The Journal of clinical psychiatry. 2010 Aug;71(8):992. | Efficacy or safety data cannot be extracted |
| 48 | Bolton 2015 | Bolton, AJ.; Dorstyn, DS.; (2015) Telepsychology for Posttraumatic Stress Disorder: A Systematic reivew. J Telemedicine and Telecare 21 (5) | Systematic review with no new useable data and any meta-analysis results not appropriate to extract |
| 49 | Bomyea 2015 | Bomyea J, Stein MB, Lang AJ. Interference control training for PTSD: A randomized controlled trial of a novel computer-based intervention. Journal of anxiety disorders. 2015 Aug 31;34:33-42. | Comparison outside protocol |
| 50 | Bomyea 2017 | Bomyea J, Lang AJ, Schnurr PP. TBI and Treatment Response in a Randomized Trial of Acceptance and Commitment Therapy. The Journal of head trauma rehabilitation. 2017 Jan. | Intervention not targeted at PTSD symptoms |
| 51 | Bordow 1979 | Bordow, S. & Porritt, D. (1979). An experimental evaluation of crisis intervention. Social Science & Medicine, 13A, 251-256. | Non-randomised group assignment |
| 52 | Boritz 2016 | Boritz T, Barnhart R, McMain SF. The influence of posttraumatic stress disorder on treatment outcomes of patients with borderline personality disorder. Journal of personality disorders. 2016 Jun;30(3):395-407. | Intervention not targeted at PTSD symptoms |
| 53 | Bottche 2016 | Böttche M, Kuwert P, Pietrzak RH, Knaevelsrud C. Predictors of outcome of an Internet‐based cognitive‐behavioural therapy for post‐traumatic stress disorder in older adults. Psychology and Psychotherapy: Theory, Research and Practice. 2016 Mar 1;89(1):82-96. | Subgroup/secondary analysis of RCT already included |
| 54 | Boudewyns 1990 | Boudewyns, P.A.; Hyer, L. (1990) Physiological response to combat memories and preliminary treatment outcome in Vietnam veteren PTSD patients treated with direct therapeutic exposure. Behavior Therapy, 21, 63-87 | Intervention not targeted at PTSD symptoms |
| 55 | Bowland 2012 | Bowland S, Edmond T, Fallot RD. Evaluation of a spiritually focused intervention with older trauma survivors. Social work. 2012 Jan 1;57(1):73-82. | Intervention not targeted at PTSD symptoms |
| 56 | Bradley 2003 | Bradley, RG.; Follingstad DR.; (2003) Group Therapy for Incarcerated Women Who Experienced Interpersonal Violence: A Pilot Study. J Trau Stress 16(4):337-340 | Population outside scope: Trials of adults in contact with the criminal justice system (not solely as a result of being a witness or victim) |
| 57 | Bradley 2005 | Bradley, R.; Greene, J.; Russ, E.; Dutra, L.; Westen, D.; (2005) A Multidimensional Meta-Analysis of Psychotherapy for PTSD. Am J Psych 162 (2): 214-227 | Systematic review with no new useable data and any meta-analysis results not appropriate to extract |
| 58 | Bradshaw 2014 | Bradshaw RA, McDonald MJ, Grace R, Detwiler L, Austin K. A randomized clinical trial of Observed and Experiential Integration (OEI): A simple, innovative intervention for affect regulation in clients with PTSD. Traumatology. 2014 Sep;20(3):161. | Sample size (N<10/arm) |
| 59 | Bremner 2017 | Bremner JD, Mishra S, Campanella C, Shah M, Kasher N, Evans S, Fani N, Shah AJ, Reiff C, Davis LL, Vaccarino V and Carmody J (2017) A Pilot Study of the Effects of Mindfulness-Based Stress Reduction on Post-traumatic Stress Disorder Symptoms and Brain Response to Traumatic Reminders of Combat in Operation Enduring Freedom/Operation Iraqi Freedom Combat Veterans with Post-traumatic Stress Disorder. Front. Psychiatry 8:157. doi: 10.3389/fpsyt.2017.00157 | Sample size (N<10/arm) |
| 60 | Brief 2013 | Brief DJ, Rubin A, Keane TM, Enggasser JL, Roy M, Helmuth E, Hermos J, Lachowicz M, Rybin D, Rosenbloom D. Web intervention for OEF/OIF veterans with problem drinking and PTSD symptoms: A randomized clinical trial. Journal of consulting and clinical psychology. 2013 Oct;81(5):890. | Intervention not targeted at PTSD symptoms |
| 61 | Brown 2013 | Brown LA, Craske MG, Glenn DE, Stein MB, Sullivan G, Sherbourne C, Bystritsky A, Welch SS, Campbell‐Sills L, Lang A, Roy‐Byrne P. CBT competence in novice therapists improves anxiety outcomes. Depression and anxiety. 2013 Feb 1;30(2):97-115. | Intervention not targeted at PTSD symptoms |
| 62 | Brown 2014 | Brown AJ, Bollini AM, Craighead LW, Astin MC, Norrholm SD, Bradley B. Self‐Monitoring of Reexperiencing Symptoms: A Randomized Trial. Journal of traumatic stress. 2014 Oct 1;27(5):519-25. | Efficacy or safety data cannot be extracted |
| 63 | Bryant 2008b | Bryant RA, Moulds ML, Guthrie RM, Dang ST, Mastrodomenico J, Nixon RD, Felmingham KL, Hopwood S, Creamer M. A randomized controlled trial of exposure therapy and cognitive restructuring for posttraumatic stress disorder. Journal of consulting and clinical psychology. 2008 Aug;76(4):695. | Comparison outside protocol |
| 64 | Bryant 2013 | Bryant RA, Mastrodomenico J, Hopwood S, Kenny L, Cahill C, Kandris E, Taylor K. Augmenting cognitive behaviour therapy for post-traumatic stress disorder with emotion tolerance training: a randomized controlled trial. FOCUS. 2013 Jul;11(3):379-86. | Paper unavailable |
| 65 | Butollo 2016 | Butollo W, Karl R, König J, Rosner R. A Randomized Controlled Clinical Trial of Dialogical Exposure Therapy versus Cognitive Processing Therapy for Adult Outpatients Suffering from PTSD after Type I Trauma in Adulthood. Psychotherapy and psychosomatics. 2016;85(1):16-26. | Comparison outside protocol |
| 66 | Cabral 2011 | Cabral, P.; Meyer, HB.; Ames, D.; (2011) Effectiveness of Yoga Therapy as a Complementary Treatment for Major Psychiatric Disorders: A Meta-Analysis . Primary Care Companion for CNS Disorders 13 (4) | Systematic review with no new useable data and any meta-analysis results not appropriate to extract |
| 67 | Carlson 2013/2016 | Carlson LE, Doll R, Stephen J, Faris P, Tamagawa R, Drysdale E, Speca M. Randomized controlled trial of mindfulness-based cancer recovery versus supportive expressive group therapy for distressed survivors of breast cancer (MINDSET). Journal of clinical oncology. 2013 Aug 5;31(25):3119-26.  Carlson LE, Tamagawa R, Stephen J, Drysdale E, Zhong L, Speca M. Randomized‐controlled trial of mindfulness‐based cancer recovery versus supportive expressive group therapy among distressed breast cancer survivors (MINDSET): long‐term follow‐up results. Psycho‐Oncology. 2016 Jul 1;25(7):750-9. | Intervention not targeted at PTSD symptoms |
| 68 | Carlson 2014 | Carlson, L.E., Tamagawa, R., Stephen, J., Doll, R., Faris, P., Dirkse, D. and Speca, M., 2014. Tailoring mind-body therapies to individual needs: patients’ program preference and psychological traits as moderators of the effects of mindfulness-based cancer recovery and supportive-expressive therapy in distressed breast cancer survivors. Journal of the National Cancer Institute Monographs, 2014(50), pp.308-314. | Subgroup/secondary analysis that is not relevant |
| 69 | Carpenter 2014 | Carpenter KM, Stoner SA, Schmitz K, McGregor BA, Doorenbos AZ. An online stress management workbook for breast cancer. Journal of behavioral medicine. 2014 Jun 1;37(3):458-68. | Efficacy or safety data cannot be extracted |
| 70 | Carter 2006b | Carter JJ. A controlled breathing course promoting social and emotional health for Vietnam veterans with chronic posttraumatic stress disorder - A randomised controlled trial [NCT00256477]. 2006. Available from: https://clinicaltrials.gov/ct2/show/NCT00256477 [accessed 28.07.2017] | Paper unavailable |
| 71 | Carter 2006a | Carter J, Byrne G. A two year study of the use of yoga in a series of pilot studies as an adjunct to ordinary psychiatric treatment in a group of Vietnam War veterans suffering from post traumatic stress disorder. Online document at: www. Therapywithyoga. com Accessed November. 2004;27. | Design: Non-randomised group assignment |
| 72 | Carter 2013 | Carter J, Gerbarg PL, Brown RP, Ware RS, D’Ambrosio C. Multi-component yoga breath program for Vietnam veteran post traumatic stress disorder: randomized controlled trial. J Trauma Stress Disor Treat 2. 2013;3:2. | Efficacy or safety data cannot be extracted |
| 73 | Casement 2012 | Casement, MD.; Swanson, LM.; (2012) A meta-analysis of imagery rehearsal for post-traumatic nightmares: Effects on nightmare frequency, sleep quality and posttraumatic stress. Clinical Psychology Review. 32 (6): 566-574 | Systematic review with no new useable data and any meta-analysis results not appropriate to extract |
| 74 | Chemtob 1997b | Chemtob, C. M., Novaco, R. W., Hamada, R. S., & Gross, D. M. (1997). Cognitive-behavioral treatment for severe anger in posttraumatic stress disorder. Journal of Consulting & Clinical Psychology, 65, 184-189 | Sample size (N<10/arm) |
| 75 | Chen 2014 | Chen, Y-R.; Hung, K-W.; Tsai, J-C.; Chu, H.; Chung, M-H.; Chen, S-R.; Liao, Y-M.; Ou, K-L.; Chang, Y-C.; Chou, K-R.; (2014) Efficacy of Eye-Movement Desensitization and Reprocessing for patients with Posttraumatic-Stress Disorder: A Meta-Analysis of Randomized Controlled Trials. PLOS-One 9 (8) | Systematic review with no new useable data and any meta-analysis results not appropriate to extract |
| 76 | Chen 2015 | Chen, L.; Zhang, G.; Hu M.; Liang, X.; (2015) Eye Movement Desensitization and Reprocessing Versus Cognitive-Behavioural Therapy for Adult Posttraumatic Stress Disorder: Systematic Review and Meta-Analysis. J of Nervous and Mental Disease. 203 (6):443-451 | Systematic review with no new useable data and any meta-analysis results not appropriate to extract |
| 77 | Chiesa 2010 | Chiesa, A.; (2010) Vipassana Meditation: Systematic Review of Current Evidence. The Jornal of Alternative and Complementary Medicine 16 (1): 37-46 | Systematic review with no new useable data and any meta-analysis results not appropriate to extract |
| 78 | Christensen 2013 | Christensen C, Barabasz A, Barabasz M. Efficacy of abreactive ego state therapy for PTSD: Trauma resolution, depression, and anxiety. International Journal of Clinical and Experimental Hypnosis. 2013 Jan 1;61(1):20-37. | Efficacy or safety data cannot be extracted |
| 79 | Church 2016b | Church D, Yount G, Rachlin K, Fox L, Nelms J. Epigenetic Effects of PTSD Remediation in Veterans Using Clinical Emotional Freedom Techniques: A Randomized Controlled Pilot Study. American Journal of Health Promotion. 2016 Aug 12:0890117116661154. | Sample size (N<10/arm) |
| 80 | Cimpianu 2017 | Cimpianu, C-L.; Strube, W.; Falkai, P.; Palm, U.; Hasan, A.; (2017) Vagus nerve stimulation in psychiarty: a systematic review of the available evidence. J Nerual Transmission 124 (1): 145-158 | Systematic review with no new useable data and any meta-analysis results not appropriate to extract |
| 81 | Clarke 2008 | Clarke SB, Rizvi SL, Resick PA. Borderline personality characteristics and treatment outcome in cognitive-behavioral treatments for PTSD in female rape victims. Behavior therapy. 2008 Mar 31;39(1):72-8. | Subgroup/secondary analysis of RCT already included |
| 82 | Classen 2001 | Classen, C., Koopman, C., Nevill-Manning, K., & Spiegel, D. (2001). A preliminary report comparing trauma-focused and present-focused group therapy against a wait-listed condition among childhood sexual abuse survivors with PTSD. Journal of Aggression, Maltreatment & Trauma, 4, 265-288. | Efficacy or safety data cannot be extracted |
| 83 | Clausen 2012 | Clausen, J., Ruff, S., Von Wiederhold, W., Heineman, T. (2012) For as long as it takes: Relationship-based play therapy for children in foster care, Psychoanalytic Social Work, 19, 43-53 | Non-RCT (no control group) |
| 84 | Cloitre 2012 | Cloitre M, Petkova E, Wang J. An examination of the influence of a sequential treatment on the course and impact of dissociation among women with PTSD related to childhood abuse. Depression and Anxiety. 2012 Aug 1;29(8):709-17. | Subgroup/secondary analysis of RCT already included |
| 85 | Cloitre 2017 | Cloitre M, Garvert DW, Weiss BJ. Depression as a moderator of STAIR Narrative Therapy for women with post-traumatic stress disorder related to childhood abuse. European journal of psychotraumatology. 2017 Jan 1;8(1):1377028. | Subgroup/secondary analysis of RCT already included |
| 86 | Clond 2016 | Clond, M.; (2016) Emotional Freedom Techniques for Anxiety: A Systematic Review With Meta-analysis. J of Nervous and Mental disease 204 (5):388-395 | Systematic review with no new useable data and any meta-analysis results not appropriate to extract |
| 87 | Connolly 2013 | Connolly SM, Roe-Sepowitz D, Sakai C, Edwards J. Utilizing community resources to treat PTSD: A randomized controlled study using Thought Field Therapy. African J Trauma Studies. 2013;3:24-32. | Non-randomised group assignment |
| 88 | Coffey 2006 | Coffey SF, Stasiewicz PR, Hughes PM, Brimo ML. Trauma-focused imaginal exposure for individuals with comorbid posttraumatic stress disorder and alcohol dependence: Revealing mechanisms of alcohol craving in a cue reactivity paradigm. Psychology of Addictive Behaviors. 2006 Dec;20(4):425. | Sample size (N<10/arm) |
| 89 | Cohen 2004b | Cohen, H., Kaplan, Z., Kotler, M., Kouperman, I., Moisa, R., & Grisaru, N. (2004). Repetitive transcranial magnetic stimulation of the right dorsolateral prefrontal cortex in posttraumatic stress disorder: a double-blind, placebo-controlled study. American Journal of Psychiatry, 161(3), 515-524. | Sample size (N<10/arm) |
| 90 | Cook 2013 | Cook JM, Thompson R, Harb GC, Ross RJ. Cognitive− behavioral treatment for posttraumatic nightmares: An investigation of predictors of dropout and outcome. Psychological Trauma: Theory, Research, Practice, and Policy. 2013 Nov;5(6):545. | Subgroup/secondary analysis that is not relevant |
| 91 | Cooper 1989 | Cooper, N.A.; Clum, G.A. (1989) Imaginal flooding as a supplimentary treatment for PTSD in combat veterens: a controlled study. Behavior Therapy, 20, 381-391 | Sample size (N<10/arm) |
| 92 | Cooper 2017a | Cooper AA, Kline AC, Graham B, Bedard-Gilligan M, Mello PG, Feeny NC, Zoellner LA. Homework “dose,” type, and helpfulness as predictors of clinical outcomes in prolonged exposure for PTSD. Behavior therapy. 2017 Mar 1;48(2):182-94. | Subgroup/secondary analysis that is not relevant |
| 93 | Cooper 2017b | Cooper AA, Zoellner LA, Roy-Byrne P, Mavissakalian MR, Feeny NC. Do changes in trauma-related beliefs predict PTSD symptom improvement in prolonged exposure and sertraline?. Journal of consulting and clinical psychology. 2017 Sep;85(9):873. | Subgroup/secondary analysis that is not relevant |
| 94 | Cort 2012 | Cort NA, Gamble SA, Smith PN, Chaudron LH, Lu N, He H, Talbot NL. Predictors of treatment outcomes among depressed women with childhood sexual abuse histories. Depression and anxiety. 2012 Jun 1;29(6):479-86. | Subgroup/secondary analysis of RCT already included |
| 95 | Craft 2013 | Craft MA, Davis GC, Paulson RM. Expressive writing in early breast cancer survivors. Journal of Advanced Nursing. 2013 Feb 1;69(2):305-15. | Intervention not targeted at PTSD symptoms |
| 96 | Craske 2011 | Craske MG, Stein MB, Sullivan G, Sherbourne C, Bystritsky A, Rose RD, Lang AJ, Welch S, Campbell-Sills L, Golinelli D, Roy-Byrne P. Disorder-specific impact of coordinated anxiety learning and management treatment for anxiety disorders in primary care. Archives of General Psychiatry. 2011 Apr 4;68(4):378-88. | Intervention outside protocol |
| 97 | Crawford 2016 | Crawford JJ, Vallance JK, Holt NL, Steed H, Courneya KS. A phase I/II pilot study assessing the preliminary efficacy of wall climbing for improving posttraumatic growth and quality of life in gynecologic cancer survivors. Mental Health and Physical Activity. 2016 Oct 31;11:60-6. | Outcomes are not of interest |
| 98 | Crespo 2010 | Crespo M, Arinero M. Assessment of the efficacy of a psychological treatment for women victims of violence by their intimate male partner. The Spanish journal of psychology. 2010 Nov;13(2):849-63. | Non-randomised group assignment |
| 99 | Crumlish 2010 | Crumlish, N.; O'Rourke, K.; (2010) A systematic review of treatments for post-traumatic stress disorder among refugees and asylum-seekers. J Nervous and Mental Disease 198 (4): 237-251 | Systematic review with no new useable data and any meta-analysis results not appropriate to extract |
| 100 | Cuijpers 2009 | Cuijpers, P.; Marks, IM.; Van Straten, A.; Cavanagh, K.; Gega, L.; Andersson, G.; (2009) Computer-Aided Psychotherapy for Anxiety Disorders: A Meta-Analytic Review. Cog Beh Therapy 38(2): 66-82 | Systematic review with no new useable data and any meta-analysis results not appropriate to extract |
| 101 | Cuijpers 2013 | Cuijpers, P.; Sijbrandij, M.; Koole, SL.; Andersson, G.; Beekman, AT.; Reynolds, CF.; (2013) The efficacy of psychotherapy and pharmacotherapy in treating depressive and anxiety disorders: a meta-analysis of direct comparisons. World Psychiatry 12 (2): 137-148 | Systematic review with no new useable data and any meta-analysis results not appropriate to extract |
| 102 | Cusack 1999 | Cusack, K. & Spates, C. R. (1999). The cognitive dismantling of Eye Movement Desensitization and Reprocessing (EMDR) treatment of Posttraumatic Stress Disorder (PTSD). Journal of Anxiety Disorders, 13, 87-99. | Non-randomised group assignment |
| 103 | Cusack 2016 | Cusack, K.; Jonas, DE.; Forneris, CA.; Wines, C.; Sonis, J.; Middleton, JC.; Feltner, C.; Brownley, KA.; Olmsted, KR.; Greenblatt, A.; Weil, A.; Gaynes, BN.; (2016) Psychological treatments for adults with posttraumatic stress disorder: A systematic review and meta-analysis. Clin Pscy Rev 43: 128-141 | Systematic review with no new useable data and any meta-analysis results not appropriate to extract |
| 104 | Cyniak-Cieciura 2015 | Cyniak-Cieciura M, Popiel A, Zawadzki B. General self-efficacy level and changes in negative postttraumatic cognitions and posttraumatic stress disorder (PTSD) symptoms among motor vehicle accident survivors after PTSD therapy. Psychol Stud. 2015;53:18-29. | Subgroup/secondary analysis of RCT already included |
| 105 | Da Silva | Da Silva, TL.; Ravindran, LN.; Ravindran, AV.; (2009) Yoga in the treatment of mood and anxiety disorders: A review. Asian J Psychiatry 2 (1): 6-16 | Systematic review with no new useable data and any meta-analysis results not appropriate to extract |
| 106 | Dalton 2013 | Dalton EJ, Greenman PS, Classen CC, Johnson SM. Nurturing connections in the aftermath of childhood trauma: A randomized controlled trial of emotionally focused couple therapy for female survivors of childhood abuse. Couple and Family Psychology: Research and Practice. 2013 Sep;2(3):209. | Efficacy or safety data cannot be extracted |
| 107 | Deacon 2004 | Deacon, BJ.; Abramowitz, JS.; (2004) Cognitive and behavioral treatments for anxiety disorders: A review of meta-analytic findings. J Clin Psyh 60 (4): 429-441 | Systematic review with no new useable data and any meta-analysis results not appropriate to extract |
| 108 | Detweiler 2015 | Detweiler MB, Lane S, Spencer L, Lutgens B, Halling MH, Rudder TF, Lehmann L. Horticultural therapy: A pilot study on modulating cortisol levels and indices of substance craving, posttraumatic stress disorder, depression, and quality of life in veterans. Alternative therapies in health and medicine. 2015 Jul 1;21(4):36. | Sample size (N<10/arm) |
| 109 | Devilly 1998 | Devilly, G. J., Spence, S. H., & Rapee, R. M. (1998). Statistical and reliable change with eye movement desensitization and reprocessing: Treating trauma within a veteran population. Behavior Therapy, 29, 435-455. | Non-randomised group assignment |
| 110 | Devilly 1999 | Devilly GJ, Spence SH. The relative efficacy and treatment distress of EMDR and a cognitive-behavior trauma treatment protocol in the amelioration of posttraumatic stress disorder. Journal of anxiety disorders. 1999 Apr 30;13(1):131-57. | Non-randomised group assignment |
| 111 | Devilly 2001 | Devilly GJ. The successful treatment of PTSD through overt cognitive behavioral therapy in non-responders to EMDR. Behavioural and Cognitive Psychotherapy. 2001 Jan;29(1):57-70. | Non-RCT (no control group) |
| 112 | Diehle 2014 | Diehle, J.; Schmitt, K.; Daams, JG.; Boer, F.; Lindauer, RJL.; (2014) Effects of Psychotherapy on Trauma-Related Cognitions in Posttraumatic Stress Disorder: A Meta-Analysis. J Traumatic Stress 27 (3): 257-264 | Systematic review with no new useable data and any meta-analysis results not appropriate to extract |
| 113 | Difede 2007a | Difede J, Cukor J, Jayasinghe N, Patt I, Jedel S, Spielman L, Giosan C, Hoffman HG. Virtual reality exposure therapy for the treatment of posttraumatic stress disorder following September 11, 2001. Journal of Clinical Psychiatry. 2007 Nov 11;68(11):1639. | Sample size (N<10/arm) |
| 114 | DiMauro 2014 | DiMauro, J.; (2014) Exposure Therapy for Posttraumatic Stress Disorder: A Meta-Analysis. Military Psychology 26(2):120-130 | Systematic review with no new useable data and any meta-analysis results not appropriate to extract |
| 115 | Dinnen 2014 | Dinnen, S.; Simiola, V.; Cook, JM.; (2014) Post-traumatic stress disorder in older adults: a systematic review of the psychotherapy treatment literature. Aging and Mental Health 19 (2): 144-150 | Systematic review with no new useable data and any meta-analysis results not appropriate to extract |
| 116 | Dodds 2015 | Dodds SE, Pace TW, Bell ML, Fiero M, Negi LT, Raison CL, Weihs KL. Feasibility of Cognitively-Based Compassion Training (CBCT) for breast cancer survivors: a randomized, wait list controlled pilot study. Supportive Care in Cancer. 2015 Dec 1;23(12):3599-608. | Efficacy or safety data cannot be extracted |
| 117 | Dorrepaal 2010 | Dorrepaal E, Thomaes K, Smit JH, van Balkom AJ, van Dyck R, Veltman DJ, Draijer N. Stabilizing group treatment for complex posttraumatic stress disorder related to childhood abuse based on psycho-education and cognitive behavioral therapy: A pilot study. Child Abuse & Neglect. 2010 Apr 30;34(4):284-8. | Non-RCT (no control group) |
| 118 | Dorrepaal 2013 | Dorrepaal E, Thomaes K, Smit JH, Veltman DJ, Hoogendoorn AW, van Balkom AJ, Draijer N. Treatment compliance and effectiveness in complex PTSD patients with co-morbid personality disorder undergoing stabilizing cognitive behavioral group treatment: A preliminary study. European journal of psychotraumatology. 2013 Dec 1;4(1):21171. | Subgroup/secondary analysis of RCT already included |
| 119 | Dorrepaal 2014 | Dorrepaal, E.; Thomaes, K.; Hoogendoorn, AW.; Veltman, DJ.; Drijer, N.; Van Balkom, AJLM.; (2014) Evidence-based treatment for adult women with child abouse-related Complex PTSD: a quantitative review. Eur J Psychotraumatology 5(1): | Systematic review with no new useable data and any meta-analysis results not appropriate to extract |
| 120 | Dossa 2012 | Dossa, NI.; Hatem, M.; (2012) Cognitive-Behavioral Therapy versus Other PTSD Psychotherapies as Treatment for Women Victims of War-Related Violence: A Systematic Review. The Scientific World Journal:ID, 181847 | Systematic review with no new useable data and any meta-analysis results not appropriate to extract |
| 121 | Drožđek 2010 | Drožđek B, Bolwerk N. Evaluation of group therapy with traumatized asylum seekers and refugees—The Den Bosch Model. Traumatology. 2010 Dec;16(4):117. | Non-randomised group assignment |
| 122 | Drožđek 2012 | Droždek B, Kamperman AM, Bolwerk N, Tol WA, Kleber RJ. Group therapy with male asylum seekers and refugees with posttraumatic stress disorder: A controlled comparison cohort study of three day-treatment programs. The Journal of nervous and mental disease. 2012 Sep 1;200(9):758-65. | Non-randomised group assignment |
| 123 | Drummond 2009 | Drummond SP. Treating Insomnia & Nightmares After Trauma: Impact on Symptoms & Quality of Life [NCT01009112]. Available from: https://clinicaltrials.gov/ct2/show/NCT01009112 [accessed 08.08.2017] | Comparison outside protocol |
| 124 | Duan-Porter 2016 | Duan-Porter, W.; Coeytaux, RR.; McDuffie, JR.; Goode, AP.; Sharma, P.; Mennella, H.; Nagi, A.; Williams, JW.; (2016) Evidence Map of Yoga for Depression, Anxiety and Posttraumatic Stress Disorder. J Phsyical Activity Health 13: 281-288 | Systematic review with no new useable data and any meta-analysis results not appropriate to extract |
| 125 | Dybdahl 2001 | Dybdahl, R. (2001) Children and mothers in war: an outcome study of a psychosocial intervention program. Child Development, 72, 4, 1214-1230 | Efficacy or safety data cannot be extracted |
| 126 | Echeburua 1996 | Echeburua, E; Corral, P.; Sarasua, B; Zubizarreta, I. (1996) Treatment of acute posttraumatic stress disorder in rape victims: an experimental study. Journal of Anxiety Disorders, 10, 3, 185-199 | Non-randomised group assignment |
| 127 | Echeburua 1997 | Echeburua, E., de Corral, P., Zubizarreta, I., & Sarasua, B. (1997). Psychological treatment of chronic posttraumatic stress disorder in victims of sexual aggression. Behavior Modification, 21, 433- 456. | Sample size (N<10/arm) |
| 128 | Edzard 2012 | Edzard, E.; Snyder, J.; Dunlop, RA.; (2012) National Centre for Complementary and Alternative Medicine-funded randomised controlled trials of acupuncture: a systematic review. Focus on Alternative and Complementary Therapies, 17(1):15-22. | Systematic review with no new useable data and any meta-analysis results not appropriate to extract |
| 129 | Ehring 2014 | Ehring, T.; Welboren, R.; Morina, N.; Wicherts, JM.; Freitag, J.; Emmelkamp, PMG.; (2014) Meta-analysis of psychological treatments for posttraumatic stress disorder in adult survivors of childhood abuse. Clin Pscyh Rev 34(8):645-657 | Systematic review with no new useable data and any meta-analysis results not appropriate to extract |
| 130 | Elkjaer 2014 | Elkjaer H, Kristensen E, Mortensen EL, Poulsen S, Lau M. Analytic versus systemic group therapy for women with a history of child sexual abuse: 1‐Year follow‐up of a randomized controlled trial. Psychology and Psychotherapy: Theory, Research and Practice. 2014 Jun 1;87(2):191-208. | Intervention not targeted at PTSD symptoms |
| 131 | Engel 2015 | Engel CC, Litz B, Magruder KM, Harper E, Gore K, Stein N, Yeager D, Liu X, Coe TR. Delivery of self training and education for stressful situations (DESTRESS-PC): a randomized trial of nurse assisted online self-management for PTSD in primary care. General hospital psychiatry. 2015 Aug 31;37(4):323-8. | Population outside scope: Trials of soldiers on active service |
| 132 | Erford 2016 | Erford, BT.; Gunther, C.; Duncan, K.; Bardhoshi, G.; Dummett, B.; Kraft, J.; Deferio, K.; Falco, M.; Ross, M.; (2016) Meta-Analysis of Counseling Outcomes for the Treatment of Posttraumatic Stress Disorder. J Couns Devplt 94 (1); 13-30 | Systematic review with no new useable data and any meta-analysis results not appropriate to extract |
| 133 | Erickson 2007 | Erickson DH, Janeck AS, Tallman K. A cognitive-behavioral group for patients with various anxiety disorders. Psychiatric Services. 2007 Sep;58(9):1205-11. | Population outside scope: <80% of the study's participants are eligible for the review and disaggregated data cannot be obtained |
| 134 | Falsetti 2001 | Falsetti, S.A.; Resnick, H.S. & Gallagher, N.G. (2001) Treatment of posttraumatic stress disorder with comorbid panic attacks: combining cognitive processing therapy with panic control treatment techniques. Group Dynamics: Theory, Research, and Practice, 5, 4, 252-260 | Cross-over study and first phase data not available |
| 135 | Feeny 2002 | Feeny, CC.; Zoellner, LA.; Foa, EB.; (2002) Treatment Outcome for Chronic PTSD Among Gemal Assault Victims with Borderline Personality Characteristics: A Preliminary Examination. J Personality Disorders 16 (1): 30-40 | Non-randomised group assignment |
| 136 | Feeny 2004 | NCT00127673. Effectiveness of PTSD Treatment: CBT Versus Sertraline. Available from: https://clinicaltrials.gov/show/NCT00127673 [accessed 06.01.17] | Unpublished (registered on clinical trials.gov and author contacted for full trial report but not provided) |
| 137 | Felmingham 2012 | Felmingham KL, Bryant RA. Gender differences in the maintenance of response to cognitive behavior therapy for posttraumatic stress disorder. Journal of Consulting and Clinical Psychology. 2012 Apr;80(2):196. | Comparison outside protocol |
| 138 | Fernandez 2008 | Fernández I, Páez D. The benefits of expressive writing after the Madrid terrorist attack: Implications for emotional activation and positive affect. British Journal of Health Psychology. 2008 Feb 1;13(1):31-4. | Intervention not targeted at PTSD symptoms |
| 139 | Feske 2008 | Feske U. Treating low-income and minority women with posttraumatic stress disorder: A pilot study comparing prolonged exposure and treatment as usual conducted by community therapists. Journal of interpersonal violence. 2008 Aug;23(8):1027-40. | Sample size (N<10/arm) |
| 140 | Fetzner 2015 | Fetzner MG, Asmundson GJ. Aerobic exercise reduces symptoms of posttraumatic stress disorder: A randomized controlled trial. Cognitive behaviour therapy. 2015 Jul 4;44(4):301-13. | Comparison outside protocol |
| 141 | Foa (unpublished) | Foa, E.B.; Zoellner, L.A. & Feeny, N.C. (unpublished) Recovery after trauma. | Paper unavailable |
| 142 | Foa 1999 | Foa, EB.; Dancu CV.; Hembree EX.; Joycos LH.; Meadows EA.; Street,GP.; A comparison of exposure therapy, stress incoulation training, and their combination for reducing postraumatic stress disorder in female assult victims (1999). J Consult and Clin Psy 67 (2): 194-200 | Non-randomised group assignment |
| 143 | Foa 2004 | Foa EB, Rauch SA. Cognitive changes during prolonged exposure versus prolonged exposure plus cognitive restructuring in female assault survivors with posttraumatic stress disorder. Journal of consulting and clinical psychology. 2004 Oct;72(5):879. | Outcomes are not of interest |
| 144 | Forbes 1994 | Forbes, D.; Creamer, M.; Rycroft, P. (1994) Eye movement desensitization and reprocessing in posttraumatic stress disorder: a pilot study using assessment measures. Journal of Behaviour Therapy & Experimental Psychiatry, 25, 2, 113-120 | Non-randomised group assignment |
| 145 | Forbes 2001 | Forbes, D., Phelps, A., & McHugh, T. (2001). Treatment of combat-related nightmares using imagery rehearsal: a pilot study. Journal of Traumatic Stress, 14, 433-442 | Non-randomised group assignment |
| 146 | Ford 2016 | Ford J, Rosman L, Wuensch K, Irvine J, Sears SF. Cognitive–Behavioral Treatment of Posttraumatic Stress in Patients With Implantable Cardioverter Defibrillators: Results From a Randomized Controlled Trial. Journal of traumatic stress. 2016 Aug 1;29(4):388-92. | Efficacy or safety data cannot be extracted |
| 147 | Forman 2012 | Forman EM, Shaw JA, Goetter EM, Herbert JD, Park JA, Yuen EK. Long-term follow-up of a randomized controlled trial comparing acceptance and commitment therapy and standard cognitive behavior therapy for anxiety and depression. Behavior Therapy. 2012 Dec 31;43(4):801-11. | Intervention not targeted at PTSD symptoms |
| 148 | Forshay 2011 | Forshay, E. Cognitive Behavioral Therapy (CBT) for PTSD in Veterans With Co-Occurring SUDs [NCT01357577]. Available from: https://clinicaltrials.gov/ct2/show/NCT01357577 [accessed 02.08.2017] | Protocol |
| 149 | Frank 1998b | Frank, E.; Anderson, B.; Stewart, B.D.; Dancu, C.; Hughes, C.; West, D. (1988) Efficacy of cognitive behavior therpy and systematic desensitization in the treatment of rape trauma. Behavior therapy, 19, 403-420 | Non-randomised group assignment |
| 150 | Franklin 2017 | Franklin CL, Cuccurullo LA, Walton JL, Arseneau JR, Petersen NJ. Face to face but not in the same place: A pilot study of prolonged exposure therapy. Journal of Trauma & Dissociation. 2017 Jan 1;18(1):116-30. | Sample size (N<10/arm) |
| 151 | Fredette 2016 | Fredette, C.; El-Baalbaki, G.; Palardy, V.; Rizkallah, E.; Guay, S.; (2016) Social support and cognitive-behavioral therapy for posttraumatic stress disorder: A systematic review. Traumatology 22(2): 131-144. | Systematic review with no new useable data and any meta-analysis results not appropriate to extract |
| 152 | Fredman 2016 | Fredman SJ, Pukay-Martin ND, Macdonald A, Wagner AC, Vorstenbosch V, Monson CM. Partner accommodation moderates treatment outcomes for couple therapy for posttraumatic stress disorder. Journal of consulting and clinical psychology. 2016 Jan;84(1):79. | Subgroup/secondary analysis that is not relevant |
| 153 | Frisman 2008 | Frisman L, Ford J, Lin HJ, Mallon S, Chang R. Outcomes of trauma treatment using the TARGET model. Journal of Groups in Addiction & Recovery. 2008 Nov 3;3(3-4):285-303. | Non-randomised group assignment |
| 154 | Frommberger 2004 | Frommberger U, Stieglitz RD, Nyberg E, Richter H, Novelli-Fischer U, Angenendt J, Zaninelli R, Berger M. Comparison between paroxetine and behaviour therapy in patients with posttraumatic stress disorder (PTSD): a pilot study. International Journal of Psychiatry in Clinical Practice. 2004 Jan 1;8(1):19-23. | Sample size (N<10/arm) |
| 155 | Frost 2014 | Frost, ND.; Laska, KM.; Wampold, BE.; (2014) The Evidence for Present-Centred Therapy as a Treatment for Posttraumatic Stress Disorder. J Trau Stress 27(1):1-8 | Systematic review with no new useable data and any meta-analysis results not appropriate to extract |
| 156 | Frueh 1996 | Frueh, B.C.; Turner, S.T.; Beidel, D.C.; Mirabella, R.F.; Jones, W.J. (1996) Trauma management therapy: a preliminary evaluation of a multicomponent behavioral treatment for combat-related PTSD. Behavior Research & Therapy, 34, 7, 533-543 | Non-randomised group assignment |
| 157 | Gallagher 2012 | Gallagher MW, Resick PA. Mechanisms of change in cognitive processing therapy and prolonged exposure therapy for PTSD: Preliminary evidence for the differential effects of hopelessness and habituation. Cognitive therapy and research. 2012 Dec 1;36(6):750-5. | Subgroup/secondary analysis of RCT already included |
| 158 | Gallegos 2016 | Gallegos AM, Streltzov NA, Stecker T. Improving Treatment Engagement for Returning Operation Enduring Freedom and Operation Iraqi Freedom Veterans With Posttraumatic Stress Disorder, Depression, and Suicidal Ideation. The Journal of nervous and mental disease. 2016 May 1;204(5):339-43. | Subgroup/secondary analysis of RCT already included |
| 159 | Gallegos 2017 | Gallegos AM, Crean HF, Pigeon WR, Heffner KL. Meditation and yoga for posttraumatic stress disorder: A meta-analytic review of randomized controlled trials. Clinical psychology review. 2017 Oct 31. | Systematic review with no new useable data and any meta-analysis results not appropriate to extract |
| 160 | Galovski 2009 | Galovski TE, Monson C, Bruce SE, Resick PA. Does cognitive–behavioral therapy for PTSD improve perceived health and sleep impairment?. Journal of traumatic stress. 2009 Jun 1;22(3):197-204. | Subgroup/secondary analysis of RCT already included |
| 161 | Galovski 2012 | Galovski TE, Blain LM, Mott JM, Elwood L, Houle T. Manualized therapy for PTSD: Flexing the structure of cognitive processing therapy. Journal of consulting and clinical psychology. 2012 Dec;80(6):968. | Cross-over study and first phase data not available |
| 162 | Galovski 2014 | Galovski TE, Elwood LS, Blain LM, Resick PA. Changes in anger in relationship to responsivity to PTSD treatment. Psychological trauma: theory, research, practice, and policy. 2014 Jan;6(1):56. | Subgroup/secondary analysis that is not relevant |
| 163 | Gamito 2010 | Gamito P, Oliveira J, Rosa P, Morais D, Duarte N, Oliveira S, Saraiva T. PTSD elderly war veterans: A clinical controlled pilot study. Cyberpsychology, Behavior, and Social Networking. 2010 Feb 1;13(1):43-8. | Sample size (N<10/arm) |
| 164 | Geiger-Brown 2015 | Geiger-Brown, JM.; Rogers, VE.; Liu, W.; Ludeman, EM.; Downton, KD.; Diaz-Abad, M.; (2015) Cognitive behavioral therapy in persons with comorbid insomnia: A meta-analysis. Sleep Medicine Reviews 23:54-67 | Systematic review with no new useable data and any meta-analysis results not appropriate to extract |
| 165 | Gelkopf 2013 | Gelkopf M, Hasson-Ohayon I, Bikman M, Kravetz S. Nature adventure rehabilitation for combat-related posttraumatic chronic stress disorder: A randomized control trial. Psychiatry research. 2013 Oct 30;209(3):485-93. | Outcome measures are not validated |
| 166 | Gerardi 2010 | Gerardi M, Rothbaum BO, Astin MC, Kelley M. Cortisol response following exposure treatment for PTSD in rape victims. Journal of aggression, maltreatment & trauma. 2010 May 27;19(4):349-56. | Subgroup/secondary analysis of RCT already included |
| 167 | Gerger 2014a | Gerger, H.; Munder, T.; Barth, J.; (2014) Specific and Nonspecific psychological Interventions for PTSD Symptoms: A Meta-analysis with Problem Complexity as a Moderator. J Clink Psych 70(7): 601-615. | Systematic review with no new useable data and any meta-analysis results not appropriate to extract |
| 168 | Gerger 2014b | Gerger, H.; Munder, T.; Gemperli, A.; Nuesch, E.; Trelle, S.; Juni, P.; Barth,J.; (2014) Integrating fragmented evidence by network meta-analysis: relative effectiveness of psychological interventions for adults with post-traumatic stress disorder. Pscyh Med 44(15): 3151-3164 | Systematic review with no new useable data and any meta-analysis results not appropriate to extract |
| 169 | Germain 2009 | Germain, V.; Marchand, A.; Bouchard, S.; Drouin, MS.; Guay, S.; (2009) Effectiveness of Cognitive Behavioural Therapy Administered by Videoconference for Posttraumatic Stress Disorder. Cog Behav Therapy 38 (1): 42-53 | Non-randomised group assignment |
| 170 | Gham 2010 | Gham GA, Reger G. Comparing Virtual Reality Exposure Therapy to Prolonged Exposure in the Treatment of Soldiers With PTSD [NCT01193725]. 2010. Available from: https://clinicaltrials.gov/ct2/show/NCT01193725 [accessed 02.08.2017] | Population outside scope: Trials of soldiers on active service |
| 171 | Ginzburg 2009 | Ginzburg K, Butler LD, Giese-Davis J, Cavanaugh CE, Neri E, Koopman C, Classen CC, Spiegel D. Shame, guilt, and posttraumatic stress disorder in adult survivors of childhood sexual abuse at risk for human immunodeficiency virus: outcomes of a randomized clinical trial of group psychotherapy treatment. The Journal of nervous and mental disease. 2009 Jul 1;197(7):536-42. | Subgroup/secondary analysis of RCT already included |
| 172 | Glavin 2017 | Glavin CE, Montgomery P. Creative bibliotherapy for post-traumatic stress disorder (PTSD): a systematic review. Journal of Poetry Therapy. 2017 Apr 3;30(2):95-107. | Systematic review with no new useable data and any meta-analysis results not appropriate to extract |
| 173 | Glynn 1999 | Glynn, S. M., Eth, S., Randolph, E. T., Foy, D. W., Urbaitis, M., Boxer, L. et al. (1999). A test of behavioral family therapy to augment exposure for combat-related posttraumatic stress disorder. Journal of Consulting & Clinical Psychology, 67, 243-251. | Efficacy or safety data cannot be extracted |
| 174 | Goetter 2015 | Goetter, EM.; bui, E.; Ojserkis, RA.; Zakarian, RJ.; Brendel, RW.; Simon, NM.; (2015) A systematic Review of Dropout From Psychotherapy for Posttraumatic Stress disorder Among Iraq and Afanistan Combat Veterans. J Traum Stress 28(5): 401-409 | Systematic review with no new useable data and any meta-analysis results not appropriate to extract |
| 175 | Goncalves 2011 | Goncalves, R.; Lages, AC.; Rodrigues, H.; Pedrozo, AL.; Coutinho, ESF.; Neylan, T.; Figueira, I.; Ventura, P.; (2011) Potenciais biomarcadores da terapia cognitivo-comportamental para o transtorno de estresse pos-traumatico: uma revisao sistematica. Arch of Clin Psyh | Systematic review with no new useable data and any meta-analysis results not appropriate to extract |
| 176 | Gonclaves 2012 | Gancalves, R.; Pedrozo, AL.; Coutinho, ESF.; Figueria, I.; Ventura, P.; (2012) Efficacy of Virtual Reality Exposure Therapy in the Treatment of PTSD: A Systematic Review. PLoS ONE 7(12): e48469. | Systematic review with no new useable data and any meta-analysis results not appropriate to extract |
| 177 | Goodson 2011 | Goodson, J.; Helstrom, A.; Halpern,JM.; Ferenschak, MP.; Gillihan,SJ.; Powers, MB.; (2011) Treatment of Posttraumatic Stress Disorder in U.S. Combat Veterans: A Meta-Analytic Review. Pscyh Reports 109(2): 573-599 | Systematic review with no new useable data and any meta-analysis results not appropriate to extract |
| 178 | Grainger 1997 | Grainger, R.D.; Levin, C.; Allen-Byrd, L.; Doctor, R.M., Lee, H. (1997) An empirical evaluation of eye movement desensitization and reprocessing (EMDR) with survivors of a natural disaster. Journal of Traumatic Stress, 10, 4, 665-671 | Efficacy or safety data cannot be extracted |
| 179 | Green 2006 | Green BL, Krupnick JL, Chung J, Siddique J, Krause ED, Revicki D, Frank L, Miranda J. Impact of PTSD comorbidity on one‐year outcomes in a depression trial. Journal of clinical psychology. 2006 Jul 1;62(7):815-35. | Intervention not targeted at PTSD symptoms |
| 180 | Gregg 2007 | Gregg, L.; Tarrier, N.; (2007) Virtual realisty in mental health. Social Psychiatry and Psychiactric Epidimilogy 42(5):343-354 | Non-systematic review |
| 181 | Griffiths 2010 | Griffiths, KM.; Farrer, L.; Christensen, H.; (2010) The efficacy of internet interventions for depression and anxiety disorders: a review of randomised controlled trials. MJA 192:S4-S11 | Non-systematic review |
| 182 | Grist 2013 | Grist, R.; Cavanagh, K.; (2013) Computerised Cognitive Behavioural Therapy for Common Mental Health Disorders, What Works, for Whom Under What Circumstances? A Systematic Review and Meta-analysis. J Contemporary Pscyhotherapy 43(4):243-251 | Systematic review with no new useable data and any meta-analysis results not appropriate to extract |
| 183 | Gutner 2013 | Gutner CA, Casement MD, Gilbert KS, Resick PA. Change in sleep symptoms across cognitive processing therapy and prolonged exposure: a longitudinal perspective. Behaviour research and therapy. 2013 Dec 31;51(12):817-22. | Subgroup/secondary analysis of RCT already included |
| 184 | Gutner 2016a | Gutner CA, Gallagher MW, Baker AS, Sloan DM, Resick PA. Time course of treatment dropout in cognitive–behavioral therapies for posttraumatic stress disorder. Psychological Trauma: Theory, Research, Practice, and Policy. 2016 Jan;8(1):115. | Non-primary study |
| 185 | Gutner 2016b | Gutner CA, Suvak MK, Sloan DM, Resick PA. Does timing matter? Examining the impact of session timing on outcome. Journal of consulting and clinical psychology. 2016 Dec;84(12):1108. | Subgroup/secondary analysis of RCT already included |
| 186 | Gwodzdziewycz 2013 | Gwozdziewycz, N.; Mehl-Madrona, L.; (2013) Meta-Analysis of the Use of Narrative Exposure Therapy for the Effects of Trauma Among Refuge Populations. Permanente Journal 17(1): 70-76 | Systematic review with no new useable data and any meta-analysis results not appropriate to extract |
| 187 | Haagen 2015 | Haagen, JFG.; Smid, GE.; Knipscgeer, JW.; Kleber, RJ.; (2015) The efficacy of recommended treatments for veterans with PTSD: A metaregression analysis. Clinical Psychology Review 40:184-94. | Systematic review with no new useable data and any meta-analysis results not appropriate to extract |
| 188 | Haagen 2016 | Haagen JF, Heide F, Mooren TM, Knipscheer JW, Kleber RJ. Predicting post‐traumatic stress disorder treatment response in refugees: Multilevel analysis. British Journal of Clinical Psychology. 2017 Mar 1;56(1):69-83. | Subgroup/secondary analysis that is not relevant |
| 189 | Haller 2016 | Haller M, Norman SB, Cummins K, Trim RS, Xu X, Cui R, Allard CB, Brown SA, Tate SR. Integrated cognitive behavioral therapy versus cognitive processing therapy for adults with depression, substance use disorder, and trauma. Journal of substance abuse treatment. 2016 Mar 31;62:38-48. | Comparison outside protocol |
| 190 | Halvorsen 2014 | Halvorsen JØ, Stenmark H, Neuner F, Nordahl HM. Does dissociation moderate treatment outcomes of narrative exposure therapy for PTSD? A secondary analysis from a randomized controlled clinical trial. Behaviour Research and Therapy. 2014 Jun 30;57:21-8. | Subgroup/secondary analysis that is not relevant |
| 191 | Hansen 2013 | Hansen, K.; Hofling, V.; Kroner-Borowik, T.; Stangier, U.; Steil, R.; (2013) Efficacy of psychological interventions aiming to reduce chronic nightmares: A meta-analysis. Clinical Psychology Review 33(1): 146-155 | Systematic review with no new useable data and any meta-analysis results not appropriate to extract |
| 192 | Harned 2014 | Harned MS, Korslund KE, Linehan MM. A pilot randomized controlled trial of Dialectical Behavior Therapy with and without the Dialectical Behavior Therapy Prolonged Exposure protocol for suicidal and self-injuring women with borderline personality disorder and PTSD. Behaviour research and therapy. 2014 Apr 30;55:7-17. | Sample size (N<10/arm) |
| 193 | Hart 2011 | Hart J. Novel Treatment of Emotional Dysfunction in Post Traumatic Stress Disorder (PTSD) [NCT01391832]. 2011. Available from: https://clinicaltrials.gov/show/NCT01391832 [accessed 03.08.2017] | Unpublished (registered on clinical trials.gov and author contacted for full trial report but not provided) |
| 194 | Haug 2012 | Haug, t.; Nordgreen, T.; Ost, LG.; Havik, OE.; (2012) Self-help treatment of anxiety disorders: A meta-analysis and meta-regression of effects and potential moderators. Clinical Psychology Review 32(5): 425-445. | Systematic review with no new useable data and any meta-analysis results not appropriate to extract |
| 195 | Haugen 2012 | Haugen, PT.; Evces, M.; Weiss, DS.; (2012) Treating posttraumatic stress disorder in first responders: A systematic review. Clinical Psychology Review 32(5): 370-380 | Systematic review with no new useable data and any meta-analysis results not appropriate to extract |
| 196 | Hembree 2003 | Hembree EA, Foa EB, Gaulin AE. Effectiveness of treatment for PTSD in community agencies [NCT00057629]. 2003. Available from: https://clinicaltrials.gov/ct2/show/NCT00057629 [accessed 03.08.2017] | Unpublished (registered on clinical trials registry and author contacted for full trial report but not provided) |
| 197 | Hembree 2004 | Hembree EA, Cahill SP, Foa EB. Impact of personality disorders on treatment outcome for female assault survivors with chronic posttraumatic stress disorder. Journal of Personality Disorders. 2004 Feb 1;18(1):117-27. | Comparison outside protocol |
| 198 | Hertlein 2004 | Hertlein, KM.; Ricci, RJ.; (2004) A Systematic Research Synthesis of EMDR Studies. Implementation of the Platinum Standard. Trauma, Violence and Abuse 5(3): 285-300 | Systematic review with no new useable data and any meta-analysis results not appropriate to extract |
| 199 | Hickling 1997 | Hickling, E.J.; Blanchard, E.B. (1997) The private practice psychologist and manual-based treatments: post-traumatic stress disorder secondary to motor vehicle accidents. Behavior Research & Therapy, 35, 3, 191-203 | Non-randomised group assignment |
| 200 | Hien 2004 | Hien DA, Cohen LR, Miele GM, Litt LC, Capstick C. Promising treatments for women with comorbid PTSD and substance use disorders. American journal of Psychiatry. 2004 Aug 1;161(8):1426-32. | Comparison outside protocol |
| 201 | Hien 2010a/2010b/2010c/2012 | Hien DA, Campbell AN, Killeen T, Hu MC, Hansen C, Jiang H, Hatch-Maillette M, Miele GM, Cohen LR, Gan W, Resko SM. The impact of trauma-focused group therapy upon HIV sexual risk behaviors in the NIDA Clinical Trials Network “Women and trauma” multi-site study. AIDS and Behavior. 2010 Apr 1;14(2):421-30.  Hien DA, Campbell AN, Ruglass LM, Hu MC, Killeen T. The role of alcohol misuse in PTSD outcomes for women in community treatment: A secondary analysis of NIDA's Women and Trauma Study. Drug and Alcohol Dependence. 2010 Sep 1;111(1):114-9. | Subgroup/secondary analysis of RCT already included |
| 202 | Hien 2017 | Hien DA, Lopez-Castro T, Papini S, Gorman B, Ruglass LM. Emotion dysregulation moderates the effect of cognitive behavior therapy with prolonged exposure for co-occurring PTSD and substance use disorders. Journal of anxiety disorders. 2017 Dec 31;52:53-61. | Subgroup/secondary analysis of RCT already included |
| 203 | Hilton 2017 | Hilton, L.; Maher, AR.; Colaiaco, B.; Apaydin, E.; Sorbero, ME.; Booth, M.; Shanman, RM.; Hempel, S.; (2017) Meditation for Posttraumatic Stress: Systematic Review and Meta-Analysis. Psychological Trauma: Theory, Research, Practice and Policy 9(4): 453-460 | Systematic review with no new useable data and any meta-analysis results not appropriate to extract |
| 204 | Hirai 2012 | Hirai M, Skidmore ST, Clum GA, Dolma S. An investigation of the efficacy of online expressive writing for trauma-related psychological distress in Hispanic individuals. Behavior therapy. 2012 Dec 31;43(4):812-24. | Comparison outside protocol |
| 205 | Ho 2012 | Ho, MSK.; Lee, CW.; (2012) Cognitive behaviour therapy versus eye movement desensitization and reprocessing for post-traumatic disorder- is it all in the homework then? European Review of Applied Psychology 62 (4): 253-260 | Systematic review with no new useable data and any meta-analysis results not appropriate to extract |
| 206 | Ho 2016 | Ho, FY-Y.; Chan, CS.; Tang,KN-S.; (2016) Cognitive-behavioral therapy for sleep disturbances in treating posttraumatic stress disorder symptoms: A met-analysis of randomised controlled trials. Clinical Pscyhology Review 43: 90-102 | Systematic review with no new useable data and any meta-analysis results not appropriate to extract |
| 207 | Hoffart 2015 | Hoffart A, Øktedalen T, Langkaas TF. Self-compassion influences PTSD symptoms in the process of change in trauma-focused cognitive-behavioral therapies: a study of within-person processes. Frontiers in psychology. 2015;6. | Comparison outside protocol |
| 208 | Holder 2017 | Holder N, Holliday R, Pai A, Surís A. Role of Borderline Personality Disorder in the Treatment of Military Sexual Trauma-related Posttraumatic Stress Disorder with Cognitive Processing Therapy. Behavioral Medicine. 2017 Jul 3;43(3):184-90. | Subgroup/secondary analysis of RCT already included |
| 209 | Hopwood 2017 | Hopwood TL, Schutte NS. A meta-analytic investigation of the impact of mindfulness-based interventions on post traumatic stress. Clinical psychology review. 2017 Nov 1;57:12-20. | Systematic review with no new useable data and any meta-analysis results not appropriate to extract |
| 210 | Hinsberger 2016 | Hinsberger, M., Holtzhausen, L., Sommer, J., Kaminer, D., Elbert, T., Seedat, S., ... & Weierstall, R. (2016). Feasibility and Effectiveness of Narrative Exposure Therapy and Cognitive Behavioral Therapy in a Context of Ongoing Violence in South Africa. | Efficacy or safety data cannot be extracted |
| 211 | Hofman 2008 | Hofman, SG.; Smits,JAJ.; (2008) Cognitive-behavioral therapy for adult anxiety disorders: A meta-analysis of randomised placebo-controlled trials. J Clinical Psychiatry 69(4): 621-632 | Systematic review with no new useable data and any meta-analysis results not appropriate to extract |
| 212 | Hofman 2014 | Hofman, SG.l Wu, JQ.; Boettcher, H.; (2014) Effect of Cognitive-Behavioral Therapy for Anxiety Disorders on Quality of Life: A Meta-Analysis. J Cons and Clin Psychology 82(3): 375-391 | Systematic review with no new useable data and any meta-analysis results not appropriate to extract |
| 213 | Hofmann 1996 | Hofmann, A. (1996). Eye movement desensitization and reprocessing: A new treatment method for post-traumatic stress disorder. Psychotherapeut, 41, 368-372. | Non-randomised group assignment |
| 214 | Hogberg 2007 | Högberg G, Pagani M, Sundin Ö, Soares J, Åberg-Wistedt A, Tärnell B, Hällström T. On treatment with eye movement desensitization and reprocessing of chronic post-traumatic stress disorder in public transportation workers–A randomized controlled trial. Nordic journal of psychiatry. 2007 Jan 1;61(1):54-61. | Sample size (N<10/arm) |
| 215 | Holliday 2014 | Holliday R, Link-Malcolm J, Morris EE, Surís A. Effects of cognitive processing therapy on PTSD-related negative cognitions in veterans with military sexual trauma. Military medicine. 2014 Oct;179(10):1077-82. | Efficacy or safety data cannot be extracted |
| 216 | Holliday 2015 | Holliday R, Williams R, Bird J, Mullen K, Surís A. The role of cognitive processing therapy in improving psychosocial functioning, health, and quality of life in veterans with military sexual trauma-related posttraumatic stress disorder. Psychological services. 2015 Nov;12(4):428. | Efficacy or safety data cannot be extracted |
| 217 | Holliday 2017 | Holliday RP, Holder ND, Williamson ML, Surís A. Therapeutic response to Cognitive Processing Therapy in White and Black female veterans with military sexual trauma-related PTSD. Cognitive behaviour therapy. 2017 Sep 3;46(5):432-46. | Efficacy or safety data cannot be extracted |
| 218 | Hollifield 2016 | Hollifield, M.; Gory, A.; Siedjak, J.; Nguyen, L.; Holmgreen, L.; Hobfoll, S.; (2016) The Benefit of Conserving and Gaining Resources after Trauma: A Systematic Review. J Clin Med 5(11: 104 | Systematic review with no new useable data and any meta-analysis results not appropriate to extract |
| 219 | Hossack 1996 | Hossack, Alex and Bentall, Richard P. (1996) Elimination of Post-traumatic Symptomatology by Relaxation and Visual-Kinesthetic Dissociation. Journal of Traumatic Stress, Vol 9, No1, 99-110 | Non-randomised group assignment |
| 220 | Hunt 2014 | Hunt, M., Chizkov, R. (2014) Are therapy dogs like Xanax? Does animal-assisted therapy impact processes relevant to cognitive behavioral psychotherapy?, Anthrozoos, 27, 457-469 | Population outside scope: Trials of people without PTSD |
| 221 | Igreja 2004 | Igreja, V., Kleijn, W. C., Schreuder, B. J., Van Dijk, J. A., & Verschuur, M. (2004). Testimony method to ameliorate post-traumatic stress symptoms. Community-based intervention study with Mozambican civil war survivors. Br.J.Psychiatry, 184, 251-257 | Non-randomised group assignment |
| 222 | Imel 2013 | Imel, ZE.; Laska, K.; Jakupcak, M.; Simpson, TL.; (2013) Meta-Analysis of Dropout in Treatment for Posttrumatic Stress Disorder. J Cons and Clin Psyh 81(3): 394-404 | Systematic review with no new useable data and any meta-analysis results not appropriate to extract |
| 223 | Ironson 2002 | Ironson, G.I., Freund, B., Strauss, J.L., & Williams, J. (2002). A comparison of two treatments for traumatic stress: A community based study of EMDR and prolonged exposure. Journal of Clinical Psychology, 58, 113-128 | Sample size (N<10/arm) |
| 224 | Isserles 2013 | Isserles M, Shalev AY, Roth Y, Peri T, Kutz I, Zlotnick E, Zangen A. Effectiveness of deep transcranial magnetic stimulation combined with a brief exposure procedure in post-traumatic stress disorder–a pilot study. Brain stimulation. 2013 May 31;6(3):377-83. | Sample size (N<10/arm) |
| 225 | Iverson 2011 | Iverson KM, Gradus JL, Resick PA, Suvak MK, Smith KF, Monson CM. Cognitive–behavioral therapy for PTSD and depression symptoms reduces risk for future intimate partner violence among interpersonal trauma survivors. Journal of consulting and clinical psychology. 2011 Apr;79(2):193. | Subgroup/secondary analysis that is not relevant |
| 226 | Jayakody 2013 | Jayakody, K.; Gunadasa, S.; Hosker, C.; (2013) Exercise for anxiety disorders: systematic review. Br J Sports Med 00:1-11 | Systematic review with no new useable data and any meta-analysis results not appropriate to extract |
| 227 | Jayawickreme 2014 | Jayawickreme, N.; Cahill, SP.; Riggs, DS.; Rauch, SAM.; Resick, PA.; Rothbaum, BO.; Foa, EB.; (2014) Primum non nocere (first do no harm): Symptom worsening and improvement in female assault victims after prolonged exposure for PTSD. Depression and Anxiety 31(5): 412-419 | Systematic review with no new useable data and any meta-analysis results not appropriate to extract |
| 228 | Jerud 2016 | Jerud AB, Pruitt LD, Zoellner LA, Feeny NC. The effects of prolonged exposure and sertraline on emotion regulation in individuals with posttraumatic stress disorder. Behaviour research and therapy. 2016 Feb 29;77:62-7. | Subgroup/secondary analysis that is not relevant |
| 229 | Johnson 2002 | Johnson, D. R. & Lubin, H. (2002). Effect of brief versus long-term inpatient treatment on homecoming stress in combat-related posttraumatic stress disorder: Three-year follow-up. Journal of Nervous & Mental Disease, 190, 47-51 | Non-randomised group assignment |
| 230 | Johnson 2006 | Johnson DR, Lubin H. The Counting Method: Applying the Rule of Parsimony to the Treatment of Posttraumatic Stress Disorder. Traumatology. 2006 Mar;12(1):83. | Sample size (N<10/arm) |
| 231 | Johnson 2018 | Johnson RA, Albright DL, Marzolf JR, Bibbo JL, Yaglom HD, Crowder SM, Carlisle GK, Willard A, Russell CL, Grindler K, Osterlind S. Effects of therapeutic horseback riding on post-traumatic stress disorder in military veterans. Military Medical Research. 2018 Dec;5(1):3. | Cross-over study and first phase data not available |
| 232 | Jonas 2013 | Jonas, DE.; Cusack, K.; Forneris, CA.; (2103) Psychological and Pharmacological Treatments for Adults with Posttraumatic Stress Disorder (PTSD). Comparative Effectiveness Reviews 92 | Systematic review with no new useable data and any meta-analysis results not appropriate to extract |
| 233 | Jun 2013 | Jun JJ, Zoellner LA, Feeny NC. Sudden gains in prolonged exposure and sertraline for chronic PTSD. Depression and anxiety. 2013 Jul 1;30(7):607-13. | Efficacy or safety data cannot be extracted |
| 234 | Kar 2011 | Kar, N.; (2011) Cognitive behavioral therapy for the treatment of post-traumatic stress disorder: a review. Neuropsychiatric Disase and Treatment 7: 167-181 | Non-systematic review |
| 235 | Karatzias 2007 | Karatzias A, Power K, McGoldrick T, Brown K, Buchanan R, Sharp D, Swanson V. Predicting treatment outcome on three measures for post-traumatic stress disorder. European archives of psychiatry and clinical neuroscience. 2007 Feb 1;257(1):40-6. | Subgroup/secondary analysis of RCT already included |
| 236 | Keane 1982 | Keane TM, Kaloupek DG. Imaginal flooding in the treatment of a posttraumatic stress disorder. Journal of Consulting and Clinical Psychology. 1982 Feb;50(1):138. | Non-RCT (no control group) |
| 237 | Keane 1989 | Keane, T. M., Fairbank, J. A., Caddell, J. M., & Zimering, R. T. (1989). Implosive (flooding) therapy reduces symptoms of PTSD in Vietnam combat veterans. Behavior Therapy, 20, 245-260. | Efficacy or safety data cannot be extracted |
| 238 | Keefe 2014 | Keefe, JR.; McCarthy, KS.; Dinger, U.; Zilcha-Mano, S.; Barber, JP.; (2014) A meta-analytic review of psychodynamic therapies for anxiety disorders. Clinc Psych Rev 34(4): 309-323 | Systematic review with no new useable data and any meta-analysis results not appropriate to extract |
| 239 | Kehle-Forbes 2013 | Kehle-Forbes, SM.; Polusny, MA.; MacDonald, R.; Murdoch, M.; Meis, LA.; Wilt, TJ.; (2013) A Systematic Review of the Efficacy of Adding Nonexposure Components to Exposure Therapy for Posttraumatic Stress Disorder. Psychological Trauma: Theory, Research, Practice and Policy 5(4): 317-322. | Systematic review with no new useable data and any meta-analysis results not appropriate to extract |
| 240 | Killeen 2008 | Killeen T, Hien D, Campbell A, Brown C, Hansen C, Jiang H, Kristman-Valente A, Neuenfeldt C, Rocz-de la Luz N, Sampson R, Suarez-Morales L. Adverse events in an integrated trauma-focused intervention for women in community substance abuse treatment. Journal of substance abuse treatment. 2008 Oct 31;35(3):304-11. | Efficacy or safety data cannot be extracted |
| 241 | Kim 2013 | Kim, Y-D.; Heo, I.; Shin, B-C.; Crawford, C.; Kang, H-W.; Lim, J-H.; (2013) Acupuncture for Posttraumatic Stress Disorder: A systematic Reivew of Randomised Controlled Trials and Prospective Clinical Trials. Evidence-Based Complementary and Alternative Medicine: ID 615857 | Systematic review with no new useable data and any meta-analysis results not appropriate to extract |
| 242 | Kimbrell 2009 | Kimbrell TA. Adjunctive Biofeedback Intervention for OIF-OEF PTSD [NCT00920036]. Available from: https://clinicaltrials.gov/show/NCT00920036 [accessed 08.08.2017] | Sample size (N<10/arm) |
| 243 | King 2013 | King AP, Erickson TM, Giardino ND, Favorite T, Rauch SA, Robinson E, Kulkarni M, Liberzon I. A pilot study of group mindfulness‐based cognitive therapy (MBCT) for combat veterans with posttraumatic stress disorder (PTSD). Depression and anxiety. 2013 Jul 1;30(7):638-45. | Non-randomised group assignment |
| 244 | King 2015 | King HC, Spence DL, Hickey AH, Sargent P, Elesh R, Connelly CD. Auricular acupuncture for sleep disturbance in veterans with post-traumatic stress disorder: a feasibility study. Military medicine. 2015 May;180(5):582-90. | Sample size (N<10/arm) |
| 245 | Kip 2013 | Kip KE, Rosenzweig L, Hernandez DF, Shuman A, Sullivan KL, Long CJ, Taylor J, McGhee S, Girling SA, Wittenberg T, Sahebzamani FM. Randomized controlled trial of accelerated resolution therapy (ART) for symptoms of combat-related post-traumatic stress disorder (PTSD). Military Medicine. 2013 Dec;178(12):1298-309. | Cross-over study and first phase data not available |
| 246 | Kip 2014 | Kip KE, Rosenzweig L, Hernandez DF, Shuman A, Diamond DM, Ann Girling S, Sullivan KL, Wittenberg T, Witt AM, Lengacher CA, Anderson B. Accelerated Resolution Therapy for treatment of pain secondary to symptoms of combat-related posttraumatic stress disorder. European journal of psychotraumatology. 2014 Dec 1;5(1):24066. | Subgroup/secondary analysis that is not relevant |
| 247 | Kitchiner 2012 | Kitchiner, NP.; Roberts, NJ.; Wilcox, D.; Bisson, JI.; (2012) Systematic review and meta-analsyis of psychosocial interventions for veterans of the military. Eur J Pscyhotraumatology 3(1) | Systematic review with no new useable data and any meta-analysis results not appropriate to extract |
| 248 | Kline 2018 | Kline AC, Cooper AA, Rytwinksi NK, Feeny NC. Long-term efficacy of psychotherapy for posttraumatic stress disorder: A meta-analysis of randomized controlled trials. Clinical psychology review. 2017 Nov 21. | Systematic review with no new useable data and any meta-analysis results not appropriate to extract |
| 249 | Knaevelsrud 2011 | Knaevelsrud C. Additive Effect of Cognitive Restructuring in a Web-based Treatment for Traumatized Arab People [NCT01508377]. 2011. Available from: https://clinicaltrials.gov/ct2/show/NCT01508377 [accessed 04.08.2017] | Comparison outside protocol |
| 250 | Kobach 2015 | Köbach, A., Schaal, S., Hecker, T., & Elbert, T. (2015). Psychotherapeutic Intervention in the Demobilization Process: Addressing Combat‐related Mental Injuries with Narrative Exposure in a First and Second Dissemination Stage. Clinical psychology & psychotherapy. | Non-randomised group assignment |
| 251 | Konig 2014 | König J, Karl R, Rosner R, Butollo W. Sudden gains in two psychotherapies for posttraumatic stress disorder. Behaviour research and therapy. 2014 Sep 30;60:15-22. | Subgroup/secondary analysis that is not relevant |
| 252 | Konuk 2006 | Konuk E, Knipe J, Eke I, Yuksek H, Yurtsever A, Ostep S. The effects of eye movement desensitization and reprocessing (EMDR) therapy on posttraumatic stress disorder in survivors of the 1999 Marmara, Turkey, earthquake. International Journal of Stress Management. 2006 Aug;13(3):291. | Non-randomised group assignment |
| 253 | Korte 2017 | Korte KJ, Bountress KE, Tomko RL, Killeen T, Moran-Santa Maria M, Back SE. Integrated Treatment of PTSD and Substance Use Disorders: The Mediating Role of PTSD Improvement in the Reduction of Depression. Journal of clinical medicine. 2017 Jan 13;6(1):9. | Efficacy or safety data cannot be extracted |
| 254 | Krakow 2001a | Krakow B, Hollifield M, Johnston L, Koss M, Schrader R, Warner TD, Tandberg D, Lauriello J, McBride L, Cutchen L, Cheng D. Imagery rehearsal therapy for chronic nightmares in sexual assault survivors with posttraumatic stress disorder: a randomized controlled trial. Jama. 2001 Aug 1;286(5):537-45. | Efficacy or safety data cannot be extracted |
| 255 | Krakow 2001b | Krakow, B., Johnston, L., Melendrez, D., Hollifield, M., Warner, T. D., Chavez-Kennedy, D. et al. (2001). An open-label trial of evidence-based cognitive behavior therapy for nightmares and insomnia in crime victims with PTSD. American Journal of Psychiatry, 158, 2043-2047. | Non-RCT (no control group) |
| 256 | Kredlow 2017 | Kredlow MA, Szuhany KL, Lo S, Xie H, Gottlieb JD, Rosenberg SD, Mueser KT. Cognitive behavioral therapy for posttraumatic stress disorder in individuals with severe mental illness and borderline personality disorder. Psychiatry research. 2017 Mar 31;249:86-93. | Subgroup/secondary analysis that is not relevant |
| 257 | Krinsley 2011 | Krinsley K. Pilot Study of an Integrated Exposure-Based Model for Posttraumatic Stress Disorder and Substance Use Disorder [NCT01274741]. Available from: https://clinicaltrials.gov/ct2/show/NCT01274741 [accessed 08.08.2017] | Unpublished (registered on clinical trials.gov and author contacted for full trial report but not provided) |
| 258 | Kruger 2014a | Krüger A, Ehring T, Priebe K, Dyer AS, Steil R, Bohus M. Sudden losses and sudden gains during a DBT-PTSD treatment for posttraumatic stress disorder following childhood sexual abuse. European journal of psychotraumatology. 2014 Dec 1;5(1):24470. | Subgroup/secondary analysis of RCT already included |
| 259 | Kruger 2014b | Krüger A, Kleindienst N, Priebe K, Dyer AS, Steil R, Schmahl C, Bohus M. Non-suicidal self-injury during an exposure-based treatment in patients with posttraumatic stress disorder and borderline features. Behaviour research and therapy. 2014 Oct 31;61:136-41. | Subgroup/secondary analysis of RCT already included |
| 260 | Krupnick 2017 | Krupnick JL, Green BL, Amdur R, Alaoui A, Belouali A, Roberge E, Cueva D, Roberts M, Melnikoff E, Dutton MA. An Internet-based writing intervention for PTSD in veterans: A feasibility and pilot effectiveness trial. Psychological Trauma: Theory, Research, Practice, and Policy. 2017 Jul;9(4):461. | Sample size (N<10/arm) |
| 261 | Kruse 2009 | Kruse J, Joksimovic L, Cavka M, Wöller W, Schmitz N. Effects of trauma‐focused psychotherapy upon war refugees. Journal of Traumatic Stress. 2009 Dec 1;22(6):585-92. | Non-randomised group assignment |
| 262 | Kuckertz 2014 | Kuckertz JM, Amir N, Boffa JW, Warren CK, Rindt SE, Norman S, Ram V, Ziajko L, Webb-Murphy J, McLay R. The effectiveness of an attention bias modification program as an adjunctive treatment for post-traumatic stress disorder. Behaviour research and therapy. 2014 Dec 31;63:25-35. | Population outside scope: Trials of soldiers on active service |
| 263 | Kuester 2016 | Kuester, A. Niemeyer, H.; Knaevelsrud, C.; (2016) Internet-based interventions for posttraumatic stress: A meta-analysis of randomised controlled trials. Clin Pscyh Rev 43:1-16 | Systematic review with no new useable data and any meta-analysis results not appropriate to extract |
| 264 | Lambert 2015 | Lambert, JE.; Alhassoon, OM.; (2015) Trauma-Focused therapy for Refugees: Meta-Analytic Findings. J Counseling Pscychology 62(1): 28-37 | Systematic review with no new useable data and any meta-analysis results not appropriate to extract |
| 265 | Lamprecht 2004 | Lamprecht F, Köhnke C, Lempa W, Sack M, Matzke M, Münte TF. Event-related potentials and EMDR treatment of post-traumatic stress disorder. Neuroscience Research. 2004 Jun 30;49(2):267-72. | Non-randomised group assignment |
| 266 | Lancee 2010 | Lancee J, Van Den Bout J, Spoormaker VI. Expanding self-help imagery rehearsal therapy for nightmares with sleep hygiene and lucid dreaming: a waiting-list controlled trial. Universitätsbibliothek der Universität Heidelberg; 2010 | Population outside scope: <80% of the study's participants are eligible for the review and disaggregated data cannot be obtained |
| 267 | Langkaas 2017 | Langkaas TF, Hoffart A, Øktedalen T, Ulvenes PG, Hembree EA, Smucker M. Exposure and non-fear emotions: A randomized controlled study of exposure-based and rescripting-based imagery in PTSD treatment. Behaviour research and therapy. 2017 Oct 1;97:33-42. | Comparison outside protocol |
| 268 | Lau 2007 | Lau M, Kristensen E. Outcome of systemic and analytic group psychotherapy for adult women with history of intrafamilial childhood sexual abuse: a randomized controlled study. Acta Psychiatrica Scandinavica. 2007 Aug 1;116(2):96-104. | Comparison outside protocol |
| 269 | Lawrence 2010 | Lawrence, S., De Silva, M., Henley, R. (2010) Sports and games for post-traumatic stress disorder (PTSD), Cochrane database of systematic reviews, CD007171 | Systematic review with no new useable data and any meta-analysis results not appropriate to extract |
| 270 | Le 2013/2014 | Le QA, Doctor JN, Zoellner LA, Feeny NC. Minimal clinically important differences for the EQ-5D and QWB-SA in Post-traumatic Stress Disorder (PTSD): results from a Doubly Randomized Preference Trial (DRPT). Health and quality of life outcomes. 2013 Apr 12;11(1):1.  Le QA, Doctor JN, Zoellner LA, Feeny NC. Cost-effectiveness of prolonged exposure therapy versus pharmacotherapy and treatment choice in posttraumatic stress disorder (the Optimizing PTSD Treatment Trial): a doubly randomized preference trial. The Journal of clinical psychiatry. 2014 Mar 15;75(3):222-30. | Efficacy or safety data cannot be extracted |
| 271 | LeBouthillier 2016 | LeBouthillier DM, Fetzner MG, Asmundson GJ. Lower cardiorespiratory fitness is associated with greater reduction in PTSD symptoms and anxiety sensitivity following aerobic exercise. Mental Health and Physical Activity. 2016 Mar 31;10:33-9. | Subgroup/secondary analysis that is not relevant |
| 272 | Lee 2002 | Lee, C., Gavriel, H., Drummond, P., Richards, J., & Greenwald, R. (2002). Treatment of PTSD: stress inoculation training with prolonged exposure compared to EMDR. Journal of Clinical Psychology, 58, 1071-1089. | Non-randomised group assignment |
| 273 | Lee 2016 | Lee, DJ.; Schnitzlein, CW.; Wolf, JP.; Vythilingam, M.; Rasmusson, AM.; Hoge,CW.; (2016) Psychotherapy versus Pharmacotherapy for posttraumatic stress disorder: Systemic Review and meta-analyses to determine first line treatments. Depression and Anxiety. 33: 792-806 | Systematic review with no new useable data and any meta-analysis results not appropriate to extract |
| 274 | Leeman 2017 | Leeman, RF.; Hefner, K.; Frohe, T.; Murrany, A.; Rosenheck, RA.; Watts, BV.; Sofuoglu, M.; (2017) Exclusion of participants based on substance use status: Findings from randomized controlled trials of treatments for PTSD. Behviour Research and Therapsy 89: 33-40 | Systematic review with no new useable data and any meta-analysis results not appropriate to extract |
| 275 | Leichsenring 2005 | Leichsenring, F.; 92005) Are psychodynamic and psychoanalytic therapies effective? A review of empirical data. Int j Psychoanalysis 86(3): 841-868. | Non-systematic review |
| 276 | Leichsenring 2014 | Leichsenring, F.; Klein, S.; (2014) Evidence for psychodynamic psychotherapy in specific mental disorders: a systematic review. Psychoanalytic Psychotherapy 28(1): 4-32 | Non-systematic review |
| 277 | Leichsenring 2015 | Leichsenring, F.; Luyten, P.; Hilsenroth, MJ.; Abbas, A.; Barber, JP.; Keefe, JR.; Leweke, F.; Rabung, S.; Steinert, C.; (2015) Psychodynamic therapy meets evidence-based medicine: a systematic review using updated criteria. The Lancet 2(7): 648-660. | Systematic review with no new useable data and any meta-analysis results not appropriate to extract |
| 278 | Leiner 2012 | Leiner AS, Kearns MC, Jackson JL, Astin MC, Rothbaum BO. Avoidant coping and treatment outcome in rape-related posttraumatic stress disorder. Journal of consulting and clinical psychology. 2012 Apr;80(2):317. | Subgroup/secondary analysis of RCT already included |
| 279 | Lenz 2016 | Lenz, SA.; Henesy, R.; Callender, K.; (2016) Effectiveness of Seeking Safety for Co-Occurning Posttraumatic Stress Disorder and Substance Use. J Counseling and Development 94(1): 51-61 | Non-systematic review |
| 280 | Lenz 2017 | Lenz AS, Haktanir A, Callender K. Meta‐Analysis of Trauma‐Focused Therapies for Treating the Symptoms of Posttraumatic Stress Disorder. Journal of Counseling & Development. 2017 Jul 1;95(3):339-53. | Systematic review with no new useable data and any meta-analysis results not appropriate to extract |
| 281 | Lester 2010 | Lester K, Artz C, Resick PA, Young-Xu Y. Impact of race on early treatment termination and outcomes in posttraumatic stress disorder treatment. Journal of consulting and clinical psychology. 2010 Aug;78(4):480. | Subgroup/secondary analysis that is not relevant |
| 282 | Lester 2016 | Lester P, Liang LJ, Milburn N, Mogil C, Woodward K, Nash W, Aralis H, Sinclair M, Semaan A, Klosinski L, Beardslee W. Evaluation of a family-centered preventive intervention for military families: parent and child longitudinal outcomes. Journal of the American Academy of Child & Adolescent Psychiatry. 2016 Jan 31;55(1):14-24. | Subgroup/secondary analysis that is not relevant |
| 283 | Liedl 2011 | Liedl A, Müller J, Morina N, Karl A, Denke C, Knaevelsrud C. Retracted: physical activity within a CBT intervention improves coping with pain in traumatized refugees: results of a randomized controlled design. Pain Medicine. 2011 Feb 1;12(2):234-45. | Article has been retracted |
| 284 | Lindauer 2006 | Lindauer RT, van Meijel EP, Jalink M, Olff M, Carlier IV, Gersons BP. Heart rate responsivity to script-driven imagery in posttraumatic stress disorder: specificity of response and effects of psychotherapy. Psychosomatic medicine. 2006 Jan 1;68(1):33-40. | Subgroup/secondary analysis that is not relevant |
| 285 | Litz 2007 | Litz BT, Engel CC, Bryant RA, Papa A. A randomized, controlled proof-of-concept trial of an Internet-based, therapist-assisted self-management treatment for posttraumatic stress disorder. American Journal of Psychiatry. 2007 Nov;164(11):1676-84. | Comparison outside protocol |
| 286 | Liverant 2012 | Liverant GI, Suvak MK, Pineles SL, Resick PA. Changes in posttraumatic stress disorder and depressive symptoms during cognitive processing therapy: Evidence for concurrent change. Journal of Consulting and Clinical Psychology. 2012 Dec;80(6):957. | Subgroup/secondary analysis that is not relevant |
| 287 | Lloyd 2014 | Lloyd D, Nixon RD, Varker T, Elliott P, Perry D, Bryant RA, Creamer M, Forbes D. Comorbidity in the prediction of Cognitive Processing Therapy treatment outcomes for combat-related posttraumatic stress disorder. Journal of anxiety disorders. 2014 Mar 31;28(2):237-40. | Efficacy or safety data cannot be extracted |
| 288 | Lopez-Castro 2015 | López‐Castro T, Hu MC, Papini S, Ruglass LM, Hien DA. Pathways to change: Use trajectories following trauma‐informed treatment of women with co‐occurring post‐traumatic stress disorder and substance use disorders. Drug and alcohol review. 2015 May 1;34(3):242-51. | Subgroup/secondary analysis that is not relevant |
| 289 | Lunney 2007 | Lunney CA, Schnurr PP. Domains of quality of life and symptoms in male veterans treated for posttraumatic stress disorder. Journal of traumatic stress. 2007 Dec 1;20(6):955-64. | Subgroup/secondary analysis of RCT already included |
| 290 | Macdonald 2011 | Macdonald A, Monson CM, Doron‐Lamarca S, Resick PA, Palfai TP. Identifying patterns of symptom change during a randomized controlled trial of cognitive processing therapy for military‐related posttraumatic stress disorder. Journal of Traumatic Stress. 2011 Jun 1;24(3):268-76. | Subgroup/secondary analysis of RCT already included |
| 291 | Macdonald 2016b | Macdonald A, Pukay-Martin ND, Wagner AC, Fredman SJ, Monson CM. Cognitive–behavioral conjoint therapy for PTSD improves various PTSD symptoms and trauma-related cognitions: Results from a randomized controlled trial. Journal of Family Psychology. 2016 Feb;30(1):157. | Subgroup/secondary analysis of RCT already included |
| 292 | Marcus 1997/2004 | Marcus, S. V., Marquis, P., & Sakai, C. (1997). Controlled study of treatment of PTSD using EMDR in an HMO setting. Psychotherapy: Theory, Research, Practice, Training, 34, 307-315.  Marcus S, Marquis P, Sakai C. Three-and 6-Month Follow-Up of EMDR Treatment of PTSD in an HMO Setting. International Journal of Stress Management. 2004 Aug;11(3):195. | Efficacy or safety data cannot be extracted |
| 293 | Markowitz 2014 | Markowitz, JC.; Lipsitz, J.; Milrod, BL.; (2014) Critical review of outcome research on interpersonal psychotherapy for anxiety disorders. Depression and Anxiety 31(4): 316-325 | Non-systematic review |
| 294 | Markowitz 2015b | Markowitz JC, Petkova E, Biyanova T, Ding K, Suh EJ, Neria Y. Exploring personality diagnosis stability following acute psychotherapy for chronic posttraumatic stress disorder. Depression and anxiety. 2015 Dec 1;32(12):919-26. | Subgroup/secondary analysis of RCT already included |
| 295 | Markowitz 2017 | Markowitz JC, Neria Y, Lovell K, Meter PE, Petkova E. History of sexual trauma moderates psychotherapy outcome for posttraumatic stress disorder. Depression and anxiety. 2017 Aug 1;34(8):692-700. | Subgroup/secondary analysis of RCT already included |
| 296 | Markowitz 2018 | Markowitz, J. C., Choo, T. H., & Neria, Y. (2018). Do Acute Benefits of Interpersonal Psychotherapy for Posttraumatic Stress Disorder Endure?. The Canadian Journal of Psychiatry, 63(1), 37-43 . | Efficacy or safety data cannot be extracted |
| 297 | Marks 1998/Lovell 2001 | Marks, I., Lovell, K., Noshirvani, H., Livanou, M., & Thrasher, S. (1998). Treatment of posttraumatic stress disorder by exposure and/or cognitive restructuring: a controlled study. Archives of General Psychiatry, 55, 317-325.  Lovell, K., Marks, I. M., Noshirvani, H., Thrasher, S., & Livanou, M. (2001). Do cognitive and exposure treatments improve various PTSD symptoms differently? A randomized controlled trial. Behavioural & Cognitive Psychotherapy, 29, 107-112. | Efficacy or safety data cannot be extracted |
| 298 | Martin 2015 | Martin EC, Dick AM, Scioli-Salter ER, Mitchell KS. Impact of a yoga intervention on physical activity, self-efficacy, and motivation in women with PTSD symptoms. The Journal of Alternative and Complementary Medicine. 2015 Jun 1;21(6):327-32. | Outcomes are not of interest |
| 299 | Marzabadi 2014 | Marzabadi A, SM HZ. The Effectiveness of Mindfulness Training in Improving the Quality of Life of the War Victims with Post Traumatic stress disorder (PTSD). Iranian journal of psychiatry. 2014 Oct;9(4):228-36. | Intervention not targeted at PTSD symptoms |
| 300 | Maxwell 2016 | Maxwell K, Callahan JL, Holtz P, Janis BM, Gerber MM, Connor DR. Comparative study of group treatments for posttraumatic stress disorder. Psychotherapy. 2016 Dec;53(4):433. | Sample size (N<10/arm) |
| 301 | Mayo-Wilson 2013 | Mayo-Wilson, E.; Montgomery, P.; (2013) Media-delivered cognitive behavioural therapy and behavioural therapy (self-help) for anxiety disorders in adults. Cochrane database of Systematic Reviews. | Systematic review with no new useable data and any meta-analysis results not appropriate to extract |
| 302 | McCann 2014 | McCann, RA.; Armstrong, CM.; Skopp, NA.; Edwards-Stewart, A.; Smolenshi, DJ.; June, JD.; Metger-Abamukong, M.; Reger, GM.; (2014) Virtual reality exposure therapy for the treatment of anxiety disorders: An evaluation of research quality. J of Anxiety Disorders 28(6): 625-631 | Non-systematic review |
| 303 | McFarlane 2012 | McFarlane, CA.; Kaplan, I.; (2012) Evidence-based psychological interventions for adult survivors of torture and trauma: A 30-year review. Transcultural Psychiatry 49: 3-4 | Non-systematic review |
| 304 | McHugh 2014 | McHugh RK, Hu MC, Campbell AN, Hilario E, Weiss RD, Hien DA. Changes in sleep disruption in the treatment of co‐occurring posttraumatic stress disorder and substance use disorders. Journal of traumatic stress. 2014 Feb 1;27(1):82-9. | Subgroup/secondary analysis that is not relevant |
| 305 | McLay 2009 | McLay RN. A Head-to-head Comparison of Virtual Reality Treatment for Post Traumatic Stress Disorder [NCT00978484]. 2009. Available from: https://clinicaltrials.gov/ct2/show/NCT00978484 [accessed 08.08.2017] | Population outside scope: Trials of soldiers on active service |
| 306 | McLay 2011 | McLay RN, Wood DP, Webb-Murphy JA, Spira JL, Wiederhold MD, Pyne JM, Wiederhold BK. A randomized, controlled trial of virtual reality-graded exposure therapy for post-traumatic stress disorder in active duty service members with combat-related post-traumatic stress disorder. Cyberpsychology, behavior, and social networking. 2011 Apr 1;14(4):223-9. | Population outside scope: Trials of soldiers on active service |
| 307 | McLay 2017 | McLay RN, Baird A, Webb-Murphy J, Deal W, Tran L, Anson H, Klam W, Johnston S. A randomized, head-to-head study of virtual reality exposure therapy for posttraumatic stress disorder. Cyberpsychology, Behavior, and Social Networking. 2017 Apr 1;20(4):218-24. | Comparison outside protocol |
| 308 | McLean 2016 | McLean, CP.; Fitzgerald, H.; (2016) Treating Posttraumatic Stress Symptoms Among people Living with HIV: a Critical Review of Intervention Trials. Current Psychiatry Reports | Non-systematic review |
| 309 | McPherson 2011 | McPherson, J.; (2011) Does Narrative Exposure Therapy Reduce PTSD in Survivors of Mass Violence? Reseach on Social Work Practice 22(1): 29-42 | Non-systematic review |
| 310 | Meffert 2014 | Meffert SM, Abdo AO, Alla OA, Elmakki YO, Omer AA, Yousif S, Metzler TJ, Marmar CR. A pilot randomized controlled trial of interpersonal psychotherapy for Sudanese refugees in Cairo, Egypt. Psychological Trauma: Theory, Research, Practice, and Policy. 2014 May;6(3):240. | Sample size (N<10/arm) |
| 311 | Meier 2015 | Meier A, McGovern MP, Lambert-Harris C, McLeman B, Franklin A, Saunders EC, Xie H. Adherence and competence in two manual-guided therapies for co-occurring substance use and posttraumatic stress disorders: clinician factors and patient outcomes. The American journal of drug and alcohol abuse. 2015 Nov 2;41(6):527-34. | Subgroup/secondary analysis that is not relevant |
| 312 | Mello 2014 | Mello, PG.; Silva, GR.; Donat, JC.; Kristensen, CH.; (2014) An Update on the Efficacy of Cognitive-Behavioral Therapy, Cognitive Therapy, and Exposure Therapy for Posttraumatic Stress Disorder. The Int J Psychiatry in Med 46(4): 339-357 | Systematic review with no new useable data and any meta-analysis results not appropriate to extract |
| 313 | Mendes 2008 | Mendes, DD.; Mello, MF.; Ventura, P.; Passarela, CDM.; Mari,JDJ.; (2008) A Systematic Review on the Effectiveness of Cognitive Behavioral Therapy for Posttraumatic Stress Disorder. The Int J Psychiatry in Med 38(3): 241-259 | Systematic review with no new useable data and any meta-analysis results not appropriate to extract |
| 314 | Metcalf 2016 | Metcalf, O.; Varker, T.; Forbes, D.; Phelps, A.; Dell, L.; DiBattista, A.; Ralph, N.; O'Donnell, M.; (2016) Efficacy of Fifteen Emerging Interventions for the Treatment of Posttraumatic Stress Disorder: A Systematic Review. 29(1): 88-92 | Systematic review with no new useable data and any meta-analysis results not appropriate to extract |
| 315 | Meyerbroker 2010 | Meyerbroker, K.; Emmelkamp, PMG.; (2010) Virtual reality exposure therapy in anxiety disorders: a systematic review of the process-and-outcome studies. Depresion and Aniety 27(10): 9330944 | Systematic review with no new useable data and any meta-analysis results not appropriate to extract |
| 316 | Mills 2016 | Mills KL, Barrett EL, Merz S, Rosenfeld J, Ewer PL, Sannibale C, Baker AL, Hopwood S, Back SE, Brady KT, Teesson M. Integrated Exposure-Based Therapy for Co-Occurring Post Traumatic Stress Disorder (PTSD) and Substance Dependence: Predictors of Change in PTSD Symptom Severity. Journal of clinical medicine. 2016 Nov 15;5(11):101. | Subgroup/secondary analysis of RCT already included |
| 317 | Minnen 2006 | Minnen AV, Foa EB. The effect of imaginal exposure length on outcome of treatment for PTSD. Journal of Traumatic Stress. 2006 Aug 1;19(4):427-38. | Non-randomised group assignment |
| 318 | Mitchell 2012 | Mitchell KS, Wells SY, Mendes A, Resick PA. Treatment improves symptoms shared by PTSD and disordered eating. Journal of traumatic stress. 2012 Oct 1;25(5):535-42. | Subgroup/secondary analysis that is not relevant |
| 319 | Miyahira 2012 | Miyahira SD, Folen RA, Hoffman HG, Garcia-Palacios A, Spira JL, Kawasaki M. The effectiveness of VR exposure therapy for PTSD in returning warfighters. Annual Review of Cybertherapy and Telemedicine. 2012 Sep 14;181:128-32. | Population outside scope: Trials of soldiers on active service |
| 320 | Mogk 2006 | Mogk, C.; Otte, S.; Reinhold-Hurley, B.; Kroner-Herwig, B.; (2006) Health effects of expressive writing on stressful or traumatic experiences - a meta-analysis. Psychosoc Med, 3 Doc06 | Systematic review with no new useable data and any meta-analysis results not appropriate to extract |
| 321 | Monson 2005 | Monson CM, Rodriguez BF, Warner R. Cognitive‐Behavioral therapy for PTSD in the real world: Do interpersonal relationships make a real difference?. Journal of Clinical Psychology. 2005 Jun 1;61(6):751-61. | Non-randomised group assignment |
| 322 | Moradi 2014 | Moradi AR, Moshirpanahi S, Parhon H, Mirzaei J, Dalgleish T, Jobson L. A pilot randomized controlled trial investigating the efficacy of MEmory Specificity Training in improving symptoms of posttraumatic stress disorder. Behaviour research and therapy. 2014 May 31;56:68-74. | Efficacy or safety data cannot be extracted |
| 323 | Morgan-Lopez 2014 | Morgan‐Lopez AA, Saavedra LM, Hien DA, Campbell AN, Wu E, Ruglass L, Patock‐Peckham JA, Bainter SC. Indirect effects of 12‐session seeking safety on substance use outcomes: Overall and attendance class‐specific effects. The American journal on addictions. 2014 May 1;23(3):218-25. | Subgroup/secondary analysis of RCT already included |
| 324 | Morina 2014 | Mornina, N.; Wicherts, JM.; Lobbrecht, J.; Priebe, S.; (2014) Remission from post-traumatic stress disorder in adults: A systematic review and meta-analysis of long term outcome studies. Clin Psych Rev 34(3): 249-255 | Systematic review with no new useable data and any meta-analysis results not appropriate to extract |
| 325 | Morina 2017a | Mornina, N.; Lancee, J.; Arntz, A.; (2017) Imagery rescripting as a clinical intervention for aversive memories: A meta-analysis. J Behaviour Therapy and Experimental Psychiatry 55: 6-15 | Systematic review with no new useable data and any meta-analysis results not appropriate to extract |
| 326 | Morina 2017c | Morina N, Malek M, Nickerson A, Bryant RA. Meta‐analysis of interventions for posttraumatic stress disorder and depression in adult survivors of mass violence in low‐and middle‐income countries. Depression and anxiety. 2017 Apr 1. | Systematic review with no new useable data and any meta-analysis results not appropriate to extract |
| 327 | Morkved 2014 | Morkved, N.; Hartmann, K.; Aarsheim, LM.; Holen, D.; Milde, AM.; Bomyea, J.; Thorp SR.; (2014) A comparison of Narrative Exposure Therapy and Prolonged Exposure therapy for PTSD. Clinical Psychology Review 34(6): 453-467 | Systematic review with no new useable data and any meta-analysis results not appropriate to extract |
| 328 | Moser 2010 | Moser JS, Cahill SP, Foa EB. Evidence for poorer outcome in patients with severe negative trauma-related cognitions receiving prolonged exposure plus cognitive restructuring: implications for treatment matching in posttraumatic stress disorder. The Journal of nervous and mental disease. 2010 Jan 1;198(1):72-5. | Subgroup/secondary analysis that is not relevant |
| 329 | Motraghi 2013 | Motraghi, TE.; Seim, RW.; Meyer, EC.; Morissette, SB.; (2014) Virtual Reality Exposure Therapy for the Treatment of Posttraumatic Stress Disorder: A Methodological Review Using CONSORT Guidelines. J Clin Psyh 70(3): 197-208 | Systematic review with no new useable data and any meta-analysis results not appropriate to extract |
| 330 | Muss 1991 | Muss D.C. (1991) A New Technique for treating post-traumatic stress disorder. British Journal of Clinical Psychology, Vol 30, pp 91-92. | Non-randomised group assignment |
| 331 | Myers 2015 | Myers US, Browne KC, Norman SB. Treatment engagement: female survivors of intimate partner violence in treatment for PTSD and alcohol use disorder. Journal of dual diagnosis. 2015 Oct 2;11(3-4):238-47. | Subgroup/secondary analysis of RCT already included |
| 332 | Nacasch 2015 | Nacasch N, Huppert JD, Su YJ, Kivity Y, Dinshtein Y, Yeh R, Foa EB. Are 60-minute prolonged exposure sessions with 20-minute imaginal exposure to traumatic memories sufficient to successfully treat PTSD? A randomized noninferiority clinical trial. Behavior therapy. 2015 May 31;46(3):328-41. | Comparison outside protocol |
| 333 | Nakeyar 2016 | Nakeyar, C.; Frewen, PA.; (2016) Evidence-Based Care for Iraqi, Kurdish, and Syrian Asylum Seekers and Refugees of the Syrian Civil War: A systematic review. Canadian Psychology 57(4): 233-245 | Systematic review with no new useable data and any meta-analysis results not appropriate to extract |
| 334 | Nelson 2013 | Nelson, RJ.; (2013) Is Virtual Reality Exposure Therapy Effective for Service Members and Veterans Experiencing Combat-Related PTSD? Traumatology 19(3): 171-178 | Non-systematic review |
| 335 | Nemiro 2015 | Nemiro, A., & Papworth, S. (2015). Efficacy of two evidence-based therapies, emotional freedom techniques (EFT) and cognitive behavioral therapy (CBT) for the treatment of gender violence in the congo: a randomized controlled trial. Energy Psychol, 7(2), 13-25. | Paper unavailable |
| 336 | Nicholl 2009 | Nicholl, C.; Thompson, A.; (2004) The psychological treatment of Post Traumatic Stress Disorder (PTSD) in adult refugees: A review of the current state of psychological therapies. J Ment Health 13(4): 351-362 | Non-systematic review |
| 337 | Nijdam 2015 | Nijdam MJ, Van Amsterdam JG, Gersons BP, Olff M. Dexamethasone-suppressed cortisol awakening response predicts treatment outcome in posttraumatic stress disorder. Journal of affective disorders. 2015 Sep 15;184:205-8. | Subgroup/secondary analysis of RCT already included |
| 338 | Nijdam 2018 | Nijdam MJ, van der Meer CA, van Zuiden M, Dashtgard P, Medema D, Qing Y, Zhutovsky P, Bakker A, Olff M. Turning wounds into wisdom: Posttraumatic growth over the course of two types of trauma-focused psychotherapy in patients with PTSD. Journal of affective disorders. 2018 Feb 1;227:424-31. | Subgroup/secondary analysis of RCT already included |
| 339 | Niles 2012 | Niles BL, Klunk-Gillis J, Ryngala DJ, Silberbogen AK, Paysnick A, Wolf EJ. Comparing mindfulness and psychoeducation treatments for combat-related PTSD using a telehealth approach. Psychological Trauma: Theory, Research, Practice, and Policy. 2012 Sep;4(5):538. | Comparison outside protocol |
| 340 | Nolan 2016 | Nolan CR. Bending without breaking: A narrative review of trauma-sensitive yoga for women with PTSD. Complementary therapies in clinical practice. 2016 Aug 1;24:32-40. | Systematic review with no new useable data and any meta-analysis results not appropriate to extract |
| 341 | Noordik 2010 | Noordik, E.; Van der Kling, JJL.; Klingen, EF.; Nieuwenhuijsen, K.; Van Dijk, FJH.; (2010) Exposure-in-vivo containing interventions to improve work functioning of workers with anxiety disorder: a systematic review. BMC Public Health 10:598 | Systematic review with no new useable data and any meta-analysis results not appropriate to extract |
| 342 | Norman 2007 | Norman S. AUDs and PTSD Treatment for Victims of Partner Violence [NCT00607412]. 2007. Available from: https://clinicaltrials.gov/ct2/show/NCT00607412 [accessed 08.08.2017] | Unpublished (registered on clinical trials.gov and author contacted for full trial report but not provided) |
| 343 | Norton 2007 | Norton, P.; Price, EC.; (2007) A Meta-Analytic Review of Adult Cognitive-Behavioral Treatment Outcome Across the Anxiety Disorders. The J Nervous and Mental Disease 195(6): 521-531 | Systematic review with no new useable data and any meta-analysis results not appropriate to extract |
| 344 | Nose 2017 | Nosè M, Ballette F, Bighelli I, Turrini G, Purgato M, Tol W, Priebe S, Barbui C. Psychosocial interventions for post-traumatic stress disorder in refugees and asylum seekers resettled in high-income countries: Systematic review and meta-analysis. PloS one. 2017 Feb 2;12(2):e0171030. | Systematic review with no new useable data and any meta-analysis results not appropriate to extract |
| 345 | Nosen 2014 | Nosen E, Littlefield AK, Schumacher JA, Stasiewicz PR, Coffey SF. Treatment of co-occurring PTSD–AUD: Effects of exposure-based and non-trauma focused psychotherapy on alcohol and trauma cue-reactivity. Behaviour research and therapy. 2014 Oct 31;61:35-42. | Subgroup/secondary analysis of RCT already included |
| 346 | Nyssen 2016 | Nyssen, OP.; Taylor, SJ.; Wong, G.; Steed, E.; Bourke, L.; Lord, J.; Ross, CA.; Hayman, S.; Field, V.; Higgins, A.; Greenhalgh, T.; Meads, C.; (2016) Does herapeutic writing help people with long-term conditions? Systematic review, realist synthesis and economic considerations. Health Technlogy Assessment 20(27) | Systematic review with no new useable data and any meta-analysis results not appropriate to extract |
| 347 | Oktedalen 2015 | Øktedalen T, Hoffart A, Langkaas TF. Trauma-related shame and guilt as time-varying predictors of posttraumatic stress disorder symptoms during imagery exposure and imagery rescripting—A randomized controlled trial. Psychotherapy Research. 2015 Sep 3;25(5):518-32. | Comparison outside protocol |
| 348 | Olatunji 2010a | Olatunji, B.; Cisler, JM.; Deacon, BJ.; (2010) Efficacy of Cognitive Behavioral Therapy for Anxiety Disorders: A Review of Meta-Analytic Findings. Psychiatric Clinics of North America 33(3): 557-577 | Paper unavailable |
| 349 | Olatunji 2010b | Olatunji, BO.; Cisler, JM.; Tolin, DF.; (2010) A meta-analysis of the influence of comorbidity on treatment outcome in the anxiety disorders. Clin Psych Rew 30(6): 642-654 | Systematic review with no new useable data and any meta-analysis results not appropriate to extract |
| 350 | Olthuis 2016 | Olthuis JV, Wozney L, Asmundson GJ, Cramm H, Lingley-Pottie P, McGrath PJ. Distance-delivered interventions for PTSD: A systematic review and meta-analysis. Journal of anxiety disorders. 2016 Dec 1;44:9-26. | Systematic review with no new useable data and any meta-analysis results not appropriate to extract |
| 351 | Oman 2015 | Oman D, Bormann JE. Mantram repetition fosters self-efficacy in veterans for managing PTSD: A randomized trial. Psychology of Religion and Spirituality. 2015 Feb;7(1):34. | Efficacy or safety data cannot be extracted |
| 352 | Omidi 2013 | Omidi A, Mohammadi A, Zargar F, Akbari H. Efficacy of mindfulness-based stress reduction on mood States of veterans with post-traumatic stress disorder. Archives of trauma research. 2013;1(4):151. | Outcome measures are not validated |
| 353 | Onton 2012 | Onton JA. Placebo-controlled Study of EEG Biofeedback Therapy as an Adjunct Treatment for PTSD, Evaluating Symptoms and EEG Dynamics [NCT01591408]. 2012. Available from: https://clinicaltrials.gov/show/NCT01591408 [accessed 08.08.2017] | Population outside scope: Trials of soldiers on active service |
| 354 | Ost 2003 | Ost, L.G.; Paunovic, N.; Gillow, A.M. (Unpublished) Cognitive behavior therapy in the prevention of chronic PTSD in crime victims. | Paper unavailable |
| 355 | Ost 2009 | Ost, LG.; (2009) Cognitive behaviour therapy for anxiety disorders: 40 years of progress. Nordic J Psychiatry 62(S47): 5-10 | Non-systematic review |
| 356 | Otis 2005 | Otis J. Integrated Treatment for Chronic Pain and PTSD [NCT00127413]. 2005. Available from: https://clinicaltrials.gov/ct2/show/NCT00127413 [accessed 11.05.2017] | Sample size (N<10/arm) |
| 357 | Otis 2010 | Otis J. Intensive Treatment of Chronic Pain and PTSD for OEF/OIF Veterans [NCT01120067]. 2010. Available from: https://clinicaltrials.gov/ct2/show/study/NCT01120067 [accessed 08.08.2017] | Intervention not targeted at PTSD symptoms |
| 358 | O'Toole 2016 | O'Toole, SK.; Solomon, SL.; Bergdahl, SA.; (2016) A Meta-Analysis of Hypnotherapeutic Techniques in the Treatment of PTSD Symptoms. J Traumatic Stress 29(1): 97-100 | Systematic review with no new useable data and any meta-analysis results not appropriate to extract |
| 359 | Otto 2003 | Otto, M.W. et al (2003) Treatment of pharmacotherapy-refratory posttraumatic stress disorder among Cambodian refugees: a pilot study of combination treatment with cognitive-behavior therapy vs sertraline alone. Behaviour Research and Therapy, 41, 1271-1276 | Sample size (N<10/arm) |
| 360 | Ougrin 2011 | Ougrin, D.; (2011) Efficacy of exposure versus cognitive therapy in anxiety disorders: systematic review and meta-analysis. BMC Psychiatry 11:200 | Systematic review with no new useable data and any meta-analysis results not appropriate to extract |
| 361 | Ovaert 2003 | Ovaert, L. B., Cashel, M. L., & Sewell, K. W. (2003). Structured group therapy for posttraumatic stress disorder in incarcerated male juveniles. Am.J.Orthopsychiatry, 73, 294-301. | Non-randomised group assignment |
| 362 | Pacella 2014 | Pacella ML, Feeny N, Zoellner L, Delahanty DL. The impact of PTSD treatment on the cortisol awakening response. Depression and anxiety. 2014 Oct 1;31(10):862-9. | Efficacy or safety data cannot be extracted |
| 363 | Paivio 2010 | Paivio SC, Jarry JL, Chagigiorgis H, Hall I, Ralston M. Efficacy of two versions of emotion-focused therapy for resolving child abuse trauma. Psychotherapy Research. 2010 May 1;20(3):353-66. | Comparison outside protocol |
| 364 | Palic 2011 | Palic, S.; Elklit, A.; (2011) Psychosocial treatment of posttraumatic stress disorder in adult refugees. A systematic review of prospective treatment outcome studies and a critique. J Affective Disorders 131(1-3): 8-23 | Systematic review with no new useable data and any meta-analysis results not appropriate to extract |
| 365 | Pantalon 1998 | Pantalon, M. V. & Motta, R. W. (1998). Effectiveness of anxiety management training in the treatment of posttraumatic stress disorder: a preliminary report. Journal of Behavior Therapy & Experimental Psychiatry, 29, 21-29. | Non-randomised group assignment |
| 366 | Parcesepe 2015 | Parcesepe, AM>; Martin, SL.; Pollock, MD.; Garcia-Moreno, C.; (2015) The effectiveness of mental health interventions for adult female survivors of sexual assault: A systematic review. Aggression and Violent Behvior 25(A): 15-25 | Systematic review with no new useable data and any meta-analysis results not appropriate to extract |
| 367 | Paunovic 2001 | Paunovic, N. & Ost, L. G. (2001). Cognitive-behavior therapy vs exposure therapy in the treatment of PTSD in refugees. Behaviour Research & Therapy, 39, 1183-1197. | Sample size (N<10/arm) |
| 368 | Pease 2009 | Pease, M., Sollom, R., Wayne, P. (2009) Acupuncture for Refugees With Posttraumatic Stress Disorder: Initial Experiences Establishing a Community Clinic, Explore: The Journal of Science and Healing, 5, 51-54 | Non-RCT (no control group) |
| 369 | Peleikis 2005 | Peleikis, DE.; Dahl, AA.; (2005) A systematic review of empirical studies of psychotherapy with women who were sexually abused as children. Psychotherapy Research 15(3): 304-315 | Systematic review with no new useable data and any meta-analysis results not appropriate to extract |
| 370 | Peniston 1991 | Peniston, E.G. & Kulkosky, P.J. (1991) Alpha-theta brainwave neuro-feedback therapy for Vietnam veterans with combat-related post-traumatic stress disorder. Medical Psychotherapy, 4, 47-60 | Outcomes are not of interest |
| 371 | Pigeon 2015 | Pigeon WR, Heffner KL, Crean H, Gallegos AM, Walsh P, Seehuus M, Cerulli C. Responding to the need for sleep among survivors of interpersonal violence: A randomized controlled trial of a cognitive–behavioral insomnia intervention followed by PTSD treatment. Contemporary clinical trials. 2015 Nov 30;45:252-60. | Protocol |
| 372 | Pitman 1996 | Pitman, R. K., Orr, S. P., Altman, B., Longpre, R. E., Poire, R. E., & Macklin, M. L. (1996). Emotional processing during eye movement desensitization and reprocessing therapy of Vietnam veterans with chronic posttraumatic stress disorder. Comprehensive Psychiatry, 37, 419-429. | Non-randomised group assignment |
| 373 | Possemato 2010 | Possemato K, Ouimette P, Geller PA. Internet-based expressive writing for kidney transplant recipients: Effects on posttraumatic stress and quality of life. Traumatology. 2010 Mar;16(1):49-54. | Efficacy or safety data cannot be extracted |
| 374 | Postel 2008 | Postel MG.; de Hann, HA.; De Jong, CAJ.; (2008) E-Therapy for Mental Health Problems: A Systematic Review. Telemedicine and e-Health 14(7):707-714 | Systematic review with no new useable data and any meta-analysis results not appropriate to extract |
| 375 | Powers 2010 | Powers, MB.; Halpern, JM.; Ferenschak, MP.; Gilihan, SJ.; Foa, EB.; (2010) A meta-analytic review of prolonged exposure for posttraumatic stress disorder. Clin Psych Rev 30(6): 635-641 | Systematic review with no new useable data and any meta-analysis results not appropriate to extract |
| 376 | Pratchett 2011 | Pratchett, LC.; Daly, K.; Bierer, LM.; Yehuda, R.; (2011) New approaches to combining pharmacotherapy and psychotherapy for posttraumatic stress disorder. Expert Opinion on Pharmacotherapy 12(15): 2339-2354 | Systematic review with no new useable data and any meta-analysis results not appropriate to extract |
| 377 | Prisco 2013 | Prisco MK, Jecmen MC, Bloeser KJ, McCarron KK, Akhter JE, Duncan AD, Balish MS, Amdur RL, Reinhard MJ. Group auricular acupuncture for PTSD-related insomnia in veterans: a randomized trial. Medical Acupuncture. 2013 Dec 1;25(6):407-22. | Sample size (N<10/arm) |
| 378 | Pruiksma 2016 | Pruiksma, K. E., Cranston, C. C., Rhudy, J. L., Micol, R. L., & Davis, J. L. (2016, December 15). Randomized Controlled Trial to Dismantle Exposure, Relaxation, and Rescripting Therapy (ERRT) for Trauma-Related Nightmares. Psychological Trauma: Theory, Research, Practice, and Policy. Advance online publication. http://dx.doi.org/10.1037/tra0000238 | Comparison outside protocol |
| 379 | Rabe 2006 | Rabe S, Dörfel D, Zöllner T, Maercker A, Karl A. Cardiovascular correlates of motor vehicle accident related posttraumatic stress disorder and its successful treatment. Applied psychophysiology and biofeedback. 2006 Dec 1;31(4):315-30. | Subgroup/secondary analysis of RCT already included |
| 380 | Rabe 2008 | Rabe S, Zoellner T, Beauducel A, Maercker A, Karl A. Changes in brain electrical activity after cognitive behavioral therapy for posttraumatic stress disorder in patients injured in motor vehicle accidents. Psychosomatic medicine. 2008 Jan 1;70(1):13-9. | Subgroup/secondary analysis of RCT already included |
| 381 | Ragsdale 1996 | Ragsdale, K. G., Cox, R. D., Finn, P., & Eisler, R. M. (1996). Effectiveness of short-term specialized inpatient treatment for war-related posttraumatic stress disorder: A role for adventure-based counseling and psychodrama. Journal of Traumatic Stress, 9, 269-283. | Non-randomised group assignment |
| 382 | Rauch 2009 | Rauch SA, Grunfeld TE, Yadin E, Cahill SP, Hembree E, Foa EB. Changes in reported physical health symptoms and social function with prolonged exposure therapy for chronic posttraumatic stress disorder. Depression and anxiety. 2009 Aug 1;26(8):732-8. | Subgroup/secondary analysis of RCT already included |
| 383 | Ready 2010 | Ready DJ, Gerardi RJ, Backscheider AG, Mascaro N, Rothbaum BO. Comparing virtual reality exposure therapy to present-centered therapy with 11 US Vietnam veterans with PTSD. Cyberpsychology, Behavior, and Social Networking. 2010 Feb 1;13(1):49-54. | Sample size (N<10/arm) |
| 384 | Rees 2013 | Rees B, Travis F, Shapiro D, Chant R. Reduction in posttraumatic stress symptoms in Congolese refugees practicing transcendental meditation. Journal of traumatic stress. 2013 Apr 1;26(2):295-8. | Non-randomised group assignment |
| 385 | Reiter 2016 | Reiter, K.; Anderson, S.; Carlsson, J.; (2016) Neurofeedback Treatment and Posttraumatic Stress Disorder: Efectiveness of Neurofeedback on Posttraumatic Stress Disorder and the Optimal Choice of Protocol. J Nervous and Mental Disease 204(2): 69-77 | Systematic review with no new useable data and any meta-analysis results not appropriate to extract |
| 386 | Renfrey 1994 | Renfrey, G. & Spates, C. R. (1994). Eye movement desensitization: a partial dismantling study. Journal of Behavior Therapy & Experimental Psychiatry, 25, 231-239. | Non-randomised group assignment |
| 387 | Renner 2011 | Renner, W., Banninger-Huber, E. & Peltzer, K. (2011) Culture-sensitive and resource oriented peer (CROP) - groups as a community based intervention for trauma survivors: a randomized controlled pilot study with refugees and asylum seekers from Chechnya. The Australasian Journal of Disaster and Trauma Studies. 2011-1:1-13 | Efficacy or safety data cannot be extracted |
| 388 | Resick 1992 | Resick, P.A.; Schnicke, M.K. (1992) Cognitive processing therapy for sexual assault victims. Journal of consulting and clinical psychology, 60, 5, 748-756 | Non-randomised group assignment |
| 389 | Resick 2003 | Resick, P. A., Nishith, P., & Griffin, M. G. (2003). How well does cognitive-behavioral therapy treat symptoms of complex PTSD? An examination of child sexual abuse survivors within a clinical trial. CNS.Spectr, 8, 340-355. | Subgroup/secondary analysis of RCT already included |
| 390 | Resick 2008 | Resick PA, Galovski TE, Uhlmansiek MO, Scher CD, Clum GA, Young-Xu Y. A randomized clinical trial to dismantle components of cognitive processing therapy for posttraumatic stress disorder in female victims of interpersonal violence. Journal of consulting and clinical psychology. 2008 Apr;76(2):243. | Comparison outside protocol |
| 391 | Resick 2012a | Resick PA, Suvak MK, Johnides BD, Mitchell KS, Iverson KM. The impact of dissociation on PTSD treatment with cognitive processing therapy. Depression and Anxiety. 2012 Aug 1;29(8):718-30. | Comparison outside protocol |
| 392 | Resick 2012b | Resick PA, Suvak MK, Johnides BD, Mitchell KS, Iverson KM. The impact of dissociation on PTSD treatment with cognitive processing therapy. Depression and Anxiety. 2012 Aug 1;29(8):718-30. | Subgroup/secondary analysis that is not relevant |
| 393 | Resick 2015 | Resick PA, Wachen JS, Mintz J, Young-McCaughan S, Roache JD, Borah AM, Borah EV, Dondanville KA, Hembree EA, Litz BT, Peterson AL. A randomized clinical trial of group cognitive processing therapy compared with group present-centered therapy for PTSD among active duty military personnel. Journal of consulting and clinical psychology. 2015 Dec;83(6):1058. | Population outside scope: Trials of soldiers on active service |
| 394 | Rhodes 2016 | Rhodes A, Spinazzola J, van der Kolk B. Yoga for adult women with chronic PTSD: A long-term follow-up study. The journal of alternative and complementary medicine. 2016 Mar 1;22(3):189-96. | Efficacy or safety data cannot be extracted |
| 395 | Rhudy 2010 | Rhudy JL, Davis JL, Williams AE, McCabe KM, Bartley EJ, Byrd PM, Pruiksma KE. Cognitive‐behavioral treatment for chronic nightmares in trauma‐exposed persons: assessing physiological reactions to nightmare‐related fear. Journal of clinical psychology. 2010 Apr 1;66(4):365-82. | Outcomes are not of interest |
| 396 | Richards 1994 | Richards, D. A., Lovell, K., & Marks, I. M. (1994). Post-traumatic stress disorder: evaluation of a behavioral treatment program. Journal of Traumatic Stress, 7, 669-680. | Non-randomised group assignment |
| 397 | Rizvi 2009 | Rizvi SL, Vogt DS, Resick PA. Cognitive and affective predictors of treatment outcome in cognitive processing therapy and prolonged exposure for posttraumatic stress disorder. Behaviour Research and Therapy. 2009 Sep 30;47(9):737-43. | Subgroup/secondary analysis of RCT already included |
| 398 | Roberts 2015 | Roberts, NP.; Roberts, PA.; Jones, N.; Bisson, JI.; (2015) Psychological interventions for post-traumatic stress disorder and comorbid substance use disorder: A systematic review and meta-analysis. Clin Psyc Rev 38: 25-38 | Systematic review with no new useable data and any meta-analysis results not appropriate to extract |
| 399 | Roberts 2016 | Roberts, NP.; Roberts, PA.; Jones, N.; Bisson, JI.; (2016) Psychological therapies for post-traumatic stress disorder and comorbid substance use disorder. Cochrane Database of Systematic Reviews. | Systematic review with no new useable data and any meta-analysis results not appropriate to extract |
| 400 | Robjant 2010 | Robjant, K., Fazel, M. (2010) The emerging evidence for Narrative Exposure Therapy: A review, Clinical Psychology Review, 1030-1039 | Non-systematic review |
| 401 | Rodrigues 2011 | Rodrigues, H.; Figueira, I.; Goncalves, R.; Mendlowicz, M.; Macedo, T.; Ventura, P.; (2011) CBT for pharmacotherapy non-remitters - a systetmatic review of a next-step strategy. J Affective Disorders 129(1-3): 219-228 | Systematic review with no new useable data and any meta-analysis results not appropriate to extract |
| 402 | Rogers 1999 | Rogers, S.; Silver, S.M.; Goss, J.; Obenchain, J.; Willis, A.; Whitney, R.L. (1999) A single session, group study of exposure and eye movement desensitization and reprocessing in treating posttraumatic stress disorder among Vietnam war veterens: Preliminary data. Journal of Anxiety Disorders, 13, 1-2, 119-130 | Sample size (N<10/arm) |
| 403 | Ronconi 2015 | Ronconi, JM.; Shiner, B.; Watts, BV.; (2015) A Meta-Analysis of Depresive Symptom Outcomes in Randomized, Controlled Trials for PTSD. J Nervous and Mental Disease 203(7): 522-529. | Systematic review with no new useable data and any meta-analysis results not appropriate to extract |
| 404 | Rosendbaum 2015 | Rosenbaum, S.; Vancampfort, D.; Steel, Z.; Newby, J.; Ward, PB.; Stubbs, B.; (2015) Physical activity in the treatment of Post-traumatic stress disorder: A systematic review and meta-analysis. Psychiatry resarch 230(2): 130-136 | Systematic review with no new useable data and any meta-analysis results not appropriate to extract |
| 405 | Rotaru 2016 | Rotaru,T-S.; Rusu A.; (2016) A Meta-Analysis for the Efficacy of Hypnotherapy in Alleviating PTSD Symptoms. Int J Clin and Expt Hypnosis 64(1): 116-136 | Systematic review with no new useable data and any meta-analysis results not appropriate to extract |
| 406 | Rothbaum (unpublished) | Rothbaum, B, et al. Randomised controlled trial of Exposure, EMDR and waitlist treatment for rape survivors with PTSD. (unpublished) | Paper unavailable |
| 407 | Rothbaum 1997 | Rothbaum, B. O. (1997). A controlled study of eye movement desensitization and reprocessing in the treatment of posttraumatic stress disordered sexual assault victims. Bulletin of the Menninger Clinic, 61, 317-334. | Sample size (N<10/arm) |
| 408 | Rothbaum 2001 | Rothbaum, B. O., Hodges, L. F., Ready, D., Graap, K., & Alarcon, R. D. (2001). Virtual reality exposure therapy for Vietnam veterans with posttraumatic stress disorder. Journal of Clinical Psychiatry, 62, 617-622 | Non-randomised group assignment |
| 409 | Roy 2006 | Roy MJ, Law W, Patt I, Difede J, Rizzo A, Graap K, Rothbaum B. Randomized controlled trial of CBT with virtual reality exposure therapy for PTSD. Annu. Rev. Cyberther. Telemed. 2006;4:39-44. | Protocol |
| 410 | Ruglass 2012 | Ruglass LM, Miele GM, Hien DA, Campbell AN, Hu MC, Caldeira N, Jiang H, Litt L, Killeen T, Hatch-Maillette M, Najavits L. Helping alliance, retention, and treatment outcomes: A secondary analysis from the NIDA clinical trials network women and trauma study. Substance use & misuse. 2012 Apr 17;47(6):695-707. | Subgroup/secondary analysis of RCT already included |
| 411 | Ruglass 2014a | Ruglass LM, Hien DA, Hu MC, Campbell AN. Associations between post‐traumatic stress symptoms, stimulant use, and treatment outcomes: A secondary analysis of NIDA's women and trauma study. The American journal on addictions. 2014 Jan 1;23(1):90-5. | Subgroup/secondary analysis of RCT already included |
| 412 | Ruglass 2014b | Ruglass LM, Hien DA, Hu MC, Campbell AN, Caldeira NA, Miele GM, Chang DF. Racial/ethnic match and treatment outcomes for women with PTSD and substance use disorders receiving community-based treatment. Community mental health journal. 2014 Oct 1;50(7):811-22. | Efficacy or safety data cannot be extracted |
| 413 | Russell (unpublished) | Russell, M.C., Treating combat related stress disorder: A multiple case study utilizing eye movement desensitization and reprocessing procedure with battlefield casualties from the Iraqi war | Non-randomised group assignment |
| 414 | Ryan 2005 | Ryan M, Nitsun M, Gilbert L, Mason H. A prospective study of the effectiveness of group and individual psychotherapy for women CSA survivors. Psychology and Psychotherapy: Theory, Research and Practice. 2005 Dec 1;78(4):465-80. | Comparison outside protocol |
| 415 | Sack 2016 | Sack M, Zehl S, Otti A, Lahmann C, Henningsen P, Kruse J, Stingl M. A Comparison of Dual Attention, Eye Movements, and Exposure Only during Eye Movement Desensitization and Reprocessing for Posttraumatic Stress Disorder: Results from a Randomized Clinical Trial. Psychotherapy and psychosomatics. 2016;85(6):357-65. | Comparison outside protocol |
| 416 | Salcioglu 2007 | Şalcıoğlu E, Başoğlu M, Livanou M. Effects of live exposure on symptoms of posttraumatic stress disorder: The role of reduced behavioral avoidance in improvement. Behaviour Research and Therapy. 2007 Oct 31;45(10):2268-79. | Subgroup/secondary analysis of RCT already included |
| 417 | Salcioglu 2010 | Şalcıoğlu E, Başoğlu M. Control-focused behavioral treatment of earthquake survivors using live exposure to conditioned and simulated unconditioned stimuli. Cyberpsychology, Behavior, and Social Networking. 2010 Feb 1;13(1):13-9. | Non-systematic review |
| 418 | Saunders 2015 | Saunders EC, McGovern MP, Lambert‐Harris C, Meier A, McLeman B, Xie H. The impact of addiction medications on treatment outcomes for persons with co‐occurring PTSD and opioid use disorders. The American journal on addictions. 2015 Dec 1;24(8):722-31. | Subgroup/secondary analysis of RCT already included |
| 419 | Saunders 2016 | Saunders EC, McLeman BM, McGovern MP, Xie H, Lambert-Harris C, Meier A. The influence of family and social problems on treatment outcomes of persons with co-occurring substance use disorders and PTSD. Journal of substance use. 2016 May 3;21(3):237-43. | Subgroup/secondary analysis of RCT already included |
| 420 | Sautter 2016 | Sautter FJ, Glynn SM, Becker‐Cretu JJ, Senturk D, Armelie AP, Wielt DB. Structured Approach Therapy for Combat‐Related PTSD in Returning US Veterans: Complementary Mediation by Changes in Emotion Functioning. Journal of traumatic stress. 2016 Aug 1;29(4):384-7. | Subgroup/secondary analysis of RCT already included |
| 421 | Schaal 2009 | Schaal S, Elbert T, Neuner F. Narrative exposure therapy versus interpersonal psychotherapy. Psychotherapy and psychosomatics. 2009;78(5):298-306. | Non-randomised group assignment |
| 422 | Scher 2017 | Scher CD, Suvak MK, Resick PA. Trauma cognitions are related to symptoms up to 10 years after cognitive behavioral treatment for posttraumatic stress disorder. Psychological trauma: theory, research, practice, and policy. 2017 Nov;9(6):750. | Efficacy or safety data cannot be extracted |
| 423 | Schnurr 2001 | Schnurr, P. P., Friedman, M. J., Lavori, P. W., & Hsieh, F. Y. (2001). Design of Department of Veterans Affairs Cooperative Study no. 420: group treatment of posttraumatic stress disorder. Controlled Clinical Trials, 22, 74-88. | Non-randomised group assignment |
| 424 | Schnurr 2009 | Schnurr PP, Lunney CA, Forshay E, Thurston VL, Chow BK, Resick PA, Foa EB. Sexual function outcomes in women treated for posttraumatic stress disorder. Journal of Women's Health. 2009 Oct 1;18(10):1549-57. | Subgroup/secondary analysis of RCT already included |
| 425 | Schnurr 2012 | Schnurr PP, Lunney CA. Work-related outcomes among female veterans and service members after treatment of posttraumatic stress disorder. Psychiatric Services. 2012 Nov;63(11):1072-9. | Subgroup/secondary analysis of RCT already included |
| 426 | Schnurr 2015 | Schnurr PP, Lunney CA. Differential effects of prolonged exposure on posttraumatic stress disorder symptoms in female veterans. Journal of consulting and clinical psychology. 2015 Dec;83(6):1154. | Subgroup/secondary analysis of RCT already included |
| 427 | Schnurr 2016 | Schnurr PP, Lunney CA. Symptom benchmarks of improved quality of life in PTSD. Depression and anxiety. 2016 Mar 1;33(3):247-55. | Subgroup/secondary analysis of RCT already included |
| 428 | Schnyder 2011 | Schnyder U, Müller J, Maercker A, Wittmann L. Brief eclectic psychotherapy for PTSD: a randomized controlled trial. The Journal of clinical psychiatry. 2011 Apr;72(4):564. | Efficacy or safety data cannot be extracted |
| 429 | Schouten 2014 | Schouten, KA.; de Niet, GJ.; Knipscheer, JW.; Kleber, RJ.; Hutschemaekers, GJM.; (2014) The Effectiveness of Art Therapy in the Treatment of Traumatized Adults. A Systematic Review on Art Therapy and Trauma. Trauma, Viloence and Abuse 16(2): 220-228 | Systematic review with no new useable data and any meta-analysis results not appropriate to extract |
| 430 | Sciarrino 2017 | Sciarrino NA, DeLucia C, O'Brien K, McAdams K. Assessing the Effectiveness of Yoga as a Complementary and Alternative Treatment for Post-Traumatic Stress Disorder: A Review and Synthesis. The Journal of Alternative and Complementary Medicine. 2017 Oct 1;23(10):747-55. | Systematic review with no new useable data and any meta-analysis results not appropriate to extract |
| 431 | Scott 2017 | Scott JC, Harb G, Brownlow JA, Greene J, Gur RC, Ross RJ. Verbal memory functioning moderates psychotherapy treatment response for PTSD-Related nightmares. Behaviour research and therapy. 2017 Apr 30;91:24-32. | Subgroup/secondary analysis that is not relevant |
| 432 | Seal 2010 | Scott K. Enhancing Cognitive Function and Reintegration in Iraq and Afghanistan Veterans With PTSD Using Computer-Based Cognitive Training [NCT01087775]. 2010. Available from: https://clinicaltrials.gov/show/NCT01552278 [accessed 09.08.2017] | Intervention not targeted at PTSD symptoms |
| 433 | Seal 2012 | Seal, K. H., Abadjian, L., McCamish, N., Shi, Y., Tarasovsky, G., Weingardt, K. (2012) A randomized controlled trial of telephone motivational interviewing to enhance mental health treatment engagement in Iraq and Afghanistan veterans, General Hospital Psychiatry, 34, 450-459 | Intervention not targeted at PTSD symptoms |
| 434 | Sebastian 2017 | Sebastian, B.; Nelms, J.; (2017) the Effectiveness of Emotional Freedom Techniques in te Treatment of Posttraumatic Stress Disorder: A Meta-Analysis. EXPOLRE: the J of Science and Healing 13(1): 16-25 | Systematic review with no new useable data and any meta-analysis results not appropriate to extract |
| 435 | Seda 2015 | Seda, G.; Sanchez-Ortuno, MM.; Welsh, CH.; Halbower, AC.; Edinger, JD.; (2015) Comparative Meta-Analysis of Prazosin and Imagery Rehersal Therapy for Nightmare Frequency, Sleep Quality, and Posttraumatic Stress. J Clin Sleep Med 11)1): 11-22 | Systematic review with no new useable data and any meta-analysis results not appropriate to extract |
| 436 | Seehausen 2015 | Seehausen A, Ripper S, Germann G, Hartmann B, Wind G, Renneberg B. Efficacy of a burn-specific cognitive-behavioral group training. Burns. 2015 Mar 31;41(2):308-16. | Non-randomised group assignment |
| 437 | Seidler 2006 | Seidler, GH.; Wagner, FE.; (2006) Comparing the efficacy of EMDR and trauma-focused cognitive-behavioral therapy in the treatment of PTSD: a meta-analytic study. Psychological medicine 36: 1515-1522 | Systematic review with no new useable data and any meta-analysis results not appropriate to extract |
| 438 | Seligowski 2015 | Seligowski, AV.; Lee, DJ.; Bardeen, JR.; Orcutt, HK.; (2015) Emotion Regulation and Posttraumatic Stress Symptoms: A Meta-Analysis. Cognitive Behaviour Therapy 44(2): 87-102 | Systematic review with no new useable data and any meta-analysis results not appropriate to extract |
| 439 | Serfaty 2016 | Serfaty M, Ridgewell A, Drennan V, Kessel A, Brewin CR, Wright A, Laycock G, Blanchard M. Helping Aged Victims of Crime (the HAVoC Study): Common crime, older people and mental illness. Behavioural and cognitive psychotherapy. 2016 Mar;44(2):140-55. | Sample size (N<10/arm) |
| 440 | Servan-Schreiber 2006 | Servan-Schreiber D, Schooler J, Dew MA, Carter C, Bartone P. Eye movement desensitization and reprocessing for posttraumatic stress disorder: a pilot blinded, randomized study of stimulation type. Psychotherapy and Psychosomatics. 2006;75(5):290-7. | Comparison outside protocol |
| 441 | Shapiro 1989 | Shapiro, F. Eye movement desensitization: a new treatment for post-traumatic stress disorder (1989) Journal of Behaviour Therapy and Experimental Psychiatry, 20, 3, 211-217 | Non-RCT (no control group) |
| 442 | Shapiro 2002 | Shapiro, F. & Maxfield, L. (2002). Eye movement desensitization and reprocessing (EMDR): Information processing in the treatment of trauma. Journal of Clinical Psychology, 58, 933-946 | Non-RCT (no control group) |
| 443 | Shemesh 2011 | Shemesh E, Annunziato RA, Weatherley BD, Cotter G, Feaganes JR, Santra M, Yehuda R, Rubinstein D. A randomized controlled trial of the safety and promise of cognitive-behavioral therapy using imaginal exposure in patients with posttraumatic stress disorder resulting from cardiovascular illness. Journal of Clinical Psychiatry. 2011 Feb 1;72(2):168. | Efficacy or safety data cannot be extracted |
| 444 | Sherr 2011 | Sherr, L.; Nagra, N.; Kulubya, G.; Catalan, J.; Clucas, C.; Harding, R.; (2011) HIV infection associated post-traumatic stress disorder and post-traumatic growth - A systematic review. Psychology, Health & Medicine, 16(5): 612-629 | Systematic review with no new useable data and any meta-analysis results not appropriate to extract |
| 445 | Shnaider 2017 | Shnaider P, Sijercic I, Wanklyn SG, Suvak MK, Monson CM. The Role of Social Support in Cognitive-Behavioral Conjoint Therapy for Posttraumatic Stress Disorder. Behavior Therapy. 2017 May 31;48(3):285-94. | Subgroup/secondary analysis of RCT already included |
| 446 | Sijbrandik 2016 | Sijbrandij, M.; Kunovski, I.; Cuijpers, P.; (2016) Effectiveness of internet-delivered cognitive behavioral therapy for posttraumatic stress disorder: A systematic review and meta-analysis. Depression and Anxiety 33: 783-791 | Systematic review with no new useable data and any meta-analysis results not appropriate to extract |
| 447 | Silver 2005 | Silver SM, Rogers S, Knipe J, Colelli G. EMDR therapy following the 9/11 terrorist attacks: a community-based intervention project in New York City. International Journal of Stress Management. 2005 Feb;12(1):29. | Non-randomised group assignment |
| 448 | Skowronek 2014 | Skowronek, IB.; Handler, L.; Guthmann, R.; (2014) Can yoga reduce symtpoms of anxiety and depression? J Fam Prac 63(7): 398-399 | Non-systematic review |
| 449 | Sloan 2011 | Sloan, DM.; Gallagher, MW.; Feinstein, BA.; Lee, DJ.; Pruneau, GM.; (2011) Efficacy of Telehealth Treatments for Posttraumatic Stress-Related Symptoms: A Meta-Analysis. Cognitive Behaviour Therapy 40(2): 111-125 | Systematic review with no new useable data and any meta-analysis results not appropriate to extract |
| 450 | Sloan 2013 | Sloan, DM.; Feinstein, BA.; Gallagher, MW.; Beck, GJ.; Keane, TM.; (2013) Efficacy of Group Treatment for Posttraumatic Stress Disorder Symptoms: A Meta-Analysis. Psychological Trauma: Theory, Research, Practice, and Policy 5(2): 176-183 | Systematic review with no new useable data and any meta-analysis results not appropriate to extract |
| 451 | Slobodin 2015 | Slobodin, O.; De Jong JTVM.; (2015) Mental health interventions for traumatized asylum seekers and refugees: What do we know about their efficacy? Int J Social Psychiartry 61(1): 17-26 | Non-systematic review |
| 452 | Smith 2005 | Smith, MT.; Huany, MI.; Manber, R.; (2005) Cognitive behaviour therapy for chronic insomnia occurring within the context of medical and psychiatric disorders. Clin Psych Rev 25(5): 559-592 | Non-systematic review |
| 453 | Smith 2015 | Smith MJ, Boteler Humm L, Fleming MF, Jordan N, Wright MA, Ginger EJ, Wright K, Olsen D, Bell MD. Virtual reality job interview training for veterans with posttraumatic stress disorder. Journal of vocational rehabilitation. 2015 Jan 1;42(3):271-9. | Outcomes are not of interest |
| 454 | Smyth 2008 | Smyth JM, Hockemeyer JR, Tulloch H. Expressive writing and post‐traumatic stress disorder: Effects on trauma symptoms, mood states, and cortisol reactivity. British Journal of Health Psychology. 2008 Feb 1;13(1):85-93. | Efficacy or safety data cannot be extracted |
| 455 | Soo 2007 | Soo, C.; Tate, RL.; (2007) Psychological treatment for anxiety in people with traumatic brain injury. Cochrane Database of Systematic Reviews. CD005239 | Systematic review with no new useable data and any meta-analysis results not appropriate to extract |
| 456 | Spence 2014 | Spence J, Titov N, Johnston L, Jones MP, Dear BF, Solley K. Internet-based trauma-focused cognitive behavioural therapy for PTSD with and without exposure components: a randomised controlled trial. Journal of affective disorders. 2014 Jun 20;162:73-80. | Comparison outside protocol |
| 457 | Stalker 1999 | Stalker CA, Fry R. A comparison of short-term group and individual therapy for sexually abused women. The Canadian Journal of Psychiatry. 1999 Mar 1;44(2):168-74. | Comparison outside protocol |
| 458 | Stapleton 2006 | Stapleton, JA.; Taylor, S.; Asmundson, GJG.; (2006) Effects of Three PTSD Treatments on Anger and Guilt: Exposure Therapy, Eye Movement Desensitization and Reprocessing, and Relaxation. J Traumatic Stress 19 (1): 19-28 | Outcomes are not of interest |
| 459 | Steenkamp 2015 | Steenkamp, MM.; Litz, BT.; Hoge, CW.; (2015) Psychotherapy for Military-Related PTSD. A Review of Randomized Clinical Trials. JAMA 314(5): 489-500 | Systematic review with no new useable data and any meta-analysis results not appropriate to extract |
| 460 | Steinmetz 2012 | Steinmetz SE, Benight CC, Bishop SL, James LE. My Disaster Recovery: a pilot randomized controlled trial of an Internet intervention. Anxiety, Stress & Coping. 2012 Sep 1;25(5):593-600. | Comparison outside protocol |
| 461 | Stephenson 2017 | Stephenson KR, Simpson TL, Martinez ME, Kearney DJ. Changes in mindfulness and posttraumatic stress disorder symptoms among veterans enrolled in mindfulness‐based stress reduction. Journal of clinical psychology. 2017 Mar 1;73(3):201-17. | Efficacy or safety data cannot be extracted |
| 462 | Stergiopoulos 2011 | Stergiopoulos, E.; Cimo, A.; Cheng, C.; Bonato, S.; Dewa, CS.; (2011) Interventions to improve work outcomes in wrok-related PTSD: a systematic review. BMC Public Health 11:838 | Systematic review with no new useable data and any meta-analysis results not appropriate to extract |
| 463 | Stewart 2009a | Stewart, CL.; Wrobel, TA.; (2009) Evaluation of the Efficacy of Pharmacotherapy and Psychotherapy in Treatment of Combat-Related Post-Traumatic Stress Disorder: A Meta-Analytic Review of Outcome Studies. Military Medicine 174.5: 460-469 | Systematic review with no new useable data and any meta-analysis results not appropriate to extract |
| 464 | Stewart 2009b | Stewart, RE.; Chambless, DL.; (2009) Cognitive-Behavioral Therapy for Adult Anxiety Disorders in Clinical Practice: A Meta-Analysis of Effectiveness Studies. J Consulting and Clinical Psychology 77(4): 595-606 | Systematic review with no new useable data and any meta-analysis results not appropriate to extract |
| 465 | Strauss 2009 | Strauss JL, Calhoun PS, Marx CE. Guided Imagery as a Therapeutic Tool in Post-Traumatic Stress Disorder. InPost-Traumatic Stress Disorder 2009 (pp. 363-373). Humana Press. | Non-RCT (no control group) |
| 466 | Stubbs 2017 | Stubbs, B.; Vancampfort, D.; Rosenbaum, S.; Firth, J.; Cosco, T.; Veronese, N.; Salum, GA.; Schuch, FB.; (2017) An examination of the anxiolytic effects of exercise for people with anxiety and stress-related disorders: A meta-analysis. Psychiatry Research 249: 102-108 | Systematic review with no new useable data and any meta-analysis results not appropriate to extract |
| 467 | Swift 2014 | Swift, JK.; Greenberg, RP.; (2014) A Treatment by Disorder Meta-Analysis of Dropout From Psychotherapy. J Psychotherapy Integration 24(3): 193-207 | Systematic review with no new useable data and any meta-analysis results not appropriate to extract |
| 468 | Tarrier 1999a/1999b | Tarrier, N., Sommerfield, C., Pilgrim, H., & Humphreys, L. (1999). Cognitive therapy or imaginal exposure in the treatment of post- traumatic stress disorder: Twelve-month follow-up. British Journal of Psychiatry, 175, 571-575.  Tarrier, N., Pilgrim, H., Sommerfield, C., Faragher, B., Reynolds, M., Graham, E. et al. (1999). A randomized trial of cognitive therapy and imaginal exposure in the treatment of chronic posttraumatic stress disorder. Journal of Consulting & Clinical Psychology, 67, 13-18. | Comparison outside protocol |
| 469 | Tarrier 2004 | Tarrier N, Sommerfield C. Treatment of chronic PTSD by cognitive therapy and exposure: 5-year follow-up. Behavior Therapy. 2004 May 31;35(2):231-46. | Subgroup/secondary analysis that is not relevant |
| 470 | Taylor 2009 | Taylor, JE.; Harvey, ST.; (2009) Effects of psychotherapy with people who have been sexually assaulted: A meta-analysis. 14(5): 273-285 | Systematic review with no new useable data and any meta-analysis results not appropriate to extract |
| 471 | Taylor 2010 | Taylor, JE.; Harvey, ST.; (2010) A meta-analysis of the effects of psychotherapy with adults sexually abused in childhood. Clinical Psychology Review 30(6): 749-767 | Systematic review with no new useable data and any meta-analysis results not appropriate to extract |
| 472 | Taylor 2014 | Taylor, DJ.; Pruiksma, KE.; (2014) Cognitive and behavioural therapy for insomnia (CBT-I) in psychiatric populations: A systematic review. Int Rev Psychiatry 26(2): 205-213 | Systematic review with no new useable data and any meta-analysis results not appropriate to extract |
| 473 | Taylor 2017 | Taylor M, Petrakis I, Ralevski E. Treatment of alcohol use disorder and co-occurring PTSD. The American journal of drug and alcohol abuse. 2017 Jul 4;43(4):391-401. | Systematic review with no new useable data and any meta-analysis results not appropriate to extract |
| 474 | Teng 2008 | Teng, EJ.; Bailey, SD.; Chaison, AD.; Peterson, NJ.; Hamilton, JD.; Dunn, NJ.; (2008) Treating Comorbid Panic Disorder in Veterans with Posttraumatic Stress Disorder. J Consul and Clin Psych 76(4): 704-710 | Intervention not targeted at PTSD symptoms |
| 475 | Teng 2013 | Teng, EJ.; Hiatt, EL.; McClair, V.; Kunik, ME.; Frueh, BC.; Stanley, MA.; (2013) Efficacy of Posttraumatic Stress Disorder Treatment for Comorbid Panic Disorder: A Critical Review and Future Directions for Treatment Research. Clinical Psychology, Science and Practice 20(3): 268-284 | Systematic review with no new useable data and any meta-analysis results not appropriate to extract |
| 476 | Ter Heide 2011 | Ter Heide FJ, Mooren T, Kleijn W, de Jongh A, Kleber R. EMDR versus stabilisation in traumatised asylum seekers and refugees: Results of a pilot study. European journal of psychotraumatology. 2011 Jan 1;2(1):5881. | Sample size (N<10/arm) |
| 477 | Thompson 1995 | Thompson J, Chung MC, Jackson G, Rosser R. A comparative trial of psychotherapy in the treatment of post‐trauma stress reactions. Clinical Psychology & Psychotherapy. 1995 Oct 1;2(3):168-76. | Intervention outside protocol |
| 478 | Thrasher 2010 | Thrasher S, Power M, Morant N, Marks I, Dalgleish T. Social support moderates outcome in a randomized controlled trial of exposure therapy and (or) cognitive restructuring for chronic posttraumatic stress disorder. The Canadian Journal of Psychiatry. 2010 Mar;55(3):187-90. | Subgroup/secondary analysis of RCT already included |
| 479 | Thunker 2012 | Thünker J, Pietrowsky R. Effectiveness of a manualized imagery rehearsal therapy for patients suffering from nightmare disorders with and without a comorbidity of depression or PTSD. Behaviour Research and Therapy. 2012 Sep 30;50(9):558-64. | Sample size (N<10/arm) |
| 480 | Tirado-Munoz 2014 | Tirado-Munoz, J.; Gilchrist, G.; Farre, M.; Hegarty, K.; Torrens, M.; (2014) The efficay of cognitive behavioural therap and advocacy interventions for women who have experienced intimate partner violence: A systematic review and meta-analysis. Annals of Medicine 46(8): 567-586 | Systematic review with no new useable data and any meta-analysis results not appropriate to extract |
| 481 | Torchalla 2012 | Torchally, I.; Nosen, L.; Rostam, H.; Allen, P.; (2012) Integrated treatment programs for individulas with concurrent substance use disorders and trauma experiences: A systematic review and meta-analysis. J Substance Abuse Treatment 42(1): 65-77 | Systematic review with no new useable data and any meta-analysis results not appropriate to extract |
| 482 | Tran 2016 | Tran, US.; Gregor, B.; (2016) The relative efficacy of bona fide psychotherapies for post-traumatic stress disorder: a meta-analytical evaluatoin of randomized controlled trials. BMC Psychiatry 16:266 | Systematic review with no new useable data and any meta-analysis results not appropriate to extract |
| 483 | Triffleman 2000 | Triffleman, E. (2000). Gender differences in a controlled pilot study of psychosocial treatments in substance dependent patients with post-traumatic stress disorder: Design considerations and outcomes. Alcoholism Treatment Quarterly, 18, 113-126. | Sample size (N<10/arm) |
| 484 | Turner 2014 | Turner, WA.; Casey, LM.; (2014) Outcomes associated with virtual reality in psychological interventions: where are we now? Clinical Psychology Review 34(8): 634-644 | Systematic review with no new useable data and any meta-analysis results not appropriate to extract |
| 485 | Ulmer 2008/2011 | Ulmer CS.Treating Co-Morbid Sleep Difficulties in Veterans With PTSD: A Pilot Study [NCT00734799]. 2008. Available from: https://www.clinicaltrials.gov/ct2/show/NCT00734799 [accessed 09.08.2017]  Ulmer CS, Edinger JD, Calhoun PS. A multi-component cognitive-behavioral intervention for sleep disturbance in veterans with PTSD: a pilot study. Journal of clinical sleep medicine: JCSM: official publication of the American Academy of Sleep Medicine. 2011 Feb 15;7(1):57. | Sample size (N<10/arm) |
| 486 | Uttley 2015 | Uttley, L.; Stevenson, M.; Scope, A.; Rawdin, A.; Sutton, A.; (2015) The clinical and cost effectiveness of group art therapy for people with non-psychotic mental health disorders: a systematic review and cost effectiveness analysis. BMS Psychiatry 15:151 | Systematic review with no new useable data and any meta-analysis results not appropriate to extract |
| 487 | Valentine (unpublished a) | Valentine, P. V. & Smith, T. E. (US). Evaluating traumatic incident reduction therapy with female inmates: A randomized controlled clinical trial. Research on Social Work Practice, 11, Jan-52. | Paper unavailable |
| 488 | Valentine (unpublished b) | Valentine, P. V. (US). Traumatic Incident Reduction I: Traumatized women inmates: Particulars of practice and research. Journal of Offender Rehabilitation, 31, 2000-2015. | Paper unavailable |
| 489 | Vally 2016 | Vally Z, Abrahams L. The effectiveness of peer-delivered services in the management of mental health conditions: a meta-analysis of studies from low-and middle-income countries. International Journal for the Advancement of Counselling. 2016 Dec 1;38(4):330-44. | Systematic review with no new useable data and any meta-analysis results not appropriate to extract |
| 490 | Valmaggia 2016 | Valmaggia, LR.; Latif, L.; Kempton, MJ.; Rus-Calafell, MR.; (2016) Virtual reality in the psychological treatment for mental health problems: An systematic review of recent evidence. Psychiatry Research 236(28): 189-195 | Systematic review with no new useable data and any meta-analysis results not appropriate to extract |
| 491 | Van Dam 2012 | Van Dam, D.; Vedel, E.; Ehring, T.; Emmelkamp, PMG.; (2012) Psychological treatments for concurrent posttraumatic stess disorder and substance use disorder: A systematic review. Clinical Psychology Review 32(3): 202-214 | Systematic review with no new useable data and any meta-analysis results not appropriate to extract |
| 492 | Van Emmerik 2013 | Van Emmerik, AP.; Reijntes, A.; Kamphuis, JH.; (2013) Writing Therapy for Posttraumatic Stress: A Meta-Analysis. Psychotherapy and Psychosomatics 82(2): 82-88 | Systematic review with no new useable data and any meta-analysis results not appropriate to extract |
| 493 | Van Loon 2013 | Van Loon, A.; Van Schaik, A.; Dekker, J.; Beekman, A.; (2013) Bridging the gap for ethnic minority adult outpatients with depression and anxiety disorders by culturally adapted treatments. J Affective Disorders 147(1-3): 9-16 | Systematic review with no new useable data and any meta-analysis results not appropriate to extract |
| 494 | van Minnen 2006 | van Minnen A, Foa EB. The effect of imaginal exposure length on outcome of treatment for PTSD. Journal of Traumatic Stress. 2006 Aug 1;19(4):427-38. | Comparison outside protocol |
| 495 | Van Minnen 2015 | Van Minnen, A.; Zoellner, LA.; Harned, MS.; Mills, K.; (2015) Changes in Comorbid Conditions After Prolonged Exposure for PTSD: a Literature Review. Current Psychiatry Reports 17:17 | Non-systematic review |
| 496 | Van Til 2013 | Van Til, L.; Fikretogul, D.; Pranger, T.; Patten, S.; Wang, J.; Wong, M.; Zamorski, M.; Loisel, P.; Corbiere, M.; Shields, N.; Thompson, J.; Pedler, D. (2013) Work Reintegration for Veterans With Mental Disorders: A Systematic Literature Review to Inform Research. Physical Therapy 93(9): 1163-1174 | Systematic review with no new useable data and any meta-analysis results not appropriate to extract |
| 497 | Van't Hof 2011 | Van't Hof, E.; Stein, DJ.; Van't Hof, E.; Cuijpers, P.; Waheed, W.; (2011) Psychological treatments for depression and anxiety disorders in low- and middle- income countries: a meta-analysis: a review. African Journal of Psychiatry 14(3): 200-207 | Paper unavailable |
| 498 | Vaughan 1994a | Vaughan, K., Armstrong, M. S., Gold, R., O'Connor, N., Jenneke, W., & Tarrier, N. (1994). A trial of eye movement desensitization compared to image habituation training and applied muscle relaxation in post-traumatic stress disorder. Journal of Behavior Therapy & Experimental Psychiatry, 25, 283-291. | Cross-over study and first phase data not available |
| 499 | Vaughan 1994b | Vaughan, K.; Wiese, M.; Gold, R, Tarrier, N. (1994) Eye movement desensitization. Symptom change in post-traumatic stress disorder. British Journal of Psychiatry, 164, 533-541 | Non-randomised group assignment |
| 500 | Verhey 2016 | Verhey, R.; Chibanda, D.; Brakarsh, J.; Seedat, S.; (2016) Psychological interventions for post-traumatic stress disorder in peple living with HIV in Resource poor settings: a systematic review. Tropical Medicine and and Int Health 21(10): 1198-1208 | Systematic review with no new useable data and any meta-analysis results not appropriate to extract |
| 501 | Voshaar 2009 | Voshaar, RCO.; Hendriks, GJ.; Keijsers, G.; Van Balkom, AJ.; (2009) Cognitive behavioural therapy for anxiety disorders in later life. Cochrane Database for Systematic Reveiws. CD007674 | Non-systematic review |
| 502 | Wade 2016 | Wade, D.; Varker, T.; Kartal, D.; Hetrick, S.; O'Donnell, M.; Forbes, D.; (2016) Gender Differences in Outcomes Following Trauma-Focused Interventions for Posttraumatic Stress Disorder: Systematic Review and Meta-Analysis. Psychological Trauma: Theory, Research, Practice and Policy. 8(3): 356-364 | Systematic review with no new useable data and any meta-analysis results not appropriate to extract |
| 503 | Wagner 2016 | Wagner AC, Torbit L, Jenzer T, Landy MS, Pukay‐Martin ND, Macdonald A, Fredman SJ, Monson CM. The Role of Posttraumatic Growth in a Randomized Controlled Trial of Cognitive–Behavioral Conjoint Therapy for PTSD. Journal of traumatic stress. 2016 Aug 1;29(4):379-83. | Subgroup/secondary analysis of RCT already included |
| 504 | Wahbeh 2014 | Wahbeh, H.; Senders, A.; Neuendorf, R.; (2014) Complementary and Alternative Medicine for Posttraumatic Stress Disorder Symtoms. A Systematic Review. J Evidence-Based Complementary and Alternative Medicine 19(3): 161-175 | Systematic review with no new useable data and any meta-analysis results not appropriate to extract |
| 505 | Wang 2013 | Wang Z, Wang J, Maercker A. Chinese My Trauma Recovery, a Web-based intervention for traumatized persons in two parallel samples: randomized controlled trial. Journal of medical Internet research. 2013 Sep;15(9). | Efficacy or safety data cannot be extracted |
| 506 | Watson 1997 | Watson, C. G., Tuorila, J. R., Vickers, K. S., Gearhart, L. P., & Mendez, C. M. (1997). The efficacies of three relaxation regimens in the treatment of PTSD in Vietnam war veterans. Journal of Clinical Psychology, 53, 917-923. | Comparison outside protocol |
| 507 | Watts 2013 | Watts, BV.; Schnurr, PP.; Mayo, L.; Young-Xu, Y.; Weeks, WB.; Friedman, MJ.; (2013) Meta-analysis of the efficacy of treatments for posttraumatic stress disorder. Journal Clinical Psychiatry 74)6): e541-550 | Paper unavailable |
| 508 | Weine 1998 | Weine, S. M., Kulenovic, A. D., Pavkovic, I., & Gibbons, R. (1998). Testimony psychotherapy in Bosnian refugees: A pilot study. American Journal of Psychiatry, 155, 1720-1726. | Non-randomised group assignment |
| 509 | Weine 2008 | Weine S, Kulauzovic Y, Klebic A, Besic S, Mujagic A, Muzurovic J, Spahovic D, Sclove S, Pavkovic I, Feetham S, Rolland J. Evaluating a multiple-family group access intervention for refugees with PTSD. 2008. April; 34(2):149-64. | Intervention not targeted at PTSD symptoms |
| 510 | Wells 2004 | Wells A, Sembi S. Metacognitive therapy for PTSD: A preliminary investigation of a new brief treatment. Journal of Behavior Therapy and Experimental Psychiatry. 2004 Dec 31;35(4):307-18. | Non-RCT (no control group) |
| 511 | Whitworth 2016 | Whitworth JW, Ciccolo JT. Exercise and post-traumatic stress disorder in military veterans: a systematic review. Military medicine. 2016 Sep 1;181(9):953-60. | Systematic review with no new useable data and any meta-analysis results not appropriate to extract |
| 512 | Williams 2013 | Williams JK, Glover DA, Wyatt GE, Kisler K, Liu H, Zhang M. A sexual risk and stress reduction intervention designed for HIV-positive bisexual African American men with childhood sexual abuse histories. Am J Public Health. 2013 Aug;103(8):1476-84. doi: 10.2105/AJPH.2012.301121. | Intervention not targeted at PTSD symptoms |
| 513 | Wilson 1995/1997 | Wilson, S. A., Becker, L. A., & Tinker, R. H. (1995). Eye movement desensitization and reprocessing (EMDR) treatment for psychologically traumatized individuals. Journal of Consulting & Clinical Psychology, 63, 928-937.  Wilson, S.A.; Becker, L.A.; Tinker, R.H. (1997) Fifteen-month follow-up of eye movement desensitization and reprocessing (EMDR) treatment for posttraumatic stress disorder and psychological trauma. Journal of Consulting & Clinical Psychology, 65, 6, 1047-1056 | Efficacy or safety data cannot be extracted |
| 514 | Wilson 1996 | Wilson, D. L., Silver, S. M., Covi, W. G., & Foster, S. (1996). Eye movement desensitization and reprocessing: effectiveness and autonomic correlates. Journal of Behavior Therapy & Experimental Psychiatry, 27, 219-229. | Sample size (N<10/arm) |
| 515 | Wilson 2018 | Wilson, G., Farrell, D., Kiernan, M. The Use of Eye-Movement Desensitization Reprocessing (EMDR) Therapy in Treating Post-traumatic Stress Disorder-A Systematic Narrative Review. Frontiers in Psychology 2018 Jun 6;9:923. | Systematic review with no new useable data and any meta-analysis results not appropriate to extract |
| 516 | Winhusen 2012 | Winhusen T, Winstanley EL, Somoza E, Brigham G. The potential impact of recruitment method on sample characteristics and treatment outcomes in a psychosocial trial for women with co-occurring substance use disorder and PTSD. Drug and alcohol dependence. 2012 Jan 1;120(1):225-8. | Subgroup/secondary analysis of RCT already included |
| 517 | Wisco 2013 | Wisco BE, Sloan DM, Marx BP. Cognitive emotion regulation and written exposure therapy for posttraumatic stress disorder. Clinical Psychological Science. 2013 Oct;1(4):435-42. | Subgroup/secondary analysis of RCT already included |
| 518 | Wisco 2016 | Wisco BE, Baker AS, Sloan DM. Mechanisms of change in written exposure treatment of posttraumatic stress disorder. Behavior therapy. 2016 Jan 31;47(1):66-74. | Subgroup/secondary analysis of RCT already included |
| 519 | Wolf 2016 | Wolf EJ, Lunney CA, Schnurr PP. The influence of the dissociative subtype of posttraumatic stress disorder on treatment efficacy in female veterans and active duty service members. Journal of consulting and clinical psychology. 2016 Jan;84(1):95. | Subgroup/secondary analysis of RCT already included |
| 520 | Woodward 2017 | Woodward E, Hackmann A, Wild J, Grey N, Clark DM, Ehlers A. Effects of psychotherapies for posttraumatic stress disorder on sleep disturbances: Results from a randomized clinical trial. Behaviour research and therapy. 2017 Oct 1;97:75-85. | Subgroup/secondary analysis of RCT already included |
| 521 | Wynn 2015 | Wynn, G. (2015) Complementary and Alternative Medicine Approaches in the Treatment of PTSD, Current Psychiatry Reports, 62 | Non-systematic review |
| 522 | York 2011 | York, A.; Crawford, C.; Walter, JAG.; Jonas, WB.; Coeytaux,R.; (2011) Acupuncture Research in Military and Veteran Populations: A Rapid Evidence Assessment of the Literature. Medical Acupuncture 23(4): 229-236 | Non-systematic review |
| 523 | Yun 2013 | Yun YH, Lee MK, Bae Y, Shon EJ, Shin BR, Ko H, Lee ES, Noh DY, Lim JY, Kim S, Kim SY. Efficacy of a training program for long-term disease-free cancer survivors as health partners: a randomized controlled trial in Korea. Asian Pacific Journal of Cancer Prevention. 2013;14(12):7229-35. | Efficacy or safety data cannot be extracted |
| 524 | Zandberg 2016a | Zandberg LJ, Rosenfield D, McLean CP, Powers MB, Asnaani A, Foa EB. Concurrent treatment of posttraumatic stress disorder and alcohol dependence: Predictors and moderators of outcome. Journal of consulting and clinical psychology. 2016 Jan;84(1):43. | Subgroup/secondary analysis of RCT already included |
| 525 | Zandberg 2016b | Zandberg LJ, Rosenfield D, Alpert E, McLean CP, Foa EB. Predictors of dropout in concurrent treatment of posttraumatic stress disorder and alcohol dependence: Rate of improvement matters. Behaviour research and therapy. 2016 May 31;80:1-9. | Subgroup/secondary analysis of RCT already included |
| 526 | Zang 2013 | Zang, Y., Hunt, N. & Cox, T. (2013). A randomized controlled pilot study: the effectiveness of narrative exposure therapy with adult survivors of the Sichuan earthquake. BMC Psychiatry, 13, 41. | Efficacy or safety data cannot be extracted |
| 527 | Zang 2017 | Zang Y, Yu J, Chazin D, Asnaani A, Zandberg LJ, Foa EB. Changes in coping behavior in a randomized controlled trial of concurrent treatment for PTSD and alcohol dependence. Behaviour research and therapy. 2017 Mar 31;90:9-15. | Subgroup/secondary analysis of RCT already included |
| 528 | Zoellner 1999 | Zoellner LA, Feeny NC, Fitzgibbons LA, Foa EB. Response of African American and Caucasian women to cognitive behavioral therapy for PTSD. Behavior Therapy. 1999 Nov 30;30(4):581-95. | Efficacy or safety data cannot be extracted |
| 529 | Zucker 2009 | Zucker TL, Samuelson KW, Muench F, Greenberg MA, Gevirtz RN. The effects of respiratory sinus arrhythmia biofeedback on heart rate variability and posttraumatic stress disorder symptoms: A pilot study. Applied psychophysiology and biofeedback. 2009 Jun 1;34(2):135. | Comparison outside protocol |

## Included in the systematic review but excluded from the network meta-analysis of psychological treatments for PTSD in adults

|  | **Study ID** | **Reference** | **Reason for exclusion** |
| --- | --- | --- | --- |
| 1 | Abramowitz 2008 | Abramowitz EG, Barak Y, Ben-Avi I, et al. (2008) Hypnotherapy in the treatment of chronic combat-related PTSD patients suffering from insomnia: a randomized, zolpidem-controlled clinical trial. Intl. Journal of Clinical and Experimental Hypnosis 56(3), 270-80 | Hypnotherapy – intervention not of interest |
| 2 | Galovski 2008/2016 | Galovski T (2008) Sleep-directed Hypnosis As A Complement To CPT In Treating PTSD [NCT00725192]. Available from: https://clinicaltrials.gov/ct2/show/NCT00725192 [accessed 02.08.2017]  Galovski TE, Harik JM, Blain LM, et al. (2016) Augmenting cognitive processing therapy to improve sleep impairment in PTSD: A randomized controlled trial. Journal of consulting and clinical psychology 84(2), 167 | Hypnotherapy – intervention not of interest |
| 3 | Bormann 2008 | Bormann JE, Thorp S, Wetherell JL, et al. (2008) A spiritually based group intervention for combat veterans with posttraumatic stress disorder: feasibility study. Journal of Holistic Nursing 26(2), 109-16 | Meditation or Mindfulness-based stress - intervention not of interest |
| 4 | Bormann 2012/2013 | Bormann JE, Liu L, Thorp SR, et al. (2012) Spiritual wellbeing mediates PTSD change in veterans with military-related PTSD. International journal of behavioural medicine 19(4), 496-502  Bormann JE, Thorp SR, Wetherell JL, et al. (2013) Meditation-based mantram intervention for veterans with posttraumatic stress disorder: a randomized trial. Psychological Trauma: Theory, Research, Practice, and Policy 5(3), 259 | Meditation or Mindfulness-based stress - intervention not of interest |
| 5 | Branstrom 2010/2012 | Bränström R, Kvillemo P, Brandberg Y, et al. (2010) Self-report mindfulness as a mediator of psychological well-being in a stress reduction intervention for cancer patients—a randomized study. Annals of behavioural medicine 39(2), 151-61  Bränström R, Kvillemo P and Moskowitz JT (2012) A randomized study of the effects of mindfulness training on psychological well-being and symptoms of stress in patients treated for cancer at 6-month follow-up. International journal of behavioural medicine 19(4), 535-42 | Meditation or Mindfulness-based stress - intervention not of interest |
| 6 | Kearney 2013 | Kearney DJ, McDermott K, Malte C, et al. (2013) Effects of participation in a mindfulness program for veterans with posttraumatic stress disorder: a randomized controlled pilot study. Journal of clinical psychology 69(1), 14-27 | Meditation or Mindfulness-based stress - intervention not of interest |
| 7 | Kearney 2016 | Kearney DJ, Simpson TL, Malte CA, et al. (2016) Mindfulness-based stress reduction in addition to usual care is associated with improvements in pain, fatigue, and cognitive failures among veterans with gulf war illness. The American journal of medicine 129(2), 204-14 | Meditation or Mindfulness-based stress - intervention not of interest |
| 8 | Levine 2005 | Levine EG, Eckhardt J and Targ E (2005) Change in post‐traumatic stress symptoms following psychosocial treatment for breast cancer. Psycho‐Oncology 14(8), 618-35 | Meditation or Mindfulness-based stress - intervention not of interest |
| 9 | Polusny 2015 | Polusny MA, Erbes CR, Thuras P, et al. (2015) Mindfulness-based stress reduction for posttraumatic stress disorder among veterans: A randomized clinical trial. JAMA 314(5), 456-65 | Meditation or Mindfulness-based stress - intervention not of interest |
| 10 | Possemato 2016 | Possemato K, Bergen‐Cico D, Treatman S, et al. (2016) A randomized clinical trial of primary care brief mindfulness training for veterans with PTSD. Journal of clinical psychology 72(3), 179-93 | Meditation or Mindfulness-based stress - intervention not of interest |
| 11 | Wahbeh 2016/Colgan 2016 | Wahbeh H, Goodrich E, Goy E and Oken BS (2016) Mechanistic pathways of mindfulness meditation in combat veterans with posttraumatic stress disorder. Journal of clinical psychology 72(4), 365-83  Colgan DD, Christopher M, Michael P and Wahbeh H (2016) The body scan and mindful breathing among veterans with PTSD: Type of intervention moderates the relationship between changes in mindfulness and post-treatment depression. Mindfulness 7(2), 372-83 | Meditation or Mindfulness-based stress - intervention not of interest |
| 12 | Davis 2012 | Davis LL, Leon AC, Toscano R, et al. (2012) A randomized controlled trial of supported employment among veterans with posttraumatic stress disorder. Psychiatric Services 63(5), 464-70 | Individual placement and support / supported employment - intervention not of interest |
| 13 | Weinstein 2016 | Weinstein N, Khabbaz F and Legate N (2016) Enhancing need satisfaction to reduce psychological distress in Syrian refugees. Journal of consulting and clinical psychology 84(7), 645 | Practical support - intervention not of interest |
| 14 | Wang 2012 | Wang Y, Hu YP, Wang WC, et al. (2012) Clinical studies on treatment of earthquake-caused posttraumatic stress disorder using electroacupuncture. Evidence-Based Complementary and Alternative Medicine 2012 [ID: 431279] | Electroacupuncture – intervention not of interest |
| 15 | Goldstein 2018 | Goldstein LA, Mehling WE, Metzler TJ, et al. (2018) Veterans Group Exercise: A randomized pilot trial of an Integrative Exercise program for veterans with posttraumatic stress. Journal of affective disorders 227, 345-52 | Exercise – intervention not of interest |
| 16 | Rosenbaum 2011/2015 | Rosenbaum S, Nguyen D, Lenehan T, et al. (2011) Exercise augmentation compared to usual care for Post Traumatic Stress Disorder: A Randomised Controlled Trial (The REAP study: R andomised E xercise A ugmentation for P TSD). BMC psychiatry 11(1), 115  Rosenbaum S, Sherrington C and Tiedemann A (2015) Exercise augmentation compared with usual care for post‐traumatic stress disorder: a randomized controlled trial. Acta Psychiatrica Scandinavica 131(5), 350-9 | Exercise – intervention not of interest |
| 17 | Watts 2012 | Watts BV, Landon B, Groft A and Young-Xu Y (2012) A sham controlled study of repetitive transcranial magnetic stimulation for posttraumatic stress disorder. Brain stimulation 5(1), 38-43 | Repetitive transcranial magnetic stimulation - intervention not of interest |
| 18 | Jindani 2015 | Jindani F, Turner N and Khalsa SB (2015) A yoga intervention for posttraumatic stress: A preliminary randomized control trial. Evidence-Based Complementary and Alternative Medicine 2015 | Yoga – intervention not of interest |
| 19 | Mitchell 2014/Dick 2014/Reddy 2014 | Mitchell KS, Dick AM, DiMartino DM, et al. (2014) A pilot study of a randomized controlled trial of yoga as an intervention for PTSD symptoms in women. Journal of Traumatic Stress 27(2), 121-8  Dick AM, Niles BL, Street AE, et al. (2014) Examining mechanisms of change in a yoga intervention for women: the influence of mindfulness, psychological flexibility, and emotion regulation on PTSD symptoms. Journal of clinical psychology 70(12), 1170-82  Reddy S, Dick AM, Gerber MR and Mitchell K (2014) The effect of a yoga intervention on alcohol and drug abuse risk in veteran and civilian women with posttraumatic stress disorder. The Journal of Alternative and Complementary Medicine 20(10), 750-6 | Yoga – intervention not of interest |
| 20 | van der Kolk 2014 | van der Kolk BA, Stone L, West J, et al. (2014) Yoga as an adjunctive treatment for posttraumatic stress disorder: A randomized controlled trial. J Clin Psychiatry 75(6), e559-65 | Yoga – intervention not of interest |
| 21 | Noohi 2017 | Noohi S, Miraghaie AM, Arabi A and Nooripour R (2017) Effectiveness of neuro-feedback treatment with alpha/theta method on PTSD symptoms and their executing function. Biomedical Research 28(5) | Neurofeedback – intervention not of interest |
| 22 | Tan 2011 | Tan G, Dao TK, Farmer L, et al. (2011) Heart rate variability (HRV) and posttraumatic stress disorder (PTSD): A pilot study. Applied Psychophysiology and Biofeedback 36, 27–35 | Biofeedback – intervention not of interest |
| 23 | van der Kolk 2016 | van der Kolk BA, Hodgdon H, Gapen M, et al. (2016) A Randomized Controlled Study of Neurofeedback for Chronic PTSD. PloS one 11(12), e0166752 | Neurofeedback – intervention not of interest |
| 24 | Bisson 2004 | Bisson JI, Shepherd JP, Joy D, et al. (2004) Early cognitive-behavioural therapy for post-traumatic stress symptoms after physical injury. Randomised controlled trial. The British Journal of Psychiatry: The Journal of Mental Science 184, 63–69 | Early treatment (within 3 months from traumatic event) |
| 25 | Jarero 2013 | Jarero I, Amaya C, Givaudan M and Miranda A. (2013) EMDR individual protocol for paraprofessional use: A randomized controlled trial with first responders. Journal of EMDR Practice and Research 7(2), 55-64 | Early treatment (within 3 months from traumatic event) |
| 26 | Johnson 2011 | Johnson DM, Zlotnick C and Perez S (2011) Cognitive behavioral treatment of PTSD in residents of battered women's shelters: results of a randomized clinical trial. Journal of consulting and clinical psychology 79(4), 542 | Early treatment (within 3 months from traumatic event) |
| 27 | Johnson 2016 | Johnson DM, Johnson NL, Perez SK, et al. (2016) Comparison of adding treatment of PTSD during and after shelter stay to standard care in residents of battered women's shelters: results of a randomized clinical trial. Journal of traumatic stress 29(4), 365-73 | Early treatment (within 3 months from traumatic event) |
| 28 | Sahler 2013 | Sahler OJ, Dolgin MJ, Phipps S, et al. (2013) Specificity of problem-solving skills training in mothers of children newly diagnosed with cancer: results of a multisite randomized clinical trial. Journal of Clinical Oncology 31(10), 1329-35 | Early treatment (within 3 months from traumatic event) |
| 29 | Sijbrandij 2007 | Sijbrandij M, Olff M, Reitsma JB, et al. (2007) Treatment of acute posttraumatic stress disorder with brief cognitive behavioral therapy: a randomized controlled trial. American Journal of Psychiatry 164(1), 82-90 | Early treatment (within 3 months from traumatic event) |
| 30 | Turpin 2005 | Turpin, G., Downs, M., Mason, S. (2005) Effectiveness of providing self-help information following acute traumatic injury: Randomised controlled trial, British Journal of Psychiatry, 187, 76-82 | Early treatment (within 3 months from traumatic event) |
| 31 | Neuner 2010 | Neuner F, Kurreck S, Ruf M, et al. (2010) Can asylum-seekers with posttraumatic stress disorder be successfully treated? A randomized controlled pilot study. Cognitive Behavior Therapy 39, 81-91 | Intervention (TF-CBT) added onto TAU |
| 32 | Ruglass 2017/ Hien 2011 | Ruglass LM, Lopez-Castro T, Papini S, et al. (2017) Concurrent treatment with prolonged exposure for co-occurring full or subthreshold posttraumatic stress disorder and substance use disorders: A randomized clinical trial. Psychotherapy and Psychosomatics 86(3), 150-61  Hien D (2011) A Randomized Trial of Concurrent Treatment for PTSD and Substance Dependence [NCT01365247]. Available from: https://clinicaltrials.gov/ct2/show/NCT01365247 [accessed 03.08.2017] | Intervention (TF-CBT) added onto TAU |
| 33 | Pabst 2014 | Pabst A, Schauer M, Bernhardt K, et al. (2014) Evaluation of Narrative Exposure Therapy (NET) for Borderline Personality Disorder with comorbid Posttraumatic Stress Disorder. Clinical Neuropsychiatry 11(4), 108-117 | Intervention (TF-CBT) added onto TAU |
| 34 | Asukai 2010 | Asukai N, Saito A, Tsuruta N, et al. (2010) Efficacy of exposure therapy for Japanese patients with posttraumatic stress disorder due to mixed traumatic events: A randomized controlled study. Journal of traumatic stress 23(6), 744-50 | Intervention (TF-CBT) compared with TAU |
| 35 | Beck 2009 | Beck JG, Coffey SF, Foy DW, et al. (2009) Group cognitive behavior therapy for chronic posttraumatic stress disorder: An initial randomized pilot study. Behavior therapy 40(1), 82-92 | Intervention (TF-CBT) added onto TAU |
| 36 | Brom 1989 | Brom D, Kleber RJ and Defares PB (1989) Brief psychotherapy for posttraumatic stress disorders. Journal of consulting and clinical psychology 57(5), 607 | Interventions (TF-CBT, psychodynamic therapy) added onto TAU |
| 37 | Power 2002 | Power K, McGoldrick T, Brown K, et al. (2002) A controlled comparison of Eye Movement Desensitization and Reprocessing versus exposure plus cognitive restructuring versus waiting list in the treatment of Posttraumatic Stress Disorder. Clinical Psychology and Psychotherapy 9, 299-318 | Interventions (TF-CBT, EMDR) added onto TAU |
| 38 | Resick 2002 | Resick PA, Nishith P, Weaver TL, et al. (2002) A comparison of cognitive-processing therapy with prolonged exposure and a waiting condition for the treatment of chronic posttraumatic stress disorder in female rape victims. Journal of Consulting & Clinical Psychology 70, 867-879 | Intervention (TF-CBT) added onto TAU |
| 39 | Rothbaum 2005 | Rothbaum B, Astin M and Marsteller F (2005) Prolonged exposure versus eye movement desensitization and reprocessing (EMDR) for PTSD rape victims. Journal of Traumatic Stress 18, 607–616 | Interventions (TF-CBT, EMDR) added onto TAU |
| 40 | Foa 2013b | Foa EB, Yusko DA, McLean CP, et al. (2013) Concurrent naltrexone and prolonged exposure therapy for patients with comorbid alcohol dependence and PTSD: a randomized clinical trial. JAMA 310(5), 488-95 | Intervention (TF-CBT) added onto TAU |
| 41 | Sannibale 2013 | Sannibale C, Teesson M, Creamer M, et al. (2013) Randomized controlled trial of cognitive behaviour therapy for comorbid post‐traumatic stress disorder and alcohol use disorders. Addiction 108(8), 1397-410 | Intervention (TF-CBT) added onto TAU |
| 42 | Cook 2010 | Cook JM, Harb GC, Gehrman PR, et al. (2010) Imagery rehearsal for posttraumatic nightmares: a randomized controlled trial. Journal of traumatic stress 23(5), 553-63 | Interventions (TF-CBT, non-TF-CBT) added onto TAU |
| 43 | Wells 2015 | Wells A, Walton D, Lovell K and Proctor D (2015) Metacognitive therapy versus prolonged exposure in adults with chronic post-traumatic stress disorder: A parallel randomized controlled trial. Cognitive Therapy and Research 39(1), 70-80 | Interventions (TF-CBT, metacognitive therapy) added onto TAU |
| 44 | Bohus 2013 | Bohus M, Dyer AS, Priebe K, et al. (2013) Dialectical Behaviour therapy for Post-traumatic Stress Disorder after Childhood Sexual Abuse in Patients with and without Borderline Personality Disorder: A randomised controlled trial. Psychotherapy and psychosomatics 82(4), 221-33 | Intervention (TF-CBT) added onto TAU |
| 45 | Coffey 2016 | Coffey SF, Schumacher JA, Nosen E, et al. (2016) Trauma-focused exposure therapy for chronic posttraumatic stress disorder in alcohol and drug dependent patients: A randomized controlled trial. Psychology of Addictive Behaviors 30(7), 778 | Intervention (TF-CBT) added onto TAU |
| 46 | Dorrepaal 2012 | Dorrepaal E, Thomaes K, Smit JH, et al. (2012) Stabilizing group treatment for complex posttraumatic stress disorder related to child abuse based on psychoeducation and cognitive behavioural therapy: A multisite randomized controlled trial. Psychotherapy and psychosomatics 81(4), 217-25 | Intervention (TF-CBT) added onto TAU |
| 47 | Duffy 2007 | Duffy M, Gillespie K and Clark DM (2007) Post-traumatic stress disorder in the context of terrorism and other civil conflict in Northern Ireland: randomised controlled trial. BMJ 334(7604), 1147 | Intervention (TF-CBT) added onto TAU |
| 48 | Forbes 2012 | Forbes D, Lloyd D, Nixon RD, et al. (2012) A multisite randomized controlled effectiveness trial of cognitive processing therapy for military-related posttraumatic stress disorder. Journal of Anxiety Disorders 26(3), 442-52 | Intervention (TF-CBT) added onto TAU |
| 49 | Maguen 2017 | Maguen S, Burkman K, Madden E, et al. (2017) Impact of killing in war: A randomized, controlled pilot trial. Journal of clinical psychology 73(9), 997-1012 | Intervention (TF-CBT) added onto TAU |
| 50 | Monson 2006 | Monson CM, Schnurr PP, Resick PA, et al. (2006) Cognitive processing therapy for veterans with military-related posttraumatic stress disorder. Journal of Consulting and clinical Psychology 74(5), 898 | Intervention (TF-CBT) added onto TAU |
| 51 | Neuner 2004 | Neuner F, Schauer M, Klaschik C, et al. (2004) A Comparison of Narrative Exposure Therapy, Supportive Counseling, and Psychoeducation for Treating Posttraumatic Stress Disorder in an African Refugee Settlement. Journal of Consulting & Clinical Psychology 72(4), 579-587 | Interventions (TF-CBT and counselling) added onto TAU |
| 52 | Akbarian 2015 | Akbarian F, Bajoghli H, Haghighi M, et al. (2015) The effectiveness of cognitive behavioural therapy with respect to psychological symptoms and recovering autobiographical memory in patients suffering from post-traumatic stress disorder. Neuropsychiatric disease and treatment 11, 395 | Intervention (TF-CBT) added onto TAU |
| 53 | Paunovic 2011 | Paunović N. (2011) Exposure inhibition therapy as a treatment for chronic posttraumatic stress disorder: A controlled pilot study. Psychology 2(06), 605 | Intervention (TF-CBT) added onto TAU |
| 54 | Bass 2013 | Bass JK, Annan J, McIvor Murray S, et al. (2013) Controlled trial of psychotherapy for Congolese survivors of sexual violence. New England Journal of Medicine 368(23), 2182-91 | Intervention (TF-CBT) added onto TAU |
| 55 | Hermenau 2013 | Hermenau K, Hecker T, Schaal S, et al. (2013) Addressing post-traumatic stress and aggression by means of narrative exposure: A randomized controlled trial with ex-combatants in the eastern DRC. Journal of Aggression, Maltreatment and Trauma 22(8), 916-934 | Intervention (TF-CBT) added onto TAU |
| 56 | Hinton 2005 | Hinton DE, Chhean D, Pich V, et al. (2005) A randomized controlled trial of cognitive‐behavior therapy for Cambodian refugees with treatment‐resistant PTSD and panic attacks: A cross‐over design. Journal of traumatic stress 18(6), 617-29 | Intervention (TF-CBT) added onto TAU |
| 57 | Hinton 2009 | Hinton DE, Hofmann SG, Pollack MH and Otto MW (2009) Mechanisms of efficacy of CBT for Cambodian refugees with PTSD: Improvement in emotion regulation and orthostatic blood pressure response. CNS neuroscience & therapeutics 15(3), 255-63 | Intervention (TF-CBT) added onto TAU |
| 58 | Kubany 2003 | Kubany ES, Hill EE and Owens JA (2003) Cognitive trauma therapy for battered women with PTSD: preliminary findings. Journal of Traumatic Stress 16(1), 81-91 | Intervention (TF-CBT) added onto TAU |
| 59 | Kubany 2004 | Kubany ES, Hill E E, Owens JA, et al. (2004) Cognitive trauma therapy for battered women with PTSD (CTT-BW) Journal of Consult. Clin.Psychol 72, 3-18 | Intervention (TF-CBT) added onto TAU |
| 60 | Mills 2012 | Mills KL, Teesson M, Back SE, et al. (2012) Integrated exposure-based therapy for co-occurring posttraumatic stress disorder and substance dependence: a randomized controlled trial. JAMA 308(7), 690-9 | Intervention (TF-CBT) added onto TAU |
| 61 | Mueser 2008 | Mueser KT, Rosenberg SD, Xie H, et al. (2008) A randomized controlled trial of cognitive-behavioral treatment for posttraumatic stress disorder in severe mental illness. Journal of consulting and clinical psychology 76(2), 259 | Intervention (TF-CBT) added onto TAU |
| 62 | Foa 2005 | Foa EB, Hembree EA, Cahill SP, et al. (2005) Randomized trial of prolonged exposure for posttraumatic stress disorder with and without cognitive restructuring: outcome at academic and community clinics. Journal of consulting and clinical psychology 73(5), 953 | Intervention (TF-CBT) added onto TAU |
| 63 | Taylor 2003 | Taylor S, Thordarson DS, Maxfield L, et al. (2003) Comparative efficacy, speed, and adverse effects of three PTSD treatments: exposure therapy, EMDR and relaxation training. Journal of Consulting & Clinical Psychology 71(2), 330-338 | Interventions (TF-CBT, EMDR) added onto TAU |
| 64 | Laugharne 2016 | Laugharne J, Kullack C, Lee CW, et al. (2016) Amygdala volumetric change following psychotherapy for posttraumatic stress disorder. The Journal of neuropsychiatry and clinical neurosciences 28(4), 312-8 | Interventions (TF-CBT, EMDR) added onto TAU |
| 65 | Nijdam 2012 | Nijdam MJ, Gersons BP, Reitsma JB, et al. (2012) Brief eclectic psychotherapy v. eye movement desensitisation and reprocessing therapy for post-traumatic stress disorder: randomised controlled trial. The British Journal of Psychiatry 200(3), 224-31 | Interventions (TF-CBT, EMDR) added onto TAU |
| 66 | Nacasch 2011 | Nacasch N, Foa EB, Huppert JD, et al. (2011) Prolonged exposure therapy for combat-and terror-related posttraumatic stress disorder: a randomized control comparison with treatment as usual. Journal of Clinical Psychiatry 72(9), 1174 | Interventions (TF-CBT, counselling) added onto TAU |
| 67 | Schnurr 2003 | Schnurr PP, Friedman MJ, Foy DW, et al. (2003) Randomized trial of trauma-focused group therapy for posttraumatic stress disorder. Archives of General Psychiatry 60, 481-489 | Interventions (TF-CBT, present-centered therapy) added onto TAU |
| 68 | Schnurr 2007/ Haug 2004 | Schnurr PP, Friedman MJ, Engel CC, et al. (2007) Cognitive behavioral therapy for posttraumatic stress disorder in women: A randomized controlled trial. JAMA 297(8), 820-30  Haug R, Engel CC, Sheliga V, et al. (2004) A randomized clinical trial of cognitive behavioral treatment for PTSD in women veterans [NCT00032617]. Available from: https://clinicaltrials.gov/ct2/show/NCT00032617 [accessed 03.08.2017] | Interventions (TF-CBT, present-centered therapy) added onto TAU |
| 69 | Suris 2013 | Surís A, Link‐Malcolm J, Chard K, et al. (2013) A randomized clinical trial of cognitive processing therapy for veterans with PTSD related to military sexual trauma. Journal of Traumatic Stress 26(1), 28-37 | Interventions (TF-CBT, present-centered therapy) added onto TAU |
| 70 | Rauch 2015 | Rauch SA, King AP, Abelson J, et al. (2015) Biological and symptom changes in posttraumatic stress disorder treatment: a randomized clinical trial. Depression and anxiety 32(3), 204-12 | Interventions (TF-CBT, present-centered therapy) added onto TAU |
| 71 | Sloan 2016b/ 2018 | Sloan DM, Unger W and Beck JG (2016) Cognitive-behavioral group treatment for veterans diagnosed with PTSD: Design of a hybrid efficacy-effectiveness clinical trial. Contemporary clinical trials 47, 123-30  Sloan DM, Unger W, Lee DJ and Beck JG (2018) A randomised controlled trail of cognitive-behavioural group treatment for veterans diagnosed with PTSD. A Randomized Controlled Trial of Group Cognitive Behavioral Treatment for Veterans Diagnosed With Chronic Posttraumatic Stress Disorder. Journal of traumatic stress. https://doi.org/10.1002/jts.22338 | Interventions (TF-CBT, present-centered therapy) added onto TAU |
| 72 | Sloan 2016a/2018 | Sloan DM, Marx BP and Resick PA (2016) Brief treatment for PTSD: A non-inferiority trial. Contemporary clinical trials 48, 76-82  Sloan DM, Marx BP, Lee DJ and Resick PA (2018) A Brief Exposure-Based Treatment vs Cognitive Processing Therapy for Posttraumatic Stress Disorder: A Randomized Noninferiority Clinical Trial. JAMA psychiatry | Interventions (TF-CBT, self-help without support) added onto TAU |
| 73 | Morath 2014 | Morath J, Gola H, Sommershof A, et al. (2014) The effect of trauma-focused therapy on the altered T cell distribution in individuals with PTSD: Evidence from a randomized controlled trial. Journal of Psychiatric Research 54, 1-0 | Intervention (TF-CBT) added onto TAU |
| 74 | Stenmark 2013 | Stenmark H, Catani C, Neuner F, et al. (2013) Treating PTSD in refugees and asylum seekers within the general health care system. A randomized controlled multicenter study. Behaviour research and therapy 51(10), 641-647 | Intervention (TF-CBT) added onto TAU |
| 75 | Hinton 2011 | Hinton DE, Hofmann SG, Rivera E, et al. (2011) Culturally adapted CBT (CA-CBT) for Latino women with treatment-resistant PTSD: A pilot study comparing CA-CBT to applied muscle relaxation. Behaviour research and therapy 49(4), 275-80 | Intervention (TF-CBT) added onto TAU |
| 76 | Margolies 2013 | Margolies SO, Rybarczyk B, Vrana SR, et al. (2013) Efficacy of a cognitive‐behavioral treatment for insomnia and nightmares in Afghanistan and Iraq veterans with PTSD. Journal of Clinical Psychology 69(10), 1026-42 | Intervention (non-TF-CBT) added onto TAU |
| 77 | Zlotnick 1997 | Zlotnick C, Shea TM, Rosen K, et al. (1997) An affect-management group for women with posttraumatic stress disorder and histories of childhood sexual abuse. Journal of Traumatic Stress 10, 425-436 | Intervention (non-TF-CBT) added onto TAU |
| 78 | Talbot 2014 | Talbot LS, Maguen S, Metzler TJ, et al. (2014) Cognitive behavioral therapy for insomnia in posttraumatic stress disorder: a randomized controlled trial. Sleep 37(2), 327-41 | Intervention (non-TF-CBT) added onto TAU |
| 79 | McGovern 2011 | McGovern MP, Lambert-Harris C, Alterman AI, et al. (2011) A randomized controlled trial comparing integrated cognitive behavioral therapy versus individual addiction counseling for co-occurring substance use and posttraumatic stress disorders. Journal of dual diagnosis 7(4), 207-27 | Intervention (non-TF-CBT) added onto TAU |
| 80 | McGovern 2015 | McGovern MP, Lambert‐Harris C, Xie H, et al. (2015) A randomized controlled trial of treatments for co‐occurring substance use disorders and post‐traumatic stress disorder. Addiction 110(7), 1194-204 | Intervention (non-TF-CBT) added onto TAU |
| 81 | Hien 2009 | Hien DA, Wells EA, Jiang H, et al. (2009) Multisite randomized trial of behavioral interventions for women with co-occurring PTSD and substance use disorders. Journal of consulting and clinical psychology 77(4), 607 | Intervention (non-TF-CBT) added onto TAU |
| 82 | Dunn 2007 | Dunn NJ, Rehm LP, Schillaci J, et al. (2007) A randomized trial of self‐management and psychoeducational group therapies for comorbid chronic posttraumatic stress disorder and depressive disorder. Journal of Traumatic Stress 20(3), 221-37 | Intervention (non-TF-CBT) added onto TAU |
| 83 | Himmerich 2016 | Himmerich HD, Willmund G, Zimmermann P, et al. (2016) Serum concentrations of Tnf-A and its soluble receptors during psychotherapy in German soldiers suffering from combat-related PTSD. Psychiatria Danubina 28(3), 293-8 | Intervention (EMDR) added onto TAU |
| 84 | Jensen 1994 | Jensen JA (1994) An investigation of eye movement desensitization and reprocessing (EMD/R) as a treatment for posttraumatic stress disorder (PTSD) symptoms of Vietnam combat veterans. Behavior Therapy 25, 311-325 | Intervention (EMDR) compared with TAU |
| 85 | Brom 2017 | Brom D, Stokar Y, Lawi C, et al. (2017) Somatic Experiencing for Posttraumatic Stress Disorder: A Randomized Controlled Outcome Study. Journal of traumatic stress 30(3), 304-12 | Intervention (combined somatic & cognitive therapies) added onto TAU |
| 86 | Gray 2017 | Gray, R., Budden-Potts, D., & Bourke, F. (2017). Reconsolidation of Traumatic Memories for PTSD: A randomized controlled trial of 74 male veterans. Psychotherapy Research, 1-19. | Intervention (cognitive therapies) added onto TAU |
| 87 | Tylee 2017 | Tylee, D. S., Gray, R., Glatt, S. J., & Bourke, F. (2017). Evaluation of the reconsolidation of traumatic memories protocol for the treatment of PTSD: a randomized, wait-list-controlled trial. Journal of Military, Veteran and Family Health, 3(1), 21-33. | Intervention (cognitive therapies) added onto TAU |
| 88 | Geronilla 2016 | Geronilla L, Minewiser L, Sacramento CA and McWilliams M (2016) EFT (emotional freedom techniques) remediates PTSD and psychological symptoms in veterans: a randomized controlled replication trial. Energy 8(2), 29 | Intervention (combined somatic & cognitive therapies) added onto TAU |
| 89 | Kaslow 2010 | Kaslow NJ, Leiner AS, Reviere S, et al. (2010) Suicidal, abused African American women's response to a culturally informed intervention. Journal of consulting and clinical psychology 78(4), 449 | Intervention (psychoeducation) added onto TAU |
| 90 | van Dam 2013 | van Dam D, Ehring T, et al. (2013) Trauma-focused treatment for posttraumatic stress disorder combined with CBT for severe substance use disorder: a randomized controlled trial. BMC psychiatry 13(1), 172 | Intervention (self-help with support) added onto TAU |
| 91 | Meshberg-Cohen 2014 | Meshberg-Cohen S, Svikis D and McMahon TJ (2014) Expressive writing as a therapeutic process for drug-dependent women. Substance abuse 35(1), 80-8 | Intervention (self-help without support) added onto TAU |
| 92 | Bass 2016 | Bass J, Murray SM, Mohammed TA, et al. (2016) A randomized controlled trial of a trauma-informed support, skills, and psychoeducation intervention for survivors of torture and related trauma in Kurdistan, Northern Iraq. Global Health: Science and Practice 4(3), 452-66 | Intervention (counselling) added onto TAU |
| 93 | Jensen 2016 | Jensen JF, Egerod I, Bestle MH, et al. (2016) A recovery program to improve quality of life, sense of coherence and psychological health in ICU survivors: a multicenter randomized controlled trial, the RAPIT study. Intensive Care Medicine 42, 1733-1743 | Intervention (psychoeducation) added onto TAU |
| 94 | Lange 2003 | Lange A, Rietdijk D, Hudcovicova M, et al. (2003) Interapy: a controlled randomized trial of the standardized treatment of posttraumatic stress through the internet. J.Consult.Clin.Psychol 71, 901-909 | Study did not report PTSD symptom change scores (only endpoint scores) |
| 95 | Knaevelsrud 2007 | Knaevelsrud C and Maercker A (2007) Internet-based treatment for PTSD reduces distress and facilitates the development of a strong therapeutic alliance: a randomized controlled clinical trial. BMC psychiatry 7(1), 13 | Outcomes of interest not reported or not possible to extract |
| 96 | Lieberman 2005 / 2006 / Ghosh Ippen 2011 | Lieberman AF, Van Horn P and Ippen CG (2005) Toward evidence-based treatment: child-parent psychotherapy with preschoolers exposed to marital violence. J Am Acad Child Adolesc Psychiatry 44(12), 1241-8  Lieberman AF, Ippen CG and Van Horn P (2006) Child-parent psychotherapy: 6-month follow-up of a randomized controlled trial. Journal of the American Academy of Child & Adolescent Psychiatry 45(8), 913-8  Ghosh I, Harris WW, Van Horn and Lieberman AF (2011) Traumatic and stressful events in early childhood: can treatment help those at highest risk? Child abuse & neglect 35(7), 504-513 | Interventions (parent child psychotherapy using play, parent training) not linked to the network |
