# Supplementary material for: Cost-effectiveness of psychological treatments for post-traumatic stress disorder in adults
Source: PLoS One. 2020 Apr 30;15(4):e0232245. doi: 10.1371/journal.pone.0232245 (PMC7192458; doi:10.1371/journal.pone.0232245)
Supplement: S7 Appendix — (DOCX) [file pone.0232245.s014.docx]

# **Appendix 7: NMA data files**

## A. Changes in PTSD symptom scores between baseline and treatment endpoint

| t[,1] | y[,1] | sd[,1] | n[,1] | t[,2] | y[,2] | sd[,2] | n[,2] | t[,3] | y[,3] | sd[,3] | n[,3] | t[,4] | y[,4] | sd[,4] | n[,4] | na[] | #Study |
| --- | --- | --- | --- | --- | --- | --- | --- | --- | --- | --- | --- | --- | --- | --- | --- | --- | --- |
| 1 | -2.00 | 9.72 | 24 | 5 | -11.20 | 10.36 | 27 | 6 | -23.10 | 9.47 | 27 | NA | NA | NA | NA | 3 | #Blanchard 2002/2003/2004 |
| 1 | -5.00 | 8.96 | 16 | 6 | -7.69 | 10.53 | 15 | NA | NA | NA | NA | NA | NA | NA | NA | 2 | #Difede 2007b |
| 1 | 0.00 | 5.60 | 11 | 6 | -5.77 | 6.10 | 12 | NA | NA | NA | NA | NA | NA | NA | NA | 2 | #Dunne 2012 |
| 1 | -1.40 | 5.56 | 14 | 6 | -22.10 | 5.89 | 14 | NA | NA | NA | NA | NA | NA | NA | NA | 2 | #Ehlers 2005 |
| 1 | -2.10 | 7.68 | 10 | 6 | -34.55 | 6.55 | 20 | NA | NA | NA | NA | NA | NA | NA | NA | 2 | #Zang 2014 |
| 1 | -0.58 | 2.98 | 17 | 6 | -6.65 | 2.74 | 17 | NA | NA | NA | NA | NA | NA | NA | NA | 2 | #Alghamdi 2015 |
| 1 | -0.10 | 0.35 | 48 | 6 | 0.00 | 0.46 | 52 | 18 | -0.10 | 0.40 | 62 | 19 | 0.00 | 0.47 | 55 | 4 | #Buhmann 2016 |
| 1 | 0.18 | 18.63 | 27 | 6 | -50.03 | 16.93 | 28 | NA | NA | NA | NA | NA | NA | NA | NA | 2 | #Chard 2005 |
| 1 | -15.00 | 19.13 | 24 | 6 | -40.00 | 18.71 | 22 | NA | NA | NA | NA | NA | NA | NA | NA | 2 | #Cloitre 2002 |
| 1 | -6.47 | 17.48 | 31 | 6 | -18.37 | 19.42 | 22 | NA | NA | NA | NA | NA | NA | NA | NA | 2 | #Falsetti 2008 |
| 1 | -1.10 | 6.50 | 14 | 6 | -5.80 | 7.48 | 14 | NA | NA | NA | NA | NA | NA | NA | NA | 2 | #Jung 2013 |
| 1 | -3.22 | 6.22 | 30 | 5 | -14.28 | 9.48 | 30 | 6 | -23.05 | 7.30 | 31 | NA | NA | NA | NA | 3 | #Ehlers 2014 |
| 1 | -2.87 | 8.16 | 24 | 6 | -12.50 | 7.10 | 25 | NA | NA | NA | NA | NA | NA | NA | NA | 2 | #Hollifield 2007 |
| 1 | -2.70 | 16.86 | 10 | 6 | -33.40 | 21.16 | 10 | NA | NA | NA | NA | NA | NA | NA | NA | 2 | #Fecteau 1999 |
| 1 | -0.29 | 0.65 | 66 | 6 | -0.60 | 1.11 | 101 | NA | NA | NA | NA | NA | NA | NA | NA | 2 | #Bolton 2014a |
| 1 | -1.00 | 2.95 | 10 | 6 | -8.00 | 3.85 | 10 | NA | NA | NA | NA | NA | NA | NA | NA | 2 | #Lindauer 2008 |
| 1 | -6.50 | 12.83 | 23 | 6 | -16.80 | 19.64 | 29 | 9 | -20.50 | 14.98 | 22 | NA | NA | NA | NA | 3 | #McDonagh 2005 |
| 1 | -3.46 | 8.16 | 23 | 6 | -12.85 | 7.54 | 41 | NA | NA | NA | NA | NA | NA | NA | NA | 2 | #Pacella 2012 |
| 6 | -19.03 | 7.96 | 89 | 18 | -23.12 | 6.81 | 23 | 19 | -20.94 | 7.16 | 26 | NA | NA | NA | NA | 3 | #Popiel 2015 |
| 18 | 0.40 | 10.10 | 31 | 19 | -5.90 | 7.09 | 34 | NA | NA | NA | NA | NA | NA | NA | NA | 2 | #Rothbaum 2006 |
| 6 | -8.1 | 9.346 | 10 | 8 | -30.36 | 12.45 | 11 | NA | NA | NA | NA | NA | NA | NA | NA | 2 | #Capezzani 2013 |
| 5 | -6.3 | 4.885 | 11 | 6 | -10.38 | 8.05 | 10 | 7 | -13.41 | 4.49 | 14 | NA | NA | NA | NA | 3 | #Foa 1991 |
| 5 | -12.06 | 13.86 | 15 | 6 | -15.18 | 12.90 | 27 | NA | NA | NA | NA | NA | NA | NA | NA | 2 | #Cottraux 2008 |
| 5 | -25.4 | 8.995 | 38 | 6 | -22.70 | 8.70 | 33 | NA | NA | NA | NA | NA | NA | NA | NA | 2 | #Cloitre 2010 |
| 5 | -2.5 | 15.48 | 11 | 6 | -18.30 | 15.57 | 10 | NA | NA | NA | NA | NA | NA | NA | NA | 2 | #Katz 2014 |
| 5 | -3.38 | 13.78 | 42 | 6 | -24.37 | 11.04 | 42 | NA | NA | NA | NA | NA | NA | NA | NA | 2 | #Castillo 2016 |
| 6 | -29.30 | 10.50 | 47 | 9 | -36.30 | 10.88 | 24 | NA | NA | NA | NA | NA | NA | NA | NA | 2 | #Ghafoori 2017 |
| 4 | -18.50 | 18.87 | 13 | 6 | -43.60 | 17.64 | 17 | 10 | -32.60 | 17.27 | 23 | NA | NA | NA | NA | 3 | #Markowitz 2015a |
| 3 | -7.15 | 11.42 | 131 | 6 | -10.01 | 11.38 | 99 | NA | NA | NA | NA | NA | NA | NA | NA | 2 | #Chambers 2014 |
| 6 | -19.56 | 7.37 | 29 | 18 | -13.43 | 6.90 | 20 | NA | NA | NA | NA | NA | NA | NA | NA | 2 | #Echiverri-Cohen 2016 |
| 1 | 2.19 | 23.02 | 22 | 7 | -14.26 | 26.80 | 21 | NA | NA | NA | NA | NA | NA | NA | NA | 2 | #Davis 2007 |
| 1 | -3.48 | 8.76 | 41 | 7 | -12.60 | 7.41 | 39 | NA | NA | NA | NA | NA | NA | NA | NA | 2 | #Krakow 2000 |
| 1 | -3.47 | 20.70 | 23 | 7 | -15.54 | 20.70 | 24 | NA | NA | NA | NA | NA | NA | NA | NA | 2 | #Davis 2011 |
| 1 | -6.20 | 15.42 | 45 | 7 | -23.60 | 16.97 | 48 | 9 | -22.20 | 15.10 | 53 | NA | NA | NA | NA | 3 | #Ford 2011 |
| 2 | -0.20 | 11.16 | 27 | 7 | -5.90 | 11.32 | 33 | NA | NA | NA | NA | NA | NA | NA | NA | 2 | #Nakamura 2017 |
| 1 | -1.40 | 8.18 | 10 | 11 | -32.70 | 12.06 | 10 | NA | NA | NA | NA | NA | NA | NA | NA | 2 | #Wells 2012 |
| 1 | -1.23 | 4.79 | 26 | 8 | -14.72 | 4.41 | 25 | NA | NA | NA | NA | NA | NA | NA | NA | 2 | #Aldahadha 2012 |
| 1 | -2.72 | 11.88 | 14 | 8 | -41.93 | 13.77 | 15 | NA | NA | NA | NA | NA | NA | NA | NA | 2 | #Acarturk 2015 |
| 1 | -3.54 | 13.82 | 49 | 8 | -38.33 | 12.81 | 49 | NA | NA | NA | NA | NA | NA | NA | NA | 2 | #Acarturk 2016 |
| 4 | -8.40 | 12.10 | 12 | 8 | -17.30 | 16.37 | 10 | NA | NA | NA | NA | NA | NA | NA | NA | 2 | #Carlson 1998 |
| 1 | -7.50 | 11.25 | 19 | 8 | -24.60 | 11.43 | 20 | NA | NA | NA | NA | NA | NA | NA | NA | 2 | #Edmond 1999/2004 |
| 1 | -3.35 | 11.51 | 29 | 8 | -14.22 | 12.13 | 18 | NA | NA | NA | NA | NA | NA | NA | NA | 2 | #Yurtsever 2018 |
| 5 | -8.45 | 11.26 | 29 | 8 | -24.64 | 12.30 | 28 | NA | NA | NA | NA | NA | NA | NA | NA | 2 | #Scheck 1998 |
| 7 | -0.11 | 0.41 | 30 | 8 | -0.23 | 0.38 | 32 | NA | NA | NA | NA | NA | NA | NA | NA | 2 | #Ter Heide 2016 |
| 8 | -17.70 | 15.35 | 23 | 12 | -15.80 | 11.20 | 23 | NA | NA | NA | NA | NA | NA | NA | NA | 2 | #Karatzias 2011 |
| 8 | -39.15 | 15.69 | 29 | 18 | -33.23 | 14.66 | 30 | NA | NA | NA | NA | NA | NA | NA | NA | 2 | #van der Kolk 2007 |
| 1 | -5.78 | 12.23 | 16 | 10 | -24.54 | 16.92 | 32 | NA | NA | NA | NA | NA | NA | NA | NA | 2 | #Krupnick 2008 |
| 1 | 0.07 | 0.37 | 38 | 5 | -0.26 | 0.37 | 75 | NA | NA | NA | NA | NA | NA | NA | NA | 2 | #Yeomans 2010 |
| 1 | 0.52 | 7.73 | 25 | 12 | -22.60 | 9.63 | 29 | NA | NA | NA | NA | NA | NA | NA | NA | 2 | #Church 2013/2014 |
| 1 | -13.39 | 30.20 | 74 | 12 | -21.09 | 29.70 | 71 | NA | NA | NA | NA | NA | NA | NA | NA | 2 | #Connolly 2011 |
| 1 | -14.20 | 9.13 | 122 | 12 | -31.90 | 8.43 | 114 | NA | NA | NA | NA | NA | NA | NA | NA | 2 | #Robson 2016 |
| 1 | -0.63 | 6.87 | 19 | 13 | -12.90 | 8.10 | 20 | NA | NA | NA | NA | NA | NA | NA | NA | 2 | #Kent 2011 |
| 2 | -12.95 | 2.51 | 25 | 14 | -3.82 | 1.83 | 27 | NA | NA | NA | NA | NA | NA | NA | NA | 2 | #Bar-Haim 2011/Badura-Brack 2015 study 1 |
| 2 | -8.76 | 2.21 | 24 | 14 | -1.51 | 2.01 | 22 | NA | NA | NA | NA | NA | NA | NA | NA | 2 | #Bar-Haim 2011/Badura-Brack 2015 study 2 |
| 2 | -5.30 | 7.61 | 38 | 14 | -4.90 | 9.09 | 34 | NA | NA | NA | NA | NA | NA | NA | NA | 2 | #Schoorl 2013 |
| 3 | -6.90 | 8.08 | 21 | 15 | -18.68 | 7.99 | 22 | NA | NA | NA | NA | NA | NA | NA | NA | 2 | #Sautter 2015 |
| 1 | -5.68 | 12.12 | 26 | 16 | -23.69 | 10.68 | 28 | NA | NA | NA | NA | NA | NA | NA | NA | 2 | #Ivarsson 2014 |
| 1 | 1.36 | 8.34 | 21 | 16 | -25.34 | 10.50 | 21 | NA | NA | NA | NA | NA | NA | NA | NA | 2 | #Lewis 2017 |
| 1 | -0.48 | 5.97 | 80 | 16 | -10.06 | 8.32 | 79 | NA | NA | NA | NA | NA | NA | NA | NA | 2 | #Knaevelsrud 2015 |
| 1 | -2.85 | 6.38 | 47 | 16 | -7.56 | 6.41 | 47 | NA | NA | NA | NA | NA | NA | NA | NA | 2 | #Knaevelsrud 2017 |
| 16 | -12.50 | 4.40 | 23 | 17 | -12.60 | 5.70 | 28 | NA | NA | NA | NA | NA | NA | NA | NA | 2 | #Littleton 2016 |
| 1 | -15.79 | 14.61 | 14 | 17 | -25.15 | 9.85 | 13 | NA | NA | NA | NA | NA | NA | NA | NA | 2 | #Hirai 2005 |
| 1 | -6.69 | 9.12 | 58 | 17 | -11.26 | 9.37 | 62 | NA | NA | NA | NA | NA | NA | NA | NA | 2 | #Kuhn 2017 |
| 1 | -5.21 | 8.28 | 19 | 17 | -16.00 | 11.81 | 23 | NA | NA | NA | NA | NA | NA | NA | NA | 2 | #Spence 2011 |
| 1 | -2.57 | 6.90 | 29 | 17 | -10.48 | 8.99 | 21 | NA | NA | NA | NA | NA | NA | NA | NA | 2 | #Xu 2016 |
| 1 | -3.56 | 8.74 | 24 | 17 | -6.69 | 7.74 | 25 | NA | NA | NA | NA | NA | NA | NA | NA | 2 | #Miner 2016 |
| 2 | -0.30 | 5.64 | 17 | 17 | -1.32 | 6.43 | 19 | NA | NA | NA | NA | NA | NA | NA | NA | 2 | #Henderson 2007 |
| 2 | -12.30 | 9.10 | 19 | 17 | -17.02 | 10.03 | 42 | NA | NA | NA | NA | NA | NA | NA | NA | 2 | #Truijens 2014 |
| 2 | 1.80 | 4.70 | 23 | 17 | -6.10 | 6.58 | 26 | NA | NA | NA | NA | NA | NA | NA | NA | 2 | #Sloan 2004 |
| 2 | -0.90 | 4.11 | 27 | 17 | -7.54 | 6.72 | 55 | NA | NA | NA | NA | NA | NA | NA | NA | 2 | #Sloan 2007 |
| 2 | -10.20 | 4.77 | 21 | 17 | -8.80 | 5.46 | 21 | NA | NA | NA | NA | NA | NA | NA | NA | 2 | #Sloan 2011 |
| t1, t2, t3, t4 indicate the coded treatment in each trial arm  y1, y2, y3, y4 indicate the mean change in effect in each trial arm  sd1, sd2, sd3, sd4 indicate the standard deviation of the mean change in effect in each trial arm  n1, n2, n3, n4 indicate the number of participants in each trial arm  na indicates number of arms in each trial  NA: non-applicable | | | | | | | | | | | | | | | | | |

Treatment codes: 1. Waitlist; 2. Attention placebo; 3. Psychoeducation; 4. Relaxation; 5. Counselling; 6. TF-CBT; 7. non-TF-CBT; 8. EMDR; 9. Present-centered therapy; 10. IPT; 11. Metacognitive therapy; 12. Combined somatic/cognitive therapies; 13. Resilience-oriented treatment; 14. Attention bias modification; 15. Couple intervention; 16. Self-help with support; 17. Self-help without support; 18. SSRI; 19. TF-CBT + SSRI

CBT: cognitive behavioural therapy; EMDR: eye movement desensitisation and reprocessing; IPT: interpersonal psychotherapy; SSRI: selective serotonin reuptake inhibitor; TF: trauma-focused

## B. Changes in PTSD symptom scores between baseline and 1-4 month follow-up

| t[,1] | y[,1] | sd[,1] | n[,1] | t[,2] | y[,2] | sd[,2] | n[,2] | t[,3] | y[,3] | sd[,3] | n[,3] | na[] | #Study |
| --- | --- | --- | --- | --- | --- | --- | --- | --- | --- | --- | --- | --- | --- |
| 1 | -3.48 | 10.05 | 41 | 5 | -14.4 | 13.75 | 41 | 12 | -13.55 | 15.26 | 44 | 3 | #van Emmerik 2008 |
| 1 | -0.11 | 0.35 | 22 | 5 | -0.24 | 0.44 | 41 | NA | NA | NA | NA | 2 | #Hijazi 2014 |
| 1 | -5.64 | 11.23 | 38 | 5 | -13.69 | 15.69 | 38 | NA | NA | NA | NA | 2 | #Jacob 2014 |
| 1 | -0.32 | 0.90 | 50 | 5 | -0.91 | 0.38 | 99 | NA | NA | NA | NA | 2 | #Weiss 2015 (study 1) |
| 1 | -0.92 | 0.36 | 64 | 5 | -1.08 | 0.57 | 154 | NA | NA | NA | NA | 2 | #Weiss 2015 (study 2) |
| 1 | -10 | 6.90 | 23 | 5 | -13.47 | 7.93 | 41 | NA | NA | NA | NA | 2 | #Pacella 2012 |
| 5 | -19.74 | 17.72 | 11 | 6 | -2.55 | 12.49 | 10 | NA | NA | NA | NA | 2 | #Hensel-Dittmann 2011 |
| 4 | -14.2 | 10.29 | 26 | 5 | -23.3 | 9.52 | 26 | NA | NA | NA | NA | 2 | #Blanchard 2002/2003/2004 |
| 4 | -22.6 | 8.39 | 38 | 5 | -24.2 | 8.69 | 33 | NA | NA | NA | NA | 2 | #Cloitre 2010 |
| 4 | -21.4 | 9.05 | 111 | 5 | -20.5 | 9.33 | 111 | NA | NA | NA | NA | 2 | #Neuner 2008 |
| 4 | -15.33 | 8.90 | 30 | 5 | -22.29 | 8.09 | 31 | NA | NA | NA | NA | 2 | #Ehlers 2014 |
| 5 | -34.3 | 13.84 | 17 | 8 | -23.1 | 11.85 | 17 | NA | NA | NA | NA | 2 | #McDonagh 2005 |
| 3 | -9.21 | 11.77 | 134 | 5 | -8.95 | 11.17 | 110 | NA | NA | NA | NA | 2 | #Chambers 2014 |
| 2 | -2.6 | 12.22 | 27 | 6 | -9.3 | 11.54 | 33 | NA | NA | NA | NA | 2 | #Nakamura 2017 |
| 6 | -25 | 17.11 | 48 | 8 | -24.4 | 15.53 | 53 | NA | NA | NA | NA | 2 | #Ford 2011 |
| 1 | -2.18 | 14.33 | 49 | 7 | -33.82 | 14.10 | 49 | NA | NA | NA | NA | 2 | #Acarturk 2016 |
| 1 | -3.62 | 10.22 | 29 | 7 | -10.50 | 11.65 | 18 | NA | NA | NA | NA | 2 | #Yurtsever 2018 |
| 6 | -0.14 | 0.41 | 32 | 7 | -0.13 | 0.42 | 31 | NA | NA | NA | NA | 2 | #Ter Heide 2016 |
| 7 | -16.2 | 15.17 | 23 | 9 | -16.8 | 12.08 | 23 | NA | NA | NA | NA | 2 | #Karatzias 2011 |
| 1 | -18.89 | 18.17 | 16 | 10 | -26.63 | 20.54 | 32 | NA | NA | NA | NA | 2 | #Krupnick 2008 |
| 3 | -9.04 | 8.06 | 20 | 11 | -21.3 | 8.05 | 21 | NA | NA | NA | NA | 2 | #Sautter 2015 |
| 1 | -4.7 | 10.37 | 30 | 3 | -7.22 | 11.09 | 29 | NA | NA | NA | NA | 2 | #Ghafoori 2016 |
| 1 | -5.13 | 9.63 | 21 | 12 | -28.52 | 11.18 | 21 | NA | NA | NA | NA | 2 | #Lewis 2017 |
| 12 | -15.8 | 4.53 | 20 | 13 | -16.2 | 4.83 | 21 | NA | NA | NA | NA | 2 | #Littleton 2016 |
| 2 | -0.24 | 5.72 | 17 | 13 | -5.95 | 5.64 | 19 | NA | NA | NA | NA | 2 | #Henderson 2007 |
| 1 | -4.47 | 4.04 | 70 | 14 | -3.66 | 6.56 | 72 | NA | NA | NA | NA | 2 | #Kazak 2004 |
| 1 | -7.3 | 8.97 | 28 | 15 | -16.7 | 9.95 | 31 | NA | NA | NA | NA | 2 | #Basoglu 2005 |
| 1 | -13.2 | 13.45 | 15 | 15 | -32.9 | 14.37 | 16 | NA | NA | NA | NA | 2 | #Basoglu 2007 |
| t1, t2, t3 indicate the coded treatment in each trial arm; y1, y2, y3 indicate the mean change in effect in each trial arm  sd1, sd2, sd3 indicate the standard deviation of the mean change in effect in each trial arm; n1, n2, n3 indicate the number of participants in each trial arm  na indicates number of arms in each trial; NA: non-applicable | | | | | | | | | | | | | |

Treatment codes: 1. Waitlist; 2. Attention placebo; 3. Psychoeducation; 4. Counselling; 5. TF-CBT; 6. non-TF-CBT; 7. EMDR; 8. Present-centered therapy; 9. Combined somatic/cognitive therapies; 10. IPT; 11. Couple intervention; 12. Self-help with support; 13. Self-help without support; 14. Family therapy; 15. Behavioural therapy

CBT: cognitive behavioural therapy; EMDR: eye movement desensitisation and reprocessing; IPT: interpersonal psychotherapy; TF: trauma-focused

## C. Dichotomous remission at treatment endpoint

| t[,1] | r[,1] | n[,1] | t[,2] | r[,2] | n[,2] | t[,3] | r[,3] | n[,3] | na[] | #Study |
| --- | --- | --- | --- | --- | --- | --- | --- | --- | --- | --- |
| 1 | 5 | 21 | 5 | 10 | 21 | 6 | 16 | 21 | 3 | #Blanchard 2002/2003/2004 |
| 1 | 8 | 29 | 6 | 24 | 28 | 14 | 6 | 28 | 3 | #Ehlers 2003 |
| 1 | 0 | 14 | 6 | 10 | 14 | NA | NA | NA | 2 | #Ehlers 2005 |
| 1 | 0 | 11 | 6 | 5 | 13 | NA | NA | NA | 2 | #Fecteau 1999 |
| 1 | 3 | 12 | 6 | 10 | 12 | NA | NA | NA | 2 | #Lindauer 2005 |
| 1 | 7 | 35 | 6 | 26 | 36 | NA | NA | NA | 2 | #Chard 2005 |
| 1 | 6 | 27 | 6 | 17 | 31 | NA | NA | NA | 2 | #Cloitre 2002 |
| 1 | 5 | 31 | 6 | 17 | 29 | NA | NA | NA | 2 | #Falsetti 2008 |
| 1 | 10 | 20 | 6 | 20 | 22 | NA | NA | NA | 2 | #Gersons 2000 |
| 1 | 1 | 17 | 6 | 5 | 17 | NA | NA | NA | 2 | #Jung 2013 |
| 1 | 2 | 10 | 6 | 8 | 10 | NA | NA | NA | 2 | #Lindauer 2008 |
| 1 | 4 | 23 | 6 | 8 | 29 | 10 | 7 | 22 | 3 | #McDonagh 2005 |
| 1 | 1 | 30 | 5 | 6 | 30 | 6 | 16 | 31 | 3 | #Ehlers 2014 |
| 1 | 4 | 27 | 6 | 9 | 28 | NA | NA | NA | 2 | #Hollifield 2007 |
| 6 | 72 | 114 | 15 | 13 | 57 | 16 | 20 | 57 | 3 | #Popiel 2015 |
| 6 | 1 | 10 | 8 | 10 | 11 | NA | NA | NA | 2 | #Capezzani 2013 |
| 5 | 1 | 14 | 6 | 4 | 14 | 7 | 7 | 17 | 3 | #Foa 1991 |
| 5 | 6 | 18 | 6 | 23 | 40 | NA | NA | NA | 2 | #Bryant 2003a |
| 5 | 4 | 29 | 6 | 10 | 31 | NA | NA | NA | 2 | #Cottraux 2008 |
| 5 | 18 | 38 | 6 | 20 | 33 | NA | NA | NA | 2 | #Cloitre 2010 |
| 3 | 5 | 32 | 6 | 7 | 38 | 9 | 8 | 40 | 3 | #Markowitz 2015a |
| 1 | 0 | 45 | 7 | 10 | 48 | 10 | 8 | 53 | 3 | #Ford 2011 |
| 1 | 3 | 49 | 8 | 30 | 49 | NA | NA | NA | 2 | #Acarturk 2016 |
| 1 | 3 | 29 | 8 | 10 | 18 | NA | NA | NA | 2 | #Yurtsever 2018 |
| 3 | 16 | 25 | 8 | 17 | 25 | NA | NA | NA | 2 | #Carletto 2016 |
| 8 | 8 | 29 | 15 | 4 | 30 | NA | NA | NA | 2 | #van der Kolk 2007 |
| 1 | 7 | 29 | 11 | 47 | 49 | NA | NA | NA | 2 | #Steinert 2017 |
| 1 | 2 | 16 | 9 | 16 | 32 | NA | NA | NA | 2 | #Krupnick 2008 |
| 1 | 4 | 20 | 12 | 13 | 20 | NA | NA | NA | 2 | #Monson 2008/2012 |
| 4 | 2 | 28 | 12 | 15 | 29 | NA | NA | NA | 2 | #Sautter 2015 |
| 1 | 14 | 31 | 13 | 22 | 31 | NA | NA | NA | 2 | #Ivarsson 2014 |
| 1 | 5 | 75 | 13 | 31 | 74 | NA | NA | NA | 2 | #Knaevelsrud 2015 |
| 1 | 3 | 24 | 14 | 21 | 22 | NA | NA | NA | 2 | #Sloan 2012 |
| 2 | 5 | 23 | 14 | 7 | 24 | NA | NA | NA | 2 | #Sloan 2011 |
| t1, t2, t3 indicate the coded treatment in each trial arm; r1, r2, r3 indicate the number of events in each trial arm; n1, n2, n3 indicate the number of participants in each trial arm  na indicates number of arms in each trial | | | | | | | | | | |

Treatment codes: 1. Waitlist; 2. Attention placebo; 3. Relaxation; 4. Psychoeducation; 5. Counselling; 6. TF-CBT; 7. non-TF-CBT; 8. EMDR; 9. IPT; 10. Present-centered therapy; 11. Psychodynamic therapy; 12. Couple intervention; 13. Self-help with support; 14. Self-help without support; 15. SSRI; 16. TF-CBT + SSRI

CBT: cognitive behavioural therapy; EMDR: eye movement desensitisation and reprocessing; IPT: interpersonal psychotherapy; SSRI: selective serotonin reuptake inhibitor; TF: trauma-focused
