# Supplementary material for: Cost-effectiveness of psychological treatments for post-traumatic stress disorder in adults
Source: PLoS One. 2020 Apr 30;15(4):e0232245. doi: 10.1371/journal.pone.0232245 (PMC7192458; doi:10.1371/journal.pone.0232245)

# **Appendix 8: Risk of bias of studies included in the NMA**

## Risk of bias graph: reviewer’s judgements about each risk of bias item presented as percentages across all included studies


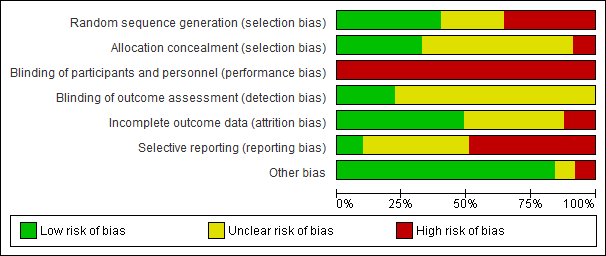


## Risk of bias graph: reviewer’s judgements about each risk of bias item presented by study.


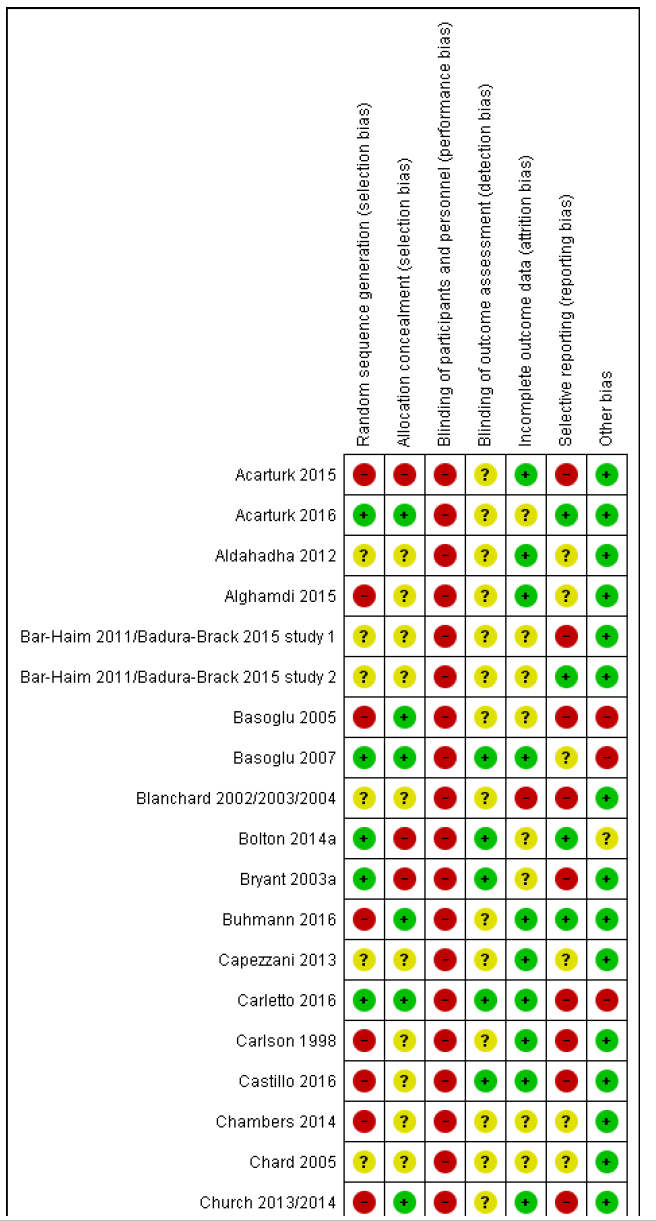


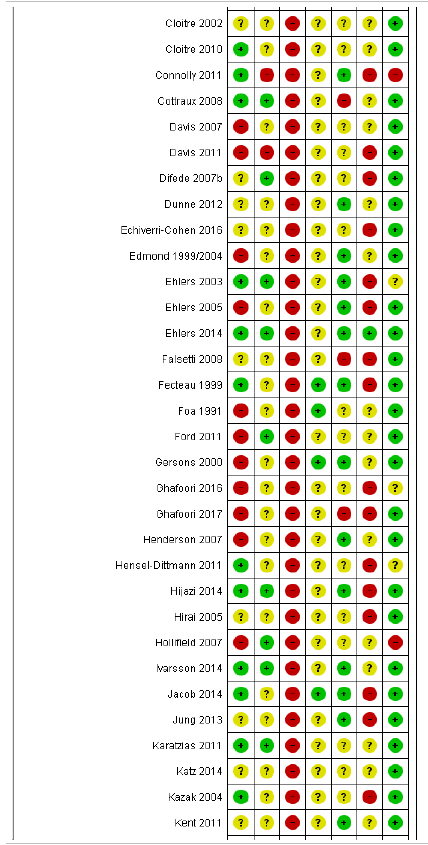

Supplement: S8 Appendix — (DOCX) [file pone.0232245.s015.docx]
