# Supplementary material for: Cost-effectiveness of psychological treatments for post-traumatic stress disorder in adults
Source: PLoS One. 2020 Apr 30;15(4):e0232245. doi: 10.1371/journal.pone.0232245 (PMC7192458; doi:10.1371/journal.pone.0232245)
Supplement: S9 Appendix — (DOCX) [file pone.0232245.s016.docx]

# **Appendix 9: Model fit statistics**

## A. Changes in PTSD symptom scores between baseline and treatment endpoint

Convergence was satisfactory for both fixed and random effects after 20,000 iterations, and the models were compared using results based on samples from a further 40,000 iterations on two chains. The random effects model provided a better fit over the fixed effect model; however, the between-trial standard deviation (posterior median sd 0.93, 95% CrI 0.77 to 1.15) was high when compared with the size of the intervention effect estimates.

| Model | Between Study Heterogeneity - Standard Deviation | | | Residual deviance^a^ | DIC^b^ |
| --- | --- | --- | --- | --- | --- |
|  | Posterior  mean | Posterior  median | 95% CrI |  |  |
| Fixed effect - consistency | - | | | 893.4 | 1381.00 |
| Random effects - consistency | 0.94 | 0.93 | 0.77 - 1.15 | 151.6 | 695.68 |
| Random effects - inconsistency | 1.03 | 1.02 | 0.82 - 1.29 | 151.1 | 697.11 |
| ^a^ Posterior mean residual deviance compared to 151 total data points  ^b^ Deviance information criteria (DIC) – lower values preferred  CrI: credible intervals | | | | | |

## B. Changes in PTSD symptom scores between baseline and 1-4 month follow-up

Convergence was satisfactory for both fixed and random effects after 20,000 iterations, and the models were compared using results based on samples from a further 40,000 iterations on two chains. The random effects model provided a better fit over the fixed effect model; however, moderate-to-high between trial heterogeneity (posterior median sd 0.59, 95% CrI 0.38 to 0.95) was observed relative to the size of the intervention effect estimates.

| Model | Between Study Heterogeneity - Standard Deviation | | | Residual deviance^a^ | DIC^b^ |
| --- | --- | --- | --- | --- | --- |
|  | Posterior mean | Posterior median | 95% CrI |  |  |
| Fixed effect – consistency | - | | | 136.00 | 300.24 |
| Random effects - consistency | 0.61 | 0.59 | 0.38 - 0.95 | 57.36 | 234.62 |
| Random effects - inconsistency | 0.58 | 0.56 | 0.33 - 1.00 | 57.26 | 235.08 |
| ^a^ Posterior mean residual deviance compared to 57 total data points  ^b^ Deviance information criteria (DIC) – lower values preferred  CrI: credible intervals | | | | | |

## C. Dichotomous remission at treatment endpoint

Convergence was satisfactory for both fixed and random effects after 20,000 iterations, and the models were compared using results based on samples from a further 40,000 iterations on two chains. The random effects model provided a better fit over the fixed effect model; however, high between trial heterogeneity (posterior median sd 1.05, 95% CrI 0.60 to 1.68) was observed relative to the size of the intervention effect estimates.

| Model | Between Study Heterogeneity - Standard Deviation | | | Residual deviance^a^ | DIC^b^ |
| --- | --- | --- | --- | --- | --- |
|  | Posterior  mean | Posterior  median | 95% CrI |  |  |
| Fixed effect - consistency | - | | | 120.10 | 410.60 |
| Random effects - consistency | 1.07 | 1.05 | 0.60 - 1.68 | 79.29 | 387.20 |
| Random effects - inconsistency | 1.05 | 1.01 | 0.54 - 1.76 | 78.95 | 388.55 |
| ^a^ Posterior mean residual deviance compared to 76 total data points  ^b^ Deviance information criteria (DIC) – lower values preferred  CrI: credible intervals | | | | | |
