# Supplementary material for: Cost-effectiveness of psychological treatments for post-traumatic stress disorder in adults
Source: PLoS One. 2020 Apr 30;15(4):e0232245. doi: 10.1371/journal.pone.0232245 (PMC7192458; doi:10.1371/journal.pone.0232245)
Supplement: S10 Appendix — (DOCX) [file pone.0232245.s017.docx]

# **Appendix 10: Inconsistency checks**

## A. Changes in PTSD symptom scores between baseline and treatment endpoint

No evidence of inconsistency was found through comparison of the consistency and inconsistency random effects models, as there were no meaningful differences between the fit of the random effects consistency and inconsistency models, and the between-study standard deviation was smaller in the consistency model (Appendix 9). Further checks for inconsistency using the node-splitting method (random effects model) did not find any evidence of inconsistency between the direct and indirect estimates. However, the difference between the direct and indirect evidence contributing to the pooled estimate of TF-CBT + SSRI versus waitlist is worth noting. Buhmann 2016 is the only study directly comparing these treatments. However the inconsistency model does not make any considerable improvements in the prediction of data points in this study, compared with the consistency model. The only study with data points that were slightly better predicted by the inconsistency model compared with the NMA model was Zang 2014. This study compared TF-CBT versus waitlist but the pooled direct and indirect estimates for this comparison are in agreement (see Deviance plot in Figure below).

### Deviance contributions for the random effects consistency and inconsistency models


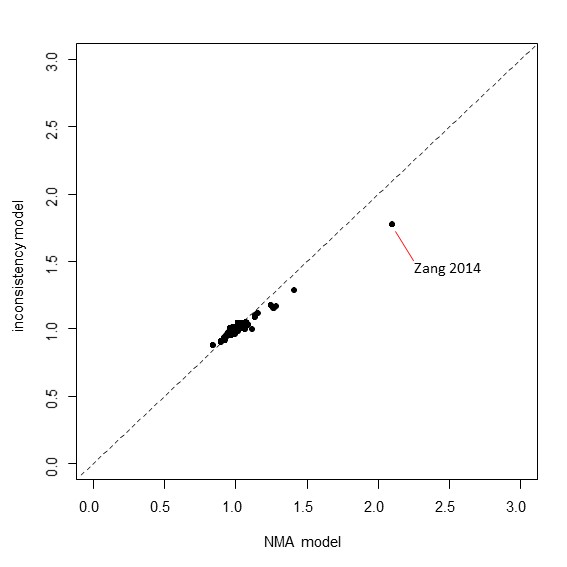


### Summary of node-splitting results

| Node split model | Heterogeneity (SD) | | Residual deviance | Data points^a^ | p-value^b^ |
| --- | --- | --- | --- | --- | --- |
|  | Median | 95% CrI |  |  |  |
| non-TF-CBT vs. Waitlist | 0.86 | (0.70, 1.08) | 82.35 | 79 | 0.32 |
| EMDR vs. Waitlist | 0.86 | (0.69, 1.07) | 83.85 | 80 | 0.58 |
| Present-centered therapy vs. Waitlist | 0.87 | (0.70, 1.09) | 81.28 | 78 | 0.30 |
| IPT vs. Waitlist | 0.86 | (0.70, 1.08) | 83.67 | 80 | 0.93 |
| Combined somatic/cognitive therapies vs. Waitlist | 0.86 | (0.70, 1.08) | 83.60 | 80 | 0.78 |
| Self-help with support vs. Waitlist | 0.86 | (0.70, 1.07) | 83.57 | 80 | 0.47 |
| Self-help without support vs. Waitlist | 0.86 | (0.69, 1.07) | 83.66 | 80 | 0.39 |
| SSRI vs. Waitlist | 0.83 | (0.67, 1.04) | 83.00 | 79 | 0.18 |
| TF-CBT + SSRI vs. Waitlist | 0.83 | (0.67, 1.04) | 83.02 | 79 | 0.07 |
| Counselling vs. Waitlist | 0.85 | (0.69, 1.08) | 82.32 | 78 | 0.41 |
| TF-CBT vs. Waitlist | 0.89 | (0.72, 1.12) | 79.06 | 76 | 0.43 |
| EMDR vs. non-TF-CBT | 0.86 | (0.70, 1.08) | 83.63 | 80 | 0.51 |
| Present-centered therapy vs. non-TF-CBT | 0.87 | (0.70, 1.09) | 82.44 | 79 | 0.65 |
| Attention placebo vs. non-TF-CBT | 0.86 | (0.70, 1.08) | 83.61 | 80 | 0.68 |
| Counselling vs. non-TF-CBT | 0.86 | (0.70, 1.08) | 82.65 | 79 | 0.39 |
| TF-CBT vs. non-TF-CBT | 0.86 | (0.70, 1.08) | 82.65 | 79 | 0.39 |
| Combined somatic/cognitive therapies vs. EMDR | 0.86 | (0.70, 1.08) | 83.62 | 80 | 0.79 |
| SSRI vs. EMDR | 0.86 | (0.70, 1.07) | 83.65 | 80 | 0.48 |
| Relaxation vs. EMDR | 0.85 | (0.69, 1.07) | 83.75 | 80 | 0.31 |
| Counselling vs. EMDR | 0.86 | (0.70, 1.08) | 83.63 | 80 | 0.95 |
| TF-CBT vs. EMDR | 0.85 | (0.68, 1.06) | 83.56 | 80 | 0.15 |
| TF-CBT vs. Present-centered therapy | 0.86 | (0.70, 1.08) | 82.47 | 79 | 0.41 |
| Relaxation vs. IPT | 0.86 | (0.70, 1.08) | 82.72 | 79 | 0.54 |
| TF-CBT vs. IPT | 0.86 | (0.70, 1.08) | 82.68 | 79 | 0.72 |
| Self-help without support vs. Attention placebo | 0.86 | (0.70, 1.08) | 83.58 | 80 | 0.68 |
| Self-help without support vs. Self-help with support | 0.86 | (0.70, 1.07) | 83.63 | 80 | 0.47 |
| TF-CBT vs. SSRI | 0.86 | (0.70, 1.08) | 81.60 | 78 | 0.44 |
| TF-CBT vs. TF-CBT + SSRI | 0.84 | (0.67, 1.05) | 81.94 | 78 | 0.91 |
| TF-CBT vs. Relaxation | 0.86 | (0.70, 1.08) | 82.67 | 79 | 0.29 |
| TF-CBT vs. Counselling | 0.86 | (0.69, 1.08) | 81.03 | 77 | 0.74 |
| **NMA (no nodes split)** | **0.85** | **(0.69, 1.07)** | **83.75** | **80** | **---** |
| a The number of data points varies due to the inclusion of multi-arm trials (van Valkenhoef *et al.* 2016). Continuous trial data were inputted as standardised mean differences, accompanied with the standard error of the mean of the baseline arm on the standardised scale in order to compute the covariance of the differences in multi-arm trials  b p-values < 0.05 are indicative of evidence of inconsistency between the direct and indirect estimates  CBT: cognitive behavioural therapy; EMDR: eye movement desensitisation and reprocessing; indiv: individual; NMA: network meta-analysis; SD: standard deviation; SSRI: selective serotonine re-uptake inhibitor; TF: trauma-focused | | | | | |

### Direct, indirect, and network estimates of relative treatment effects based on node-splitting results


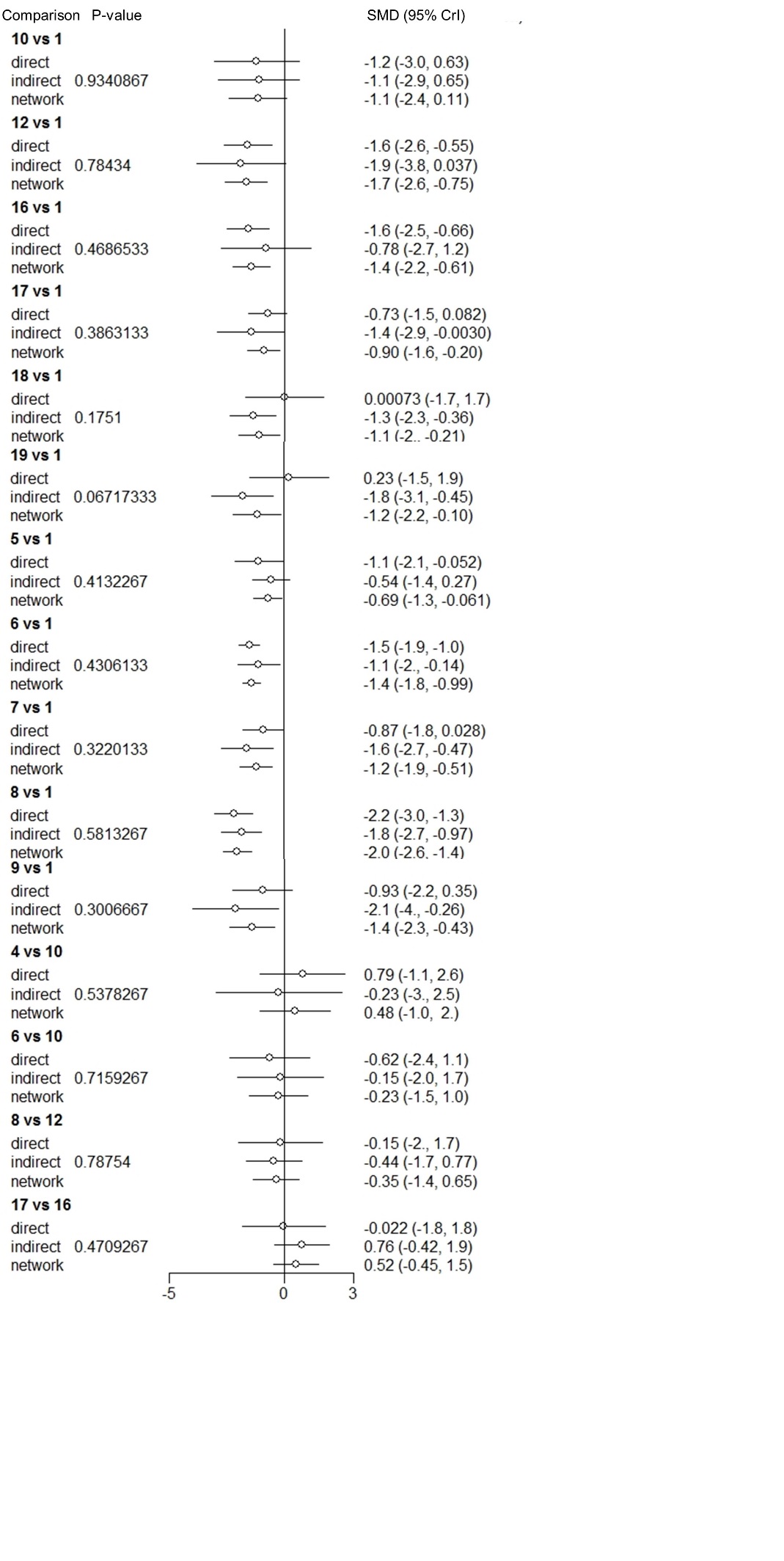


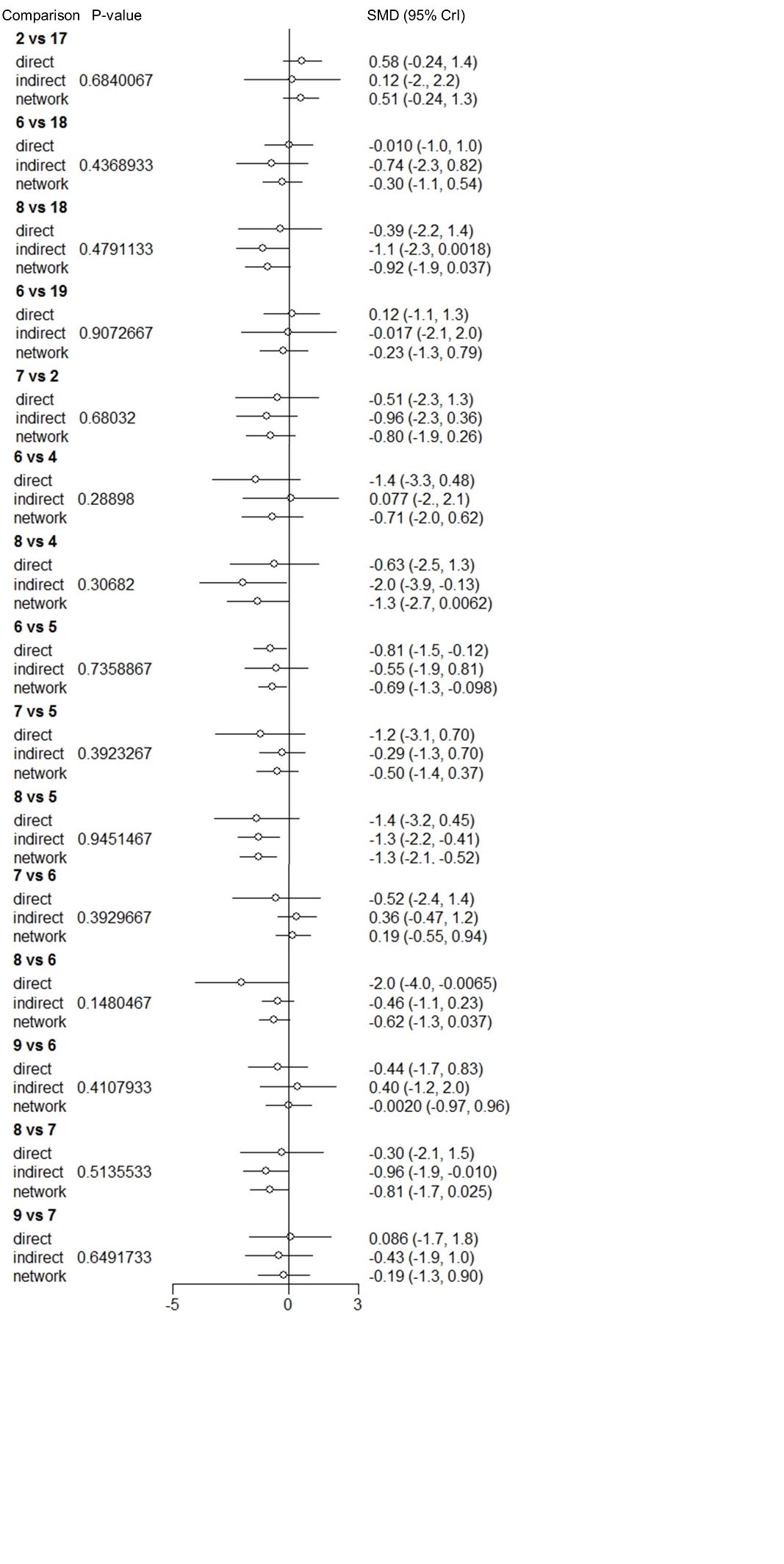


Treatment codes: 1. Waitlist; 2. Attention placebo; 3. Psychoeducation; 4. Relaxation; 5. Counselling; 6. TF-CBT; 7. non-TF-CBT; 8. EMDR; 9. Present-centered therapy; 10. IPT; 11. Metacognitive therapy; 12. Combined somatic/cognitive therapies; 13. Resilience-oriented treatment; 14. Attention bias modification; 15. Couple intervention; 16. Self-help with support; 17. Self-help without support; 18. SSRI; 19. TF-CBT + SSRI

## B. Changes in PTSD symptom scores between baseline and 1-4 month follow-up

No evidence of inconsistency was found through comparison of the consistency and inconsistency random effects models, as little difference was observed between the models in terms of the posterior median between-study standard deviation, posterior mean residual deviance and DIC (Appendix 9). In addition, there were no meaningful improvements in the prediction of data points by the inconsistency model. No evidence of inconsistency was found through further checks for inconsistency using the node-splitting method (random effects model) (see Deviance plot in Figure below).

### Deviance contributions for the random effects consistency and inconsistency models


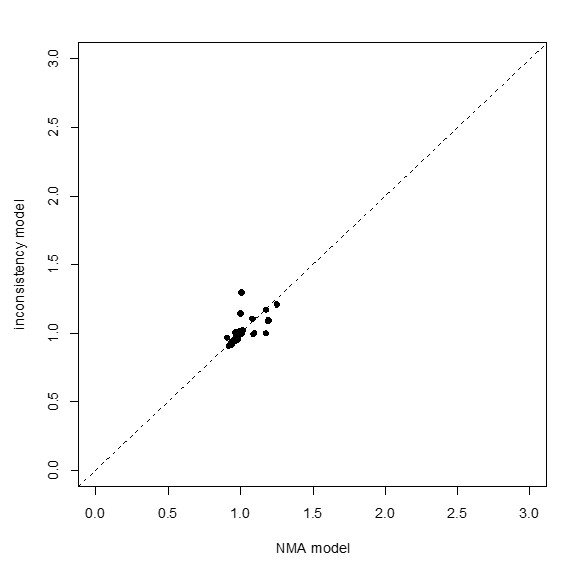


### Summary of node-splitting results

| Node split model | Heterogeneity (SD) | | Residual deviance | Data points^a^ | p-value^b^ |
| --- | --- | --- | --- | --- | --- |
|  | Median | 95% CrI |  |  |  |
| Self-help with support vs. Waitlist | 0.6 | (0.37, 1.02) | 28.51 | 28 | 0.58 |
| Psychoeducation vs. Waitlist | 0.57 | (0.35, 0.98) | 29.69 | 29 | 0.54 |
| TF-CBT vs. Waitlist | 0.53 | (0.32, 0.90) | 28.19 | 28 | 0.22 |
| EMDR vs. Waitlist | 0.49 | (0.28, 0.85) | 30.1 | 29 | 0.07 |
| TF-CBT vs. Present-centered therapy | 0.56 | (0.34, 0.96) | 30.04 | 29 | 0.49 |
| non-TF-CBT vs. Present-centered therapy | 0.56 | (0.34, 0.96) | 30.06 | 29 | 0.49 |
| Self-help without support vs. Self-help with support | 0.58 | (0.36, 0.98) | 29.73 | 29 | 0.66 |
| TF-CBT vs. Self-help with support | 0.56 | (0.33, 0.97) | 28.62 | 28 | 0.16 |
| Attention placebo vs. Self-help without support | 0.58 | (0.36, 0.97) | 29.68 | 29 | 0.67 |
| non-TF-CBT vs. Attention placebo | 0.58 | (0.36, 0.97) | 29.68 | 29 | 0.67 |
| TF-CBT vs. Psychoeducation | 0.57 | (0.35, 0.97) | 29.7 | 29 | 0.54 |
| non-TF-CBT vs. TF-CBT | 0.53 | (0.32, 0.90) | 29.72 | 29 | 0.17 |
| EMDR vs. non-TF-CBT | 0.49 | (0.28, 0.85) | 30.09 | 29 | 0.07 |
| NMA (no nodes split) | **0.56** | **(0.34, 0.92)** | **29.83** | **29** | **---** |
| a The number of data points varies due to the inclusion of multi-arm trials (van Valkenhoef *et al.* 2016). Continuous trial data were inputted as standardised mean differences, accompanied with the standard error of the mean of the baseline arm on the standardised scale in order to compute the covariance of the differences in multi-arm trials  b p-values < 0.05 are indicative of evidence of inconsistency between the direct and indirect estimates  CBT: cognitive behavioural therapy; EMDR: eye movement desensitisation and reprocessing; indiv: individual; NMA: network meta-analysis; SD: standard deviation; TF: trauma-focused | | | | | |

### Direct, indirect, and network estimates of relative treatment effects based on node-splitting results


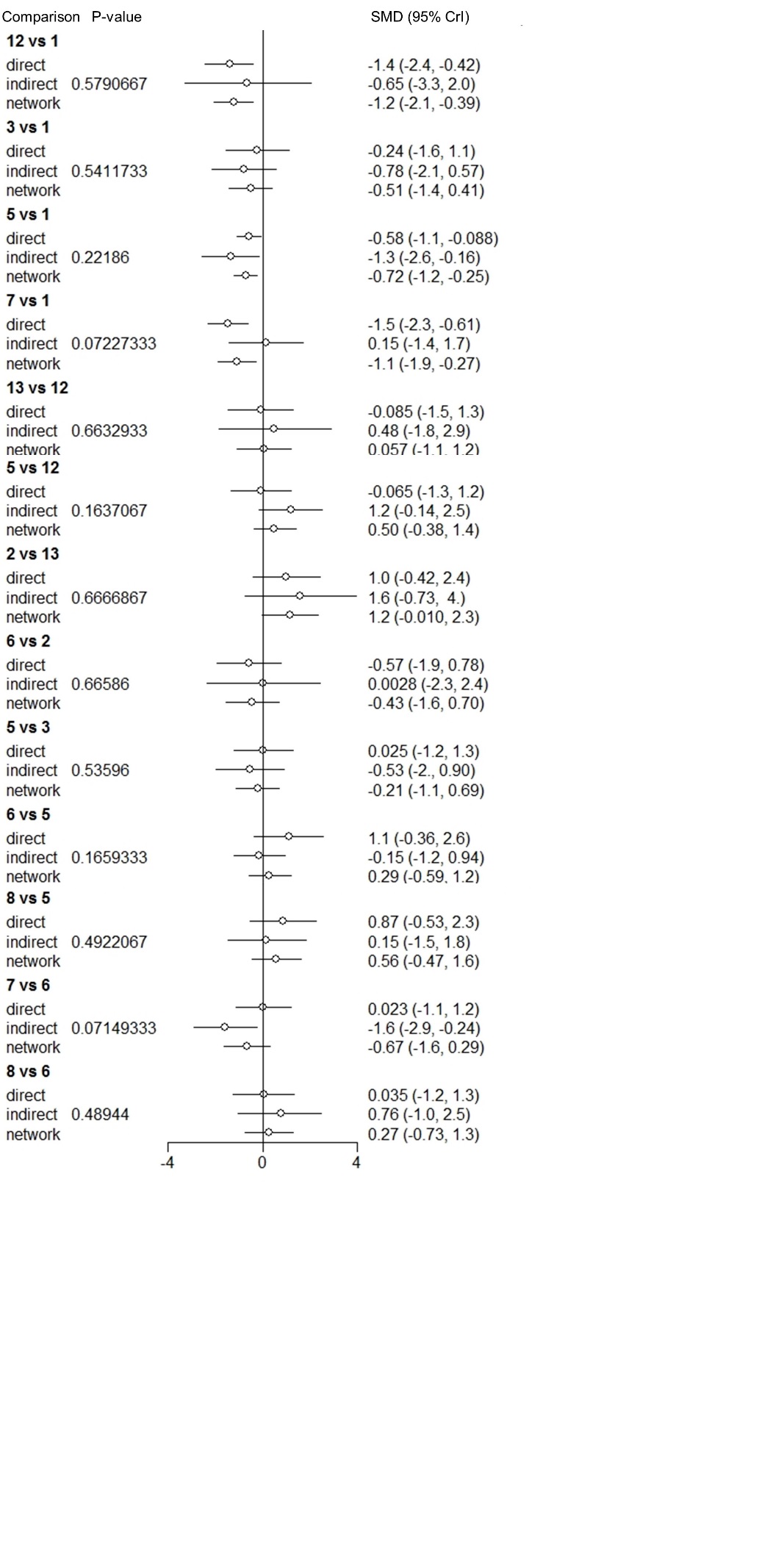


Treatment codes: 1. Waitlist; 2. Attention placebo; 3. Psychoeducation; 4. Counselling; 5. TF-CBT; 6. non-TF-CBT; 7. EMDR; 8. Present-centered therapy; 9. Combined somatic/cognitive therapies; 10. IPT; 11. Couple intervention; 12. Self-help with support; 13. Self-help without support; 14. Family therapy; 15. Behavioural therapy

## C. Dichotomous remission at treatment endpoint

No evidence of inconsistency was found through comparison of the consistency and inconsistency random effects models, as little difference was observed between the models in terms of the posterior median between-study standard deviation, posterior mean residual deviance and DIC (Appendix 9). Nevertheless, the inconsistency model notably better predicted data points in Capezzani 2013 (comparing TF-CBT and EMDR), indicating evidence of potential inconsistency. Further checks for inconsistency using the node-splitting method (random effects model) revealed evidence of inconsistency between the direct and indirect estimates contributing to the pooled estimate of TF-CBT versus EMDR. In addition, there was evidence of inconsistency between the direct and indirect estimates of TF-CBT versus self-help without support, which were directly compared in Ehlers 2003. The inconsistency model minimally improved the prediction of one data point in this study, compared to the consistency model (see Deviance plot in Figure below).

### Deviance contributions for the random effects consistency and inconsistency models


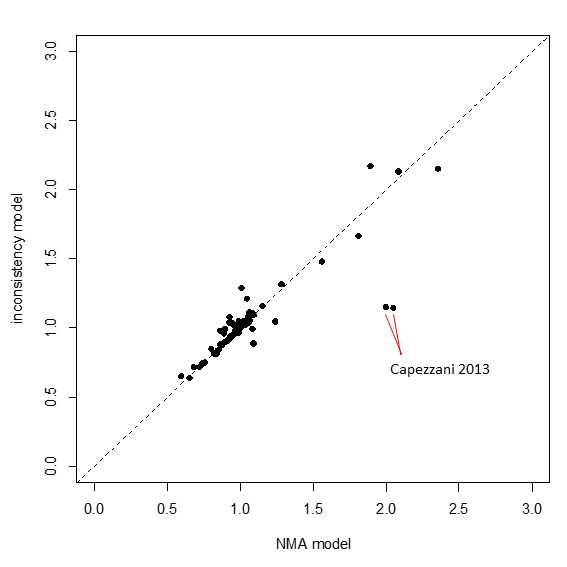


### Summary of node-splitting results

| Node split model | Heterogeneity (SD) | | Residual deviance | Data points^a^ | p-value^b^ |
| --- | --- | --- | --- | --- | --- |
|  | Median | 95% CrI |  |  |  |
| non-TF-CBT vs. Waitlist | 0.64 | (0.07, 1.26) | 48.58 | 41 | 0.87 |
| EMDR vs. Waitlist | 1.10 | (0.64, 1.76) | 78.58 | 76 | 0.50 |
| IPT vs. Waitlist | 1.09 | (0.62, 1.77) | 79.08 | 76 | 0.72 |
| Counselling vs. Waitlist | 1.18 | (0.69, 1.93) | 76.75 | 74 | 0.86 |
| TF-CBT vs. Waitlist | 0.68 | (0.12, 1.38) | 77.09 | 72 | 0.83 |
| Present-centred therapy vs. non-TF-CBT | 1.08 | (0.62, 1.75) | 78.01 | 75 | 0.61 |
| Counselling vs. non-TF-CBT | 1.12 | (0.64, 1.82) | 78.35 | 75 | 0.74 |
| TF-CBT vs. non-TF-CBT | 1.12 | (0.65, 1.84) | 78.25 | 75 | 0.91 |
| SSRI vs. EMDR | 1.03 | (0.57, 1.70) | 79.32 | 76 | 0.26 |
| Relaxation vs. EMDR | 1.08 | (0.62, 1.76) | 79.02 | 76 | 0.50 |
| TF-CBT vs. EMDR | 0.87 | (0.44, 1.45) | 78.96 | 76 | 0.01 |
| Relaxation vs. IPT | 1.12 | (0.65, 1.84) | 77.9 | 75 | 0.52 |
| TF-CBT vs. IPT | 1.13 | (0.65, 1.83) | 77.87 | 75 | 0.84 |
| TF-CBT vs. Present-centred therapy | 1.04 | (0.58, 1.73) | 77.63 | 75 | 0.65 |
| TF-CBT vs. Self-help without support | 0.47 | (0.04, 1.08) | 82.32 | 75 | 0.00 |
| TF-CBT vs. SSRI | 1.04 | (0.57, 1.71) | 78.26 | 75 | 0.26 |
| TF-CBT vs. Relaxation | 1.13 | (0.65, 1.85) | 77.82 | 75 | 0.56 |
| NMA (no nodes split) | **1.05** | **(0.60, 1.69)** | **79.22** | **76** | **---** |
| a The number of data points varies due to the inclusion of multi-arm trials (van Valkenhoef *et al.* 2016). Data for the non-TF-CBT vs. Waitlist node split model were inputted as log odds ratios, accompanied with the standard error of the log odds of the baseline arm in order to compute the covariance of the differences in multi-arm trials; a continuity correction was applied. Data for all other node split models were inputted at arm-level (i.e., numerators and denominators).  b p-values < 0.05 are indicative of evidence of inconsistency between the direct and indirect estimates  CBT: cognitive behavioural therapy; EMDR: eye movement desensitisation and reprocessing; indiv: individual; NMA: network meta-analysis; SD: standard deviation; SSRI: selective serotonine uptake inhibitor; TF: trauma-focused | | | | | |

### Direct, indirect, and network estimates of relative treatment effects based on node-splitting results


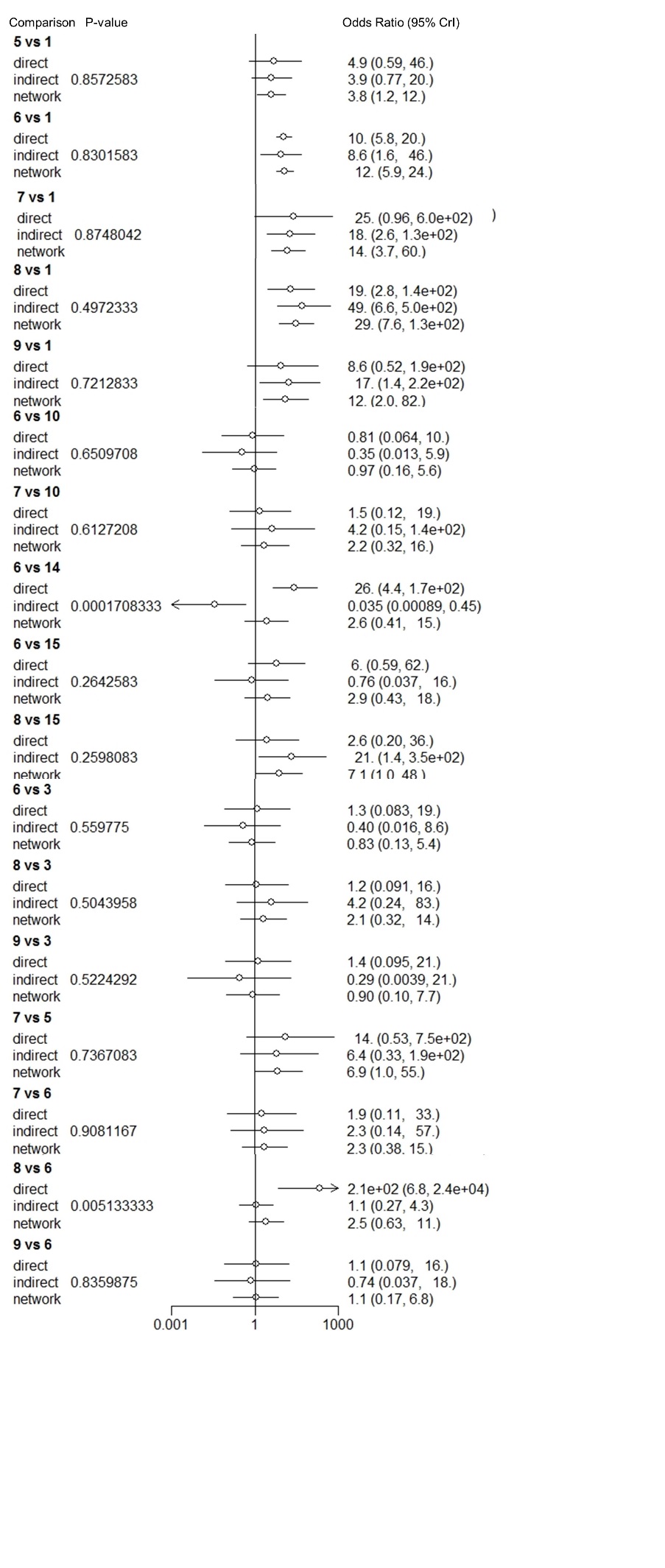


Treatment codes: 1. Waitlist; 2. Attention placebo; 3. Relaxation; 4. Psychoeducation; 5. Counselling; 6. TF-CBT; 7. non-TF-CBT; 8. EMDR; 9. IPT; 10. Present-centred therapy; 11. Psychodynamic therapy; 12. Couple intervention; 13. Self-help with support; 14. Self-help without support; 15. SSRI; 16. TF-CBT + SSRI. Continuity correction was applied in node split model for 7 vs. 1 comparison.
