# Supplementary material for: Cost-effectiveness of psychological treatments for post-traumatic stress disorder in adults
Source: PLoS One. 2020 Apr 30;15(4):e0232245. doi: 10.1371/journal.pone.0232245 (PMC7192458; doi:10.1371/journal.pone.0232245)
Supplement: S11 Appendix — (DOCX) [file pone.0232245.s018.docx]

# **Appendix 11: Relative effects between all pairs of interventions: direct, indirect and combined (NMA) results**

## A. Standardised mean differences (changes in PTSD symptom scores) between baseline and treatment endpoint

All NMA estimates are reported based on the results from the random effects model that assumes consistency (Dias *et al.* 2013a); the direct and indirect estimates are reported based on results given by the node-split models (Dias *et al.* 2013b). Direct and indirect estimates are presented when available.

Negative values favour first intervention in the comparison.

| **Comparison** | **Effect: standardised mean difference (SMD)** | | |
| --- | --- | --- | --- |
|  | **NMA**  **median SMD (95% CrI)** | **Direct**  **median SMD (95% CI)** | **Indirect**  **median SMD (95% CI)** |
| Attention placebo vs. Waitlist | -0.39 (-1.42, 0.63) |  | -0.39 (-1.42, 0.63) |
| Psychoeducation vs. Waitlist | -1.21 (-3.13, 0.71) |  | -1.21 (-3.13, 0.71) |
| Relaxation vs. Waitlist | -0.73 (-2.15, 0.70) |  | -0.73 (-2.15, 0.70) |
| Counselling vs. Waitlist | -0.72 (-1.41, -0.05) | -1.08 (-2.11, -0.05) | -0.54 (-1.36, 0.27) |
| TF-CBT vs. Waitlist | -1.46 (-1.87, -1.05) | -1.48 (-1.94, -1.03) | -1.07 (-2.00, -0.14) |
| non-TF-CBT vs. Waitlist | -1.22 (-1.95, -0.49) | -0.87 (-1.77, 0.03) | -1.58 (-2.69, -0.47) |
| EMDR vs. Waitlist | -2.07 (-2.70, -1.44) | -2.16 (-3.00, -1.33) | -1.83 (-2.71, -0.97) |
| Present-centered therapy vs. Waitlist | -1.42 (-2.45, -0.40) | -0.93 (-2.22, 0.35) | -2.11 (-3.97, -0.26) |
| IPT vs. Waitlist | -1.19 (-2.54, 0.15) | -1.20 (-3.04, 0.63) | -1.10 (-2.86, 0.65) |
| Metacognitive therapy vs. Waitlist | -3.04 (-5.09, -0.98) | -3.04 (-5.09, -0.98) |  |
| Combined somatic/cognitive therapies vs. Waitlist | -1.69 (-2.66, -0.73) | -1.59 (-2.62, -0.55) | -1.89 (-3.81, 0.04) |
| Resilience-oriented treatment vs. Waitlist | -1.63 (-3.59, 0.32) | 1.63 (-3.59, 0.32) | - |
| Attention bias modification vs. Waitlist | 2.13 (0.63, 3.65) |  | 2.13 (0.63, 3.65) |
| Couple intervention vs. Waitlist | -2.67 (-5.41, 0.06) |  | -2.67 (-5.41, 0.06) |
| Self-help with support vs. Waitlist | -1.46 (-2.33, -0.59) | -1.56 (-2.47, -0.66) | -0.78 (-2.73, 1.17) |
| Self-help without support vs. Waitlist | -0.91 (-1.67, -0.15) | -0.73 (-1.54, 0.08) | -1.45 (-2.89, 0.00) |
| SSRI vs. Waitlist | -1.14 (-2.09, -0.19) | 0.00 (-1.69, 1.70) | -1.34 (-2.34, -0.36) |
| TF-CBT + SSRI vs. Waitlist | -1.21 (-2.35, -0.07) | 0.23 (-1.46, 1.94) | -1.78 (-3.11, -0.45) |
|  | | | |
| Psychoeducation vs. Attention placebo | -0.81 (-2.99, 1.34) |  | -0.81 (-2.99, 1.34) |
| Relaxation vs. Attention placebo | -0.33 (-2.09, 1.41) |  | -0.33 (-2.09, 1.41) |
| Counselling vs. Attention placebo | -0.33 (-1.55, 0.88) |  | -0.33 (-1.55, 0.88) |
| TF-CBT vs. Attention placebo | -1.07 (-2.16, 0.02) |  | -1.07 (-2.16, 0.02) |
| non-TF-CBT vs. Attention placebo | -0.83 (-1.97, 0.32) | -0.51 (-2.30, 1.29) | -0.96 (-2.29, 0.36) |
| EMDR vs. Attention placebo | -1.67 (-2.87, -0.48) |  | -1.67 (-2.87, -0.48) |
| Present-centered therapy vs. Attention placebo | -1.03 (-2.46, 0.40) |  | -1.03 (-2.46, 0.40) |
| IPT vs. Attention placebo | -0.79 (-2.48, 0.88) |  | -0.79 (-2.48, 0.88) |
| Metacognitive therapy vs. Attention placebo | -2.64 (-4.95, -0.35) |  | -2.64 (-4.95, -0.35) |
| Combined somatic/cognitive therapies vs. Attention placebo | -1.30 (-2.71, 0.11) |  | -1.30 (-2.71, 0.11) |
| Resilience-oriented treatment vs. Attention placebo | -1.23 (-3.47, 0.97) |  | -1.23 (-3.47, 0.97) |
| Attention bias modification vs. Attention placebo | 2.53 (1.42, 3.65) | 2.53 (1.42, 3.65) |  |
| Couple intervention vs. Attention placebo | -2.28 (-5.22, 0.64) |  | -2.28 (-5.22, 0.64) |
| Self-help with support vs. Attention placebo | -1.06 (-2.34, 0.20) |  | -1.06 (-2.34, 0.20) |
| Self-help without support vs. Attention placebo | -0.52 (-1.32, 0.29) | -0.58 (-1.39, 0.24) | -0.12 (-2.21, 1.96) |
| SSRI vs. Attention placebo | -0.75 (-2.14, 0.64) |  | -0.75 (-2.14, 0.64) |
| TF-CBT + SSRI vs. Attention placebo | -0.82 (-2.35, 0.71) |  | -0.82 (-2.35, 0.71) |
|  | | | |
| Relaxation vs. Psychoeducation | 0.48 (-1.88, 2.84) |  | 0.48 (-1.88, 2.84) |
| Counselling vs. Psychoeducation | 0.48 (-1.50, 2.46) |  | 0.48 (-1.50, 2.46) |
| TF-CBT vs. Psychoeducation | -0.25 (-2.12, 1.62) | -0.25 (-2.12, 1.62) |  |
| non-TF-CBT vs. Psychoeducation | -0.01 (-2.05, 2.03) |  | -0.01 (-2.05, 2.03) |
| EMDR vs. Psychoeducation | -0.86 (-2.86, 1.14) |  | -0.86 (-2.86, 1.14) |
| Present-centered therapy vs. Psychoeducation | -0.21 (-2.34, 1.93) |  | -0.21 (-2.34, 1.93) |
| IPT vs. Psychoeducation | 0.02 (-2.29, 2.33) |  | 0.02 (-2.29, 2.33) |
| Metacognitive therapy vs. Psychoeducation | -1.83 (-4.66, 0.98) |  | -1.83 (-4.66, 0.98) |
| Combined somatic/cognitive therapies vs. Psychoeducation | -0.49 (-2.61, 1.65) |  | -0.49 (-2.61, 1.65) |
| Resilience-oriented treatment vs. Psychoeducation | -0.43 (-3.16, 2.31) |  | -0.43 (-3.16, 2.31) |
| Attention bias modification vs. Psychoeducation | 3.34 (0.91, 5.80) |  | 3.34 (0.91, 5.80) |
| Couple intervention vs. Psychoeducation | -1.47 (-3.40, 0.49) | -1.47 (-3.40, 0.49) |  |
| Self-help with support vs. Psychoeducation | -0.25 (-2.35, 1.86) |  | -0.25 (-2.35, 1.86) |
| Self-help without support vs. Psychoeducation | 0.30 (-1.76, 2.36) |  | 0.30 (-1.76, 2.36) |
| SSRI vs. Psychoeducation | 0.06 (-2.04, 2.16) |  | 0.06 (-2.04, 2.16) |
| TF-CBT + SSRI vs. Psychoeducation | 0.00 (-2.20, 2.18) |  | 0.00 (-2.20, 2.18) |
|  | | | |
| Counselling vs. Relaxation | 0.00 (-1.54, 1.52) |  | 0.00 (-1.54, 1.52) |
| TF-CBT vs. Relaxation | -0.73 (-2.16, 0.69) | -1.41 (-3.30, 0.48) | 0.08 (-1.97, 2.12) |
| non-TF-CBT vs. Relaxation | -0.49 (-2.06, 1.09) |  | -0.49 (-2.06, 1.09) |
| EMDR vs. Relaxation | -1.34 (-2.76, 0.09) | -0.63 (-2.54, 1.29) | -2.00 (-3.86, -0.13) |
| Present-centered therapy vs. Relaxation | -0.69 (-2.43, 1.03) |  | -0.69 (-2.43, 1.03) |
| IPT vs. Relaxation | -0.46 (-2.10, 1.17) | -0.79 (-2.64, 1.07) | 0.23 (-2.50, 2.96) |
| Metacognitive therapy vs. Relaxation | -2.31 (-4.81, 0.20) |  | -2.31 (-4.81, 0.20) |
| Combined somatic/cognitive therapies vs. Relaxation | -0.97 (-2.67, 0.72) |  | -0.97 (-2.67, 0.72) |
| Resilience-oriented treatment vs. Relaxation | -0.90 (-3.34, 1.51) |  | -0.90 (-3.34, 1.51) |
| Attention bias modification vs. Relaxation | 2.86 (0.80, 4.94) |  | 2.86 (0.80, 4.94) |
| Couple intervention vs. Relaxation | -1.95 (-5.01, 1.13) |  | -1.95 (-5.01, 1.13) |
| Self-help with support vs. Relaxation | -0.73 (-2.40, 0.93) |  | -0.73 (-2.40, 0.93) |
| Self-help without support vs. Relaxation | -0.18 (-1.80, 1.42) |  | -0.18 (-1.80, 1.42) |
| SSRI vs. Relaxation | -0.42 (-2.07, 1.24) |  | -0.42 (-2.07, 1.24) |
| TF-CBT + SSRI vs. Relaxation | -0.48 (-2.26, 1.31) |  | -0.48 (-2.26, 1.31) |
|  | | | |
| TF-CBT vs. Counselling | -0.73 (-1.37, -0.09) | -0.81 (-1.50, -0.12) | -0.55 (-1.91, 0.81) |
| non-TF-CBT vs. Counselling | -0.49 (-1.43, 0.44) | -1.22 (-3.15, 0.70) | -0.29 (-1.28, 0.70) |
| EMDR vs. Counselling | -1.34 (-2.19, -0.49) | -1.38 (-3.20, 0.45) | -1.30 (-2.20, -0.41) |
| Present-centered therapy vs. Counselling | -0.69 (-1.88, 0.48) |  | -0.69 (-1.88, 0.48) |
| IPT vs. Counselling | -0.46 (-1.93, 1.01) |  | -0.46 (-1.93, 1.01) |
| Metacognitive therapy vs. Counselling | -2.31 (-4.46, -0.15) |  | -2.31 (-4.46, -0.15) |
| Combined somatic/cognitive therapies vs. Counselling | -0.97 (-2.13, 0.19) |  | -0.97 (-2.13, 0.19) |
| Resilience-oriented treatment vs. Counselling | -0.90 (-2.98, 1.16) |  | -0.90 (-2.98, 1.16) |
| Attention bias modification vs. Counselling | 2.86 (1.23, 4.51) |  | 2.86 (1.23, 4.51) |
| Couple intervention vs. Counselling | -1.95 (-4.71, 0.83) |  | -1.95 (-4.71, 0.83) |
| Self-help with support vs. Counselling | -0.74 (-1.84, 0.38) |  | -0.74 (-1.84, 0.38) |
| Self-help without support vs. Counselling | -0.18 (-1.19, 0.82) |  | -0.18 (-1.19, 0.82) |
| SSRI vs. Counselling | -0.42 (-1.50, 0.67) |  | -0.42 (-1.50, 0.67) |
| TF-CBT + SSRI vs. Counselling | -0.48 (-1.74, 0.78) |  | -0.48 (-1.74, 0.78) |
|  | | | |
| non-TF-CBT vs. TF-CBT | 0.24 (-0.56, 1.04) | -0.52 (-2.39, 1.35) | 0.36 (-0.47, 1.20) |
| EMDR vs. TF-CBT | -0.61 (-1.30, 0.08) | -2.01 (-4.01, -0.01) | -0.46 (-1.14, 0.23) |
| Present-centered therapy vs. TF-CBT | 0.04 (-0.99, 1.07) | -0.44 (-1.71, 0.83) | 0.40 (-1.21, 2.02) |
| IPT vs. TF-CBT | 0.27 (-1.09, 1.63) | 0.62 (-1.12, 2.37) | 0.15 (-1.72, 2.03) |
| Metacognitive therapy vs. TF-CBT | -1.58 (-3.67, 0.51) |  | -1.58 (-3.67, 0.51) |
| Combined somatic/cognitive therapies vs. TF-CBT | -0.23 (-1.28, 0.80) |  | -0.23 (-1.28, 0.80) |
| Resilience-oriented treatment vs. TF-CBT | -0.17 (-2.18, 1.83) |  | -0.17 (-2.18, 1.83) |
| Attention bias modification vs. TF-CBT | 3.59 (2.03, 5.16) |  | 3.59 (2.03, 5.16) |
| Couple intervention vs. TF-CBT | -1.21 (-3.91, 1.49) |  | -1.21 (-3.91, 1.49) |
| Self-help with support vs. TF-CBT | 0.00 (-0.96, 0.97) |  | 0.00 (-0.96, 0.97) |
| Self-help without support vs. TF-CBT | 0.55 (-0.31, 1.41) |  | 0.55 (-0.31, 1.41) |
| SSRI vs. TF-CBT | 0.31 (-0.60, 1.23) | 0.01 (-1.02, 1.04) | 0.74 (-0.82, 2.29) |
| TF-CBT + SSRI vs. TF-CBT | 0.25 (-0.86, 1.35) | -0.12 (-1.34, 1.09) | 0.02 (-2.03, 2.06) |
|  | | | |
| EMDR vs. non-TF-CBT | -0.85 (-1.75, 0.05) | -0.30 (-2.08, 1.49) | -0.96 (-1.92, -0.01) |
| Present-centered therapy vs. non-TF-CBT | -0.20 (-1.37, 0.97) | 0.09 (-1.67, 1.85) | -0.43 (-1.87, 1.01) |
| IPT vs. non-TF-CBT | 0.04 (-1.50, 1.55) |  | 0.04 (-1.50, 1.55) |
| Metacognitive therapy vs. non-TF-CBT | -1.82 (-4.00, 0.35) |  | -1.82 (-4.00, 0.35) |
| Combined somatic/cognitive therapies vs. non-TF-CBT | -0.47 (-1.67, 0.72) |  | -0.47 (-1.67, 0.72) |
| Resilience-oriented treatment vs. non-TF-CBT | -0.41 (-2.51, 1.68) |  | -0.41 (-2.51, 1.68) |
| Attention bias modification vs. non-TF-CBT | 3.36 (1.76, 4.95) |  | 3.36 (1.76, 4.95) |
| Couple intervention vs. non-TF-CBT | -1.46 (-4.27, 1.36) |  | -1.46 (-4.27, 1.36) |
| Self-help with support vs. non-TF-CBT | -0.24 (-1.37, 0.88) |  | -0.24 (-1.37, 0.88) |
| Self-help without support vs. non-TF-CBT | 0.31 (-0.68, 1.30) |  | 0.31 (-0.68, 1.30) |
| SSRI vs. non-TF-CBT | 0.08 (-1.09, 1.24) |  | 0.08 (-1.09, 1.24) |
| TF-CBT + SSRI vs. non-TF-CBT | 0.01 (-1.32, 1.35) |  | 0.01 (-1.32, 1.35) |
|  | | | |
| Present-centered therapy vs. EMDR | 0.65 (-0.54, 1.82) |  | 0.65 (-0.54, 1.82) |
| IPT vs. EMDR | 0.88 (-0.56, 2.31) |  | 0.88 (-0.56, 2.31) |
| Metacognitive therapy vs. EMDR | -0.97 (-3.12, 1.18) |  | -0.97 (-3.12, 1.18) |
| Combined somatic/cognitive therapies vs. EMDR | 0.37 (-0.69, 1.44) | 0.15 (-1.68, 1.96) | 0.44 (-0.77, 1.66) |
| Resilience-oriented treatment vs. EMDR | 0.44 (-1.63, 2.49) |  | 0.44 (-1.63, 2.49) |
| Attention bias modification vs. EMDR | 4.20 (2.58, 5.83) |  | 4.20 (2.58, 5.83) |
| Couple intervention vs. EMDR | -0.61 (-3.39, 2.20) |  | -0.61 (-3.39, 2.20) |
| Self-help with support vs. EMDR | 0.61 (-0.47, 1.69) |  | 0.61 (-0.47, 1.69) |
| Self-help without support vs. EMDR | 1.16 (0.18, 2.14) |  | 1.16 (0.18, 2.14) |
| SSRI vs. EMDR | 0.92 (-0.10, 1.95) | 0.39 (-1.40, 2.18) | 1.14 (0.00, 2.29) |
| TF-CBT + SSRI vs. EMDR | 0.86 (-0.38, 2.10) |  | 0.86 (-0.38, 2.10) |
|  | | | |
| IPT vs. Present-centered therapy | 0.23 (-1.44, 1.90) |  | 0.23 (-1.44, 1.90) |
| Metacognitive therapy vs. Present-centered therapy | -1.62 (-3.93, 0.67) |  | -1.62 (-3.93, 0.67) |
| Combined somatic/cognitive therapies vs. Present-centered therapy | -0.28 (-1.67, 1.13) |  | -0.28 (-1.67, 1.13) |
| Resilience-oriented treatment vs. Present-centered therapy | -0.21 (-2.44, 2.01) |  | -0.21 (-2.44, 2.01) |
| Attention bias modification vs. Present-centered therapy | 3.56 (1.75, 5.37) |  | 3.56 (1.75, 5.37) |
| Couple intervention vs. Present-centered therapy | -1.26 (-4.14, 1.64) |  | -1.26 (-4.14, 1.64) |
| Self-help with support vs. Present-centered therapy | -0.04 (-1.38, 1.31) |  | -0.04 (-1.38, 1.31) |
| Self-help without support vs. Present-centered therapy | 0.51 (-0.76, 1.77) |  | 0.51 (-0.76, 1.77) |
| SSRI vs. Present-centered therapy | 0.28 (-1.08, 1.63) |  | 0.28 (-1.08, 1.63) |
| TF-CBT + SSRI vs. Present-centered therapy | 0.21 (-1.29, 1.70) |  | 0.21 (-1.29, 1.70) |
|  | | | |
| Metacognitive therapy vs. IPT | -1.85 (-4.30, 0.61) |  | -1.85 (-4.30, 0.61) |
| Combined somatic/cognitive therapies vs. IPT | -0.51 (-2.14, 1.14) |  | -0.51 (-2.14, 1.14) |
| Resilience-oriented treatment vs. IPT | -0.45 (-2.83, 1.94) |  | -0.45 (-2.83, 1.94) |
| Attention bias modification vs. IPT | 3.33 (1.32, 5.34) |  | 3.33 (1.32, 5.34) |
| Couple intervention vs. IPT | -1.49 (-4.52, 1.55) |  | -1.49 (-4.52, 1.55) |
| Self-help with support vs. IPT | -0.27 (-1.87, 1.33) |  | -0.27 (-1.87, 1.33) |
| Self-help without support vs. IPT | 0.28 (-1.26, 1.82) |  | 0.28 (-1.26, 1.82) |
| SSRI vs. IPT | 0.04 (-1.56, 1.66) |  | 0.04 (-1.56, 1.66) |
| TF-CBT + SSRI vs. IPT | -0.02 (-1.75, 1.71) |  | -0.02 (-1.75, 1.71) |
|  | | | |
| Combined somatic/cognitive therapies vs. Metacognitive therapy | 1.34 (-0.92, 3.62) |  | 1.34 (-0.92, 3.62) |
| Resilience-oriented treatment vs. Metacognitive therapy | 1.41 (-1.43, 4.25) |  | 1.41 (-1.43, 4.25) |
| Attention bias modification vs. Metacognitive therapy | 5.18 (2.63, 7.71) |  | 5.18 (2.63, 7.71) |
| Couple intervention vs. Metacognitive therapy | 0.37 (-3.06, 3.79) |  | 0.37 (-3.06, 3.79) |
| Self-help with support vs. Metacognitive therapy | 1.58 (-0.65, 3.81) |  | 1.58 (-0.65, 3.81) |
| Self-help without support vs. Metacognitive therapy | 2.13 (-0.07, 4.32) |  | 2.13 (-0.07, 4.32) |
| SSRI vs. Metacognitive therapy | 1.89 (-0.36, 4.15) |  | 1.89 (-0.36, 4.15) |
| TF-CBT + SSRI vs. Metacognitive therapy | 1.82 (-0.52, 4.19) |  | 1.82 (-0.52, 4.19) |
|  | | | |
| Resilience-oriented treatment vs. Combined somatic/cognitive therapies | 0.06 (-2.13, 2.25) |  | 0.06 (-2.13, 2.25) |
| Attention bias modification vs. Combined somatic/cognitive therapies | 3.83 (2.05, 5.63) |  | 3.83 (2.05, 5.63) |
| Couple intervention vs. Combined somatic/cognitive therapies | -0.98 (-3.88, 1.91) |  | -0.98 (-3.88, 1.91) |
| Self-help with support vs. Combined somatic/cognitive therapies | 0.23 (-1.06, 1.54) |  | 0.23 (-1.06, 1.54) |
| Self-help without support vs. Combined somatic/cognitive therapies | 0.79 (-0.46, 2.01) |  | 0.79 (-0.46, 2.01) |
| SSRI vs. Combined somatic/cognitive therapies | 0.55 (-0.78, 1.88) |  | 0.55 (-0.78, 1.88) |
| TF-CBT + SSRI vs. Combined somatic/cognitive therapies | 0.48 (-0.99, 1.96) |  | 0.48 (-0.99, 1.96) |
|  | | | |
| Attention bias modification vs. Resilience-oriented treatment | 3.77 (1.31, 6.24) |  | 3.77 (1.31, 6.24) |
| Couple intervention vs. Resilience-oriented treatment | -1.04 (-4.39, 2.29) |  | -1.04 (-4.39, 2.29) |
| Self-help with support vs. Resilience-oriented treatment | 0.17 (-1.96, 2.32) |  | 0.17 (-1.96, 2.32) |
| Self-help without support vs. Resilience-oriented treatment | 0.72 (-1.37, 2.83) |  | 0.72 (-1.37, 2.83) |
| SSRI vs. Resilience-oriented treatment | 0.49 (-1.68, 2.67) |  | 0.49 (-1.68, 2.67) |
| TF-CBT + SSRI vs. Resilience-oriented treatment | 0.42 (-1.84, 2.70) |  | 0.42 (-1.84, 2.70) |
|  | | | |
| Couple intervention vs. Attention bias modification | -4.81 (-7.95, -1.70) |  | -4.81 (-7.95, -1.70) |
| Self-help with support vs. Attention bias modification | -3.59 (-5.29, -1.91) |  | -3.59 (-5.29, -1.91) |
| Self-help without support vs. Attention bias modification | -3.05 (-4.42, -1.68) |  | -3.05 (-4.42, -1.68) |
| SSRI vs. Attention bias modification | -3.28 (-5.06, -1.50) |  | -3.28 (-5.06, -1.50) |
| TF-CBT + SSRI vs. Attention bias modification | -3.34 (-5.24, -1.46) |  | -3.34 (-5.24, -1.46) |
|  | | | |
| Self-help with support vs. Couple intervention | 1.22 (-1.65, 4.09) |  | 1.22 (-1.65, 4.09) |
| Self-help without support vs. Couple intervention | 1.77 (-1.07, 4.60) |  | 1.77 (-1.07, 4.60) |
| SSRI vs. Couple intervention | 1.52 (-1.32, 4.38) |  | 1.52 (-1.32, 4.38) |
| TF-CBT + SSRI vs. Couple intervention | 1.46 (-1.47, 4.39) |  | 1.46 (-1.47, 4.39) |
|  | | | |
| Self-help without support vs. Self-help with support | 0.55 (-0.51, 1.60) | -0.02 (-1.82, 1.79) | 0.76 (-0.42, 1.95) |
| SSRI vs. Self-help with support | 0.31 (-0.96, 1.60) |  | 0.31 (-0.96, 1.60) |
| TF-CBT + SSRI vs. Self-help with support | 0.25 (-1.20, 1.68) |  | 0.25 (-1.20, 1.68) |
|  | | | |
| SSRI vs. Self-help without support | -0.23 (-1.44, 0.98) |  | -0.23 (-1.44, 0.98) |
| TF-CBT + SSRI vs. Self-help without support | -0.30 (-1.67, 1.06) |  | -0.30 (-1.67, 1.06) |
|  | | | |
| TF-CBT + SSRI vs. SSRI | -0.07 (-1.11, 0.99) | -0.07 (-1.11, 0.99) |  |
| CBT: cognitive behavioural therapy; CI: confidence intervals; CrI: credible intervals; EMDR: eye movement desensitisation and reprocessing; SMD: standardised mean difference; SSRI: selective serotonine reuptake inhibitor; TF: trauma-focused | | | |

## B. Standardised mean differences (changes in PTSD symptom scores) between baseline and 1-4 month follow-up

All NMA estimates are reported based on the results from the random effects model that assumes consistency (Dias *et al.* 2013a); the direct and indirect estimates are reported based on results given by the node-split models (Dias *et al.* 2013b). Direct and indirect estimates are presented when available.

Negative values favour first intervention in the comparison.

| **Comparison** | **Effect: standardised mean difference (SMD)** | | |
| --- | --- | --- | --- |
|  | **NMA**  **median SMD (95% CrI)** | **Direct**  **median SMD (95% CrI)** | **Indirect**  **median SMD (95% CrI)** |
| Attention placebo vs. Waitlist | -0.02 (-1.35, 1.33) |  | -0.02 (-1.35, 1.33) |
| Psychoeducation vs. Waitlist | -0.51 (-1.47, 0.44) | -0.24 (-1.57, 1.10) | -0.78 (-2.15, 0.57) |
| Counselling vs. Waitlist | -0.30 (-1.12, 0.53) |  | -0.30 (-1.12, 0.53) |
| TF-CBT vs. Waitlist | -0.73 (-1.23, -0.25) | -0.58 (-1.08, -0.09) | -1.34 (-2.59, -0.16) |
| non-TF-CBT vs. Waitlist | -0.43 (-1.35, 0.53) |  | -0.43 (-1.35, 0.53) |
| EMDR vs. Waitlist | -1.13 (-1.94, -0.27) | -1.47 (-2.31, -0.61) | 0.15 (-1.42, 1.72) |
| Present-centered therapy vs. Waitlist | -0.16 (-1.29, 1.01) |  | -0.16 (-1.29, 1.01) |
| Combined somatic/cognitive therapies vs. Waitlist | -1.18 (-2.75, 0.43) |  | -1.18 (-2.75, 0.43) |
| IPT vs. Waitlist | -0.39 (-1.76, 0.97) | -0.39 (-1.76, 0.97) |  |
| Couple intervention vs. Waitlist | -2.04 (-3.72, -0.36) |  | -2.04 (-3.72, -0.36) |
| Self-help with support vs. Waitlist | -1.26 (-2.12, -0.42) | -1.40 (-2.43, -0.42) | -0.65 (-3.31, 2.05) |
| Self-help without support vs. Waitlist | -1.19 (-2.52, 0.13) |  | -1.19 (-2.52, 0.13) |
| Family therapy vs. Waitlist | 0.15 (-1.13, 1.43) | 0.15 (-1.13, 1.43) |  |
| Behavioural therapy vs. Waitlist | -1.19 (-2.16, -0.21) | -1.19 (-2.16, -0.21) |  |
|  | | | |
| Psychoeducation vs. Attention placebo | -0.49 (-2.11, 1.10) |  | -0.49 (-2.11, 1.10) |
| Counselling vs. Attention placebo | -0.28 (-1.77, 1.20) |  | -0.28 (-1.77, 1.20) |
| TF-CBT vs. Attention placebo | -0.71 (-2.06, 0.61) |  | -0.71 (-2.06, 0.61) |
| non-TF-CBT vs. Attention placebo | -0.41 (-1.58, 0.76) | -0.57 (-1.91, 0.78) | 0.00 (-2.35, 2.45) |
| EMDR vs. Attention placebo | -1.11 (-2.53, 0.33) |  | -1.11 (-2.53, 0.33) |
| Present-centered therapy vs. Attention placebo | -0.14 (-1.65, 1.39) |  | -0.14 (-1.65, 1.39) |
| Combined somatic/cognitive therapies vs. Attention placebo | -1.16 (-3.11, 0.83) |  | -1.16 (-3.11, 0.83) |
| IPT vs. Attention placebo | -0.37 (-2.31, 1.53) |  | -0.37 (-2.31, 1.53) |
| Couple intervention vs. Attention placebo | -2.02 (-4.16, 0.10) |  | -2.02 (-4.16, 0.10) |
| Self-help with support vs. Attention placebo | -1.25 (-2.62, 0.10) |  | -1.25 (-2.62, 0.10) |
| Self-help without support vs. Attention placebo | -1.17 (-2.37, 0.03) | -1.01 (-2.43, 0.42) | -1.57 (-3.95, 0.73) |
| Family therapy vs. Attention placebo | 0.17 (-1.70, 2.02) |  | 0.17 (-1.70, 2.02) |
| Behavioural therapy vs. Attention placebo | -1.17 (-2.83, 0.47) |  | -1.17 (-2.83, 0.47) |
|  | | | |
| Counselling vs. Psychoeducation | 0.22 (-0.94, 1.38) |  | 0.22 (-0.94, 1.38) |
| TF-CBT vs. Psychoeducation | -0.22 (-1.17, 0.73) | 0.02 (-1.23, 1.29) | -0.53 (-1.96, 0.90) |
| non-TF-CBT vs. Psychoeducation | 0.08 (-1.18, 1.38) |  | 0.08 (-1.18, 1.38) |
| EMDR vs. Psychoeducation | -0.62 (-1.84, 0.65) |  | -0.62 (-1.84, 0.65) |
| Present-centered therapy vs. Psychoeducation | 0.36 (-1.05, 1.79) |  | 0.36 (-1.05, 1.79) |
| Combined somatic/cognitive therapies vs. Psychoeducation | -0.67 (-2.49, 1.20) |  | -0.67 (-2.49, 1.20) |
| IPT vs. Psychoeducation | 0.12 (-1.55, 1.80) |  | 0.12 (-1.55, 1.80) |
| Couple intervention vs. Psychoeducation | -1.53 (-2.91, -0.14) | -1.53 (-2.91, -0.14) |  |
| Self-help with support vs. Psychoeducation | -0.75 (-2.01, 0.49) |  | -0.75 (-2.01, 0.49) |
| Self-help without support vs. Psychoeducation | -0.67 (-2.28, 0.93) |  | -0.67 (-2.28, 0.93) |
| Family therapy vs. Psychoeducation | 0.67 (-0.94, 2.26) |  | 0.67 (-0.94, 2.26) |
| Behavioural therapy vs. Psychoeducation | -0.67 (-2.04, 0.69) |  | -0.67 (-2.04, 0.69) |
|  | | | |
| TF-CBT vs. Counselling | -0.43 (-1.10, 0.22) | -0.43 (-1.10, 0.22) |  |
| non-TF-CBT vs. Counselling | -0.13 (-1.25, 1.02) |  | -0.13 (-1.25, 1.02) |
| EMDR vs. Counselling | -0.83 (-1.95, 0.31) |  | -0.83 (-1.95, 0.31) |
| Present-centered therapy vs. Counselling | 0.15 (-1.11, 1.43) |  | 0.15 (-1.11, 1.43) |
| Combined somatic/cognitive therapies vs. Counselling | -0.88 (-2.62, 0.91) |  | -0.88 (-2.62, 0.91) |
| IPT vs. Counselling | -0.09 (-1.70, 1.51) |  | -0.09 (-1.70, 1.51) |
| Couple intervention vs. Counselling | -1.74 (-3.54, 0.06) |  | -1.74 (-3.54, 0.06) |
| Self-help with support vs. Counselling | -0.96 (-2.09, 0.15) |  | -0.96 (-2.09, 0.15) |
| Self-help without support vs. Counselling | -0.89 (-2.39, 0.60) |  | -0.89 (-2.39, 0.60) |
| Family therapy vs. Counselling | 0.45 (-1.08, 1.97) |  | 0.45 (-1.08, 1.97) |
| Behavioural therapy vs. Counselling | -0.89 (-2.17, 0.38) |  | -0.89 (-2.17, 0.38) |
|  | | | |
| non-TF-CBT vs. TF-CBT | 0.30 (-0.60, 1.24) | 1.11 (-0.36, 2.57) | -0.15 (-1.23, 0.94) |
| EMDR vs. TF-CBT | -0.40 (-1.30, 0.54) |  | -0.40 (-1.30, 0.54) |
| Present-centered therapy vs. TF-CBT | 0.58 (-0.49, 1.68) | 0.87 (-0.53, 2.27) | 0.15 (-1.45, 1.85) |
| Combined somatic/cognitive therapies vs. TF-CBT | -0.45 (-2.06, 1.22) |  | -0.45 (-2.06, 1.22) |
| IPT vs. TF-CBT | 0.34 (-1.11, 1.79) |  | 0.34 (-1.11, 1.79) |
| Couple intervention vs. TF-CBT | -1.31 (-2.98, 0.37) |  | -1.31 (-2.98, 0.37) |
| Self-help with support vs. TF-CBT | -0.53 (-1.43, 0.37) | 0.07 (-1.20, 1.34) | -1.18 (-2.50, 0.14) |
| Self-help without support vs. TF-CBT | -0.46 (-1.79, 0.89) |  | -0.46 (-1.79, 0.89) |
| Family therapy vs. TF-CBT | 0.88 (-0.49, 2.26) |  | 0.88 (-0.49, 2.26) |
| Behavioural therapy vs. TF-CBT | -0.45 (-1.54, 0.63) |  | -0.45 (-1.54, 0.63) |
|  | | | |
| EMDR vs. non-TF-CBT | -0.70 (-1.69, 0.30) | 0.02 (-1.14, 1.19) | -1.59 (-2.92, -0.24) |
| Present-centered therapy vs. non-TF-CBT | 0.28 (-0.78, 1.33) | 0.04 (-1.24, 1.31) | 0.76 (-1.03, 2.45) |
| Combined somatic/cognitive therapies vs. non-TF-CBT | -0.74 (-2.43, 0.94) |  | -0.74 (-2.43, 0.94) |
| IPT vs. non-TF-CBT | 0.04 (-1.64, 1.69) |  | 0.04 (-1.64, 1.69) |
| Couple intervention vs. non-TF-CBT | -1.60 (-3.51, 0.26) |  | -1.60 (-3.51, 0.26) |
| Self-help with support vs. non-TF-CBT | -0.83 (-2.01, 0.30) |  | -0.83 (-2.01, 0.30) |
| Self-help without support vs. non-TF-CBT | -0.76 (-2.14, 0.60) |  | -0.76 (-2.14, 0.60) |
| Family therapy vs. non-TF-CBT | 0.58 (-1.03, 2.17) |  | 0.58 (-1.03, 2.17) |
| Behavioural therapy vs. non-TF-CBT | -0.75 (-2.13, 0.58) |  | -0.75 (-2.13, 0.58) |
|  | | | |
| Present-centered therapy vs. EMDR | 0.97 (-0.31, 2.24) |  | 0.97 (-0.31, 2.24) |
| Combined somatic/cognitive therapies vs. EMDR | -0.05 (-1.42, 1.32) | -0.05 (-1.42, 1.32) |  |
| IPT vs. EMDR | 0.73 (-0.88, 2.33) |  | 0.73 (-0.88, 2.33) |
| Couple intervention vs. EMDR | -0.91 (-2.78, 0.93) |  | -0.91 (-2.78, 0.93) |
| Self-help with support vs. EMDR | -0.14 (-1.31, 0.99) |  | -0.14 (-1.31, 0.99) |
| Self-help without support vs. EMDR | -0.06 (-1.57, 1.41) |  | -0.06 (-1.57, 1.41) |
| Family therapy vs. EMDR | 1.28 (-0.27, 2.79) |  | 1.28 (-0.27, 2.79) |
| Behavioural therapy vs. EMDR | -0.06 (-1.35, 1.20) |  | -0.06 (-1.35, 1.20) |
|  | | | |
| Combined somatic/cognitive therapies vs. Present-centered therapy | -1.02 (-2.86, 0.85) |  | -1.02 (-2.86, 0.85) |
| IPT vs. Present-centered therapy | -0.24 (-2.04, 1.54) |  | -0.24 (-2.04, 1.54) |
| Couple intervention vs. Present-centered therapy | -1.88 (-3.88, 0.09) |  | -1.88 (-3.88, 0.09) |
| Self-help with support vs. Present-centered therapy | -1.11 (-2.47, 0.22) |  | -1.11 (-2.47, 0.22) |
| Self-help without support vs. Present-centered therapy | -1.03 (-2.64, 0.56) |  | -1.03 (-2.64, 0.56) |
| Family therapy vs. Present-centered therapy | 0.30 (-1.43, 2.00) |  | 0.30 (-1.43, 2.00) |
| Behavioural therapy vs. Present-centered therapy | -1.03 (-2.54, 0.45) |  | -1.03 (-2.54, 0.45) |
|  | | | |
| IPT vs. Combined somatic/cognitive therapies | 0.78 (-1.33, 2.86) |  | 0.78 (-1.33, 2.86) |
| Couple intervention vs. Combined somatic/cognitive therapies | -0.86 (-3.19, 1.42) |  | -0.86 (-3.19, 1.42) |
| Self-help with support vs. Combined somatic/cognitive therapies | -0.09 (-1.89, 1.67) |  | -0.09 (-1.89, 1.67) |
| Self-help without support vs. Combined somatic/cognitive therapies | -0.01 (-2.05, 1.99) |  | -0.01 (-2.05, 1.99) |
| Family therapy vs. Combined somatic/cognitive therapies | 1.33 (-0.73, 3.36) |  | 1.33 (-0.73, 3.36) |
| Behavioural therapy vs. Combined somatic/cognitive therapies | -0.01 (-1.90, 1.83) |  | -0.01 (-1.90, 1.83) |
|  | | | |
| Couple intervention vs. IPT | -1.65 (-3.81, 0.52) |  | -1.65 (-3.81, 0.52) |
| Self-help with support vs. IPT | -0.88 (-2.50, 0.74) |  | -0.88 (-2.50, 0.74) |
| Self-help without support vs. IPT | -0.80 (-2.71, 1.11) |  | -0.80 (-2.71, 1.11) |
| Family therapy vs. IPT | 0.54 (-1.35, 2.43) |  | 0.54 (-1.35, 2.43) |
| Behavioural therapy vs. IPT | -0.80 (-2.47, 0.89) |  | -0.80 (-2.47, 0.89) |
|  | | | |
| Self-help with support vs. Couple intervention | 0.77 (-1.10, 2.63) |  | 0.77 (-1.10, 2.63) |
| Self-help without support vs. Couple intervention | 0.85 (-1.26, 2.97) |  | 0.85 (-1.26, 2.97) |
| Family therapy vs. Couple intervention | 2.19 (0.08, 4.30) |  | 2.19 (0.08, 4.30) |
| Behavioural therapy vs. Couple intervention | 0.85 (-1.09, 2.78) |  | 0.85 (-1.09, 2.78) |
|  | | | |
| Self-help without support vs. Self-help with support | 0.07 (-1.11, 1.27) | -0.08 (-1.46, 1.29) | 0.48 (-1.85, 2.91) |
| Family therapy vs. Self-help with support | 1.41 (-0.12, 2.96) |  | 1.41 (-0.12, 2.96) |
| Behavioural therapy vs. Self-help with support | 0.08 (-1.21, 1.37) |  | 0.08 (-1.21, 1.37) |
|  | | | |
| Family therapy vs. Self-help without support | 1.34 (-0.50, 3.19) |  | 1.34 (-0.50, 3.19) |
| Behavioural therapy vs. Self-help without support | 0.00 (-1.64, 1.64) |  | 0.00 (-1.64, 1.64) |
|  | | | |
| Behavioural therapy vs. Family therapy | -1.34 (-2.94, 0.27) |  | -1.34 (-2.94, 0.27) |
| CBT: cognitive behavioural therapy; CI: confidence intervals; CrI: credible intervals; EMDR: eye movement desensitisation and reprocessing; SMD: standardised mean difference; TF: trauma-focused | | | |

## C. Dichotomous remission at treatment endpoint

All NMA estimates are reported based on the results from the random effects model that assumes consistency (Dias *et al.* 2013a); the direct and indirect estimates are reported based on results given by the node-split models (Dias *et al.* 2013b). Direct and indirect estimates are presented when available.

Positive values favour first intervention in the comparison.

| **Comparison** | **Effect: log-odds ratio (LOR)** | | |
| --- | --- | --- | --- |
|  | **NMA**  **median LOR (95% CrI)** | **Direct**  **median LOR (95% CrI)** | **Indirect**  **median LOR (95% CrI)** |
| Attention placebo vs. Waitlist | 1.08 (-1.97, 4.24) |  | 1.08 (-1.97, 4.24) |
| Relaxation vs. Waitlist | 2.64 (0.77, 4.59) |  | 2.64 (0.77, 4.59) |
| Psychoeducation vs. Waitlist | -0.74 (-4.66, 3.07) |  | -0.74 (-4.66, 3.07) |
| Counselling vs. Waitlist | 1.33 (0.20, 2.51) | 1.59 (-0.52, 3.83) | 1.36 (-0.26, 3.01) |
| TF-CBT vs. Waitlist | 2.45 (1.79, 3.19) | 2.33 (1.76, 3.00) | 2.15 (0.44, 3.83) |
| non-TF-CBT vs. Waitlist | 3.01 (1.31, 4.84) | 3.20 (-0.04, 6.40) | 2.90 (0.96, 4.90) |
| EMDR vs. Waitlist | 3.36 (2.04, 4.84) | 2.95 (1.04, 4.91) | 3.89 (1.89, 6.22) |
| IPT vs. Waitlist | 2.52 (0.71, 4.40) | 2.15 (-0.65, 5.22) | 2.83 (0.35, 5.39) |
| Present-centred therapy vs. Waitlist | 2.48 (0.75, 4.36) | 2.48 (0.75, 4.36) |  |
| Psychodynamic therapy vs. Waitlist | 4.58 (1.87, 7.57) | 4.58 (1.87, 7.57) |  |
| Couple intervention vs. Waitlist | 2.12 (-0.51, 4.83) | 2.12 (-0.51, 4.83) |  |
| Self-help with support vs. Waitlist | 1.76 (0.03, 3.49) | 1.76 (0.03, 3.49) |  |
| Self-help without support vs. Waitlist | 1.50 (-0.16, 3.32) | 1.50 (-0.16, 3.32) |  |
| SSRI vs. Waitlist | 1.39 (-0.45, 3.42) |  | 1.39 (-0.45, 3.42) |
| TF-CBT + SSRI vs. Waitlist | 1.63 (-0.61, 4.00) |  | 1.63 (-0.61, 4.00) |
|  | | | |
| Relaxation vs. Attention placebo | 1.57 (-2.09, 5.16) |  | 1.57 (-2.09, 5.16) |
| Psychoeducation vs. Attention placebo | -1.82 (-6.86, 3.05) |  | -1.82 (-6.86, 3.05) |
| Counselling vs. Attention placebo | 0.26 (-3.07, 3.49) |  | 0.26 (-3.07, 3.49) |
| TF-CBT vs. Attention placebo | 1.37 (-1.79, 4.47) |  | 1.37 (-1.79, 4.47) |
| non-TF-CBT vs. Attention placebo | 2.20 (-1.39, 5.82) |  | 2.20 (-1.39, 5.82) |
| EMDR vs. Attention placebo | 2.28 (-1.09, 5.67) |  | 2.28 (-1.09, 5.67) |
| IPT vs. Attention placebo | 1.45 (-2.16, 5.01) |  | 1.45 (-2.16, 5.01) |
| Present-centred therapy vs. Attention placebo | 1.41 (-2.16, 4.96) |  | 1.41 (-2.16, 4.96) |
| Psychodynamic therapy vs. Attention placebo | 3.53 (-0.70, 7.72) |  | 3.53 (-0.70, 7.72) |
| Couple intervention vs. Attention placebo | 1.05 (-3.08, 5.13) |  | 1.05 (-3.08, 5.13) |
| Self-help with support vs. Attention placebo | 0.69 (-2.92, 4.18) |  | 0.69 (-2.92, 4.18) |
| Self-help without support vs. Attention placebo | 0.43 (-2.13, 3.02) | 0.43 (-2.13, 3.02) |  |
| SSRI vs. Attention placebo | 0.32 (-3.29, 3.94) |  | 0.32 (-3.29, 3.94) |
| TF-CBT + SSRI vs. Attention placebo | 0.55 (-3.25, 4.37) |  | 0.55 (-3.25, 4.37) |
|  | | | |
| Psychoeducation vs. Relaxation | -3.38 (-7.81, 0.85) |  | -3.38 (-7.81, 0.85) |
| Counselling vs. Relaxation | -1.31 (-3.44, 0.80) |  | -1.31 (-3.44, 0.80) |
| TF-CBT vs. Relaxation | -0.19 (-2.07, 1.69) | 0.23 (-2.49, 2.95) | -0.93 (-4.11, 2.15) |
| non-TF-CBT vs. Relaxation | 0.64 (-1.90, 3.31) |  | 0.64 (-1.90, 3.31) |
| EMDR vs. Relaxation | 0.72 (-1.12, 2.64) | 0.18 (-2.39, 2.78) | 1.43 (-1.44, 4.42) |
| IPT vs. Relaxation | -0.11 (-2.28, 2.03) | 0.33 (-2.35, 3.05) | -1.24 (-5.55, 3.05) |
| Present-centred therapy vs. Relaxation | -0.15 (-2.69, 2.43) |  | -0.15 (-2.69, 2.43) |
| Psychodynamic therapy vs. Relaxation | 1.95 (-1.42, 5.44) |  | 1.95 (-1.42, 5.44) |
| Couple intervention vs. Relaxation | -0.51 (-3.82, 2.77) |  | -0.51 (-3.82, 2.77) |
| Self-help with support vs. Relaxation | -0.87 (-3.47, 1.67) |  | -0.87 (-3.47, 1.67) |
| Self-help without support vs. Relaxation | -1.14 (-3.64, 1.45) |  | -1.14 (-3.64, 1.45) |
| SSRI vs. Relaxation | -1.24 (-3.65, 1.28) |  | -1.24 (-3.65, 1.28) |
| TF-CBT + SSRI vs. Relaxation | -1.01 (-3.80, 1.85) |  | -1.01 (-3.80, 1.85) |
|  | | | |
| Counselling vs. Psychoeducation | 2.07 (-1.89, 6.19) |  | 2.07 (-1.89, 6.19) |
| TF-CBT vs. Psychoeducation | 3.19 (-0.65, 7.20) |  | 3.19 (-0.65, 7.20) |
| non-TF-CBT vs. Psychoeducation | 4.02 (-0.19, 8.47) |  | 4.02 (-0.19, 8.47) |
| EMDR vs. Psychoeducation | 4.11 (0.11, 8.32) |  | 4.11 (0.11, 8.32) |
| IPT vs. Psychoeducation | 3.26 (-0.95, 7.61) |  | 3.26 (-0.95, 7.61) |
| Present-centred therapy vs. Psychoeducation | 3.24 (-0.94, 7.57) |  | 3.24 (-0.94, 7.57) |
| Psychodynamic therapy vs. Psychoeducation | 5.35 (0.64, 10.23) |  | 5.35 (0.64, 10.23) |
| Couple intervention vs. Psychoeducation | 2.86 (0.16, 5.81) | 2.86 (0.16, 5.81) |  |
| Self-help with support vs. Psychoeducation | 2.50 (-1.69, 6.79) |  | 2.50 (-1.69, 6.79) |
| Self-help without support vs. Psychoeducation | 2.24 (-1.87, 6.58) |  | 2.24 (-1.87, 6.58) |
| SSRI vs. Psychoeducation | 2.14 (-2.04, 6.58) |  | 2.14 (-2.04, 6.58) |
| TF-CBT + SSRI vs. Psychoeducation | 2.37 (-2.01, 6.98) |  | 2.37 (-2.01, 6.98) |
|  | | | |
| TF-CBT vs. Counselling | 1.12 (0.12, 2.15) | 1.12 (0.12, 2.15) |  |
| non-TF-CBT vs. Counselling | 1.94 (0.03, 4.01) | 2.61 (-0.63, 6.62) | 1.86 (-1.12, 5.25) |
| EMDR vs. Counselling | 2.03 (0.37, 3.79) |  | 2.03 (0.37, 3.79) |
| IPT vs. Counselling | 1.19 (-0.89, 3.26) |  | 1.19 (-0.89, 3.26) |
| Present-centred therapy vs. Counselling | 1.16 (-0.79, 3.18) |  | 1.16 (-0.79, 3.18) |
| Psychodynamic therapy vs. Counselling | 3.25 (0.29, 6.43) |  | 3.25 (0.29, 6.43) |
| Couple intervention vs. Counselling | 0.79 (-2.10, 3.71) |  | 0.79 (-2.10, 3.71) |
| Self-help with support vs. Counselling | 0.43 (-1.66, 2.48) |  | 0.43 (-1.66, 2.48) |
| Self-help without support vs. Counselling | 0.17 (-1.81, 2.25) |  | 0.17 (-1.81, 2.25) |
| SSRI vs. Counselling | 0.06 (-1.97, 2.24) |  | 0.06 (-1.97, 2.24) |
| TF-CBT + SSRI vs. Counselling | 0.29 (-2.08, 2.80) |  | 0.29 (-2.08, 2.80) |
|  | | | |
| non-TF-CBT vs. TF-CBT | 0.82 (-0.95, 2.75) | 0.62 (-2.21, 3.49) | 0.84 (-1.97, 4.04) |
| EMDR vs. TF-CBT | 0.91 (-0.46, 2.35) | 5.37 (1.91, 10.09) | 0.08 (-1.31, 1.45) |
| IPT vs. TF-CBT | 0.07 (-1.76, 1.92) | 0.11 (-2.54, 2.77) | -0.31 (-3.31, 2.87) |
| Present-centred therapy vs. TF-CBT | 0.03 (-1.72, 1.85) | 0.21 (-2.35, 2.75) | 1.05 (-1.78, 4.35) |
| Psychodynamic therapy vs. TF-CBT | 2.13 (-0.70, 5.16) |  | 2.13 (-0.70, 5.16) |
| Couple intervention vs. TF-CBT | -0.32 (-3.08, 2.43) |  | -0.32 (-3.08, 2.43) |
| Self-help with support vs. TF-CBT | -0.69 (-2.59, 1.13) |  | -0.69 (-2.59, 1.13) |
| Self-help without support vs. TF-CBT | -0.95 (-2.70, 0.88) | -3.24 (-5.15, -1.48) | 3.36 (0.79, 7.03) |
| SSRI vs. TF-CBT | -1.06 (-2.86, 0.85) | -1.79 (-4.13, 0.53) | 0.28 (-2.74, 3.31) |
| TF-CBT + SSRI vs. TF-CBT | -0.82 (-3.01, 1.42) | -0.82 (-3.01, 1.42) |  |
|  | | | |
| EMDR vs. non-TF-CBT | 0.08 (-2.22, 2.33) |  | 0.08 (-2.22, 2.33) |
| IPT vs. non-TF-CBT | -0.75 (-3.40, 1.76) |  | -0.75 (-3.40, 1.76) |
| Present-centred therapy vs. non-TF-CBT | -0.79 (-2.79, 1.15) | -0.41 (-2.93, 2.12) | -1.44 (-4.93, 1.93) |
| Psychodynamic therapy vs. non-TF-CBT | 1.31 (-2.10, 4.74) |  | 1.31 (-2.10, 4.74) |
| Couple intervention vs. non-TF-CBT | -1.15 (-4.50, 2.11) |  | -1.15 (-4.50, 2.11) |
| Self-help with support vs. non-TF-CBT | -1.51 (-4.19, 0.95) |  | -1.51 (-4.19, 0.95) |
| Self-help without support vs. non-TF-CBT | -1.77 (-4.33, 0.74) |  | -1.77 (-4.33, 0.74) |
| SSRI vs. non-TF-CBT | -1.88 (-4.50, 0.69) |  | -1.88 (-4.50, 0.69) |
| TF-CBT + SSRI vs. non-TF-CBT | -1.64 (-4.55, 1.19) |  | -1.64 (-4.55, 1.19) |
|  | | | |
| IPT vs. EMDR | -0.83 (-3.02, 1.27) |  | -0.83 (-3.02, 1.27) |
| Present-centred therapy vs. EMDR | -0.87 (-3.10, 1.34) |  | -0.87 (-3.10, 1.34) |
| Psychodynamic therapy vs. EMDR | 1.22 (-1.90, 4.46) |  | 1.22 (-1.90, 4.46) |
| Couple intervention vs. EMDR | -1.23 (-4.28, 1.74) |  | -1.23 (-4.28, 1.74) |
| Self-help with support vs. EMDR | -1.60 (-3.89, 0.54) |  | -1.60 (-3.89, 0.54) |
| Self-help without support vs. EMDR | -1.86 (-4.06, 0.33) |  | -1.86 (-4.06, 0.33) |
| SSRI vs. EMDR | -1.96 (-3.86, -0.05) | -0.97 (-3.58, 1.60) | -3.04 (-5.87, -0.30) |
| TF-CBT + SSRI vs. EMDR | -1.73 (-4.19, 0.72) |  | -1.73 (-4.19, 0.72) |
|  | | | |
| Present-centred therapy vs. IPT | -0.03 (-2.53, 2.52) |  | -0.03 (-2.53, 2.52) |
| Psychodynamic therapy vs. IPT | 2.07 (-1.26, 5.54) |  | 2.07 (-1.26, 5.54) |
| Couple intervention vs. IPT | -0.39 (-3.66, 2.85) |  | -0.39 (-3.66, 2.85) |
| Self-help with support vs. IPT | -0.76 (-3.30, 1.75) |  | -0.76 (-3.30, 1.75) |
| Self-help without support vs. IPT | -1.02 (-3.47, 1.53) |  | -1.02 (-3.47, 1.53) |
| SSRI vs. IPT | -1.13 (-3.62, 1.48) |  | -1.13 (-3.62, 1.48) |
| TF-CBT + SSRI vs. IPT | -0.90 (-3.69, 2.01) |  | -0.90 (-3.69, 2.01) |
|  | | | |
| Psychodynamic therapy vs. Present-centred therapy | 2.10 (-1.23, 5.53) |  | 2.10 (-1.23, 5.53) |
| Couple intervention vs. Present-centred therapy | -0.36 (-3.61, 2.85) |  | -0.36 (-3.61, 2.85) |
| Self-help with support vs. Present-centred therapy | -0.72 (-3.29, 1.70) |  | -0.72 (-3.29, 1.70) |
| Self-help without support vs. Present-centred therapy | -0.98 (-3.45, 1.49) |  | -0.98 (-3.45, 1.49) |
| SSRI vs. Present-centred therapy | -1.09 (-3.62, 1.48) |  | -1.09 (-3.62, 1.48) |
| TF-CBT + SSRI vs. Present-centred therapy | -0.86 (-3.67, 1.98) |  | -0.86 (-3.67, 1.98) |
|  | | | |
| Couple intervention vs. Psychodynamic therapy | -2.46 (-6.40, 1.38) |  | -2.46 (-6.40, 1.38) |
| Self-help with support vs. Psychodynamic therapy | -2.83 (-6.23, 0.40) |  | -2.83 (-6.23, 0.40) |
| Self-help without support vs. Psychodynamic therapy | -3.09 (-6.46, 0.23) |  | -3.09 (-6.46, 0.23) |
| SSRI vs. Psychodynamic therapy | -3.19 (-6.64, 0.22) |  | -3.19 (-6.64, 0.22) |
| TF-CBT + SSRI vs. Psychodynamic therapy | -2.96 (-6.64, 0.67) |  | -2.96 (-6.64, 0.67) |
|  | | | |
| Self-help with support vs. Couple intervention | -0.36 (-3.56, 2.78) |  | -0.36 (-3.56, 2.78) |
| Self-help without support vs. Couple intervention | -0.63 (-3.79, 2.62) |  | -0.63 (-3.79, 2.62) |
| SSRI vs. Couple intervention | -0.74 (-3.97, 2.64) |  | -0.74 (-3.97, 2.64) |
| TF-CBT + SSRI vs. Couple intervention | -0.50 (-3.95, 3.08) |  | -0.50 (-3.95, 3.08) |
|  | | | |
| Self-help without support vs. Self-help with support | -0.26 (-2.65, 2.26) |  | -0.26 (-2.65, 2.26) |
| SSRI vs. Self-help with support | -0.36 (-2.88, 2.30) |  | -0.36 (-2.88, 2.30) |
| TF-CBT + SSRI vs. Self-help with support | -0.14 (-2.94, 2.81) |  | -0.14 (-2.94, 2.81) |
|  | | | |
| SSRI vs. Self-help without support | -0.11 (-2.64, 2.46) |  | -0.11 (-2.64, 2.46) |
| TF-CBT + SSRI vs. Self-help without support | 0.13 (-2.71, 2.97) |  | 0.13 (-2.71, 2.97) |
|  | | | |
| TF-CBT + SSRI vs. SSRI | 0.23 (-2.02, 2.46) | 0.23 (-2.02, 2.46) |  |
| CBT: cognitive behavioural therapy; CI: confidence intervals; CrI: credible intervals; EMDR: eye movement desensitisation and reprocessing; LOR: log-odds ratio; SSRI: selective serotonine reuptake inhibitor; TF: trauma-focused | | | |
