# Supplementary material for: Cost-effectiveness of psychological treatments for post-traumatic stress disorder in adults
Source: PLoS One. 2020 Apr 30;15(4):e0232245. doi: 10.1371/journal.pone.0232245 (PMC7192458; doi:10.1371/journal.pone.0232245)
Supplement: S12 Appendix — (DOCX) [file pone.0232245.s019.docx]

# **Appendix 12: Results of the NICE guideline NMA**

## A. Changes in PTSD symptom scores between baseline and treatment endpoint

**Random effects model used**

**Posterior median standard deviation: 0.88 (95% CrI 0.73 to 1.10); residual deviance 157.3**

**No evidence of inconsistency detected**

| **Intervention** | **N** | **k** | **Mean SMD (95% CrI) vs waitlist** |
| --- | --- | --- | --- |
| Couple intervention | 22 | 1 | **-3.49 (-6.22 to -0.75)** |
| Metacognitive therapy | 10 | 1 | **-3.03 (-4.99 to -1.06)** |
| TF-CBT mixed | 28 | 1 | **-2.83 (-4.70 to -0.98)** |
| TF-CBT group >12 sessions | 42 | 1 | **-2.38 (-4.34 to -0.46)** |
| TF-CBT individual <8 sessions | 160 | 5 | **-2.26 (-3.23 to -1.30)** |
| Psychoeducation | 152 | 2 | **-2.02 (-4.01 to -0.02)** |
| EMDR | 260 | 11 | **-1.98 (-2.59 to -1.37)** |
| Combined somatic/cognitive therapies | 237 | 4 | **-1.67 (-2.59 to -0.75)** |
| Resilience-oriented treatment | 20 | 1 | -1.62 (-3.50 to 0.25) |
| Self-help with support | 198 | 5 | **-1.46 (-2.28 to -0.64)** |
| TF-CBT individual 8-12 sessions | 443 | 13 | **-1.43 (-2.00 to -0.88)** |
| Present-centered therapy | 99 | 3 | **-1.32 (-2.33 to -0.33)** |
| Behavioural therapy | 47 | 2 | -1.20 (-2.52 to 0.11) |
| non-TF-CBT | 209 | 7 | **-1.19 (-1.90 to -0.49)** |
| IPT | 55 | 2 | -1.16 (-2.47 to 0.13) |
| TF-CBT individual 8-12 sessions + SSRI | 115 | 3 | -1.06 (-2.17 to 0.02) |
| SSRI | 166 | 5 | **-1.02 (-1.94 to -0.11)** |
| TF-CBT individual >12 sessions | 173 | 6 | **-0.94 (-1.71 to -0.17)** |
| Self-help without support | 335 | 11 | **-0.91 (-1.64 to -0.18)** |
| Counselling | 278 | 9 | **-0.70 (-1.39 to -0.01)** |
| Relaxation | 25 | 2 | -0.67 (-2.07 to 0.69) |
| TF-CBT group 8-12 sessions | 57 | 3 | -0.65 (-1.75 to 0.45) |
| Attention placebo | 221 | 9 | -0.39 (-1.36 to 0.59) |
| Family therapy | 72 | 1 | 0.15 (-1.66 to 1.94) |
| Waitlist | 1425 | 46 | Reference |
| Attention bias modification | 83 | 3 | 2.14 (0.73 to 3.59) |
| CrI: credible intervals; EMDR: eye movement desensitisation reprocessing; IPT: interpersonal psychotherapy; SMD: standardised mean difference; SSRI: selective serotonin reuptake inhibitor; TF-CBT: trauma-focused cognitive behavioural therapy  k: number of randomised controlled trials (RCTs) that assessed each intervention; N: number randomised to each treatment across RCTs  Negative values indicate a better effect for the intervention compared with the reference treatment (waitlist).  **In bold** effects where the 95% CrI do not cross the line of no effect (SMD=0) | | | |

## B. Changes in PTSD symptom scores between baseline and 1-4 month follow-up

**Random effects model used**

**Posterior median standard deviation: 0.65 (95% CrI 0.41 to 1.13); residual deviance 51.37**

**Evidence of inconsistency detected**

| **Intervention** | **N** | **K** | **Mean SMD (95% CrI) vs waitlist** |
| --- | --- | --- | --- |
| Couple intervention | 21 | 1 | **-1.93 (-3.84 to -0.03)** |
| Self-help with support | 85 | 3 | **-1.22 (-2.17 to -0.26)** |
| Self-help without support | 40 | 2 | -1.17 (-2.60 to 0.30) |
| Combined somatic/cognitive therapies | 23 | 1 | -1.16 (-2.95 to 0.61) |
| EMDR | 121 | 4 | **-1.13 (-2.06 to -0.19)** |
| TF-CBT individual 8-12 sessions | 400 | 7 | **-0.86 (-1.52 to -0.21)** |
| TF-CBT individual >12 sessions | 50 | 2 | -0.75 (-2.24 to 0.72) |
| TF-CBT individual <8 sessions | 303 | 4 | -0.52 (-1.33 to 0.30) |
| non-TF-CBT | 123 | 4 | -0.45 (-1.53 to 0.67) |
| Psychoeducation | 183 | 3 | -0.40 (-1.51 to 0.71) |
| IPT | 32 | 1 | -0.39 (-1.92 to 1.14) |
| Counselling | 205 | 4 | -0.30 (-1.29 to 0.69) |
| Present-centered therapy | 70 | 2 | -0.17 (-1.67 to 1.35) |
| Attention placebo | 44 | 2 | -0.01 (-1.50 to 1.52) |
| Waitlist | 383 | 11 | reference |
| CrI: credible intervals; EMDR: eye movement desensitisation reprocessing; IPT: interpersonal psychotherapy; SMD: standardised mean difference; TF-CBT: trauma-focused cognitive behavioural therapy  k: number of randomised controlled trials (RCTs) that assessed each intervention; N: number randomised to each treatment across RCTs  Negative values indicate a better effect for the intervention compared with the reference treatment (waitlist).  **In bold** effects where the 95% CrI do not cross the line of no effect (SMD=0) | | | |

## C. Dichotomous remission at treatment endpoint

**Random effects model used**

**Posterior median standard deviation: 1.00 (95% CrI 0.51 to 1.74); residual deviance 78.51**

**Evidence of inconsistency detected**

| **Intervention** | **N** | **k** | **Mean LOR (95% CrI) vs waitlist** |
| --- | --- | --- | --- |
| Psychodynamic therapy | 49 | 1 | **4.60 (1.84 to 7.53)** |
| non-TF-CBT | 65 | 2 | **3.66 (1.80 to 5.73)** |
| TF-CBT individual 8-12 sessions | 300 | 8 | **3.39 (2.33 to 4.59)** |
| TF-CBT individual <8 sessions | 30 | 2 | **3.37 (0.67 to 6.95)** |
| EMDR | 132 | 5 | **3.35 (1.98 to 4.82)** |
| Relaxation | 57 | 2 | **3.02 (1.13 to 4.98)** |
| IPT | 72 | 2 | **2.96 (1.10 to 4.91)** |
| Present-centered therapy | 75 | 2 | **2.58 (0.78 to 4.50)** |
| TF-CBT group >12 sessions | 22 | 1 | 2.54 (-0.25 to 5.45) |
| TF-CBT mixed | 36 | 1 | 2.43 (-0.02 to 4.94) |
| TF-CBT individual 8-12 sessions + SSRI | 57 | 1 | **2.38 (0.05 to 4.85)** |
| TF-CBT individual >12 sessions | 146 | 6 | **2.25 (1.12 to 3.46)** |
| Couple intervention | 49 | 2 | 2.14 (-0.47 to 4.79) |
| SSRI | 87 | 2 | **1.95 (0.01 to 4.01)** |
| Self-help without support | 74 | 3 | **1.79 (0.11 to 3.65)** |
| Self-help with support | 105 | 2 | **1.76 (0.08 to 3.48)** |
| Counselling | 150 | 6 | **1.71 (0.51 to 2.98)** |
| Attention placebo | 23 | 1 | 1.38 (-1.63 to 4.56) |
| TF-CBT group 8-12 sessions | 67 | 3 | 0.93 (-0.74 to 2.53) |
| Psychoeducation | 28 | 1 | -0.76 (-4.61 to 2.99) |
| Waitlist | 625 | 23 | Reference |
| CrI: credible intervals; EMDR: eye movement desensitisation reprocessing; IPT: interpersonal psychotherapy; LOR: log-odds ratio; SSRI: selective serotonin reuptake inhibitor; TF-CBT: trauma-focused cognitive behavioural therapy  k: number of randomised controlled trials (RCTs) that assessed each intervention; N: number randomised to each treatment across RCTs  Positive values indicate a better effect for the intervention compared with the reference treatment (waitlist).  **In bold** effects where the 95% CrI do not cross the line of no effect (LOR=0) | | | |
