# Supplementary material for: Cost-effectiveness of psychological treatments for post-traumatic stress disorder in adults
Source: PLoS One. 2020 Apr 30;15(4):e0232245. doi: 10.1371/journal.pone.0232245 (PMC7192458; doi:10.1371/journal.pone.0232245)

# **Appendix 13: Pairwise sub-analyses**

## A. Trauma-focused CBT versus waitlist – PTSD symptom scores between baseline and treatment endpoint: Sub-analysis by specific TF-CBT intervention


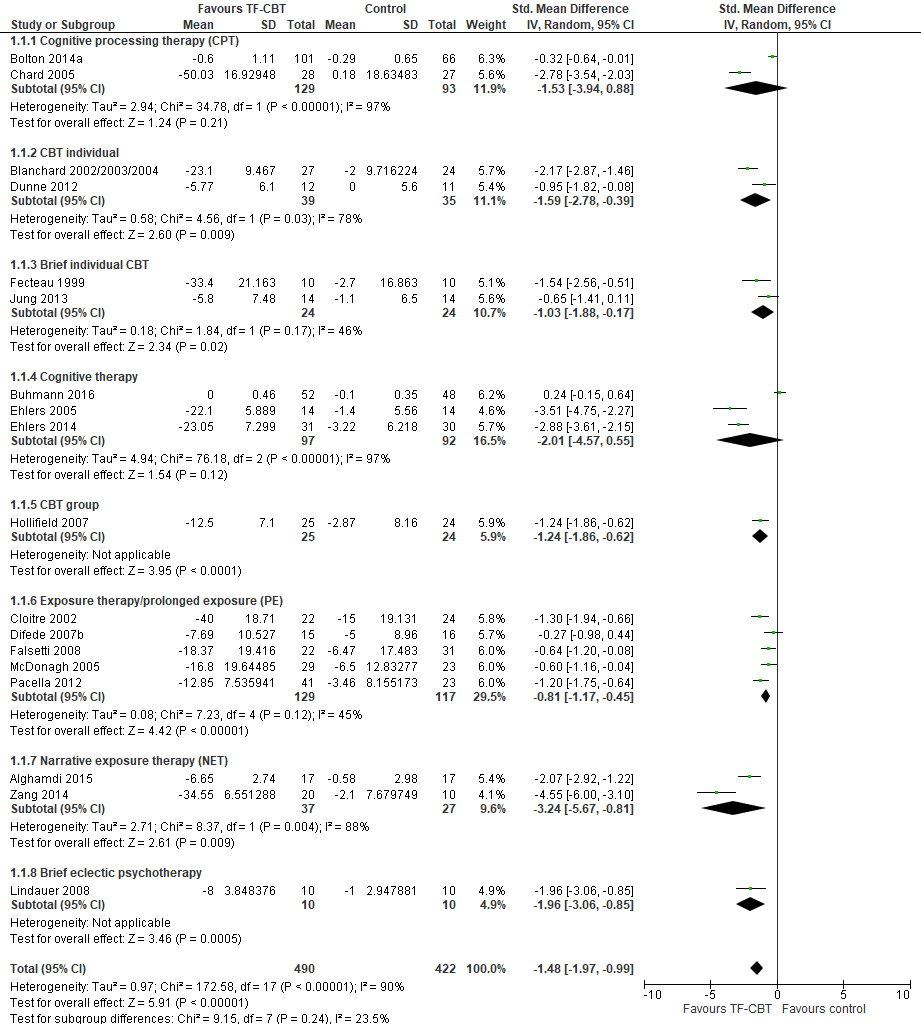


## B. Trauma-focused CBT versus waitlist – PTSD symptom scores between baseline and treatment endpoint: Sub-analysis by method of analysis (intention to treat [ITT] or completer)


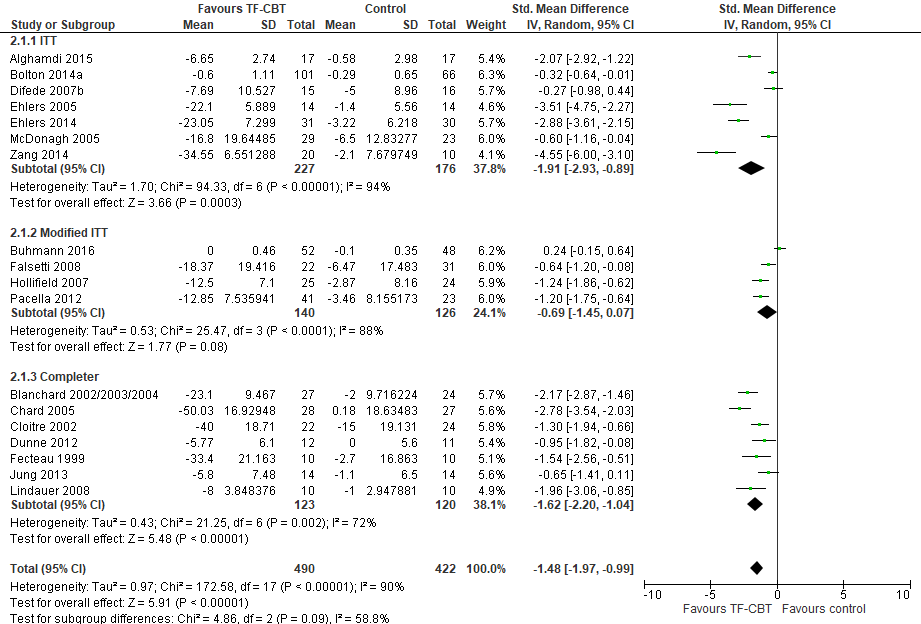


## C. Trauma-focused CBT versus waitlist – PTSD symptom scores between baseline and treatment endpoint: Sub-analysis by multiplicity of trauma


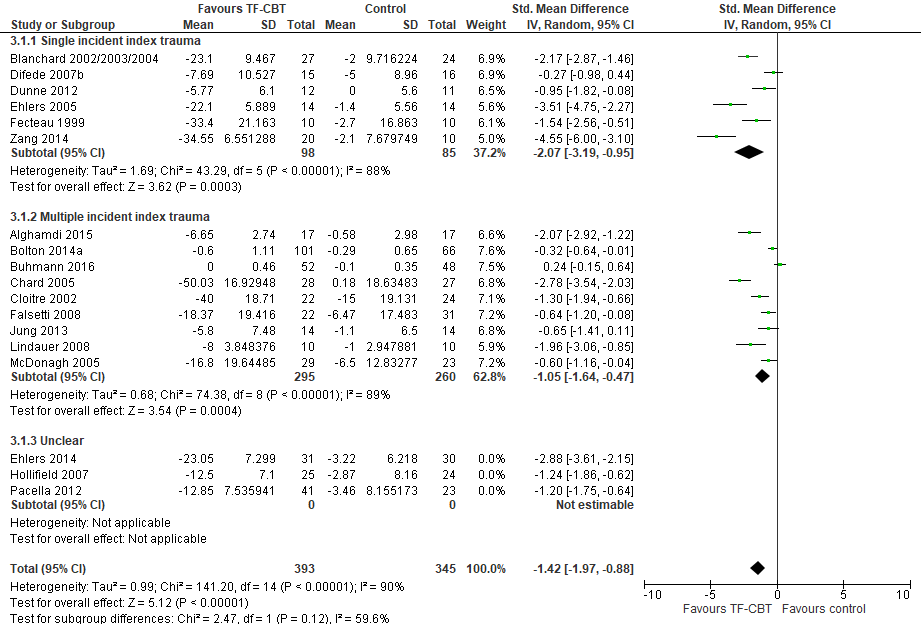

Supplement: S13 Appendix — (DOCX) [file pone.0232245.s020.docx]
