# Supplementary material for: Cost-effectiveness of psychological treatments for post-traumatic stress disorder in adults
Source: PLoS One. 2020 Apr 30;15(4):e0232245. doi: 10.1371/journal.pone.0232245 (PMC7192458; doi:10.1371/journal.pone.0232245)
Supplement: S14 Appendix — (DOCX) [file pone.0232245.s021.docx]

# **Appendix 14: References in the online supplementary material**

**Brooks SP, Gelman A** (1998). Alternative methods for monitoring convergence of iterative simulations. *Journal of Computational and Graphical Statistics* **7**, 434-455.

**Chinn S** (2000). A simple method for converting an odds ratio to effect size for use in meta-analysis. *Statistics in Medicine* **19**, 3127-3131.

**Cohen J** (1969). *Statistical power analysis for the behavioral sciences.* Academic Press: New York.

**Cooper H, Hedges LV, Valentine JC** (2009). *The Handbook of Research Synthesis and Meta-analysis.* Russel Sage Foundation: New York.

**Dempster A** (1997). The direct use of likelihood for significance testing. *Statistics and Computing* **7**, 247-252.

**Dias S, Ades AE, Welton NJ, Jansen JP, Sutton AJ** (2018). *Network Meta-analysis for Decision-Making.* Wiley: Hoboken NJ.

**Dias S, Sutton AJ, Ades AE, Welton NJ** (2013a). Evidence synthesis for decision making 2: a generalized linear modeling framework for pairwise and network meta-analysis of randomized controlled trials. *Medical Decision Making* **33**, 607-617.

**Dias S, Welton NJ, Caldwell DM, Ades AE** (2010). Checking consistency in mixed treatment comparison meta-analysis. *Statistics in Medicine* **29**, 932-944.

**Dias S, Welton NJ, Sutton AJ, Caldwell DM, Lu G, Ades AE** (2013b). Evidence synthesis for decision making 4: inconsistency in networks of evidence based on randomized controlled trials. *Medical Decision Making* **33**, 641-656.

**Lunn DJ, Thomas A, Best N, Spiegelhalter D** (2000). WinBUGS-A Bayesian modelling framework: Concepts, structure, and extensibility. *Statistics and Computing* **10**, 325-337.

**Mavranezouli I, Megnin-Viggars O, Grey N, Bhutani G, Leach J, Daly C, Dias S, Welton NJ, Katona C, El-Leithy S, Greenberg N, Stockton S, Pilling S** (under review). Cost effectiveness of psychological interventions for post-traumatic stress disorder in adults. *PLOS One,* under review.

**Spiegelhalter D, Thomas A, Best N, Lunn DJ** (2003). *WinBUGS user manual: version 1.4*. MRC Biostatistics Unit: Cambridge.

**Spiegelhalter DJ, Best NG, Carlin BP, van der Linde A** (2002). Bayesian measures of model complexity and fit. *Journal of the Royal Statistical Society: Series B* **64**, 583-616.

**van Valkenhoef G, Dias S, Ades AE, Welton NJ** (2016). Automated generation of node-splitting models for assessment of inconsistency in network meta-analysis. *Research Synthesis Methods* **7**, 80-93.

**van Valkenhoef G, Kuiper J** (2016). *gemtc: Network Meta-Analysis Using Bayesian Methods. R package version 0.8-2*. https://CRAN.R-project.org/package=gemtc. Accessed 20 June 2018
