# Supplementary material for: Cost-effectiveness of psychological treatments for post-traumatic stress disorder in adults
Source: PLoS One. 2020 Apr 30;15(4):e0232245. doi: 10.1371/journal.pone.0232245 (PMC7192458; doi:10.1371/journal.pone.0232245)
Supplement: Attachment — Submitted filename: PTSD adult HE model Responses to reviewers R2.docx [file pone.0232245.s023.docx]

**Responses to reviewers' comments:**

Reviewer #1

The authors' revisions are responsive and their rebuttals are generally persuasive. A few points of clarification would be helpful.

We thank the reviewer for their useful comments.

1. The much lower percentages of studies requiring a PTSD diagnosis evaluating somatic and psychoeducation interventions (25-33%) vs. in EMDR or TF-CBT (75-78%), and the very few studies (3-4) for each of the former modalities, makes conclusions for those modalities quite uncertain. From their NMA, can the authors provide any information (briefly) to help readers determine if the PTSD symptom severity was comparable at baseline for those studies versus for the studies of TF-CBT and EMDR? This would be helpful in judging whether this is really an apples-to-apples comparison of cost effectiveness that should favor somatic and psychoeducation modalities over TF-CBT.

Response: we agree that the very few studies for somatic and psychoeducation interventions make conclusions for those modalities quite uncertain, and this is reflected in the Discussion of the paper on NICE recommendations:

“Psychoeducation was shown to be cost-effective based on limited and inconclusive clinical evidence; therefore, it was not recommended as a stand-alone intervention, but as part of individual TF-CBT. Finally, the committee noted the evidence of high clinical and cost-effectiveness for combined somatic/cognitive therapies, but also considered their particularly limited evidence base beyond treatment endpoint and the lack of specific indications for these interventions, and decided not to recommend them but instead to make a recommendation for further research” (page 36, lines 365-371).

Regarding inclusion criteria on PTSD diagnosis versus clinically important PTSD symptoms: We have already acknowledged this issue as a potential factor contributing to the high heterogeneity of the NMA: “Both NMAs were characterised by high between-trial heterogeneity, which is likely to have been caused by heterogeneity across populations included in the trials considered in the NMAs, for example, in terms of the presence of a formal PTSD diagnosis, the severity, complexity and chronicity of PTSD symptoms…” (page 30, lines 187-190).

Unfortunately it was not possible to determine if severity was comparable across trials at baseline using the mean PTSD symptom severity scores, because the studies have used a range of different PTSD symptom scales and no mapping function that could allow comparison of severity levels across the scales is available. We have attempted to assess comparability of populations by looking at the % of the mean score over cut-off or the % of mean score out of the total score across studies, but we concluded that this was not reliable because it is expected that different symptom scales will have different levels of redundancy built in.

However, we note that the categorisation of diagnosis versus clinically important symptoms is based on inclusion criteria so it is possible that most of the participants in the ‘clinically important symptom’ studies could have a diagnosis, they were just not required to do so in order to participate in the trial, and this could well have been for pragmatic reasons to do with trial management rather than an indication of differing severity. It is also the case that for scales that are based closely on diagnostic criteria, e.g. PTSD Checklist (PCL), scoring above the clinical threshold may be regarded as comparable to receiving a diagnosis. The studies that evaluate combined somatic and cognitive therapies all use scales that are based on diagnostic criteria (e.g. PCL and Modified PTSD Symptom Scale [MPSS-SR]), and the majority of the studies evaluating psychoeducation also use scales based on diagnostic criteria (e.g. PCL and Davidson Trauma Scale).

We have now added the above points in the Discussion section (page 30, lines 199 to page 31, line 235).

2. The finding that self-help plus support had a relatively strong QUALY outcome and cost-effectiveness, with a relatively large number of studies most of which required a PTSD diagnosis, deserves a bit more highlighting in the Discussion.

Response: we have now added discussion on the cost-effectiveness of self-help with support (page 36, lines 350-58). We have covered discussion on the issue of formal diagnosis of PTSD vs clinically important PTSD symptoms and the potential impact on the comparability of populations regarding baseline symptom severity – please refer to our response to your previous comment.
